# Different adaptation strategies of two citrus scion/rootstock combinations in response to drought stress

**DOI:** 10.1371/journal.pone.0177993

**Published:** 2017-05-17

**Authors:** Joadson Dutra de Souza, Edson Mario de Andrade Silva, Mauricio Antônio Coelho Filho, Raphaël Morillon, Diego Bonatto, Fabienne Micheli, Abelmon da Silva Gesteira

**Affiliations:** 1 Universidade Estadual de Santa Cruz (UESC), Departamento de Ciências Biológicas (DCB), Centro de Biotecnologia e Genética (CBG), Rodovia Ilhéus-Itabuna, Ilhéus-BA, Brazil; 2 Embrapa Mandioca e Fruticultura, Departamento de Biologia Molecular, Rua Embrapa, s/n°, Cruz das Almas, Bahia, Brazil; 3 CIRAD, UMR AGAP, Montpellier, France; 4 Universidade Federal do Rio Grande do Sul (UFRGS), Departamento de Biologia Molecular e Biotecnologia, Centro de Biotecnologia, Avenida Bento Goncalves 9500–Predio 43421, Porto Alegre-RS, Brazil; Estacion Experimental del Zaidin, SPAIN

## Abstract

Scion/rootstock interaction is important for plant development and for breeding programs. In this context, polyploid rootstocks presented several advantages, mainly in relation to biotic and abiotic stresses. Here we analyzed the response to drought of two different scion/rootstock combinations presenting different polyploidy: the diploid (2x) and autotetraploid (4x) Rangpur lime (*Citrus limonia*, Osbeck) rootstocks grafted with 2x Valencia Delta sweet orange (*Citrus sinensis*) scions, named V/2xRL and V/4xRL, respectively. Based on previous gene expression data, we developed an interactomic approach to identify proteins involved in V/2xRL and V/4xRL response to drought. A main interactomic network containing 3,830 nodes and 97,652 edges was built from V/2xRL and V/4xRL data. Exclusive proteins of the V/2xRL and V/4xRL networks (2,056 and 1,001, respectively), as well as common to both networks (773) were identified. Functional clusters were obtained and two models of drought stress response for the V/2xRL and V/4xRL genotypes were designed. Even if the V/2xRL plant implement some tolerance mechanisms, the global plant response to drought was rapid and quickly exhaustive resulting in a general tendency to dehydration avoidance, which presented some advantage in short and strong drought stress conditions, but which, in long terms, does not allow the plant survival. At the contrary, the V/4xRL plants presented a response which strong impacts on development but that present some advantages in case of prolonged drought. Finally, some specific proteins, which presented high centrality on interactomic analysis were identified as good candidates for subsequent functional analysis of citrus genes related to drought response, as well as be good markers of one or another physiological mechanism implemented by the plants.

## Introduction

The citrus culture in Brazil occurs mainly in dryland, and for this reason, breeding citrus programs focused in the selection and use of scion-rootstock combinations with better responses to drought conditions [1–4]. Drought tolerance could be more or less intense and could be influenced by the stress duration or severity, the plant age or developmental stage, as well as by the competition with the neighbor plants [5]. Moreover, some authors affirm that most of the characteristics associated to drought tolerance can be an advantage under severe drought, but can have, under moderate drought, an opposite effect–and vice-versa [5]. The mechanisms developed by the plants subjected to drought can be divided in two categories: prevention of stress and tolerance to stress [6, 7]. Prevention occurs by the efficiency to absorb water by the radicular system, whether by high root deepening or by higher density [8]. The plant continue growing, albeit at a reduced rate, even in the absence of irrigation [5]. In the other hand, the mechanisms of tolerance aim to protect the cell of serious injuries when the mechanisms of prevention are not still sufficient. Then the plant develop some strategies as stomata closure to avoid water losses by transpiration, growth reduction and leaf senescence [9].

Strategies for drought tolerance are highly relevant in the case of rootstock selection and multiplication. In Brazil, the rootstock the most used is the Rangpur lime due to its characteristics in inducing high scion productivity, precocity and drought tolerance; this rootstock is used in most of the areas/states in Southern and Northeast of Brazil. The Rangpur lime presents high root growing, high root hydraulic conductivity, better water use capacity and lower stomatic conductance [10–12]. It is known that genotypes that maintain stomatic conductance under drought also maintain high growth level and have a higher mass accumulation [13]. Even the risk of deleterious symptoms related to stress duration in leaves exists, it is considered that the mass or supply accumulation could be a positive aspect for the plant recovery when rehydrated [14]. For this reason, in field conditions, plants grafted on Rangpur lime were considered more tolerant to drought [15, 16]. Another factor influencing the behavior of plants in relation to drought is the polyploidy [17–19]. In citrus, it has been shown that tetraploid plants (4x) cultivated in greenhouse and subjected to drought presented higher drought tolerance than the respective diploid plants (2x) [20–22]. Such behavior could be associated to morpho-physiological differences more favorable in the 4x plants, such as lower stomata density, deeper major roots and thicker surface roots, as well as, the existence of genes differentially expressed in roots and associated to abscisic acid production [20].

According to the strategy developed, different molecular and biochemical processes as well as different interaction between them occurred in the plant submitted to stress. In short, plant cells perceive stress stimulus by various sensors that in turn activate signaling pathways involving secondary messengers, plant hormones, signal transducers and transcriptional regulators [23, 24]. Multiple signals therefore converge to regulate stress-inducible genes that encode proteins and enzymes directly involved in stress metabolism, contributing to the specificity of the acclimation response to stress stimulus [25]. To better understand these interactions, as well as to identify key genes and proteins involved in these interactions, comprehensive studies called omics may be used. The omics are powerful approaches to identify key genes for important traits, to clarify events of physiological mechanisms and to reveal unknown metabolic pathways in crops. The data are analyzed by bioinformatics tools and many important genes, proteins, metabolites and metabolic pathways have been identified by these approaches [26]. In this context, increasingly, the interactomic (also called systems biology) approach appears as an important tool to support the elucidation of a biological system (or part of it), allowing the efficient exploration of high throughput data and the integration of information obtained using different molecular methods [27]. Interactomic uses the comparative-based concept of orthology for functional characterization and classification of molecules [28] and, in plants, Arabidopsis is generally used as the best model due to availability of large databanks of genes and proteins but mainly of a large amount of protein-protein interaction (PPI) data [29]. Here, we used the data published by [20] in interactomic analysis to identify proteins involved in plant response (different combination of scion/rootstock) to drought, and to build a model of the molecular and metabolic response of the different combination of scion/rootstock in relation to drought.

## Material and methods

### Initial data sets

**Fig 1:**
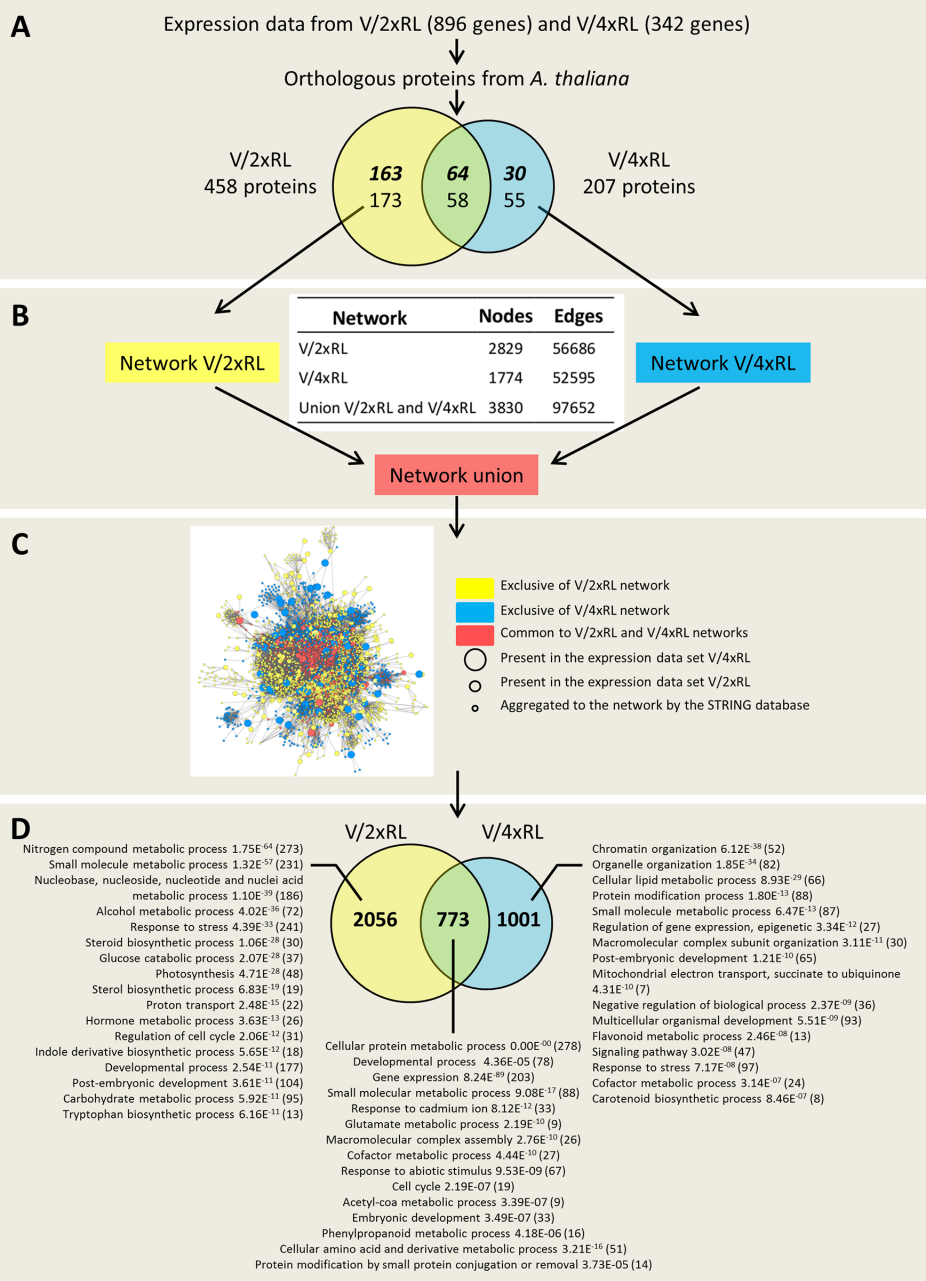
Scheme of the data mining from gene expression to the identification of the main biological processes from V/2xRL and V/4xRL. **A.** Venn diagram from *A*. *thaliana* proteins orthologous of the V/2xRL and V/4xRL sequences differentially expressed [20]. Bold-italic and normal style indicate proteins corresponding to up-regulated and down-regulated genes, respectively. **B.** PPI network characteristics. **C.** Main PPI network corresponding to the union of the V/2xRL and V/4xRL specific networks. **D.** Venn diagram of the V/2xRL and V/4xRL specific PPI networks. The main metabolic processes were indicated with the corresponding *e*-value and amount of associated proteins (under parenthesis).

For this study, we used Citrus gene expression data previously obtained [20]. Briefly, gene expression had been obtained by microarrays from diploid (2x) and autotetraploid (4x) clones of Rangpur lime (*Citrus limonia*, Osbeck) rootstocks grafted with 2x Valencia Delta sweet orange (*Citrus sinensis*) scions, named V/2xRL and V/4xRL, respectively, both combination submitted or not to drought. Trees were grown in 4 L pots containing fresh commercial soil, and regular fertilization as previously described [20, 22]. Transcriptomic data were obtained from 4 randomly selected independent biological replicates (leaf samples) per tree combination (V/2xRL *vs* V/4xRL) and condition (control *vs* submitted to drought) [20]. Two data sets were available: one corresponding to genes differentially expressed in V/2xRL genotype in response to water deficit (comparison control *vs* drought), the other corresponding to genes differentially expressed in V/4xRL genotype in response to water deficit (comparison control *vs* drought) [20] (S1 Table; Fig 1A).

### Protein-protein interaction network construction

For the construction of the PPI network from the transcriptomics data of citrus, orthologous protein sequences of *Arabidopsis thaliana* were used. The search, in Arabidopsis, of the protein orthologous sequences from citrus, was made using the GetAtOrt tool avaliable at the Citrus Functional Genomics Project databank (CFGP; http://bioinfo.ibmcp.upv.es/genomics/cfgpDB/). The two data sets of Arabidopsis orthologous proteins corresponding to V/2xRL and V/4xRL were compared using the VennPlex program, which is able to build a Venn diagram and at the same time to identify up- and down-regulated sequences [30]. Only proteins exclusive of each data set were selected as input for the system biology analysis (Fig 1A). The networks were built using the STRING 10 software (http://www.string-db.org) according to the following parameters: use of the co-expression, experiments and co-occurrence databases; no more than 50 interactions; and confidence value of 0.4. The proteins that did not present any connection with the general network were submitted again to the STRING software; such process was repeated until no more connections were found. The generated sub-graphs were associated using the Cytoscape 3.2.1 (http://www.cytoscape.org) [31] with the use of the merge networks tool, to generate the final networks (Fig 1B and 1C).

### Gene ontology analysis

Gene ontology clustering analysis was performed using the Biological Network Gene Ontology (BiNGO) software v.2.44, a Cytoscape plugin available at http://www.cytoscape.org [32]. The degree of functional enrichment for a given cluster and category was quantitatively assessed (p value) by hypergeometric distribution and a multiple test correction was applied using the false discovery rate (FDR) algorithm, fully implemented in the BiNGO software [32]. Overrepresented biological process categories were generated after FDR correction, with a significance level of 0.05.

### Network centrality analysis

The CentiScaPe® plugin [33] was applied to the PPI networks to conduct the Degree and Betweenness centrality analyses in order to detect hub, bottleneck and hub-bottleneck nodes. Hubs were defined as highly connected nodes, i.e. nodes for which the individual node degree value was higher than the limit defined by the plugin for this variable in the all network. Bottlenecks were defined as nodes with betweenness value higher than the limit defined by the plugin for this variable in the all network.

### Building of the V/2xRL and V/4xRL model in response to drought

To build the models, the following tools were used: i) the PPI networks and ii) the metabolic pathways as described by KEGG and Mercator software [34]. The Mercator software was used to map all the transcripts previously identified [20] for both plant combination, generating a text file in which each protein from the input was mapped in one or more BINs [34]. The files generated by the Mercator software were used in the MapMan program; the MapMan program is used to visualize high throughput data and meta-analysis and is adapted to annotate plant omics data [35, 36]. The MapMan program was also used to correlate each transcript to its expression level. Moreover, initial microarray data were used to identify in the models the proteins that participated to a given biological process even if they did not generated any orthologous in Arabidopsis or were absent from the networks.

## Results and discussion

### Network building and analysis

**Fig 2:**
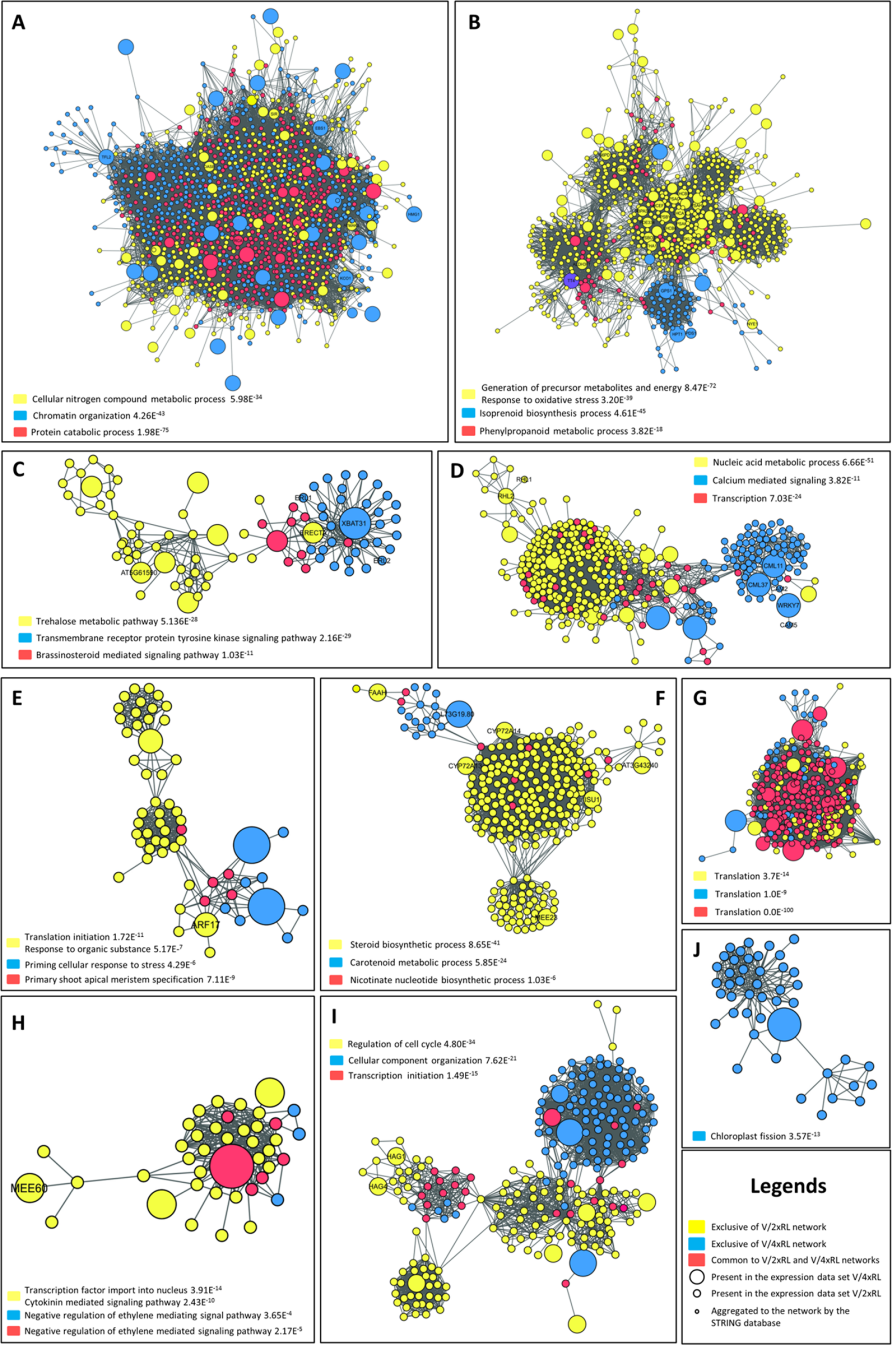
Functional clusters obtained from the general PPI network presented in the Fig 1C. Only the functions corresponding to the majority of the proteins involved were indicated. **A.** Cellular processes including protein catabolism and proteolysis ubiquitin-dependent. **B.** Metabolic processes including oxidative stress and photosynthesis. **C.** Phosphorylation and signaling pathway, and trehalose metabolic process. **D.** Nucleic metabolic process, transcription and gene expression, and RNA processing. **E.** Translation initiation and response to chemical, wounding and endogenous and exogenous stimulus. **F.** Lipid metabolic process, steroid, sterol and terpenoid biosynthesis processes. **G.** Protein metabolic process and translation. **H.** Response and signaling to cytokinin, regulation of ethylene mediated signaling pathway, negative regulation of two-component signal transduction system (phosphorelay). **I.** Cellular organization, regulation of cell cycle, membrane fusion and cellular component assembly, and transcription initiation. **J.** Organelle organization and glycolipid and galactolipid biosynthetic processes.

**Fig 3:**
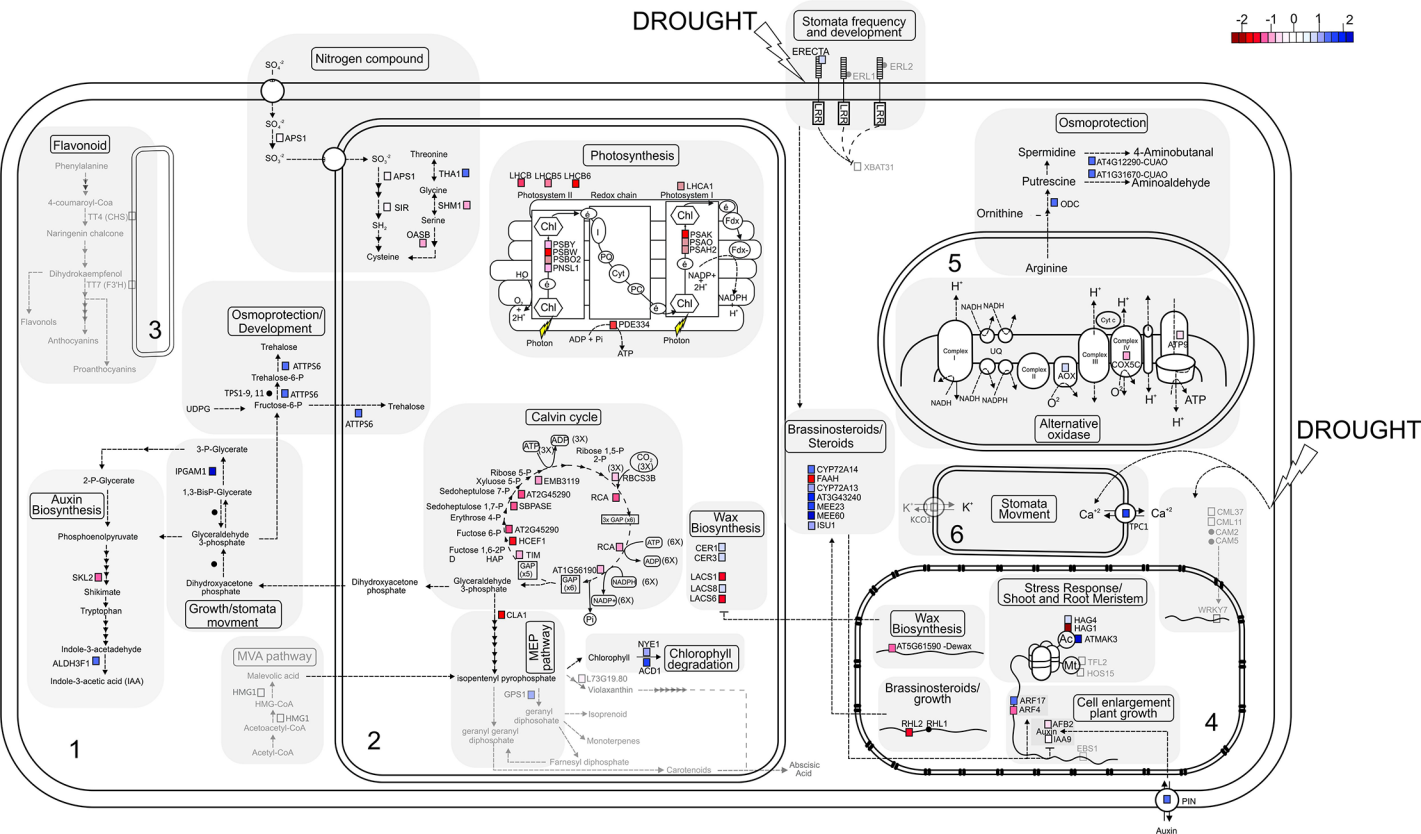
Model of metabolic pathways of V/2xRL subjected to drought according to transcriptomic and interactomic data. Numbers 1 to 6 correspond to cytoplasm, chloroplast, endoplasmic reticulum, nucleus, mitochondrion, vacuole, respectively. Color scale represents the gene fold change: repression is indicated in red scale while overexpression is indicated in blue scale.

Based on the Allario et al. work [20], 896 and 342 differentially expressed genes (between plant submitted to drought and control plants) from V/2xRL and V/4xRL, respectively, were used for PPI networks building. From them, 458 and 207 proteins of V/2xRL and V/4xRL, respectively, were orthologous to *A*. *thaliana* proteins. The Venn diagram showed that 122 proteins were common between V/2xRL and V/4xRL protein sets; from them 64 and 58 were up-regulated and down-regulated, respectively. From the 336 V/2xRL exclusive proteins, 163 and 173 were up-regulated and down-regulated, respectively. In the case of the 85 V/4xRL exclusive proteins, 30 and 55 were up-regulated and down-regulated, respectively (Fig 1A). From the specific V/2xRL and V/4xRL proteins, two *A*. *thaliana* PPI networks were build (Fig 1B). The V/2xRL network contained 2,829 nodes and 56,686 edges while the V/4xRL network contained 1,774 nodes and 52,595 edges. The union of both networks generated a unique main network containing 3,830 nodes and 97,652 edges (Fig 1B). The overlap of the two specific V/2xRL and V/4xRL networks allowed the identification of proteins exclusive of the V/2xRL network (2,056), exclusive of the V/4xRL network (1,001), and common to both networks (773) (Fig 1C and 1D). Exclusive proteins from V/2xRL corresponded to biological processes such ‘Photosynthesis’, ‘Sterol biosynthetic process’, ‘Hormone metabolic process’, ‘Indole derivative biosynthetic process’, ‘Flavonoid metabolic process’ and ‘Carotenoid biosynthetic process’, among others (Fig 1D). The main network contained several functional clusters identified by the BiNGO software (see Material and methods) and represented on the Fig 2. For each cluster, exclusive proteins from each network (V/2xRL *vs* V/4xRL) as well as common proteins of both V/2xRL and V/4xRL were indicated. Interestingly, some functional clusters presented very well compartmentalized protein groups according to the specific network (V/2xRL *vs* V/4xRL) (Fig 2C, 2D, 2E, 2F, 2H, 2I and 2J). The ‘Phosphorylation and signaling pathway, and trehalose metabolic process’ cluster showed that the proteins related to trehalose metabolic pathway were specific from the V/2xRL network, while the V/4xRL network mainly contained proteins associated to transmembrane receptor protein tyrosine kinase signaling pathway (Fig 2C). Common proteins between V/2xRL and V/4xRL were related to brassinosteroid mediated signaling pathway and connected the two specific networks (Fig 2C). In the cluster corresponding to ‘Nucleic metabolic process, transcription and gene expression, and RNA processing’ (Fig 2D), proteins related to nucleic acid metabolic process were found specifically in the V/2xRL network while proteins related to calcium mediated signaling were found specifically in the V/4xRL network. Proteins related to transcription were present in both networks and located mainly at the intersection of the two other specific groups of proteins (Fig 2D). The cluster corresponding to ‘Translation initiation and response to chemical, wounding and endogenous and exogenous stimulus’ showed specific V/2xRL proteins related to response to organic substance and specific V/4xRL proteins related to priming cellular response to stress (Fig 2E). Common proteins were related to primary shoot apical meristem specification and were connected to the two other groups of proteins (Fig 2E). The cluster corresponding to ‘Lipid metabolic process, steroid, sterol and terpenoid biosynthesis processes’ contained proteins from the V/2xRL network related to steroid biosynthetic process, proteins from the V/4xRL network related to carotenoid metabolic process, and few and dispersed common proteins associated to nicotinate nucleotide biosynthetic process (Fig 2F). Systems biology studies, through network analysis, lead to the challenge of the topology and function network understanding. For this reason, the cluster analysis made here was highly relevant because it allowed to select, inside the network, protein groups with high connectivity that are generally related with well-defined biological processes; such groups of proteins constitute functional modules or protein complexes. Functional modules are groups of proteins whose interactions occur in distinct place or time, as signalization or metabolic pathways, among others. On the other hand, the protein complexes participate to molecular machineries occurring in the same local and time [37–39]. Here, the clusters are mainly a mixture of functional modules and protein complexes. This phenomena is typical of clusters from biological networks, which are composed by sub-graphs, which, in turn, are responsible for the biological processes inside the network [40]. Sub-graphs, such as those observed in the Fig 2G, showed the quality of the clustering of the present work, allowing the separation of protein complexes validated by the low p-value gene ontology (e.g. “Translation”). The cluster showed in the Fig 2B also contained multiple biological processes represented by sub-graphs such as the protein set related to photosynthesis and Calvin cycle in the V/2xRL plant (see also Fig 3). Generally, interatomic of complex organisms does not allow such a so clear separation of functional modules as it could be observed for unicellular organisms or small biological systems [40]. Here, we observed a very good separation between V/2xRL and V/4xRL proteins in several clusters, indicating that distinct metabolic pathways were involved in the response of each genotype to drought.

### Cellular and physiological models for V/2xRL and V/4xRL

#### Photosynthesis and carbon fixation

**Fig 4:**
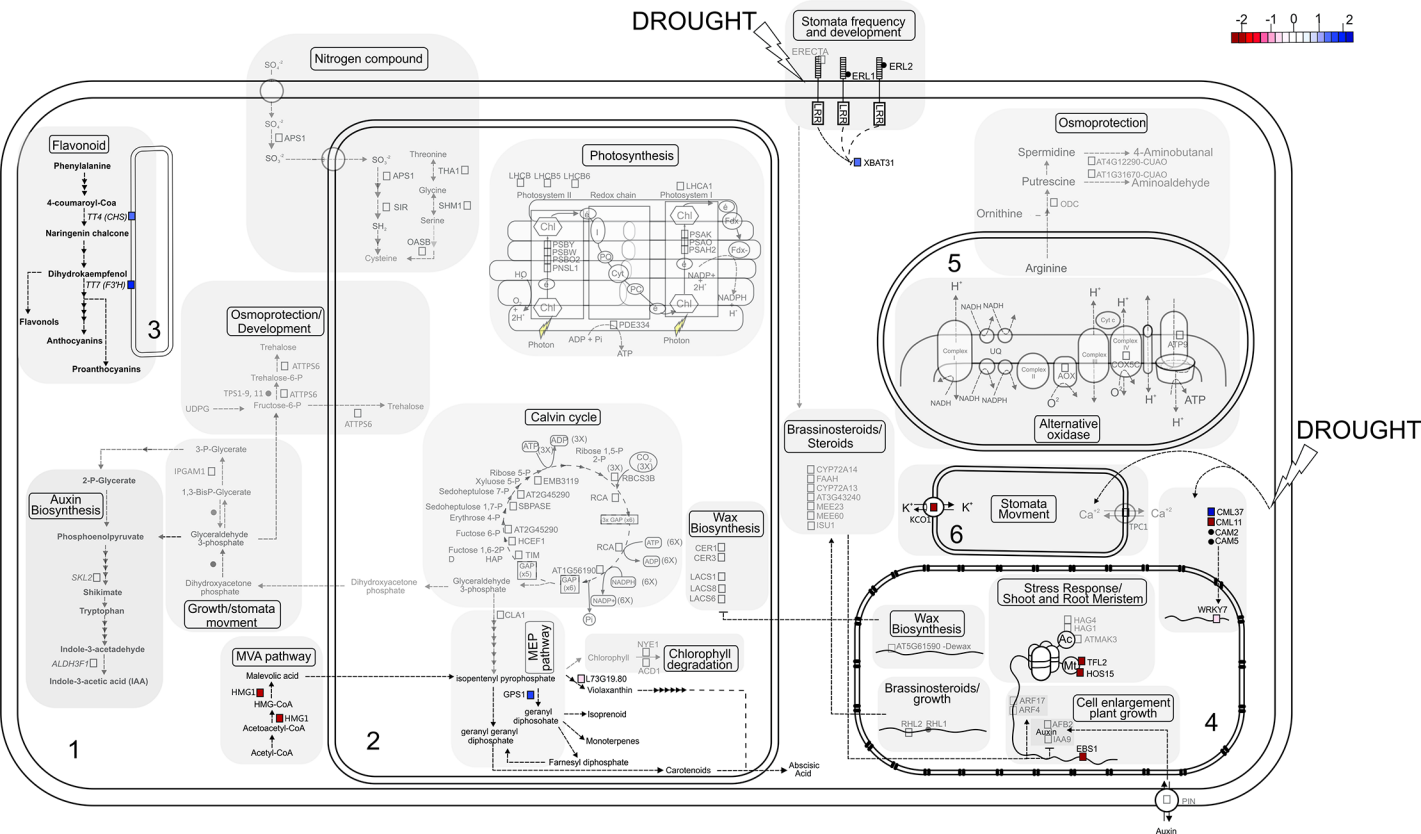
Model of metabolic pathways of V/4xRL subjected to drought according to transcriptomic and interactomic analysis. Numbers 1 to 6 correspond to cytoplasm, chloroplast, endoplasmic reticulum, nucleus, mitochondrion, vacuole, respectively. Color scale represents the gene fold change: repression is indicated in red scale while overexpression is indicated in blue scale.

**Fig 5:**
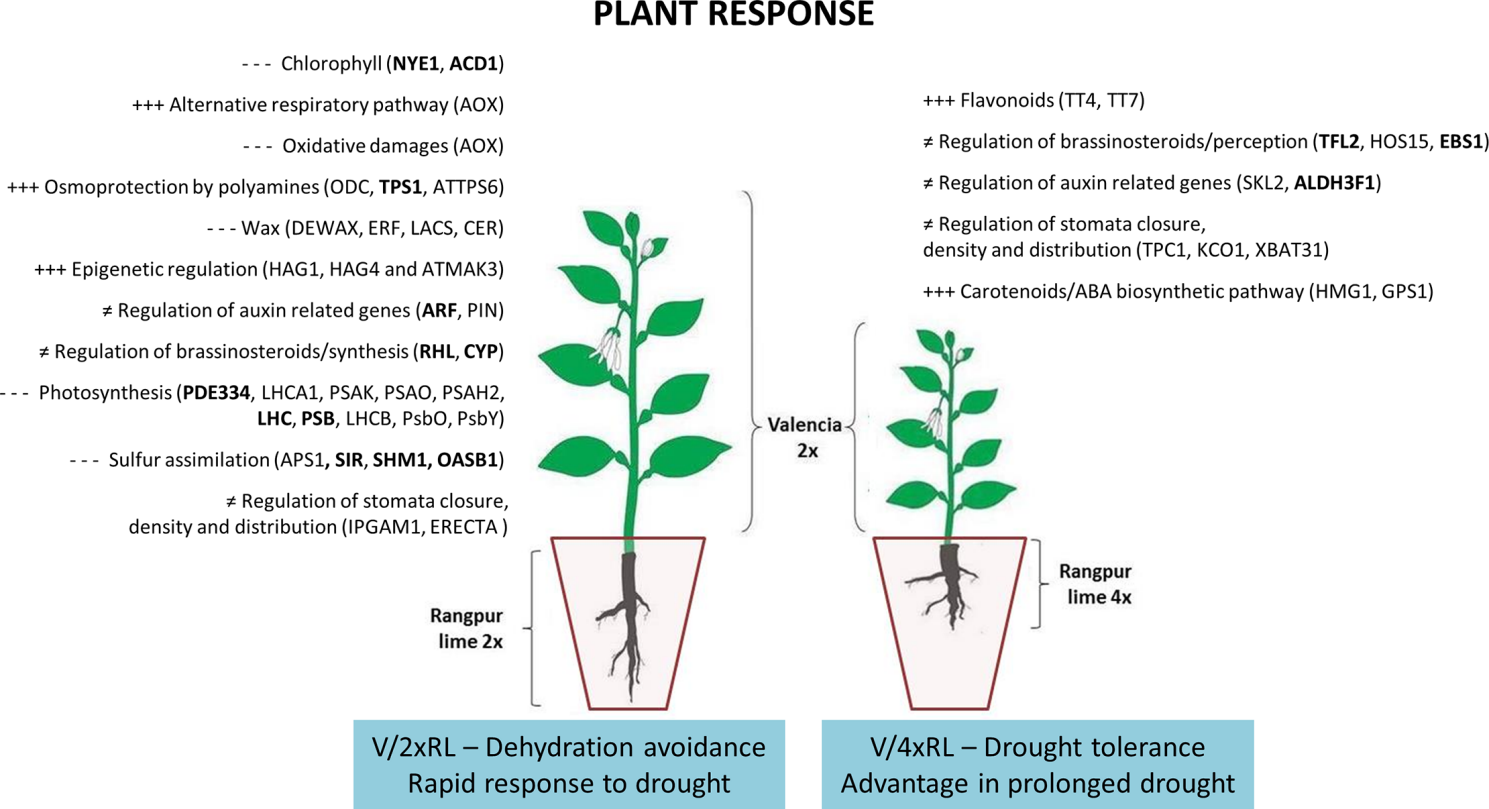
Scheme of the scion response to drought in each scion/rootstock combination (V/2xRL vs V/4xRL). +++: presence/high level;- - -: absence/low level; ≠: differential level.

In V/2xRL, all the genes related to photosynthesis were repressed in drought stress conditions, while no differential expression was observed in the V/4xRL plants (Figs 3 and 4). Basically the repressed genes were related to the photosystems I and II (PSI and PSII, respectively), except the ATP synthase subunit b chloroplastic-like (PDE334), which is directly associated with the electron transport chain. The PDE334 gene was repressed in the V/2xRL plants (-1.282 fold change; Fig 3, Table 1). Substantial changes in ATP synthase contents in response to drought [41, 42] have been previously reported as well as changes in plant assimilation capacities. Physiological analysis of lines with reduced ATP synthase expression revealed a strongly increased proton motive force (pmf) across the thylakoid membrane, leading to the activation of photoprotective mechanisms and downregulation of linear electron flux in low light. This situation resulted in repression of leaf assimilation and plant growth, supporting a central role of the ATP synthase in regulating photosynthesis. Loss of ATP synthase activity resulted in drastic acidification of the thylakoid lumen and subsequent breakdown of photosynthetic electron transfer and assimilation [43]. The PDE334 protein was identified as a hub-bottleneck in the PPI network centrality analysis (Table 1; S2 Table) and is present in the specific V/2xRL network related to ‘Generation of precursor metabolites and energy’ cluster (Fig 2B, S3 Table). The PSI is a multi-subunit protein complex located in the thylakoid membranes of green plants and algae where one of the first steps of solar energy conversion by light-driven electron transport is initiated [44]. In plants, the PSI complex consists of at least 19 protein subunits [45]. In our work, four subunits were present in the PPI network (LHCA1, PSAK, PSAO, PSAH2, fold change -0.903, -1.969, -1.343 and -1.023, respectively; Table 1, Fig 3). Elimination of PsaK in plants using either antisense or gene knock-out technology has demonstrated that PsaK is involved in binding of Lhca2 and Lhca3 [46, 47]. The PsaO subunit was discovered in *A*. *thaliana* during characterization of a mutant deficient in PsaN [48]. PsaO seems to be present in higher plants, mosses and green algae but has no counterpart in cyanobacteria. Arabidopsis plants devoid of the PsaO core subunit showed 50% reduction in state transitions [49], indicating the role of this protein in putative binding of mobile LHCII. An even more drastic effect on state transitions was demonstrated by Lunde et al. who suppressed the expression of the PsaH and PsaL core subunits in Arabidopsis [50]. Plants lacking PsaH were essentially unable to perform state transitions and were locked in State 1, indicating direct involvement of PsaH as a docking site for the mobile phospho-LHCII under state 2 conditions. Importantly, in the absence of PsaH, nonphotochemical fluorescence quenching was identical upon illumination with light 1 and light 2, and LHCII still underwent phosphorylation in state 2. These results suggest that the majority of LHCII in the PsaH null plants remains attached to PSII in spite of the unaffected LHCII phosphorylation [51]. In fact, PsaH was involved in balancing of the excitation energy between PSI and PSII via state 1 –state 2 transition [50]. In this process a mobile pool of LHCII moves from PSII to PSI under light conditions that favors PSII and vice versa [52]. The PSII is a multi-subunit pigment-protein complex found in thylakoid membranes of oxygenic photosynthetic organisms, including cyanobacteria, algae, and plants [53, 54]. Driven by light, PSII catalyzes electron transfer from water to plastoquinone. Therefore, PSII is also known as a water-plastoquinone oxidoreductase. In our work, two types of core proteins were present in the network and also differentially expressed at transcriptional level: light-harvesting complex (LHC) proteins (LHCB, LHCB5 and LHBC6, gene expression fold change -1.127, -1.57 and -1.57, respectively; Table 1, Fig 3); and PSB group (PSBO2, PSBW, PSBY and PNSL1, gene expression fold change -1.122, -1.469, -0.985 and -0.853, respectively; Table 1, Fig 3). In V/2xRL plants, several of these subunits were identified as hubs in the PPI network centrality analysis (Table 1; S2 Table) and only PSBW and PSNL1 have no centrality. Most of these subunits were present in the specific V/2xRL PPI network related to ‘Generation of precursor metabolites and energy’ cluster (Fig 2B, S3 Table). The PSII-light-harvesting antenna (i.e., light-harvesting complex II, abbreviated as LHCII) in land plants is an integral membrane complex. LHCII contains three major trimeric PSII light-harvesting chlorophyll a/b-binding (LHCB) proteins LHCB1, LHCB2, and LHCB3 and three minor monomeric LHCB proteins LHCB4, LHCB5, and LHCB6 [55, 56]. According to Girolomoni et al. [57], LHCBM4 and LHCBM6, rather than having an essential function in photon capture, are likely to be involved in photoprotective mechanisms with a specific function within a pool of LHCII proteins free or very loosely connected to the PSII supercomplex. PsbO appears to regulate functioning of PSII. Indeed, removal of PsbO from PSII leads to partial loss of the manganese ions from the catalytic center, decreased oxygen production, and perturbed dynamics of water at the active site and of the reaction cycle [58]. PsbO2 is the minor isoform in the wild-type. Mutants defective in this gene have been shown to be affected in the dephosphorylation of the D1 protein of PSII. To reveal the function of PsbY within PSII of Arabidopsis, [59] analyses PsbY knock-out plants and compared them to wild type and to complemented mutant lines. The authors showed that in the absence of PsbY protein, low potential form and plants depleted of PsbY were found to be more susceptible to photoinhibition. However, analyses of cyanobacterial mutants with inactivated psbY gene demonstrated that loss of PsbY has no dramatic effect on PSII activity [60]. As general conclusion, the V/2xRL plants presented a reduction of the photosynthesis that may be related to the rapid response to drought of these plants (Figs 3 and 5) as well as to water depletion observed during this response [22].

**Table 1:** List of the sequences involved in the main metabolic processes described in the Figs 3 and 4. ID: Accession number.

*Arabidopsis thaliana* ID	*Citrus clementina* ID	Putative function	Gene expression on microrray^a^		PPI network		
			Type	Fold change	Type^b^	Cluster	Centrality^c^
ACD1	KN0AAP3YA21	Pheophorbide a oxygenase family with rieske 2Fe-2s domain-containing	2x	1.894	2x	−	B
AFB2	C31705D07	Auxin signaling F-box 2	2x	-0.779	−	−	−
ALDH3F1	C02019C10	Aldehyde dehydrogenase family 3 member f1-like	2x	1.183	2x	Fig 2B	HB
AOX	KN0AAP7YK07	Alternative oxidase	2x	0.879	−	Fig 2B	−
APS1	C34205B10	Sulfate adenylyltransferase 1	2x	-0.903	−	−	−
ARF17	KN0AAP8YN01	Auxin response factor 17	2x	1.222	2x	Fig 2E	B
ARF4	IC0AAA48CE01	Auxin response factor 4	2x	-1.162	2x	−	B
AT1G31670	C07010E12	Copper amine oxidase family	2x	1.252	2x	−	C
AT1G56190	C31501D05	Phosphoglycerate kinase	2x	-0.903	−	−	−
AT2G45290	C02022D07	Transketolase	2x	-1.192	2x	Fig 2B	B
AT3G43240	IC0AAA47CF07	Arid/brightdna	2x	2.072	2x	Fig 2F	C
AT4G12290	C01017A05	Copper amine oxidase family	2x	1.057	2x	−	C
AT5G61590	C01002B06	Ethylene-responsive transcription factor ERF107	2x	-2.622	2x	Fig 2C	C
ATMAK3	KN0AAP4YC19	N-alpha-acetyltransferase MAK3	2x	3.784	2x	Fig 2A	C
ATP9	C05054A09	ATP9 (mitochondrion)	2x	-0.867	−	−	−
ATTPS6	C31403D05	alpha,alpha-trehalose-phosphate synthase	2x	1.566	2x	Fig 2C	C
CAM2	−	Calmodulin 2	−	−	4x	Fig 2D	B
CAM5	−	Calmodulin 5	−	−	4x	Fig 2D	C
CER1	KN0AAQ13YH02	Fatty acid hydroxylase superfamily	2x	0.914	−	−	−
CER3	C02002B06	Eceriferum 1-like	2x	0.898	−	−	−
CLA1	C31504D11	DEF (cla1)	2x	-1.065	−	−	−
CML11	C02007D09	Calmodulin	4x	2.722	4x	Fig 2D	C
CML37	KN0AAP7YM03	Calmodulin	4x	-2.371	4x	Fig 2D	C
COX5C	C06018C04	Cytochrome oxidase subunit 5	2x	-1.054	−	−	C
CYP72A13	C02015E10	Cytochrome p450	2x	1.164	2x	Fig 2F	H
CYP72A14	C05065E02	Cytochrome p450	2x	1.360	2x	Fig 2F	H
EBS1	C08011G05	EMS-mutagenized BRI1 suppressor 1	4x	-2.201	4x	Fig 2A	B
EMB3119	C31601F06	Ribose 5-phosphate isomerase	2x	-0.902	−		−
ERECTA	C02008C12	Erecta receptor kinase	2x	0.905	2x	Fig 2C	C
ERL1	−	Erecta-like 1 receptor kinase	−	−	4x	Fig 2C	C
ERL2	−	Erecta-like 2 receptor kinase	−	−	4x	Fig 2C	C
FAAH	C06020H11	Fatty acid amide hydrolase	2x	-1.652	2x	Fig 2F	C
GPS1	IC0AAA90AC08	Geranyl diphosphate synthase 1	4x	1.642	4x	Fig 2B	H
HAG1	IC0AAA26CD08	Histone acetyltransferase of the gnat family 1	2x	-2.922	2x	Fig 2I	C
HAG4	C02008H08	Histone acetyltransferase of the myst family 1	2x	0.867	2x	Fig 2I	C
HCEF1	C31007A10	High cyclic electron flow 1	2x	-1.421	2x	Fig 2B	HB
HMG1	IC0AAA41DG07	3-hydroxy-3-methylglutaryl reductase	4x	-2.616	4x	Fig 2A	C
HOS15	IC0AAA32AC03	WD-40 repeat family	4x	-3.032	4x	−	C
HPT1	C05068G03	Homogentisate phytyltransferase 1	2x_4x	-2.613	4x	Fig 2B	C
IAA9	C32104D04	Indole-3-acetic acid inducible partial c	2x	-0.782	2x	−	C
IPGAM1	IC0AAA90AA03	Phosphoglycerate mutase	2x	2.727	2x	Fig 2B	C
ISU1	C34207H04	Iron-sulfur cluster	2x	1.0687	2x	Fig 2F	C
KCO1	C31202C03	Two-pore potassium channel 1-like	4x	-5.194	4x	Fig 2A	C
L73G19.80	C34006A02	Beta-carotene hydroxylase	4x	0.925	4x	Fig 2F	C
LACS1	C20004C04	AMP-dependent synthetase and ligase family	2x	-1.262	2x	−	H
LACS6	C05056H01	Long-chain acyl-synthetase	2x	-1.574	−	−	−
LACS8	C31504H07	AMP-dependent synthetase and ligase family	2x	0.937	−	−	−
LHCA1	C16005G04	Chlorophyll a-b binding chloroplastic isoform x1	2x	-0.903	2x	Fig 2B	H
LHCB	C32005E07	Light-harvesting chlorophyll b-binding	2x	-1.127	2x	Fig 2B	HB
LHCB5	C32008B06	Chlorophyll a b-binding-like	2x	-1.57	2x	Fig 2B	H
LHCB6	C32008B06	Light harvesting complex photosystem II subunit 6	2x	-1.57	2x	Fig 2B	H
MEE23	C06053H11	Ternal effect embryo arrest 23	2x	1.7311	2x	Fig 2F	C
MEE60	IC0AAA18CA12	Ternal effect embryo arrest 60	2x	2.9931	2x	Fig 2F	C
NYE1	C02026E07	Non-yellowing 1	2x	1.3	2x	Fig 2B	B
OASB	C31007H03	O-acetylserine (thiol) lyase b	2x	-1.317	2x	Fig 2B	B
ODC	IC0AAA46AH09	Ornithine decarboxylase	2x	1.544	−	−	−
PDE334	C02017B02	ATP synthase subunit b chloroplastic-like	2x	-1.282	2x	Fig 2B	HB
PDS1	C34206B03	4-hydroxyphenylpyruvate dioxygenase	4x	1.873	2x	Fig 2B	C
PIN	C04034B12	Kinase pinoid	2x	1.422	−	−	−
PNSL1	C31002F01	Photosystem II reaction center family	2x	-0.853	−	−	−
PSAH2	C01017F05	Photosystem I reaction center subunit chloroplastic-like	2x	-1.023	2x	Fig 2B	H
PSAK	C05072A10	Photosystem I reaction center subunit chloroplastic	2x	-1.343	2x	Fig 2B	H
PSAO	C07012D04	Photosystem I subunit O	2x	-1.966	2x	Fig 2B	HB
PSBO2	C31403H07	Photosystem II subunit O-2	2x	-1.122	2x	Fig 2B	H
PSBW	C31604G05	Photosystem II reaction center W		-1.469	2x	−	−
PSBY	C31007B05	At1g67740 f12a21_13	2x	-0.985	2x	Fig 2B	H
RBCS3B	C31604D03	Ribulose bisphosphate carboxylase (small chain) family	2x	-0.713	−	−	−
RCA	C05804A10	Rubisco activase	2x_4x	-1.168	2x	Fig 2A	HB
RHL1	−	Root hairless 1	−	−	2x	Fig 2D	C
RHL2	IC0AAA41AG09	Root hairless 2	2x	-1.4625	2x	Fig 2D	B
SBPASE	C31001E04	Sedoheptulose-bisphosphatase precursor	2x	-1.218	2x	Fig 2B	HB
SHM1	C05073H08	Glycine hydroxymethyl transferase	2x	-1.012	2x	Fig 2B	HB
SIR	C31007H03	Sulfite reductase	2x	-1.317	2x	Fig 2A	B
SKL2	C08031D04	Probable inactive shikimate kinase like chloroplastic	2x	-1.389	2x	Fig 2B	C
TFL2	IC0AAA12CC07	Like heterochromatin (lhp1)	4x	-2.535	4x	Fig 2A	HB
THA1	C08036F01	Threonine aldolase 1	2x	1.425	2x	Fig 2B	C
TIM	C32001D11	Triosephosphate isomerase	2x_4x	-0.82	2x	Fig 2A	HB
TPC1	C05075C09	Two-pore channel 1		1.778	2x	Fig 2B	−
TPS1	−	Trehalose-phosphatase/synthase 1	−	−	2x	Fig 2C	C
TPS11	−	Trehalose-phosphatase/synthase 11	−	−	2x	Fig 2C	C
TPS2	−	Trehalose-phosphatase/synthase 2	−	−	2x	Fig 2C	B
TPS3	−	Trehalose-phosphatase/synthase 3	−	−	2x	Fig 2C	B
TPS4	−	Trehalose-phosphatase/synthase 4	−	−	2x	Fig 2C	C
TPS5	−	Trehalose-phosphatase/synthase 5	−	−	2x	Fig 2C	C
TPS7	−	Trehalose-phosphatase/synthase 7	−	−	2x	Fig 2C	B
TPS8	−	Trehalose-phosphatase/synthase 8	−	−	2x	Fig 2C	B
TPS9	−	Trehalose-phosphatase/synthase 9	−	−	2x	Fig 2C	B
TT4	C32013G05	Chalcone synthase family	2x_4x	1.089	4x	Fig 2B	B
TT7	C21007C11	Flavonoid 3-monooxygenase	4x	2.321	4x	−	B
WRKY7	C31802C04	WRKY transcription factor	4x	-3.9967	4x	Fig 2D	C
XBAT31	C34208B09	Putative E3 ubiquitin-protein ligase	4x	1.1898	4x	Fig 2C	C

^a^ According to Allario et al. (2013).

^b^ 2x: V/2xRL; 4x: V/4xRL.

^c^ B: bottleneck; C: common; HB: hub-bottleneck.

#### Shoot and root meristem development

The V/2xRL and V/4xRL plants submitted to drought conditions presented different expression pattern of genes in relation to cell enlargement and plant growth. In V/2xRL three acetyltransferases (HAG1, HAG4 and ATMAK3) were differentially expressed while no differential expression of these genes was observed in the V/4xRL plants (Figs 3 and 4). The HAG1 gene was highly repressed and the ATMAK3 and HAG4 genes were overexpressed (-2.922, 3.784 and 0.867 fold change, respectively; Table 1, Fig 3). These acetyltransferases are known to be involved in the chromatin remodeling and unpacking, leading to transcription regulation in different physiological situations as in response to drought [61]. Moreover, the GNAT and MYST families of acetyltransferases–from which the HAG1 and HAG4 belong–are known to be involved in the epigenetic regulation of gene expression [62]. The corresponding proteins were found in the specific V/2xRL sub-network (Fig 2I; S3 Table). The differential expression of these acetyltransferases would lead to the expression of ARF17 and repression of ARF4 (1.222 and -1.162 fold change, respectively; Table 1, Fig 3), two auxin-responsive factors [63]. The ARF genes have been related to meristem function and organogenesis control in both shoots and roots through the direct regulation of kinase pinoid (PIN) gene, which, in the V/4xRL plants was overexpressed (1.422 fold change; Table 1, Fig 3) [64]. The PIN protein is an auxin transporter related to root gravitropism and hydrotropism [65, 66]. The ARF4 and ARF17 proteins were present in the specific V/2xRL sub-network and were identified as bottlenecks in the network centrality analysis (Fig 2E, Table 1, S2 Table). In fact, these proteins seemed to be essential for recycling of PIN auxin transporters and for various auxin-dependent developmental processes [65]. In V/2xRL plants, the auxin signalization seemed closely coordinated with the brassinosteroid biosynthetic pathway and associated to cell enlargement and root development (root hairless/RHL genes; Fig 3); the RHL2 protein was identified as a bottleneck in the network centrality analysis (Table 1, S2 Table) and is present in the specific V/2xRL subnetwork (Fig 2D). It has been evidenced that a crosstalk between auxin and brassinosteroid pathways occurs in plant during development mainly through enhancement by brassinoteroids of ARF and PIN genes and repression of AUX/AAI complex expression [67]. In V/2xRL plants, genes related to brassinosteroid pathway were overexpressed (Fig 3) and some of the corresponding proteins, CYP72A13 and CYP72A14 corresponding to cytochrome P450, were identified as hubs in the network centrality analysis (Table 1; Fig 2F; S3 Table). In the V/4xRL plants, the TFL2, HOS15 and EBS1 genes were highly repressed and completely different signalization pathways were related to development (Fig 4). In other works, TFL2 gene product appears to have a dual role in regulating meristem activity, one being to regulate the meristem response to light signals affecting the development of the plant and the other being the maintenance of inflorescence meristem identity [68]. The EBS1 gene has been identified as a suppressor of the growth defects of a brassinosteroid receptor mutant, bri1-9, in an allele-specific manner by restoring its brassinosteroid sensitivity [69]. Because it has been shown that EBS1 directly affects brassinosteroid perception at the cell surface but without causing constitutive activation of brassinosteroid signaling [69], the repression of EBS1 in V/4xRL may be related to reduction of brassinosteroid sensitivity in these plants and consequently to the plant size reduction that is one of the element of the typical phenotype of plants defective in brassinosteroid biosynthesis [70]. Moreover TFL2 and EBS1 proteins were identified as hub-bottleneck and bottleneck, respectively, in the network centrality analysis (Table 1, Fig 2F, S3 Table) and are present in the specific V/4xRL subnetwork (Fig 2A). In the V/4xRL plants, two other genes involved in the IAA biosynthetic pathway were differentially expressed, a shikimate kinase (SKL2) and an aldehyde dehydrogenase (ALDH3F1) repressed and overexpressed, respectively (-1.389 and 1.183; Table 1, Fig 2B, S3 Table). ALDH3F1 protein was identified as a hub-bottleneck in the network centrality analysis (Fig 2, Table 1, S2 Table). Thus the balance and crosstalk between brassinosteroids and auxin represent an important element of the regulation of the shoot and root development of the V/2xRL and V/4xRL plants (Fig 5).

#### Wax biosynthesis

In V/2xRL, the AT5G61590-Dewax and the long-chain acyl-CoA synthetase LACS1 and LACS6 genes were repressed (-1.262 and -1.574 fold change, respectively; Table 1, Fig 3) and the CER1, CER3 and LACS8 genes were lowly expressed under drought (0.914, 0.898 and 0.937 fold change, respectively; Table 1, Fig 3) while no differential expression was observed for these genes in the V/4xRL stressed plants (Fig 4). Globally, in V/2xRL, the wax biosynthetic pathway was repressed through the action of the DEWAX gene encoding an ERF-type transcription factor known to negatively regulate wax biosynthesis genes such LACS and CER genes [71, 72]. This results may be related to the molecular and physiological differences in root and stem cuticle thickness observed between 2x and 4x stressed plants [20, 22]. In both Allario and coll. works, the 4x plants (grafted or not with Valencia scion) presented thicker cuticle on roots and stem, but no difference was observed in leaves [20, 22]. In a general way, the V/4xRL presented higher wax biosynthesis and a physical protection against dehydration higher than the V/2xRL (Fig 5).

#### Osmoprotection

In the V/2xRL, the ornithine decarboxylase (ODC) and two members of the copper amine oxidase family (AT4G12290 and AT1G31670) were overexpressed under drought (1.544, 1.057, 1.252 fold change; Table 1, Fig 3) while no differential expression was observed for these genes in the V/4xRL stressed plants (Fig 4). These genes are responsible for the transformation of arginine to putrescine and spermidine (polyamine group) and subsequently to 4-aminobutanal and aminoaldehyde, all molecules related to osmoprotection and/or water balance homeostasis in stressed plants. Polyamines are ubiquitous organic-amines whose accumulation in plants is related to protection against drought or salt stress [73]. Here the osmoprotection was activated in the V/2xRL plants subjected to drought in comparison with the V/4xRL ones (Figs 3 and 4) even if the V/4xRL presented higher global tolerance to drought [20]. Moreover, in V/2xRL plants, trehalose-6-phosphate synthase (TPS1) and alpha, alpha trehalose phosphate synthase (ATTPS6) were overexpressed (1.566 fold change; Table 1, Fig 3). Both genes are involved in the biosynthesis pathway of trehalose, a non-reducing disaccharide known to be a stress protectant molecule found in several organisms including plants. Generally the constitutive level of trehalose in plant cells is not very high; therefore, it is believed to act as a signaling molecule under stress conditions [74]. In V/2xRL plants, several TPS proteins were identified as bottleneck in the network centrality analysis (Table 1, S2 Table). The presence of several genes related to osmoprotection in the V/2xRL plants–which did not present the global higher tolerance to drought compared to the V/4xRL plants [20, 22]–suggested that V/2xRL and V/4xRL plants used different mechanisms to lead to drought stress, that the osmoprotection through polyamines and trehalose is not necessarily the most efficient, and that the V/4xRL plants would develop alternative and more efficient mechanism to lead to this abiotic stress (Fig 5).

#### Sulfur assimilation and amino acid synthesis

In V/2xRL plants submitted to drought, several genes related to sulfur assimilation and amino acid synthesis were differentially expressed while no differential expression was observed in V/4xRL (Figs 3 and 4). The genes APS1, SIR, SHM1 and OASB1 were repressed (-0.903, -1.317, -1.012 and -1.317 fold changes, respectively; Table 1, Fig 3) while the gene THA1 was overexpressed (1.425 fold change; Table 1, Fig 3). The sulfur assimilation seems to play an important role in drought and oxidative stress [75–78]. The interaction between hydrogen sulfide (H_2_S) and nitric oxide (NO) has been shown and was related to stomatal aperture/closure via ABA-dependent pathway [75]. Moreover, the synthesis of several osmoprotectants is coordinated with the sulfur assimilation [76, 78]. The sulfur assimilation is also related to the regulation of cysteine synthesis during drought [75, 76]. Among other mechanisms, the OAS may participate in the regulation of partitioning between primary and secondary sulfur metabolism during drought stress [76]. Interestingly, the SIR, SHM1 and OASB1 proteins were identified as bottlenecks or hub-bottlenecks in the network centrality analysis (Table 1, S2 Table). The V/2xRL plants present differential expression (repression) of sulfur assimilation genes that may be related to less protein synthesis as well as less oxidative damages (Fig 3 and Fig 5).

#### Respiration

In the V/2xRL plants submitted to drought, some genes of the respiratory pathway are differentially expressed (Fig 3) while no differential expression was observed in the V/4xRL plants grown in the same conditions (Fig 4). The alternative oxidase (AOX) was slightly expressed while the cytochrome oxidase subunit 5 (COX5C) and the ATP9 were slightly repressed (0.879, -1.054 and -0.867 fold change; Table 1, Fig 3). The corresponding proteins belong to the mitochondrial electron transport chain and were associated to the specific ‘generation of precursor metabolites and energy/response to oxidative stress’ sub-network (Fig 2B). AOX is known to be involved in mediating signalling and metabolic activities during stress response–including drought–in plants [79]. AOX indirectly controls the synthesis of molecules like hydrogen peroxide, superoxide, nitric oxide and is able to reduce oxidative damage [79, 80]. Moreover, it has been shown that AOX is essential to maintaining respiration in the light, and that this non-energy conserving respiration maintains photosynthesis during drought by promoting energy balance in the chloroplast [81]. The differential AOX gene expression in the V/2xRL suggested that this plant preferentially used the alternative respiratory pathways limiting oxidative damages in the respiratory and photosynthetic apparatus in response to drought (Fig 5).

#### Stomata movement, conductance and density

The V/2xRL and V/4xRL plants submitted to drought presented different gene expression in relation to stomata movement, conductance and density. Two different two-pore channels related genes were differentially expressed: the two-pore channel 1 (TPC1) in V/2xRL plants and the two pore K^+^ channel (KCO1) in V/4xRL plants (1.778 and -5.195 fold changes, respectively; Table 1, Figs 3 and 4). It has been shown that, in stomata, the two pore K^+^ channel is involved in vacuolar K^+^ release and that the removal of this channel (in transgenic plants) led to lower stomatal closure kinetics [82]. The TPC1 gene is related to the regulation of the conductance of sodium and calcium ions; the TCP1 role in stomata is controversial, some works indicated that it could be related also to stomatal closure [83, 84] or that TCP1 does not have any role in guard cell movement [85]. In the V/2xRL plants, phosphoglycerate mutase (IPGAM1) gene is overexpressed (2.727 fold changes; Table 1, Fig 3). IPGAM1 gene is involved in the glycolysis and it has been shown that both are critical for guard cell function and stomatal opening/closure but is not involved in guard cell size or stomatal density [86]. In both V/2xRL and V/4xRL plants, ubiquitin E3 ligase (ERECTA) genes were found, and in V/2xRL one gene is slightly overexpressed (0.905 fold change, respectively; Table 1, Fig 3 and Fig 4). The ERECTA genes are known to be involved in the control of stomatal density and distribution [87]. The ERECTA protein was present in the specific V/2xRL subnetwork (Fig 2C). In the V/4xRL plants, another ubiquitin E3 ligase (XBAT31) was overexpressed (1.1898 fold change; Table 1, Fig 4); this protein has been related also to stomata closure. XBAT31 protein was present in the specific V/4xRL subnetwork (Fig 2C). In both V/2xRL and V/4xRL plants, different genes were involved in opening/closure of stomata and/or stomata density and this could be related to the global previous results showing that non difference between the two plants were observed in relation to these parameters [20, 22].

#### Mevalonate, terpenoid and carotenoid biosynthetic pathways

In the V/4xRL plants, the genes involved in the mevalonate acid and carotenoid biosynthetic pathways were differentially expressed while such differential expression was not observed in the V/2xRL plants (Figs 3 and 4). The 3-hydoxy-3-methylglutaryl reductase (HMG1) gene was repressed while the geranyl diphosphate synthase (GPS1) gene was overexpressed (-2.616 and 1.642 fold changes, respectively; Table 1, Fig 4). A recent work altering the expression of all the mevalonate pathway genes in the context of operon using CRISPRi system led to the striking of a balance between terpenoid production and cell growth [88]. Furthermore, non-volatile isoprenoids, such as carotenoids, and phenylpropanoids play a recognized antioxidant function in plant response to different environmental constrains, including drought stress [89]. Moreover, the carotenoid pathway is closely related to ABA biosynthesis pathway, which has been shown to be more involved in V/4xRL than in V/2xRL physiology [22]. In the V/2xRL plants, the MEP/terpenoid pathway was shifted to the overexpression of genes involved in chlorophyll degradation (NYE1, ACD1, 1.3 and 1.894 fold changes, respectively; Table 1, Fig 3); both proteins were identified as bottlenecks in the network central analysis (Table 1, S2 Table). In other works, chlorophyll degradation has been shown to be related to response to different stresses as drought [90], and some results showed that plants with higher chlorophyll content were also more resistant to stress [91]. The V/2xRL and V/4xRL plants presented different regulation pathways related to mevalonate, terpenoid and carotenoid biosynthesis (Figs 3 and 4). V/4xRL plants presented pathways directed to the carotenoids and ABA production that could be related to stomatal regulation and, consequently, lower water loss through transpiration (Figs 4 and 5). The V/2xRL plants may have less chlorophyll content leading to reduction of long term stress resistance (Figs 3 and 5).

#### Flavonoids

In the V/4xRL plants, genes of the flavonoid biosynthesis pathway (TT4 and TT7) were overexpressed (1.089 and 2.321 fold changes, respectively; Table 1, Fig 4) while no differential expression was observed in the V/2xRL plants (Fig 3). This results were coherent with other works showing that flavonoid over-accumulation was key to enhanced tolerance to abiotic stresses [92]. Considering that V/4xRL plants presented a general higher tolerance to drought, the flavonoids may be one of the important elements of the behavior difference between V/4xRL and V/2xRL plants. Moreover, the TT4 and TT7 proteins were identified as bottlenecks in the network centrality analysis (Table 1, S2 Table); TT4 was present in the ‘Isoprenoid biosynthesis process’ subnetwork (Fig 2B).

## Conclusion

When submitted to stress conditions the plants reprogram their metabolism and growth through different biosynthetic pathways. It was possible to observe that two distinct strategies were developed by the V/2xRL and V/4xRL plants submitted to the drought (Fig 5) in the experimental conditions used by Allario et al. [20]. The V/2xRL plant data indicated a reduction of the photosynthesis, reduction of sulfur assimilation leading to less protein synthesis, and increase of chlorophyll degradation. Taking into account that the V/2xRL combination presents a higher water extraction capacity in the soil [22], and that this plant maintain its development (e.g. growth), even under drought stress, it can be assume that the V/2xRL plant consumed all the fraction of available water before the V/4xRL. The RL genotype–when considered as *pied-franc*–shows an increase of the root system size in drought condition, with the objective of seeking water resources in deeper regions of the soil [93]. Considering that the V/RL plants were grown in confined conditions (e.g. pot), it may occurred that, after consuming the entire available water fraction, the V/2xRL plant did not have any possibility of water uptake, which resulted in the reduction of its photosynthetic efficiency, with subsequent foliar abscission and, consequently, plant death. In resume, even if the V/2xRL plant implement some tolerance mechanisms such as reduction of oxidative damages through the use of the alternative respiratory pathway, increase of osmoprotection and epigenetic regulation, the global plant response to drought was rapid and quickly exhaustive resulting in a general tendency to dehydration avoidance, which presented some advantage in short and strong drought stress conditions, but which, in long terms, does not allow the plant survival. At the contrary, the V/4xRL plants presented a higher ABA content when compared to the V/2xRL [20, 22], associated to the expression of genes related to carotenoid/ABA biosynthesis (Fig 5) leading to stomatal regulation and, consequently, lower water loss through transpiration. Thus, the V/4xRL combination loses less water by dewatering compared to the V/2xRL combination. This idea is reinforced by the higher content of wax in the V/4xRL plant (and less wax biosynthesis in V/2xRL plants), which is known to contribute to drought tolerance. In resume, the V/4xRL plants presented a response which impacts on development (e.g. plant size) but that present some advantages in prolonged drought. It is interesting to note that both combination activated genes related to auxin and brassinosteroid pathway, as well as to stomata regulation (closure, density and distribution) but with the involvement of different genes that may be related to fine tune of physiological regulation at cellular or organism level. Some similar differential responses (avoidance *vs* tolerance) were observed in other citrus genotypes (RL *pied-franc vs* Sunki Maravilha) in response to drought [93] indicating that each genotype and/or combination scion/rootstock would present a differential response to drought stress through activation of distinct gene and protein pathways. The response also depends of the location, duration and severity of the stress. Finally, here, some specific proteins, which presented high centrality on interactomic analysis (e.g. NYE1, ACD1, TPS1, ARFs, RHLs, CYPs, PDE334, LHCs, SIR, SHM1, OASB1, TFL2, EBS1 and ALDH3F1) could be good candidates for subsequent functional analysis of citrus genes related to drought response, as well as be good markers of one or another physiological mechanism implemented by the plants.

## Supporting information

S1 Table List of the genes differentially expressed on microarray according to Allario et al. (2013).
(DOCX)

S2 Table Centrality of the proteins from the global network. B: bottleneck; C: common; HB: hub-bottleneck.
(DOCX)

S3 Table List of the proteins present in the clusters showed in the Fig 2. (DOCX)

## Abbreviations

2x: diploid
4x: tetraploid
PPI: protein-protein interaction
PSI: photosystem I
PSII: photosystem II
RL: Rangpur lime
V: Valencia Delta sweet orange
