# Supplementary material for: Different adaptation strategies of two citrus scion/rootstock combinations in response to drought stress
Source: PLoS One. 2017 May 17;12(5):e0177993. doi: 10.1371/journal.pone.0177993 (PMC5435350; doi:10.1371/journal.pone.0177993)
Supplement: S1 Table — (2013). (DOCX) [file pone.0177993.s001.docx]

**S1 Table.** List of the genes differentially expressed on microarray according to Allario et al. (2013).

| V/2xRL | | V/4xRL | |
| --- | --- | --- | --- |
| Acession number | Fold change | Acession number | Fold change |
| KN0AAK3DD11 | 5.777535692 | C34209G11 | 4.333374704 |
| KN0AAA1BE09 | 5.709301424 | IC0AAA56DE05 | 4.296057649 |
| C05072A04 | 4.649901589 | IC0AAA19BG10 | 3.700710974 |
| KN0AAK3DG06 | 4.610367202 | IC0AAA21BB04 | 3.393256713 |
| C08028G03 | 4.424571925 | IC0AAA81CB07 | 3.314175923 |
| C31402D06 | 4.2936696 | KN0AAL1BB02 | 3.289253398 |
| C32202H07 | 4.191467004 | IC0AAA76CE03 | 3.28555915 |
| C04031B09 | 4.169873398 | C02020F07 | 3.281588103 |
| C34209G11 | 4.101119134 | C31208B12 | 3.252171377 |
| IC0AAA7DG09 | 3.970601637 | KN0AAP8YC14 | 3.149503087 |
| C06021A07 | 3.931382342 | IC0AAA23DD03 | 3.074588283 |
| IC0AAA48CD07 | 3.906270524 | C08007C09 | 3.046696616 |
| IC0AAA36AA04 | 3.795611376 | C31207G02 | 3.003166021 |
| KN0AAP4YC19 | 3.783601392 | KN0AAI2AH02 | 2.909501555 |
| C08002A07 | 3.781613294 | IC0AAA50DC09 | 2.745181369 |
| KN0AAQ1YC10 | 3.628696207 | C02007D09 | 2.721624802 |
| C34100C05 | 3.626953937 | IC0AAA31CB09 | 2.717408446 |
| C18006C10 | 3.565379776 | IC0AAA84AD04 | 2.714864084 |
| C18011D06 | 3.545948664 | IC0AAA45CC03 | 2.700228702 |
| C02025A10 | 3.508847995 | IC0AAA96AD04 | 2.634985873 |
| C02020F07 | 3.455450367 | C31709B05 | 2.609974395 |
| IC0AAA61BA08 | 3.350466091 | IC0AAA35CC06 | 2.603230849 |
| C18024B08 | 3.192406823 | KN0AAP1YH19 | 2.598276069 |
| C18010F11 | 3.157386954 | KN0AAQ13YE06 | 2.590892714 |
| IC0AAA5BH04 | 3.143779606 | IC0AAA21CF06 | 2.589179302 |
| IC0AAA31AE07 | 3.133368724 | IC0AAA32DG02 | 2.573998022 |
| IC0AAA37DG08 | 3.079041089 | C31402D06 | 2.539701012 |
| IC0AAA72CE04 | 3.077036311 | C04033H06 | 2.510866344 |
| C16012D07 | 3.072599645 | C08004G06 | 2.466118094 |
| C08028B07 | 3.051540023 | C18021G04 | 2.462610462 |
| C31803F06 | 3.036712321 | IC0AAA46BF09 | 2.448814037 |
| C31207C12 | 3.019285594 | C05811A03 | 2.435311259 |
| IC0AAA18CA12 | 2.993128839 | IC0AAA80AB06 | 2.425701155 |
| C04033H06 | 2.989839242 | C31502C10 | 2.397918235 |
| KN0AAB2CF11 | 2.959999109 | IC0AAA64BB03# | 2.396076704 |
| C34103C02 | 2.958503364 | IC0AAA7AF08 | 2.368960184 |
| C31203F11 | 2.890734845 | KN0AAH2CC01 | 2.354655792 |
| C31401E04 | 2.853686893 | C21007C11 | 2.32142317 |
| IC0AAA51AH01 | 2.841206408 | C05074G08 | 2.295074099 |
| C04011E09 | 2.818760718 | C06054B04 | 2.288039574 |
| C31208B12 | 2.817429216 | C08008H01 | 2.277274711 |
| C08026F02 | 2.806284893 | C04011E09 | 2.2395128 |
| IC0AAA8DA03 | 2.801181976 | IC0AAA85BA11 | 2.23352646 |
| C08007C09 | 2.774727154 | KN0AAQ1YO02 | 2.19814522 |
| IC0AAA70DA07 | 2.72673466 | C34103C02 | 2.153229143 |
| IC0AAA90AA03 | 2.726722273 | C34100C05 | 2.14541334 |
| C08008H06 | 2.701248129 | C31401E04 | 2.10901857 |
| IC0AAA31AA01 | 2.694259083 | C31203F11 | 2.06568669 |
| C32103H04 | 2.690255575 | IC0AAA8DA03 | 2.018425106 |
| C18021G04 | 2.670931271 | C02019G12 | 2.001364625 |
| C31106H08 | 2.648322562 | C18010F11 | 1.949279655 |
| IC0AAA12CF11 | 2.639024355 | C31207C07 | 1.939119557 |
| C08008H01 | 2.636553239 | C06017C03 | 1.934041071 |
| C31502C10 | 2.623102541 | KN0AAP5YB07 | 1.929490023 |
| IC0AAA11CB09 | 2.578961131 | KN0AAK2AE05 | 1.919453976 |
| C16016C04 | 2.577339009 | C31207D09 | 1.869014473 |
| C31207C07 | 2.56794416 | C16016C04 | 1.861065841 |
| C04029G04 | 2.556889445 | C31302B05 | 1.843278389 |
| IC0AAA86BB07 | 2.553763203 | C05075C09 | 1.826184129 |
| C08009G11 | 2.548786916 | C08036D01 | 1.801485629 |
| C31707G09 | 2.541312912 | C31106H08 | 1.762866562 |
| IC0AAA37DF09 | 2.532616237 | C04035E05 | 1.731330377 |
| C05073D01 | 2.514670452 | C05072D08 | 1.714570935 |
| C31208A08 | 2.509371003 | C34107H12 | 1.712505395 |
| C31106H02 | 2.50857068 | IC0AAA37DF09 | 1.711961887 |
| C31104C09 | 2.496940069 | C31104C09 | 1.708864788 |
| C08020F09 | 2.475836013 | C01020E02 | 1.690181945 |
| C05134C10 | 2.462511092 | C08011D05 | 1.674806926 |
| C31302B05 | 2.436204577 | C05136D03 | 1.648196658 |
| C05136D03 | 2.405478426 | IC0AAA90AC08 | 1.641980805 |
| C05808D08 | 2.393920683 | C04018D09 | 1.64126445 |
| KN0AAK2AE05 | 2.390819153 | C18019E01 | 1.630099197 |
| C05074C01 | 2.355939 | C31809D05 | 1.628645324 |
| KN0AAA1BG12 | 2.35421035 | C19004B01 | 1.618027295 |
| C31207D09 | 2.349614065 | C20003E08 | 1.617048199 |
| C04035E05 | 2.347141638 | C31707G09 | 1.61510477 |
| C04022A03 | 2.343429177 | C34004H11 | 1.6139711 |
| IC0AAA87DB03 | 2.327619388 | C04034C07 | 1.588317223 |
| KN0AAP5YB07 | 2.324937007 | C34209H07 | 1.559105484 |
| KN0AAK3BH12 | 2.313571469 | IC0AAA46AH09 | 1.544374781 |
| C08011D05 | 2.302541455 | C04013E10 | 1.543872784 |
| C32101G06 | 2.260460252 | C31106H02 | 1.538220369 |
| C31405G02 | 2.259909364 | C08029D05 | 1.500833884 |
| IC0AAA73CD10 | 2.24341642 | C31505C05 | 1.490039499 |
| IC0AAA47DF04 | 2.237300282 | C31003D02 | 1.48111576 |
| C08025G10 | 2.217314923 | C08035F07 | 1.471356806 |
| KN0AAP13YE12 | 2.216593599 | C18012F09 | 1.469887526 |
| C05075C11 | 2.208583796 | C08025G10 | 1.464007722 |
| C31703A05 | 2.204190521 | C21008D05 | 1.459890922 |
| C08017C08 | 2.202076582 | C01019C09 | 1.458174557 |
| KN0AAI1BD07 | 2.19512765 | C34209G03 | 1.428113922 |
| C18019D04 | 2.189300582 | C01019G09 | 1.427935393 |
| KN0AAB2DC06 | 2.182299948 | C05068E04 | 1.385612644 |
| C08032C08 | 2.173347604 | C32104G12 | 1.360788515 |
| C08035F07 | 2.161393749 | C31207D04 | 1.357684719 |
| C08007D08 | 2.158616667 | C31009D05 | 1.355282593 |
| C02019G12 | 2.146099714 | C31801G10 | 1.352942663 |
| C19004B01 | 2.145283221 | C34204F12 | 1.339534494 |
| IC0AAA39DF01 | 2.139457949 | C32102B08 | 1.336532081 |
| C34107H12 | 2.138360304 | IC0AAA63AB05 | 1.335365896 |
| C08036D01 | 2.128728739 | C31405G02 | 1.322644737 |
| C31803F10 | 2.101932267 | C32010D05 | 1.316731835 |
| C18023G08 | 2.098778932 | C31303B12 | 1.304815823 |
| IC0AAA35AF06 | 2.074678015 | C06003B11 | 1.30406897 |
| IC0AAA47CF07 | 2.072075419 | C19002C12 | 1.30078245 |
| C05133B12 | 2.069795302 | C18015E08 | 1.299008668 |
| KN0AAK2BB02 | 2.053789226 | C31808C01 | 1.283698212 |
| C04027H07 | 2.052289788 | C08036F01 | 1.27640629 |
| C18009G09 | 2.049528515 | C04027H07 | 1.264472016 |
| C04018D09 | 2.047480516 | C01019H06 | 1.255096834 |
| C31809D05 | 2.042076797 | C34206B03 | 1.24728577 |
| C21008D05 | 2.01030444 | KN0AAL2BB04 | 1.233353468 |
| C02008E07 | 1.990267733 | C31404H08 | 1.230317696 |
| C18012F09 | 1.976974611 | C18005E03 | 1.227800784 |
| C18019E01 | 1.971093933 | C04035A10 | 1.200742113 |
| IC0AAA46AH09 | 1.969981558 | C34208B09 | 1.189776463 |
| C18015E08 | 1.965042566 | C05076C10 | 1.189195426 |
| C01020E02 | 1.945585819 | C05065G09 | 1.182705805 |
| IC0AAA73AF05 | 1.938536928 | C34201F01 | 1.178760037 |
| C32102B08 | 1.928251214 | C32007C03 | 1.174753203 |
| C34106B01 | 1.925466774 | C31404A06 | 1.173216772 |
| C19004G08 | 1.915876107 | C34001F11 | 1.166164969 |
| KN0AAA1BA02 | 1.914054561 | C02026C02 | 1.161059952 |
| IC0AAA4AB03 | 1.911602785 | C34008C07 | 1.153261493 |
| C08029A02 | 1.893868647 | KN0AAK1DA01 | 1.15194129 |
| KN0AAP3YA21 | 1.893806786 | C31303B02 | 1.143438624 |
| C31808C01 | 1.878668134 | C16015G06 | 1.136977685 |
| KN0AAH3DD09 | 1.874727705 | C31204C05 | 1.11102928 |
| C34206B03 | 1.872996781 | C18015E04 | 1.108153156 |
| C06006A01 | 1.869344624 | C03001E10 | 1.096499984 |
| C04034D08 | 1.86323963 | C31808C07 | 1.090941967 |
| KN0AAA2BE02 | 1.854647861 | C32013G05 | 1.088531187 |
| C34007D01 | 1.845399916 | C08035G08 | 1.051198021 |
| C32201G02 | 1.845048182 | C19004G08 | 1.040875316 |
| C05068E04 | 1.837669749 | C31803F07 | 1.039153172 |
| C04035B07 | 1.831618326 | C04004C09 | 1.038660589 |
| C31303B12 | 1.813921198 | C32108D01 | 1.038364409 |
| C02026C02 | 1.800166462 | C04010B11 | 1.014988472 |
| C34004H11 | 1.790546653 | C16014H07 | 0.981100314 |
| C05075C09 | 1.777941144 | C31304D11 | 0.979735769 |
| C04035A10 | 1.775086196 | C02008D05 | 0.962160585 |
| C34208B04 | 1.770529451 | C31804G04 | 0.938447283 |
| IC0AAA10DH07 | 1.748033057 | C32005F03 | 0.928189584 |
| C06053H11 | 1.73113785 | C34202D11 | 0.927363717 |
| KN0AAK1DA01 | 1.716146439 | C34006A02 | 0.924641273 |
| C31003D02 | 1.715766539 | C34107H03 | 0.910675634 |
| IC0AAA79AE11 | 1.708260304 | C31206F10 | -0.84566981 |
| C05075H02 | 1.685423691 | C31701H09 | -0.88223301 |
| C19002C12 | 1.678733905 | C04017G03 | -0.88829233 |
| C06017C03 | 1.664274112 | C31403H07 | -0.90018124 |
| C18023B02 | 1.657556154 | C31708F05 | -0.90084702 |
| C08038G07 | 1.657006276 | IC0AAA5CD09 | -0.91622434 |
| C34204F12 | 1.656684488 | C05811B09 | -0.92432406 |
| C06003B11 | 1.645593712 | C31810G06 | -0.93226581 |
| C18012E09 | 1.636825235 | C31806H11 | -0.933895 |
| C31404H08 | 1.629088363 | C32001B06 | -0.94512231 |
| C08035B03 | 1.621045984 | C05070G03 | -0.96096575 |
| C02008D05 | 1.611284146 | C01018F10 | -0.96210664 |
| C18005E03 | 1.609397013 | IC0AAA24CB11 | -0.96273524 |
| C31204C05 | 1.602278107 | C32006C06 | -0.96424771 |
| KN0AAL2BB04 | 1.600962894 | C05076F06 | -0.97517654 |
| C08024A12 | 1.593454569 | C31404C01 | -0.98534504 |
| C34010E01 | 1.584697143 | C07010G07 | -0.99865341 |
| IC0AAA63AB05 | 1.579826788 | C31808C11 | -1.01949518 |
| C31403D05 | 1.566463068 | C16013C03 | -1.02245017 |
| C05065G09 | 1.548257316 | C31304H04 | -1.02778723 |
| C34205C03 | 1.527223752 | C31602F10 | -1.02847213 |
| C31404A06 | 1.52175456 | KN0AAP5YN15 | -1.03659989 |
| C31802D05 | 1.520358001 | C06001A06 | -1.04345302 |
| C34009B07 | 1.51674917 | C02013F09 | -1.05539484 |
| C34207F06 | 1.516471128 | C31305H08 | -1.06869137 |
| C21001B11 | 1.509981814 | C05056D02 | -1.09276096 |
| C31009D05 | 1.506302697 | C02015C07 | -1.10498668 |
| C01019G09 | 1.50119757 | C07007D04 | -1.11994184 |
| C34208D10 | 1.486855021 | C07002C08 | -1.12400975 |
| IC0AAA15AB03 | 1.481937551 | C34010E02 | -1.1398451 |
| C34101A03 | 1.478331745 | C02014F10 | -1.14529734 |
| IC0AAA40AC01 | 1.47774484 | C01017C12 | -1.14629511 |
| C05076C10 | 1.44583948 | C31505H05 | -1.15138289 |
| C34201F01 | 1.444933861 | C31601A01 | -1.16076872 |
| C18015E04 | 1.441038529 | C05056H01 | -1.1748131 |
| C05072D08 | 1.434818674 | C05802F08 | -1.17565929 |
| C34207A01 | 1.432937465 | C04004H06 | -1.1897175 |
| C32108D01 | 1.428113574 | C31701A02 | -1.19399655 |
| C08036F01 | 1.424699414 | C01012D05 | -1.21192395 |
| IC0AAA40BG02 | 1.422301496 | C02002F03 | -1.21248907 |
| C04034B12 | 1.421593926 | C02013F01 | -1.23522043 |
| C18015E06 | 1.416610614 | C08020F03 | -1.24150137 |
| C18004C08 | 1.416111609 | C31601H11 | -1.26153622 |
| C34101H12 | 1.413257904 | C32004E07 | -1.26322361 |
| C04035A03 | 1.405898513 | C08036C12 | -1.27820742 |
| C18004B12 | 1.404527139 | C07008E05 | -1.34152912 |
| C31802G03 | 1.39602207 | C01003C11 | -1.34618117 |
| C08002C09 | 1.391418248 | C32009A07 | -1.35364386 |
| C16015A09 | 1.384207432 | C31303E07 | -1.36696161 |
| C08035D05 | 1.380443395 | C04034B03 | -1.38563431 |
| C34107H03 | 1.377208653 | C31405H04 | -1.39964593 |
| IC0AAA71CG11 | 1.376835424 | C07003C08 | -1.40195269 |
| C06018C11 | 1.375266223 | KN0AAA3CC07 | -1.41386178 |
| C31403B04 | 1.372640198 | C04004H06 | -1.41404049 |
| C31005C03 | 1.370411519 | C05071C02 | -1.41972879 |
| C04013E10 | 1.366343123 | C31802C04 | -1.42170457 |
| C04030G01 | 1.365915918 | KN0AAI1CA01 | -1.4221028 |
| C04034C07 | 1.364131291 | C01013F06 | -1.43154103 |
| C18004A04 | 1.363076345 | C05068G03 | -1.45916001 |
| C31805F10 | 1.36241307 | KN0AAA3AE04 | -1.49167013 |
| C31001E09 | 1.360546601 | C02017H03 | -1.49847306 |
| C05065E02 | 1.360172865 | C31502D02 | -1.51386796 |
| C31705F01 | 1.358295657 | C16000A10 | -1.53750301 |
| C31803F07 | 1.358212419 | C04004H06 | -1.53875084 |
| C08029D05 | 1.356438489 | C18007F05 | -1.54379284 |
| C34209H07 | 1.353866722 | C04004H06 | -1.55220418 |
| C34007H07 | 1.343925615 | C04004H06 | -1.5573227 |
| C34103F12 | 1.337635233 | KN0AAI3BD03 | -1.56896979 |
| C01019H06 | 1.331548052 | C02023C12 | -1.5757758 |
| C31403A03 | 1.314517889 | KN0AAQ6YP16 | -1.57626931 |
| C18017H11 | 1.313812027 | C04004H06 | -1.58653155 |
| C08001H09 | 1.31099411 | C31802F07 | -1.60880993 |
| C16015G06 | 1.307701362 | KN0AAI2DE02 | -1.63957051 |
| KN0AAH2DF06 | 1.307040343 | C31402E11 | -1.65765422 |
| C31804G04 | 1.304047461 | C04023E07 | -1.66307572 |
| C01014E05 | 1.302728095 | C04004H06 | -1.67083761 |
| C32104G12 | 1.302395367 | C31703E12 | -1.67467987 |
| C02026E07 | 1.299649683 | C05057G09 | -1.69165612 |
| C31100B11 | 1.293935863 | C04004H06 | -1.70520358 |
| C02021B05 | 1.286925786 | C04004H06 | -1.73477101 |
| C08007E09 | 1.286128368 | C04004H06 | -1.74634753 |
| C31304D11 | 1.283782128 | C04004H06 | -1.75190713 |
| C02011H08 | 1.280939406 | C04004H06 | -1.77277302 |
| C31502C07 | 1.27930736 | C04004H06 | -1.79786746 |
| C05811H10 | 1.27741146 | C32011E02 | -1.79891123 |
| IC0AAA28AB11 | 1.273957536 | C31603D03 | -1.82941951 |
| C04013A11 | 1.266528747 | C31008F07 | -1.8596796 |
| C08035G08 | 1.265993137 | C16012H12 | -1.87805248 |
| C32007C03 | 1.265546874 | C31302A07 | -1.90417057 |
| C34109E03 | 1.264517949 | C05057H10 | -1.90744212 |
| C34202D11 | 1.26443834 | C05069E05 | -1.91698191 |
| C20002A04 | 1.259394271 | C08032B07 | -1.92288154 |
| C34009E01 | 1.258701559 | C01015D11 | -1.93903649 |
| C05141F05 | 1.252652436 | C06019F11 | -1.94688426 |
| C07010E12 | 1.252310048 | C01008H04 | -1.96766332 |
| C34207E09 | 1.249840197 | C04004H06 | -1.97498012 |
| C01011B08 | 1.248531834 | IC0AAA20AC04 | -2.05810904 |
| C04035H04 | 1.247685318 | C04004H06 | -2.06105984 |
| C34107C11 | 1.247280935 | C31504C12 | -2.07795083 |
| C07008C05 | 1.245963723 | C05057A11 | -2.07938632 |
| C04006B06 | 1.242759408 | C08018D04 | -2.09858444 |
| C31707B03 | 1.240231154 | C34100H01 | -2.11184628 |
| C31003F02 | 1.236499733 | C34002G02 | -2.12858665 |
| C16015A05 | 1.235279788 | KN0AAB1DE04 | -2.17450902 |
| C18004H09 | 1.230458949 | C08001H08 | -2.1877296 |
| C31103D11 | 1.228973991 | C1 (cal1) | -2.19684499 |
| KN0AAP8YN01 | 1.22170778 | C08011G05 | -2.20081081 |
| C18021C03 | 1.220957327 | C19003H10 | -2.25751516 |
| C34110F10 | 1.21696111 | C18023H12 | -2.2815494 |
| C05075C10 | 1.2091017 | C02019E04 | -2.28278004 |
| KN0AAP9YM02 | 1.208798572 | IC0AAA56CC11 | -2.28305927 |
| C08011B05 | 1.202240431 | C02023H07 | -2.28813384 |
| C06014D04 | 1.201449765 | IC0AAA20CG05 | -2.29634028 |
| C34010E08 | 1.194969751 | KN0AAA1AE07 | -2.33161644 |
| C01012E09 | 1.194846926 | C31102C12 | -2.34278841 |
| IC0AAA43DA02 | 1.193224423 | C08015C05 | -2.37094088 |
| IC0AAA67CC07 | 1.190737122 | KN0AAP7YM03 | -2.37111423 |
| C08018F03 | 1.190406114 | C21008F12 | -2.37681985 |
| C03001E10 | 1.189684851 | IC0AAA20BG12% | -2.41123542 |
| C31204B02 | 1.182821171 | IC0AAA39AF07 | -2.41215509 |
| C02019C10 | 1.182634646 | C31604E05 | -2.41574224 |
| C04006E11 | 1.173221893 | C18009E10 | -2.42680602 |
| IC0AAA1CB06 | 1.171046928 | C34109B02 | -2.4481519 |
| C34009C08 | 1.17097982 | C02003F06 | -2.45166115 |
| IC0AAA17DE06 | 1.170749201 | C21004G03 | -2.45628557 |
| C01020H05 | 1.168181323 | CcGA20ox1 | -2.46453528 |
| C02015E10 | 1.163802259 | IC0AAA14CE10 | -2.47460193 |
| C31502B06 | 1.163780818 | IC0AAA14AB04 | -2.50115117 |
| C31504B08 | 1.162100446 | C08024C03 | -2.50227399 |
| C08011A09 | 1.159273516 | KN0AAP11YP14 | -2.51362158 |
| C05065G08 | 1.15877548 | C03006F07 | -2.52153796 |
| C01018G04 | 1.154390777 | C08011H04 | -2.52347417 |
| C31207D04 | 1.152596385 | C18015B05 | -2.52441332 |
| C04010G10 | 1.151038246 | IC0AAA49CC09 | -2.52497044 |
| C01020B10 | 1.150494473 | IC0AAA43CH12 | -2.52581754 |
| C32005F03 | 1.149550417 | IC0AAA12CC07 | -2.53472571 |
| C05802D05 | 1.139499835 | C2 (cal2) | -2.54249819 |
| C01016D07 | 1.138513607 | C07012B07 | -2.54816854 |
| C31006C04 | 1.133629333 | C31807A01 | -2.56373131 |
| IC0AAA35AC01 | 1.13284048 | C31304G05 | -2.57632862 |
| C06001G12 | 1.1308233 | C01012H03 | -2.58614733 |
| C31603A08 | 1.12849127 | C18011F08 | -2.60539893 |
| C34008C07 | 1.126489009 | IC0AAA41DG07 | -2.61609434 |
| C05076H07 | 1.115896091 | KN0AAP11YH09 | -2.61745367 |
| C31808C07 | 1.112981998 | KN0AAP3YO13 | -2.62947374 |
| C31207A03 | 1.107948663 | C31707H07 | -2.63645163 |
| C18015G07 | 1.105991659 | IC0AAA37BD03 | -2.64601793 |
| C02027F11 | 1.103050064 | C31804D06 | -2.64636796 |
| C31103F09 | 1.098681601 | C05057A04 | -2.65190055 |
| C31810D08 | 1.097818096 | C08001G12 | -2.69238138 |
| C06020A06 | 1.093437311 | C21001G01 | -2.69770528 |
| C34210F03 | 1.079529964 | IC0AAA33BF11 | -2.71235586 |
| C08008G07 | 1.079515247 | C08020C10 | -2.71257776 |
| C32010D05 | 1.072781727 | KN0AAP4YG14 | -2.73350257 |
| C06018E06 | 1.071243538 | C01015F08 | -2.73640925 |
| C18006G01 | 1.068831162 | KN0AAP2YO03 | -2.73841914 |
| C34207H04 | 1.068691404 | C31402H07 | -2.75236299 |
| C01014C09 | 1.066048594 | C31704G03 | -2.76291452 |
| C31505G12 | 1.060684921 | C32013G09 | -2.77833058 |
| C01017A05 | 1.056520982 | IC0AAA3CG07 | -2.78327768 |
| C08002A11 | 1.054555339 | C08001B06 | -2.78327895 |
| C31801G09 | 1.053114162 | C02012D08 | -2.80667708 |
| C18005G07 | 1.048382985 | C01009F12 | -2.82515234 |
| C34209G03 | 1.041017871 | KN0AAB1AF06 | -2.82892205 |
| C06008E06 | 1.039718093 | KN0AAI2AG12 | -2.8886473 |
| IC0AAA84BC03 | 1.035091949 | IC0AAA24CG06 | -2.92370612 |
| C06023F02 | 1.033984673 | C31708B05 | -2.94146885 |
| C05065E11 | 1.033353271 | C08004G08 | -2.95181877 |
| C04010D09 | 1.033130122 | IC0AAA34DC12 | -3.01769575 |
| C32103G11 | 1.032296532 | C34209F10 | -3.02075976 |
| IC0AAA68BE12 | 1.030021731 | KN0AAP4YN05 | -3.0297512 |
| C06016F10 | 1.024824328 | IC0AAA32AC03 | -3.03248129 |
| C01013F04 | 1.024462804 | IC0AAA25DC02 | -3.08155302 |
| IC0AAA38DD10 | 1.022216235 | KN0AAA2BG05 | -3.14039332 |
| C04023D07 | 1.016837827 | KN0AAI2DD08 | -3.14776684 |
| C16012A05 | 1.013203039 | IC0AAA18DF09 | -3.20688796 |
| C32010E06 | 1.010486606 | IC0AAA35DC01 | -3.2523107 |
| C05071E05 | 1.007723604 | C01017B04 | -3.33663466 |
| C04017E10 | 1.004378021 | C31305G10 | -3.3847748 |
| C03001B03 | 1.001568389 | KN0AAP10YF23 | -3.45097144 |
| C04026C09 | 0.996725621 | C32108G11 | -3.46398717 |
| C08034G07 | 0.995830851 | C05065C12 | -3.46637381 |
| C18005C05 | 0.993633275 | C32010C03 | -3.4836061 |
| IC0AAA30BG02 | 0.993186341 | IC0AAA75DA11 | -3.49965799 |
| IC0AAA73BE05 | 0.990254925 | C04026G03 | -3.5985493 |
| KN0AAQ6YM10 | 0.990203004 | C07003E04 | -3.61284911 |
| C08025D07 | 0.989508338 | KN0AAP6YP01 | -3.75106094 |
| C31703C06 | 0.989283009 | C32001C06 | -3.97796623 |
| C18015E05 | 0.987569287 | IC0AAA65CE05 | -3.99667225 |
| C08039A10 | 0.985992971 | C32009D05 | -4.37140894 |
| C02011A08 | 0.982865766 | IC0AAA13AE09 | -4.45586651 |
| C05069A12 | 0.982824745 | C06011C07 | -4.73307359 |
| C05808D01 | 0.980440736 | C05074B09 | -4.74816205 |
| C34010C04 | 0.979634777 | C32006E04 | -5.02691228 |
| C02023C11 | 0.976365092 | C31202C03 | -5.19440822 |
| C34205B09 | 0.972048134 | IC0AAA51AH01 | -5.21727524 |
| C31708D11 | 0.972036821 |  |  |
| KN0AAQ4YJ15 | 0.970946874 |  |  |
| C31702G12 | 0.96759636 |  |  |
| KN0AAP13YG22 | 0.967179216 |  |  |
| C31002B10 | 0.957137274 |  |  |
| IC0AAA57CB06 | 0.956161394 |  |  |
| C04035E01 | 0.953933313 |  |  |
| C08038C02 | 0.949472598 |  |  |
| C18025B06 | 0.947573021 |  |  |
| C31706C08 | 0.944444949 |  |  |
| C02024G07 | 0.944179965 |  |  |
| IC0AAA38AE09 | 0.940869393 |  |  |
| KN0AAP3YA21 | 0.9400521 |  |  |
| C05074B02 | 0.93992546 |  |  |
| C31703F01 | 0.937417395 |  |  |
| C31801B03 | 0.937150267 |  |  |
| C31504H07 | 0.936925129 |  |  |
| KN0AAP1YF16 | 0.935098177 |  |  |
| C31008H08 | 0.934585052 |  |  |
| C05073F04 | 0.929847553 |  |  |
| C31504H09 | 0.929817491 |  |  |
| C31804B08 | 0.929041391 |  |  |
| C02018F04 | 0.925319468 |  |  |
| C01005C05 | 0.924130157 |  |  |
| C34110A07 | 0.923802842 |  |  |
| C31801G10 | 0.921290536 |  |  |
| KN0AAP1YE17 | 0.920433142 |  |  |
| KN0AAQ13YH02 | 0.913956274 |  |  |
| C02008C12 | 0.905427334 |  |  |
| C02002C08 | 0.902179174 |  |  |
| C31705B10 | 0.901382031 |  |  |
| C02002B06 | 0.897687056 |  |  |
| C31403E06 | 0.895460994 |  |  |
| C16013F10 | 0.894411032 |  |  |
| C06022G11 | 0.8941947 |  |  |
| C31208G10 | 0.890340605 |  |  |
| IC0AAA78BC09 | 0.889607007 |  |  |
| C07012H07 | 0.886058883 |  |  |
| C31402D08 | 0.885966634 |  |  |
| C05057E03 | 0.880215979 |  |  |
| KN0AAP7YK07 | 0.879431533 |  |  |
| KN0AAP8YC14 | 0.878289755 |  |  |
| C34005A01 | 0.875904309 |  |  |
| C02008H08 | 0.866730126 |  |  |
| C08036G08 | 0.863424256 |  |  |
| C04033D12 | 0.861546927 |  |  |
| C34102H10 | 0.858856516 |  |  |
| C31601G07 | 0.857147201 |  |  |
| C31301B10 | 0.852294892 |  |  |
| C31108H10 | 0.850781084 |  |  |
| C01009G06 | 0.843243448 |  |  |
| C32005G09 | 0.839048053 |  |  |
| C08007C02 | 0.833661461 |  |  |
| C34004G03 | 0.830626866 |  |  |
| C03008G04 | 0.825996407 |  |  |
| KN0AAK1DB08 | 0.820449301 |  |  |
| C20006G06 | 0.818786216 |  |  |
| C18016C06 | 0.814373807 |  |  |
| C34204G10 | 0.805254084 |  |  |
| KN0AAP5YJ19 | 0.803788651 |  |  |
| C31604D01 | 0.802524947 |  |  |
| C34106F07 | 0.798991464 |  |  |
| C31501A01 | 0.797233736 |  |  |
| C31303B02 | 0.794995222 |  |  |
| C34202F05 | 0.793149746 |  |  |
| C31503B04 | 0.7927691 |  |  |
| C31004B03 | 0.791619241 |  |  |
| C31207B02 | 0.789203993 |  |  |
| C01009F06 | 0.786018632 |  |  |
| C31605F06 | 0.779344844 |  |  |
| C02011B01 | 0.776948654 |  |  |
| C20008G07 | 0.774426123 |  |  |
| C02024C08 | 0.772155321 |  |  |
| C04022A06 | 0.769320706 |  |  |
| C05055C10 | 0.769167209 |  |  |
| C32003C06 | 0.758049453 |  |  |
| C16014H07 | 0.756981191 |  |  |
| C05809B01 | 0.755754088 |  |  |
| IC0AAA22CG09 | 0.752402998 |  |  |
| C07003B08 | 0.752390814 |  |  |
| KN0AAP4YG01 | 0.751731511 |  |  |
| C34006C04 | 0.732340379 |  |  |
| C34204F09 | 0.731073732 |  |  |
| IC0AAA67DG09 | 0.729279418 |  |  |
| C02016E09 | 0.727549901 |  |  |
| C34207E11 | 0.726432597 |  |  |
| C07012D01 | 0.720575652 |  |  |
| C08020E04 | 0.678564624 |  |  |
| C31801A12 | -0.68967619 |  |  |
| C31605A08 | -0.69162124 |  |  |
| C01016B03 | -0.69764117 |  |  |
| C31604D03 | -0.7127786 |  |  |
| C05057A09 | -0.71882386 |  |  |
| KN0AAP6YJ14 | -0.72165028 |  |  |
| C31805B05 | -0.72552684 |  |  |
| C05065A02 | -0.72835423 |  |  |
| KN0AAP10YO16 | -0.73133617 |  |  |
| C31503F01 | -0.74067844 |  |  |
| C06052C02 | -0.74488668 |  |  |
| C16013H11 | -0.75865394 |  |  |
| C31601H03 | -0.76554192 |  |  |
| C34209F12 | -0.76907682 |  |  |
| C08005F09 | -0.77233429 |  |  |
| C05076E10 | -0.77590759 |  |  |
| C31705D07 | -0.77879469 |  |  |
| C06014H01 | -0.78093653 |  |  |
| C32104D04 | -0.78151298 |  |  |
| C06016F08 | -0.78275095 |  |  |
| C31009A08 | -0.79236867 |  |  |
| C01009E11 | -0.79516771 |  |  |
| C03007B07 | -0.79702945 |  |  |
| C31707B07 | -0.79917076 |  |  |
| C01009A05 | -0.80836943 |  |  |
| C32003A11 | -0.80867083 |  |  |
| C20004B05 | -0.80998763 |  |  |
| C34109A11 | -0.81383657 |  |  |
| C31503A07 | -0.81904573 |  |  |
| C32001D11 | -0.82025675 |  |  |
| C01018F10 | -0.82265123 |  |  |
| IC0AAA26AF03 | -0.82530427 |  |  |
| C02017H03 | -0.82969885 |  |  |
| C07005A04 | -0.83207108 |  |  |
| C32007A12 | -0.83420558 |  |  |
| C31602F10 | -0.83674133 |  |  |
| C04027H02 | -0.83933838 |  |  |
| C31006G09 | -0.84131798 |  |  |
| C05139A05 | -0.84684869 |  |  |
| C34003B03 | -0.84768779 |  |  |
| C31004H06 | -0.84927139 |  |  |
| C31002F01 | -0.85253269 |  |  |
| C34004E04 | -0.85306679 |  |  |
| C18001E11 | -0.85857483 |  |  |
| C01009G02 | -0.86086451 |  |  |
| C05002A03 | -0.86265425 |  |  |
| C05054A09 | -0.86670861 |  |  |
| C31007E04 | -0.86713072 |  |  |
| C31702H12 | -0.87171432 |  |  |
| C20008A02 | -0.871879 |  |  |
| C04004H06 | -0.87390271 |  |  |
| C01017F11 | -0.8752945 |  |  |
| C02016E06 | -0.87704986 |  |  |
| C07003H08 | -0.88743858 |  |  |
| C08005B03 | -0.89152852 |  |  |
| C34109F01 | -0.89537708 |  |  |
| C04032G12 | -0.89689455 |  |  |
| C05076E11 | -0.89879875 |  |  |
| C04004H06 | -0.89978615 |  |  |
| C31601F06 | -0.9017408 |  |  |
| IC0AAA48DB11 | -0.90183225 |  |  |
| C31302G04 | -0.90301624 |  |  |
| C31501D05 | -0.90321239 |  |  |
| C16005G04 | -0.90328981 |  |  |
| C34205B10 | -0.90336321 |  |  |
| C04027E01 | -0.9071569 |  |  |
| C02023E11 | -0.91219241 |  |  |
| C31804A05 | -0.91264559 |  |  |
| C31702F01 | -0.91365788 |  |  |
| C05131H11 | -0.91550604 |  |  |
| C34207H06 | -0.91668504 |  |  |
| C04011F11 | -0.92083672 |  |  |
| C20007G06 | -0.92539959 |  |  |
| C05131E06 | -0.92731294 |  |  |
| C01002A10 | -0.92973604 |  |  |
| C32003G02 | -0.92993687 |  |  |
| C01002A01 | -0.94302217 |  |  |
| C01018A04 | -0.95247006 |  |  |
| C01008D11 | -0.9634568 |  |  |
| C08007F12 | -0.96357658 |  |  |
| C31503G01 | -0.96611985 |  |  |
| C31501C06 | -0.96639719 |  |  |
| C01011G01 | -0.9665761 |  |  |
| C07007G05 | -0.97530935 |  |  |
| C34206G04 | -0.97582831 |  |  |
| C08033F04 | -0.98466435 |  |  |
| C05075A03 | -0.9846953 |  |  |
| C31007B05 | -0.98472622 |  |  |
| C31404C01 | -0.98619142 |  |  |
| C20010H06 | -0.98969603 |  |  |
| IC0AAA78DF05 | -0.99100556 |  |  |
| IC0AAA27DB05 | -0.99182393 |  |  |
| C32105C04 | -0.99293618 |  |  |
| C01003D04 | -0.99414524 |  |  |
| KN0AAP5YA02 | -0.99514452 |  |  |
| C31200C01 | -0.99597089 |  |  |
| C32008C11 | -0.99612335 |  |  |
| C06019C03 | -0.99703503 |  |  |
| IC0AAA41CA10 | -0.99770029 |  |  |
| C18007H09 | -1.00170774 |  |  |
| C04029A07 | -1.00402262 |  |  |
| C01012B03 | -1.00402859 |  |  |
| C03004F01 | -1.00584226 |  |  |
| C31304C06 | -1.00700039 |  |  |
| C08004E02 | -1.01138097 |  |  |
| C06020E02 | -1.01161326 |  |  |
| C08016C11 | -1.01179971 |  |  |
| C05073H08 | -1.01246685 |  |  |
| C04004H06 | -1.01423892 |  |  |
| C16012G05 | -1.01494819 |  |  |
| C34105G10 | -1.01509931 |  |  |
| C01003E03 | -1.0207169 |  |  |
| C34106D11 | -1.02337641 |  |  |
| C01017F05 | -1.02342534 |  |  |
| C34100A05 | -1.02462936 |  |  |
| C07009H11 | -1.02837946 |  |  |
| C34010A03 | -1.03337922 |  |  |
| C32001B06 | -1.0379711 |  |  |
| C08012C07 | -1.03806046 |  |  |
| C34005E02 | -1.03854014 |  |  |
| C05133C09 | -1.0386664 |  |  |
| C01008G07 | -1.04098397 |  |  |
| C31005C10 | -1.04616509 |  |  |
| C32103G05 | -1.0506561 |  |  |
| C31502D02 | -1.05371966 |  |  |
| C06018C04 | -1.05419958 |  |  |
| C31303F07 | -1.05440173 |  |  |
| IC0AAA66CD02 | -1.05536216 |  |  |
| C05071C02 | -1.05568171 |  |  |
| C05056H02 | -1.06356645 |  |  |
| C31701C02 | -1.06411157 |  |  |
| C31805A05 | -1.06471488 |  |  |
| C31504D11 | -1.06480688 |  |  |
| C03011H08 | -1.06584246 |  |  |
| C07008A11 | -1.06680885 |  |  |
| C02013F09 | -1.0672337 |  |  |
| C31007C10 | -1.0686366 |  |  |
| C20005F02 | -1.07646084 |  |  |
| C01009E01 | -1.07720719 |  |  |
| C04004H06 | -1.08026958 |  |  |
| C31504G02 | -1.08037984 |  |  |
| C31304H04 | -1.08045372 |  |  |
| C31504H10 | -1.08133698 |  |  |
| C04004H06 | -1.08412622 |  |  |
| C01003F07 | -1.08470479 |  |  |
| C07001C08 | -1.09190976 |  |  |
| C06017B07 | -1.09395761 |  |  |
| C31601A01 | -1.09514778 |  |  |
| C32004E07 | -1.09639555 |  |  |
| C31702D04 | -1.09898483 |  |  |
| C06004D02 | -1.10200291 |  |  |
| KN0AAP2YD02 | -1.10474952 |  |  |
| C08020F03 | -1.1055829 |  |  |
| C03011H07 | -1.10572023 |  |  |
| C01005A04 | -1.10719855 |  |  |
| C01020H02 | -1.10727113 |  |  |
| C34103H04 | -1.10860905 |  |  |
| C32107F12 | -1.11654878 |  |  |
| C31403H07 | -1.12173973 |  |  |
| C02014F10 | -1.12308431 |  |  |
| C04006F06 | -1.12560852 |  |  |
| C32005E07 | -1.12664378 |  |  |
| C05138E09 | -1.13087344 |  |  |
| C05076F06 | -1.13370427 |  |  |
| IC0AAA45BA06 | -1.14006469 |  |  |
| C05803E06 | -1.1540314 |  |  |
| C01016C07 | -1.15520152 |  |  |
| C31705G10 | -1.16160139 |  |  |
| IC0AAA48CE01 | -1.16227348 |  |  |
| C05056E09 | -1.16243696 |  |  |
| C01013C10 | -1.16458063 |  |  |
| C31810G06 | -1.16550196 |  |  |
| C08016D05 | -1.16617422 |  |  |
| C05804A10 | -1.16772706 |  |  |
| C05803F12 | -1.16955969 |  |  |
| C06023A05 | -1.17257007 |  |  |
| KN0AAP1YP13 | -1.17726019 |  |  |
| C31009F01 | -1.18292145 |  |  |
| C08039H10 | -1.18860928 |  |  |
| C07007D05 | -1.1896244 |  |  |
| C02022D07 | -1.1919167 |  |  |
| IC0AAA55AF05 | -1.19591292 |  |  |
| C05076A12 | -1.1984749 |  |  |
| C02013E02 | -1.20064022 |  |  |
| C08012E01 | -1.20769388 |  |  |
| C16013D10 | -1.21256883 |  |  |
| C18004G07 | -1.21458689 |  |  |
| C31001E04 | -1.21758714 |  |  |
| C01006A06 | -1.22031923 |  |  |
| C31404A07 | -1.22209948 |  |  |
| C31009D09 | -1.22512923 |  |  |
| C05056D02 | -1.2287212 |  |  |
| C01002F08 | -1.2288447 |  |  |
| C31503G11 | -1.23119797 |  |  |
| C31505H05 | -1.23669527 |  |  |
| C32006C06 | -1.24148284 |  |  |
| C31601A12 | -1.24406786 |  |  |
| C16013C03 | -1.24548805 |  |  |
| C16016F10 | -1.24830914 |  |  |
| C20004C04 | -1.26225739 |  |  |
| C05802F08 | -1.26970207 |  |  |
| C31708F11 | -1.27926684 |  |  |
| C31603A10 | -1.28110671 |  |  |
| C02017B02 | -1.2818881 |  |  |
| C01020E08 | -1.28947177 |  |  |
| C04011H06 | -1.29488789 |  |  |
| C04004H06 | -1.29551664 |  |  |
| C07004A03 | -1.29716516 |  |  |
| C05809F01 | -1.31230042 |  |  |
| C31007H03 | -1.31701449 |  |  |
| C01019F09 | -1.31767783 |  |  |
| KN0AAA3AE04 | -1.31860895 |  |  |
| C04004H06 | -1.3252447 |  |  |
| C01011C12 | -1.32570575 |  |  |
| C31702A08 | -1.33035479 |  |  |
| C31101F03 | -1.33837461 |  |  |
| C01009B04 | -1.34018201 |  |  |
| C05072A10 | -1.34286154 |  |  |
| C04004H06 | -1.34367498 |  |  |
| C04023E07 | -1.34518352 |  |  |
| C04004H06 | -1.35177964 |  |  |
| C18007G06 | -1.3595065 |  |  |
| C16000A10 | -1.36258385 |  |  |
| C31808C11 | -1.36647872 |  |  |
| C31701H09 | -1.36650037 |  |  |
| C04004H06 | -1.37054821 |  |  |
| C04004H06 | -1.37307531 |  |  |
| C31701B06 | -1.38531358 |  |  |
| C04004H06 | -1.38536297 |  |  |
| C08031D04 | -1.3891744 |  |  |
| C31209A05 | -1.39040843 |  |  |
| C01014H01 | -1.39431893 |  |  |
| C19003F01 | -1.40205998 |  |  |
| C31601H11 | -1.40686281 |  |  |
| C07008E05 | -1.4138246 |  |  |
| C31701A02 | -1.41576872 |  |  |
| C16012E06 | -1.41863865 |  |  |
| C01013A10 | -1.41872603 |  |  |
| C31007A10 | -1.4209818 |  |  |
| C02024G06 | -1.42186479 |  |  |
| C01003D10 | -1.42282651 |  |  |
| C31102G04 | -1.42688794 |  |  |
| C31010E11 | -1.42879824 |  |  |
| KN0AAA3CC07 | -1.43196923 |  |  |
| C34107F11 | -1.43555349 |  |  |
| C31806H11 | -1.43755409 |  |  |
| C01017G01 | -1.43874232 |  |  |
| C08034B04 | -1.4535879 |  |  |
| IC0AAA41AG09 | -1.4625369 |  |  |
| C31604G05 | -1.46902279 |  |  |
| C19003E01 | -1.47401572 |  |  |
| C02021D07 | -1.47453276 |  |  |
| IC0AAA5CD09 | -1.47638127 |  |  |
| C01008A03 | -1.47759186 |  |  |
| C05801E01 | -1.49823722 |  |  |
| KN0AAB1CA09 | -1.51619774 |  |  |
| C01003C11 | -1.53896231 |  |  |
| C04004H06 | -1.54001472 |  |  |
| C07004C07 | -1.55470538 |  |  |
| C32008B06 | -1.56975811 |  |  |
| C05056H01 | -1.57407866 |  |  |
| C04004H06 | -1.57627927 |  |  |
| C07002C08 | -1.58350233 |  |  |
| C31303E07 | -1.58425079 |  |  |
| KN0AAP5YN15 | -1.58831141 |  |  |
| C31209E08 | -1.59049928 |  |  |
| C08038E06 | -1.59863479 |  |  |
| KN0AAB3AH08 | -1.60103758 |  |  |
| C06001E08 | -1.61490891 |  |  |
| KN0AAQ6YP16 | -1.61667011 |  |  |
| C04004H06 | -1.61737441 |  |  |
| C02025G09 | -1.62091677 |  |  |
| C08007G08 | -1.62976773 |  |  |
| KN0AAI2DE02 | -1.63875873 |  |  |
| IC0AAA64AD10 | -1.64429866 |  |  |
| C04004H06 | -1.64870953 |  |  |
| C06020H11 | -1.65211013 |  |  |
| C01019A07 | -1.65295767 |  |  |
| C07010G07 | -1.65449108 |  |  |
| C20005D02 | -1.6588612 |  |  |
| C01013F06 | -1.67163058 |  |  |
| C31603H06 | -1.69023635 |  |  |
| C31710H10 | -1.69382332 |  |  |
| C04004H06 | -1.70038665 |  |  |
| C04004H06 | -1.70168531 |  |  |
| C34101E02 | -1.70196593 |  |  |
| C16012D10 | -1.70492417 |  |  |
| C31103F06 | -1.70574667 |  |  |
| C04004H06 | -1.70697723 |  |  |
| C08015D03 | -1.71991065 |  |  |
| C07003C08 | -1.72167587 |  |  |
| C04004H06 | -1.72317981 |  |  |
| C31802F07 | -1.72475904 |  |  |
| C04004H06 | -1.7350421 |  |  |
| C05138A12 | -1.73891785 |  |  |
| C31402C06 | -1.75825969 |  |  |
| KN0AAI3BD03 | -1.76164654 |  |  |
| C01006A03 | -1.76452654 |  |  |
| C05054A05 | -1.77311289 |  |  |
| C06019F11 | -1.78694039 |  |  |
| C31701G04 | -1.78984446 |  |  |
| KN0AAP1YK16 | -1.79515392 |  |  |
| C31703D08 | -1.804301 |  |  |
| C01020D08 | -1.83482365 |  |  |
| C31708F05 | -1.84692097 |  |  |
| IC0AAA13BA07 | -1.84782656 |  |  |
| C05140E02 | -1.8585892 |  |  |
| IC0AAA3BG11 | -1.86217958 |  |  |
| C01017C12 | -1.86295517 |  |  |
| C34010E02 | -1.86702644 |  |  |
| IC0AAA93BE11 | -1.87037346 |  |  |
| C01012D05 | -1.87687067 |  |  |
| C05811G02 | -1.88051532 |  |  |
| KN0AAI3CH05 | -1.88525079 |  |  |
| C31008F07 | -1.88530988 |  |  |
| KN0AAP8YG09 | -1.89084306 |  |  |
| C05073D09 | -1.89120897 |  |  |
| C07010B11 | -1.89384227 |  |  |
| IC0AAA31CF06 | -1.90107128 |  |  |
| C04004H06 | -1.9077098 |  |  |
| C31305H08 | -1.91404884 |  |  |
| IC0AAA20AB05 | -1.91432758 |  |  |
| C04004H06 | -1.92360485 |  |  |
| C31405A10 | -1.92774312 |  |  |
| IC0AAA95AB07 | -1.93331725 |  |  |
| C07007D04 | -1.93655713 |  |  |
| IC0AAA87DE04 | -1.94614056 |  |  |
| C21005D07 | -1.94930287 |  |  |
| C31602A09 | -1.9579385 |  |  |
| KN0AAK1BF01 | -1.95813147 |  |  |
| C31603C09 | -1.95945133 |  |  |
| C07012D04 | -1.96628711 |  |  |
| C04004H06 | -1.96830241 |  |  |
| C31402E11 | -1.97178642 |  |  |
| C05069D02 | -1.98065303 |  |  |
| IC0AAA10CH03 | -1.99047 |  |  |
| IC0AAA61AB02 | -1.99972124 |  |  |
| C08009F06 | -2.0000511 |  |  |
| C21005D05 | -2.00566674 |  |  |
| KN0AAB1CC04 | -2.01043174 |  |  |
| C04004H06 | -2.0257883 |  |  |
| C34207D12 | -2.02837002 |  |  |
| C20001H03 | -2.03574378 |  |  |
| C06053F09 | -2.04645499 |  |  |
| IC0AAA97AG12 | -2.05922997 |  |  |
| IC0AAA78AF05 | -2.06266845 |  |  |
| C07006D07 | -2.06302649 |  |  |
| KN0AAA1AF04 | -2.06789927 |  |  |
| C01008H04 | -2.07149372 |  |  |
| C18007F05 | -2.08567038 |  |  |
| C31010D03 | -2.09021032 |  |  |
| C04004H06 | -2.10051926 |  |  |
| C08024G07 | -2.10243955 |  |  |
| KN0AAQ8YL15 | -2.1032158 |  |  |
| KN0AAP2YL01 | -2.10606564 |  |  |
| C07011A07 | -2.10845462 |  |  |
| C08015H08 | -2.10906819 |  |  |
| C5(cal5) | -2.10915018 |  |  |
| C08007E05 | -2.11327867 |  |  |
| C02002F03 | -2.11349738 |  |  |
| C04004H06 | -2.1199037 |  |  |
| IC0AAA87CA06 | -2.12289553 |  |  |
| C04004H06 | -2.12937141 |  |  |
| C02027H10 | -2.12949039 |  |  |
| C01014F10 | -2.13192557 |  |  |
| C18025E03 | -2.13871347 |  |  |
| KN0AAL1AD12 | -2.14975172 |  |  |
| KN0AAB1AF06 | -2.15292342 |  |  |
| C34203H09 | -2.16513775 |  |  |
| C18020E09 | -2.18966689 |  |  |
| IC0AAA61DB01 | -2.19246172 |  |  |
| IC0AAA52DB04 | -2.19766265 |  |  |
| KN0AAP6YP01 | -2.20361286 |  |  |
| C08021D12 | -2.21773257 |  |  |
| C34002G02 | -2.22142714 |  |  |
| KN0AAQ13YH04 | -2.22160736 |  |  |
| C32009A07 | -2.22562347 |  |  |
| C08024D10 | -2.23203912 |  |  |
| C04004H06 | -2.25674663 |  |  |
| C31405H04 | -2.25772809 |  |  |
| C31802C04 | -2.26603962 |  |  |
| C31405A03 | -2.27299524 |  |  |
| C31603D03 | -2.28666861 |  |  |
| KN0AAB1CC01 | -2.28854651 |  |  |
| C05076B12 | -2.28949092 |  |  |
| IC0AAA43DC08 | -2.2965053 |  |  |
| IC0AAA6DC07 | -2.30836813 |  |  |
| IC0AAA94BG07 | -2.31331229 |  |  |
| C08005D02 | -2.31688827 |  |  |
| C31604E09 | -2.31792524 |  |  |
| C16012H12 | -2.33115644 |  |  |
| C32009D10 | -2.33464304 |  |  |
| C31204A01 | -2.34584339 |  |  |
| C08022C07 | -2.3562965 |  |  |
| KN0AAQ8YM13 | -2.36706708 |  |  |
| C08009F11 | -2.39589478 |  |  |
| C31706A02 | -2.39655094 |  |  |
| C01001C06 | -2.41085121 |  |  |
| IC0AAA49BC07 | -2.42861267 |  |  |
| C32011E02 | -2.44024507 |  |  |
| KN0AAA1DB01 | -2.46316144 |  |  |
| C08036C12 | -2.46875922 |  |  |
| C08021C03 | -2.48182735 |  |  |
| C05054C10 | -2.48262676 |  |  |
| C31604E05 | -2.4858623 |  |  |
| IC0AAA70CE07 | -2.49370322 |  |  |
| IC0AAA70AC04 | -2.51528467 |  |  |
| C18015D05 | -2.51542515 |  |  |
| C34206F02 | -2.5777549 |  |  |
| C31703E12 | -2.58066487 |  |  |
| C05068G03 | -2.61283416 |  |  |
| IC0AAA19CC10 | -2.62025439 |  |  |
| C01002B06 | -2.62237571 |  |  |
| C05057G09 | -2.73535835 |  |  |
| C31804D06 | -2.75738746 |  |  |
| IC0AAA84BD03 | -2.76086106 |  |  |
| IC0AAA17AG12 | -2.76420765 |  |  |
| C05057H10 | -2.77555447 |  |  |
| C05069E05 | -2.79049665 |  |  |
| IC0AAA8DA12 | -2.79447081 |  |  |
| IC0AAA8DD04 | -2.80312028 |  |  |
| C31304G05 | -2.81386214 |  |  |
| C06051D11 | -2.87086191 |  |  |
| C03006F07 | -2.89286962 |  |  |
| C31010E02 | -2.90633562 |  |  |
| IC0AAA26CD08 | -2.92235106 |  |  |
| KN0AAQ7YP09 | -2.97252845 |  |  |
| C02012D08 | -2.98940556 |  |  |
| C06051B02 | -3.02792109 |  |  |
| C31504C12 | -3.16978284 |  |  |
| C31704G03 | -3.17394943 |  |  |
| IC0AAA90AH08 | -3.25317572 |  |  |
| C31302A07 | -3.28022963 |  |  |
| KN0AAA2BG05 | -3.29029059 |  |  |
| C34109B02 | -3.33654657 |  |  |
| IC0AAA16AG08 | -3.3840093 |  |  |
| C05057A11 | -3.41773626 |  |  |
| C32010C03 | -3.477882 |  |  |
| C34100H01 | -3.49014425 |  |  |
| C01009A10 | -3.49355414 |  |  |
| C01015D11 | -3.5010849 |  |  |
| C02023C12 | -3.53614808 |  |  |
| IC0AAA41DA02 | -3.53998978 |  |  |
| IC0AAA5AE04 | -3.66184574 |  |  |
| C31707H07 | -3.66732232 |  |  |
| C01017B04 | -3.70267555 |  |  |
| IC0AAA18DF09 | -3.72594753 |  |  |
| C08013A10 | -3.76411791 |  |  |
| C32013G09 | -3.78236282 |  |  |
| IC0AAA20AC04 | -3.90773309 |  |  |
| C08035B09 | -4.02475273 |  |  |
| C01009F12 | -4.15476645 |  |  |
| KN0AAA2BF04 | -4.15616417 |  |  |
| C31708B05 | -4.15906694 |  |  |
| KN0AAP7YO06 | -4.21087755 |  |  |
| C05065C12 | -4.34467212 |  |  |
| C31305G10 | -4.46580304 |  |  |
| C32001C06 | -4.55305556 |  |  |
| C32009D05 | -4.61236022 |  |  |
| C07003E04 | -4.77688864 |  |  |
| C32006E04 | -5.09472033 |  |  |
| C06005A10 | -5.17000705 |  |  |
| IC0AAA35DG06 | -5.20327927 |  |  |
| KN0AAK3BE10 | -5.99469069 |  |  |
| C08004G06 | -6.27731152 |  |  |
| C08013G01 | -6.64368514 |  |  |
| TOTAL | 896 | - | 342 |
