# Supplementary material for: Different adaptation strategies of two citrus scion/rootstock combinations in response to drought stress
Source: PLoS One. 2017 May 17;12(5):e0177993. doi: 10.1371/journal.pone.0177993 (PMC5435350; doi:10.1371/journal.pone.0177993)
Supplement: S2 Table — B: bottleneck; C: common; HB: hub-bottleneck. (DOCX) [file pone.0177993.s002.docx]

**S2 Table.** Centrality of the proteins from the global network. B: bottleneck; C: common; HB: hub-bottleneck.

| Gene ID | Network | Microarray | Betweenness value | Degree value | Centralidade |
| --- | --- | --- | --- | --- | --- |
| LUT2 | 4x | - | 0.0 | 3.0 | C |
| CRTISO | 4x | - | 0.0 | 3.0 | C |
| LYC | 4x | - | 14156.333333333332 | 9.0 | B |
| L73G19.80 | 4x | - | 10574.000000001352 | 6.0 | C |
| LUT1 | 2x_4x | - | 1179.145169617838 | 98.0 | HB |
| F6N7.5 | 4x | - | 10574.000000001352 | 6.0 | C |
| NPQ1 | 4x | - | 0.3333333333333333 | 4.0 | C |
| ABA1 | 4x | - | 67059.74844143739 | 13.0 | B |
| AT1G77670 | 4x | - | 0.0 | 1.0 | C |
| CYP97A3 | 4x | - | 10574.333333334685 | 7.0 | C |
| AT5G57300 | 4x | - | 0.0 | 1.0 | C |
| ZDS | 4x | - | 2.0 | 4.0 | C |
| D27 | 4x | - | 3544.333333333333 | 5.0 | C |
| PSB27 | 4x | - | 0.0 | 2.0 | C |
| AT5G36130 | 2x_4x | - | 1179.145169617838 | 98.0 | H |
| AT1G78140 | 4x | - | 0.0 | 1.0 | C |
| CCD7 | 4x | - | 0.0 | 1.0 | C |
| AT2G41040 | 4x | - | 0.0 | 1.0 | C |
| NAPRT1 | 2x_4x | 2x | 11864.362616216682 | 3.0 | B |
| NAPRT2 | 2x_4x | - | 3674.692102674686 | 4.0 | C |
| RAD50 | 2x_4x | - | 2104.683622889817 | 10.0 | B |
| MRE11 | 2x_4x | - | 326.09876109337495 | 8.0 | HB |
| AT2G33840 | 4x | - | 201.20177997705858 | 23.0 | C |
| KU80 | 4x | - | 3519.351721961753 | 51.0 | H |
| KU70 | 4x | - | 17446.055570130065 | 91.0 | HB |
| AT1G28350 | 4x | - | 201.20177997705858 | 23.0 | C |
| MSH2 | 2x_4x | - | 4042.2598950136103 | 35.0 | HB |
| TERT | 4x | - | 105.5217504383505 | 12.0 | C |
| MSH5 | 2x_4x | - | 1606.7196083063284 | 34.0 | HB |
| AT1G29630 | 4x | - | 59.79748208879081 | 14.0 | C |
| AT3G21300 | 4x | - | 253.6458377675272 | 6.0 | C |
| MNS3 | 4x | - | 0.5479384003974168 | 2.0 | C |
| SAUL1 | 2x_4x | - | 65.20198990801754 | 33.0 | C |
| LIG6 | 2x_4x | - | 1188.7514927230804 | 24.0 | HB |
| PUB38 | 4x | - | 0.0 | 1.0 | C |
| RK3 | 4x | 4x | 18305.039984107076 | 10.0 | B |
| AT2G02550 | 4x | - | 59.79748208879081 | 14.0 | C |
| AT3G14890 | 4x | - | 27.66318379537292 | 11.0 | C |
| PUP7 | 4x | - | 0.0 | 1.0 | C |
| APTx | 4x | - | 1.3071517770161643 | 6.0 | C |
| ATM | 2x_4x | - | 4375.951153210922 | 36.0 | C |
| MLH1 | 4x | - | 0.4285714285714285 | 8.0 | C |
| WRNExO | 4x | - | 0.0 | 4.0 | C |
| ATLIG4 | 4x | - | 12.756842402792682 | 9.0 | C |
| PMS1 | 4x | - | 0.4285714285714285 | 8.0 | C |
| PUB14 | 2x_4x | - | 65.20198990801754 | 33.0 | C |
| AT1G18090 | 4x | - | 59.79748208879081 | 14.0 | C |
| AT2G32170 | 4x | 4x | 0.0 | 1.0 | C |
| PUB29 | 4x | - | 0.0 | 1.0 | C |
| PUB9 | 2x_4x | - | 65.20198990801754 | 33.0 | C |
| WRKY7 | 4x | 2x | 3544.0 | 2.0 | C |
| CAM5 | 4x | - | 0.0 | 1.0 | C |
| PUB13 | 4x | - | 0.0 | 1.0 | C |
| PUB45 | 4x | - | 0.0 | 1.0 | C |
| fls2 | 4x | - | 4541.3684247079045 | 10.0 | C |
| xBAT31 | 4x | 4x | 912.9691001502499 | 37.0 | C |
| CHR1 | 4x | - | 357.316139427482 | 60.0 | H |
| HTA12 | 4x | - | 842.0407625354727 | 63.0 | H |
| RAD54 | 4x | - | 9.644963706600459 | 7.0 | C |
| VIP5 | 4x | - | 1795.692618886604 | 58.0 | H |
| PPAN | 4x | - | 79.24048627550086 | 23.0 | C |
| VIP4 | 4x | - | 308.2777530446998 | 32.0 | C |
| AT3G09480 | 4x | - | 336.73169782835083 | 67.0 | H |
| HTA10 | 2x_4x | - | 205.49265676416454 | 12.0 | H |
| CKA2 | 4x | - | 1674.4929527114327 | 40.0 | C |
| AT3G30430 | 2x_4x | - | 137.5002104235908 | 35.0 | C |
| MCM4 | 4x | - | 1584.6458762844793 | 37.0 | C |
| CKB1 | 4x | - | 64.72233703188628 | 27.0 | C |
| SPT16 | 4x | - | 31159.146964265477 | 138.0 | HB |
| AT5G02570 | 4x | - | 336.73169782835083 | 67.0 | H |
| GAMMA-H2Ax | 2x_4x | - | 103.73862536909861 | 11.0 | H |
| HTA13 | 2x_4x | - | 184.46856697312603 | 15.0 | H |
| AT5G59690.1 | 4x | - | 255.31544131841358 | 60.0 | H |
| AT2G35280 | 2x_4x | - | 137.5002104235908 | 35.0 | C |
| CHR4 | 4x | - | 432.32159891221124 | 53.0 | H |
| AT3G45930.1 | 4x | - | 255.31544131841358 | 60.0 | H |
| HTA4 | 4x | - | 3087.9167536490436 | 73.0 | H |
| HTA7 | 4x | - | 842.0407625354727 | 63.0 | H |
| AT1G07660 | 2x_4x | - | 2729.621322598984 | 15.0 | H |
| BSK8 | 2x_4x | - | 0.9166666666666666 | 4.0 | C |
| BSK1 | 2x_4x | - | 3369.8993657324963 | 4.0 | C |
| AT1G05910 | 4x | - | 543.784608930672 | 45.0 | C |
| AT5G59970.1 | 4x | - | 255.31544131841358 | 61.0 | H |
| HTA9 | 4x | - | 3087.9167536490436 | 73.0 | H |
| AT3G46320.1 | 4x | - | 255.31544131841358 | 60.0 | H |
| HTB2 | 2x_4x | - | 1886.8528494896698 | 12.0 | H |
| LEC1 | 4x | - | 5.29344612719906 | 7.0 | C |
| ASG3 | 4x | - | 357.316139427482 | 60.0 | H |
| AT3G47110 | 4x | - | 123.07534727187733 | 6.0 | C |
| BAK1 | 2x_4x | - | 107.3431017667994 | 9.0 | C |
| SERK1 | 2x_4x | - | 0.0 | 3.0 | C |
| CHR11 | 4x | - | 357.316139427482 | 60.0 | H |
| KAPP | 2x_4x | - | 310.4329537008398 | 5.0 | C |
| EMS1 | 4x | - | 123.07534727187733 | 6.0 | C |
| HTA8 | 2x_4x | - | 3096.077580451517 | 19.0 | HB |
| AT3G16600 | 4x | - | 2074.3709865763285 | 41.0 | C |
| AT2G37470 | 4x | - | 336.73169782835083 | 67.0 | H |
| AT1G74875 | 2x_4x | - | 137.5002104235908 | 35.0 | C |
| CHR18 | 2x_4x | - | 10.610907426369259 | 27.0 | C |
| ERL1 | 4x | - | 123.07534727187733 | 7.0 | C |
| AT2G04740 | 4x | - | 1659.106299612223 | 39.0 | C |
| RPK2 | 4x | - | 123.07534727187733 | 6.0 | C |
| HTA11 | 2x_4x | - | 1868.676212699223 | 16.0 | HB |
| HTB11 | 4x | - | 336.73169782835083 | 67.0 | H |
| AT2G28720 | 4x | - | 336.73169782835083 | 67.0 | H |
| HTB9 | 4x | - | 336.73169782835083 | 67.0 | H |
| GTA2 | 4x | - | 1107.1526817415383 | 42.0 | C |
| AT3G54460 | 4x | - | 2074.3709865763285 | 41.0 | C |
| TOPII | 2x_4x | - | 693.8756112833408 | 17.0 | HB |
| RAT5 | 4x | - | 2777.2156777003074 | 64.0 | H |
| AT1G08170 | 4x | - | 336.73169782835083 | 67.0 | H |
| AT1G07660.1 | 4x | - | 240.67675753376608 | 59.0 | H |
| ASHH1 | 4x | - | 2211.414157342055 | 42.0 | C |
| AT1G63210 | 4x | - | 7039.501445107221 | 64.0 | HB |
| HTB1 | 2x_4x | - | 37.99204470804929 | 8.0 | H |
| CHR17 | 4x | - | 357.316139427482 | 60.0 | H |
| RPA70C | 2x_4x | - | 323.2042620805563 | 37.0 | C |
| AT5G07810 | 2x_4x | - | 10.610907426369259 | 27.0 | C |
| AT1G07820.1 | 4x | - | 240.67675753376608 | 59.0 | H |
| CSN5B | 2x_4x | - | 766.905486960596 | 6.0 | C |
| CKB4 | 4x | - | 64.72233703188628 | 26.0 | C |
| AT2G23070 | 4x | - | 1674.4929527114327 | 40.0 | C |
| SYD | 4x | - | 5514.936942976159 | 49.0 | B |
| BRM | 4x | - | 86.23318606044373 | 10.0 | C |
| NF-YB2 | 4x | - | 5.29344612719906 | 7.0 | C |
| HTB4 | 4x | - | 336.73169782835083 | 67.0 | H |
| GTC2 | 4x | - | 31159.146964265477 | 138.0 | HB |
| ELF8 | 4x | - | 1129.1877947913244 | 32.0 | C |
| AT1G11100 | 4x | - | 2074.3709865763285 | 41.0 | C |
| CKA3 | 4x | - | 1674.4929527114327 | 40.0 | C |
| CAND1 | 4x | - | 0.30722812005950917 | 5.0 | C |
| GTB1 | 4x | - | 7039.501445107221 | 64.0 | HB |
| At3g22480 | 4x | - | 526.6064894767426 | 8.0 | C |
| PFD1 | 4x | - | 3544.0 | 5.0 | C |
| AT2G40860 | 4x | - | 1712.6465471368895 | 39.0 | C |
| AT3G53650 | 4x | - | 336.73169782835083 | 67.0 | H |
| PKR2 | 4x | - | 306.13495534166213 | 50.0 | C |
| RPA70B | 2x_4x | - | 339.3590541796589 | 43.0 | H |
| BKI1 | 2x_4x | - | 0.0 | 2.0 | C |
| AT3G15120 | 4x | 2x | 543.784608930672 | 45.0 | C |
| AT5G41560 | 4x | - | 1.635799548630938 | 7.0 | C |
| DCAF1 | 4x | - | 53.269587013898025 | 14.0 | C |
| PHP | 4x | - | 5680.624586240349 | 64.0 | HB |
| CKB3 | 4x | - | 64.72233703188628 | 27.0 | C |
| CHR34 | 4x | - | 9.644963706600459 | 7.0 | C |
| SLD5 | 4x | - | 0.3333333333333333 | 6.0 | C |
| HIRA | 4x | - | 754.9475167118391 | 52.0 | H |
| CHR5 | 4x | - | 9.644963706600459 | 7.0 | C |
| AT3G17380 | 4x | 4x | 0.23579954863093777 | 3.0 | C |
| AT2G34210 | 4x | - | 1107.1526817415383 | 42.0 | C |
| CKA1 | 4x | - | 1674.4929527114327 | 40.0 | C |
| KTF1 | 4x | - | 1107.1526817415383 | 42.0 | C |
| ELF7 | 4x | - | 1098.7653870787206 | 41.0 | C |
| HTA2 | 2x_4x | - | 6.59809418865003 | 4.0 | H |
| NF-YB4 | 4x | - | 5.543446127199059 | 9.0 | C |
| NF-YB8 | 4x | - | 5.29344612719906 | 7.0 | C |
| HIS4 | 2x_4x | - | 12391.334004644785 | 17.0 | HB |
| COP1 | 4x | - | 9491.898365293904 | 70.0 | H |
| DWA1 | 4x | - | 199.41925653319203 | 11.0 | C |
| CHR40 | 4x | - | 9.644963706600459 | 7.0 | C |
| PRL1 | 2x_4x | - | 327.41229543875477 | 86.0 | HB |
| AT4G36180 | 4x | - | 123.07534727187733 | 6.0 | C |
| AT1G52950 | 2x_4x | - | 137.5002104235908 | 35.0 | C |
| AT3G28040 | 4x | - | 123.07534727187733 | 6.0 | C |
| NF-YB3 | 4x | - | 5.29344612719906 | 7.0 | C |
| MCM5 | 2x_4x | - | 2301.448341317296 | 41.0 | HB |
| GSO2 | 4x | - | 123.07534727187733 | 6.0 | C |
| CSN5A | 2x_4x | - | 301.04943840019746 | 5.0 | C |
| AT3G47570 | 4x | - | 123.07534727187733 | 6.0 | C |
| DET1 | 4x | - | 19.297921049530533 | 6.0 | C |
| H2AxA | 4x | - | 842.0407625354727 | 63.0 | H |
| AT5G63930 | 4x | - | 123.07534727187733 | 6.0 | C |
| CKB2 | 4x | - | 64.72233703188628 | 26.0 | C |
| HTA6 | 2x_4x | - | 4229.195121208235 | 14.0 | H |
| AT3G32280 | 4x | - | 9.644963706600459 | 7.0 | C |
| IMK2 | 2x_4x | 2x | 0.0 | 1.0 | C |
| CHR42 | 4x | - | 9.644963706600459 | 7.0 | C |
| CHR38 | 4x | - | 9.644963706600459 | 7.0 | C |
| AT2G40770 | 4x | - | 9.791305170015093 | 10.0 | C |
| CPL1 | 4x | - | 27.21645014784414 | 4.0 | C |
| GSO1 | 4x | - | 123.07534727187733 | 6.0 | C |
| AT3G31900 | 4x | - | 9.644963706600459 | 7.0 | C |
| EFS | 4x | - | 2019.2166769746686 | 41.0 | C |
| PFD3 | 4x | - | 7439.06786256407 | 9.0 | C |
| AIP3 | 4x | - | 663.6924091264442 | 10.0 | C |
| EFR | 4x | - | 812.4412645772919 | 7.0 | C |
| AT1G75640 | 4x | - | 123.07534727187733 | 6.0 | C |
| AT5G49770 | 4x | - | 123.07534727187733 | 6.0 | C |
| NF-YB10 | 4x | - | 5.29344612719906 | 7.0 | C |
| ATRx | 4x | - | 37.59367332561381 | 8.0 | C |
| PSY1R | 4x | - | 123.07534727187733 | 6.0 | C |
| BRI1 | 2x_4x | 2x | 38617.86659735915 | 19.0 | B |
| RPA1A | 2x_4x | - | 210.65310674004957 | 36.0 | C |
| AT3G32100 | 2x_4x | - | 137.5002104235908 | 35.0 | C |
| AT1G74360 | 4x | - | 123.07534727187733 | 6.0 | C |
| ICU2 | 2x_4x | - | 30998.518449967967 | 88.0 | HB |
| AT1G36030 | 2x_4x | - | 137.5002104235908 | 35.0 | C |
| chr31 | 4x | - | 9.644963706600459 | 7.0 | C |
| RPA70D | 2x_4x | - | 2642.9428186972737 | 51.0 | H |
| DWA2 | 4x | - | 0.2282706093189964 | 9.0 | C |
| NF-YB6 | 4x | - | 5.29344612719906 | 7.0 | C |
| AT1G36510 | 2x_4x | - | 137.5002104235908 | 35.0 | C |
| AT3G49670.1 | 4x | - | 123.07534727187733 | 6.0 | C |
| DRD1 | 4x | - | 17.90951490853461 | 8.0 | C |
| AT5G25930 | 4x | - | 123.07534727187733 | 6.0 | C |
| AT4G28650 | 4x | - | 123.07534727187733 | 6.0 | C |
| AT3G32260 | 2x_4x | - | 137.5002104235908 | 35.0 | C |
| AT3G47090 | 4x | - | 123.07534727187733 | 6.0 | C |
| MOM | 4x | - | 9.644963706600459 | 7.0 | C |
| BRL3 | 4x | - | 2805.051806255475 | 9.0 | C |
| NF-YB7 | 4x | - | 5.29344612719906 | 8.0 | C |
| ERL2 | 4x | - | 123.07534727187733 | 6.0 | C |
| PEPR1 | 4x | - | 1621.4608214062225 | 8.0 | C |
| HSL2 | 4x | - | 123.07534727187733 | 6.0 | C |
| COP8 | 4x | - | 0.23579954863093777 | 7.0 | C |
| PSKR2 | 4x | - | 123.07534727187733 | 6.0 | C |
| AT1G05120 | 4x | - | 9.644963706600459 | 7.0 | C |
| ERECTA | 4x | 2x | 123.07534727187733 | 7.0 | C |
| PFD5 | 2x_4x | - | 37.007968143218825 | 6.0 | B |
| BIK1 | 2x_4x | - | 755.571484953289 | 6.0 | C |
| DDB2 | 4x | - | 43.77845412988509 | 11.0 | C |
| ROC1 | 4x | - | 0.0 | 1.0 | C |
| AT2G24130 | 4x | - | 123.07534727187733 | 6.0 | C |
| AT3G47580 | 4x | - | 123.07534727187733 | 6.0 | C |
| BSK3 | 2x_4x | - | 32.95871770241671 | 4.0 | C |
| AT1G21780 | 4x | - | 0.23579954863093777 | 3.0 | C |
| AT1G67623 | 2x_4x | - | 137.5002104235908 | 35.0 | C |
| NF-YB5 | 4x | - | 5.29344612719906 | 8.0 | C |
| AT5G37450 | 4x | - | 123.07534727187733 | 6.0 | C |
| CPL2 | 4x | - | 27.21645014784414 | 4.0 | C |
| AT3G32330 | 4x | - | 9.644963706600459 | 7.0 | C |
| AT5G65700.1 | 4x | - | 123.07534727187733 | 6.0 | C |
| AT2G05642 | 2x_4x | - | 2670.020269574619 | 36.0 | C |
| AT4G19130 | 2x_4x | - | 137.5002104235908 | 35.0 | C |
| BRL2 | 4x | - | 55.90748204483625 | 3.0 | C |
| NF-YB1 | 4x | - | 5.29344612719906 | 7.0 | C |
| CxE18 | 4x | 4x | 0.0 | 1.0 | C |
| SEC10 | 2x_4x | - | 0.0 | 3.0 | B |
| ExO70D1 | 4x | - | 15.559277923380376 | 23.0 | C |
| AT4G17830 | 4x | - | 0.5 | 4.0 | C |
| OTC | 4x | - | 2499.608031272042 | 7.0 | C |
| ExO70D3 | 4x | - | 15.559277923380376 | 23.0 | C |
| AT1G10385 | 4x | - | 15430.17547368623 | 67.0 | HB |
| CASP | 4x | - | 133058.82906116333 | 64.0 | HB |
| SQN | 4x | - | 0.035954973532221296 | 5.0 | C |
| AT4G17560 | 2x_4x | - | 554.8151185009241 | 22.0 | B |
| SEC1B | 4x | - | 306.47061174221324 | 53.0 | H |
| SYP112 | 4x | - | 3.6215746186947815 | 8.0 | C |
| SEC5A | 4x | - | 55848.404778063545 | 66.0 | HB |
| NTMC2T2.1 | 4x | - | 0.17740648981811588 | 7.0 | C |
| ExO70H2 | 4x | - | 15.559277923380376 | 23.0 | C |
| SEC5B | 4x | - | 52304.4047780649 | 65.0 | HB |
| eIF6A | 2x_4x | - | 16.36956476394711 | 9.0 | C |
| AT1G79150 | 4x | - | 88.17570967546841 | 16.0 | C |
| VPS9B | 4x | - | 17981.06307450406 | 31.0 | B |
| ExO70H4 | 4x | - | 15.559277923380376 | 23.0 | C |
| ExO70E1 | 4x | - | 15.559277923380376 | 23.0 | C |
| AT1G77030 | 4x | - | 278.9413937181149 | 19.0 | C |
| ExO70H5 | 4x | - | 15.559277923380376 | 23.0 | C |
| KEU | 2x_4x | - | 0.0 | 1.0 | HB |
| AT5G47190 | 2x_4x | - | 781.8332710724416 | 23.0 | B |
| AT3G08980 | 4x | - | 4.666666666666666 | 13.0 | C |
| AT5G06360 | 2x_4x | - | 0.0 | 1.0 | C |
| EMB3105 | 2x_4x | - | 2318.74183870341 | 31.0 | HB |
| SEC15A | 4x | - | 119.5807611607124 | 44.0 | C |
| SEC6 | 4x | - | 34.351978760721025 | 44.0 | C |
| RABE1c | 4x | - | 62.722729424485394 | 40.0 | C |
| SEC3B | 4x | - | 9799.868274082392 | 76.0 | HB |
| CAK1AT | 4x | - | 11.398838052238814 | 8.0 | C |
| CDKB2;1 | 2x_4x | - | 9015.409178335463 | 64.0 | HB |
| ExO84B | 4x | - | 15430.17547368623 | 67.0 | HB |
| CML11 | 4x | 4x | 1190.6292059260093 | 3.0 | C |
| RABE1e | 4x | - | 62.722729424485394 | 40.0 | C |
| SEC3A | 4x | - | 9799.868274082392 | 76.0 | HB |
| CAM3 | 4x | - | 10.629205925999996 | 2.0 | C |
| AT3G22660 | 4x | - | 141.716954081766 | 17.0 | C |
| CAM6 | 4x | - | 10.629205925999996 | 2.0 | C |
| ExO70B1 | 4x | - | 15.559277923380376 | 23.0 | C |
| ExO70E2 | 4x | - | 15.559277923380376 | 24.0 | C |
| RAB8 | 4x | - | 62.722729424485394 | 40.0 | C |
| AT3G15710 | 4x | - | 3358.4205050749174 | 16.0 | C |
| AT2G25100 | 4x | - | 276.59569495439627 | 16.0 | C |
| RAB1C | 4x | - | 5816.3478409074505 | 12.0 | C |
| STN7 | 4x | - | 0.08955223880597014 | 3.0 | C |
| AT1G10180 | 4x | - | 15430.17547368623 | 67.0 | HB |
| AT2G47090 | 4x | - | 0.09749343507068287 | 6.0 | C |
| Plsp2A | 4x | - | 1.487179487179487 | 9.0 | C |
| TON1B | 4x | - | 0.0 | 2.0 | C |
| AT1G24240 | 4x | - | 3241.9335372448836 | 15.0 | C |
| NTMC2T6.2 | 4x | - | 0.17740648981811588 | 7.0 | C |
| AT5G59840 | 4x | - | 62.722729424485394 | 40.0 | C |
| ExO70A1 | 4x | - | 15.559277923380376 | 23.0 | C |
| CAM2 | 4x | - | 7094.629205926018 | 3.0 | B |
| PLSP1 | 4x | - | 1.487179487179487 | 9.0 | C |
| AT3G60950 | 4x | - | 4912.128438507323 | 13.0 | C |
| ExO70H1 | 4x | - | 15.559277923380376 | 24.0 | C |
| AT3G53590 | 4x | - | 62.722729424485394 | 40.0 | C |
| SEC15B | 4x | - | 119.5807611607124 | 44.0 | C |
| CDKB1;1 | 4x | - | 11.398838052238814 | 8.0 | C |
| ExO70H6 | 4x | - | 15.559277923380376 | 23.0 | C |
| AT5G04600 | 2x_4x | - | 6083.113406749128 | 48.0 | HB |
| SEC8 | 4x | - | 1678.2194788823178 | 43.0 | C |
| AT4G11630 | 4x | - | 3241.9335372448836 | 15.0 | C |
| SYTB | 4x | - | 0.17740648981811588 | 7.0 | C |
| RAB1A | 4x | - | 5816.3478409074505 | 12.0 | C |
| ExO70H7 | 4x | - | 15.559277923380376 | 23.0 | C |
| ExO70H8 | 4x | - | 15.559277923380376 | 23.0 | C |
| RAB8C | 4x | - | 62.722729424485394 | 40.0 | C |
| ExO70B2 | 4x | - | 15.559277923380376 | 23.0 | C |
| SYP124 | 4x | - | 3.6215746186947815 | 8.0 | C |
| SEC1A | 2x_4x | - | 0.0 | 1.0 | HB |
| AT2G27480 | 4x | - | 0.0 | 1.0 | C |
| TPP | 4x | - | 1.487179487179487 | 9.0 | C |
| ADC2 | 4x | - | 0.0 | 3.0 | C |
| ExO70A3 | 4x | - | 15.559277923380376 | 23.0 | C |
| PDE318 | 2x_4x | - | 0.0 | 1.0 | C |
| ExO70D2 | 4x | - | 15.559277923380376 | 23.0 | C |
| AT2G21010 | 4x | - | 0.17740648981811588 | 7.0 | C |
| SYP131 | 4x | - | 3.6215746186947815 | 8.0 | C |
| MEMB11 | 4x | - | 81.75135923979741 | 17.0 | C |
| ExO70A2 | 4x | - | 15.559277923380376 | 23.0 | C |
| VPS9A | 4x | - | 17981.06307450406 | 31.0 | B |
| AT3G01830 | 2x_4x | - | 25.23723015140212 | 21.0 | C |
| MEMB12 | 4x | - | 81.75135923979741 | 17.0 | C |
| AT3G62240 | 4x | - | 0.09749343507068287 | 6.0 | C |
| ExO70H3 | 4x | - | 15.559277923380376 | 23.0 | C |
| AT4 | 4x | - | 15.559277923380376 | 23.0 | C |
| AT2G41410 | 4x | - | 10.629205925999996 | 2.0 | C |
| AT3G49000 | 2x_4x | - | 6779.926077074136 | 11.0 | C |
| RPC14 | 2x_4x | - | 4191.719233416162 | 33.0 | B |
| AT1G50920 | 4x | - | 2786.232151695084 | 21.0 | C |
| MSS3 | 4x | 2x | 10.629205925999996 | 2.0 | C |
| ExO70G2 | 4x | - | 15.559277923380376 | 23.0 | C |
| AT1G06200 | 4x | - | 4.666666666666666 | 13.0 | C |
| AGD11 | 4x | - | 10.629205925999996 | 2.0 | C |
| SYTA | 4x | 2x | 0.17740648981811588 | 7.0 | C |
| AT1G18530 | 4x | - | 10.629205925999996 | 2.0 | C |
| AT4G25730 | 4x | - | 754.9779251394082 | 12.0 | C |
| AT3G61030 | 4x | - | 4912.128438507323 | 13.0 | C |
| AT1G24620 | 4x | - | 10.629205925999996 | 2.0 | C |
| PRF4 | 4x | - | 0.02631578947368421 | 2.0 | C |
| eIF6B | 2x_4x | - | 56.667461734870734 | 20.0 | C |
| AT1G10300 | 2x_4x | - | 0.0 | 1.0 | C |
| RA-5 | 4x | - | 5816.3478409074505 | 12.0 | C |
| SYP111 | 2x_4x | - | 63787.09889498 | 17.0 | B |
| CAM4 | 2x_4x | - | 11324.099297348159 | 5.0 | B |
| ExO70G1 | 4x | - | 15.559277923380376 | 23.0 | C |
| ARGAH1 | 4x | - | 99.9255101117282 | 6.0 | C |
| ADC1 | 4x | - | 0.0 | 3.0 | C |
| CAM7 | 4x | - | 10.629205925999996 | 2.0 | C |
| NTMC2T6.1 | 4x | - | 0.17740648981811588 | 7.0 | C |
| AT1G29960 | 4x | - | 1.487179487179487 | 9.0 | C |
| AT1G53530 | 4x | - | 1.487179487179487 | 9.0 | C |
| ATSYTF | 4x | - | 0.17740648981811588 | 7.0 | C |
| SYP122 | 4x | - | 3.6215746186947815 | 8.0 | C |
| AT3G03400 | 4x | - | 10.629205925999996 | 2.0 | C |
| CDKB2;2 | 4x | - | 0.08955223880597014 | 6.0 | C |
| AT5G13240 | 4x | 2x | 0.28 | 8.0 | C |
| AT2G37990 | 4x | - | 85.72756069692356 | 13.0 | C |
| AT1G52600 | 4x | - | 3358.4205050749174 | 16.0 | C |
| CML42 | 4x | - | 10.629205925999996 | 2.0 | C |
| ExO70F1 | 4x | - | 15.559277923380376 | 23.0 | C |
| ExO70C1 | 4x | - | 15.559277923380376 | 23.0 | C |
| ExO70C2 | 4x | - | 15.559277923380376 | 23.0 | C |
| AT3G23860 | 2x_4x | - | 5654.0 | 2.0 | C |
| SYP121 | 4x | - | 3.6215746186947815 | 8.0 | C |
| AT5G11750 | 4x | - | 3241.9335372448836 | 15.0 | C |
| AT1G23465 | 4x | - | 1.487179487179487 | 9.0 | C |
| AT3G28956 | 4x | - | 773.4853390045146 | 10.0 | C |
| AT5G62950 | 4x | - | 773.4853390045146 | 10.0 | C |
| NSF | 2x_4x | - | 105.51912298250357 | 61.0 | HB |
| AT5G23710 | 2x_4x | - | 484.9424181483579 | 9.0 | C |
| AT1G06790 | 2x_4x | - | 584.6481221301059 | 13.0 | C |
| NTMC2T5.2 | 4x | 4x | 0.17740648981811588 | 7.0 | C |
| AT5G13830 | 4x | - | 754.9779251394082 | 12.0 | C |
| STN8 | 4x | - | 0.08955223880597014 | 3.0 | C |
| AT5G04170 | 4x | - | 0.0 | 1.0 | C |
| AT5G01230 | 4x | - | 0.5714285714285714 | 8.0 | C |
| AT1G32250 | 4x | - | 10.629205925999996 | 2.0 | C |
| SYTD | 4x | - | 0.17740648981811588 | 7.0 | C |
| CAM9 | 4x | - | 1190.6292059260093 | 3.0 | C |
| SYTC | 4x | - | 0.17740648981811588 | 7.0 | C |
| CML30 | 4x | - | 10.629205925999996 | 2.0 | C |
| AT1G21550 | 4x | - | 10.629205925999996 | 2.0 | C |
| ATFP8 | 4x | - | 5816.3478409074505 | 12.0 | C |
| SYP132 | 4x | - | 3.6215746186947815 | 8.0 | C |
| SYP125 | 4x | - | 3.6215746186947815 | 8.0 | C |
| CML23 | 4x | - | 10.629205925999996 | 2.0 | C |
| AT3G03430 | 4x | - | 10.629205925999996 | 2.0 | C |
| AT5G49530 | 2x_4x | - | 1639.0908166040776 | 5.0 | C |
| PFN2 | 4x | - | 0.02631578947368421 | 2.0 | C |
| SYP123 | 4x | - | 3.6215746186947815 | 8.0 | C |
| AT2G41090 | 4x | - | 10.629205925999996 | 2.0 | C |
| AT1G18210 | 4x | - | 10.629205925999996 | 2.0 | C |
| PRF1 | 4x | - | 0.02631578947368421 | 2.0 | C |
| TON1A | 4x | - | 717.7899635664188 | 3.0 | C |
| CML38 | 4x | - | 10.629205925999996 | 2.0 | C |
| AT4G03290 | 4x | - | 10.629205925999996 | 2.0 | C |
| VPS54 | 4x | - | 0.1350067842605156 | 6.0 | C |
| AT4G26470 | 4x | - | 10.629205925999996 | 2.0 | C |
| NTMC2T5.1 | 4x | - | 0.17740648981811588 | 7.0 | C |
| AT3G59440 | 4x | - | 10.629205925999996 | 2.0 | C |
| AT3G10190 | 4x | 2x | 10.629205925999996 | 2.0 | C |
| AT1G73630 | 4x | - | 10.629205925999996 | 2.0 | C |
| AT3G24110 | 4x | - | 10.629205925999996 | 2.0 | C |
| AT3G47480 | 4x | - | 10.629205925999996 | 2.0 | C |
| CML43 | 4x | - | 10.629205925999996 | 2.0 | C |
| AT4G24830 | 2x_4x | - | 0.0 | 1.0 | C |
| NTMC2T4 | 4x | - | 0.17740648981811588 | 7.0 | C |
| AT2G36180 | 4x | - | 10.629205925999996 | 2.0 | C |
| AT1G76640 | 4x | - | 10.629205925999996 | 2.0 | C |
| PRF3 | 4x | - | 0.02631578947368421 | 2.0 | C |
| AT3G10300 | 4x | 2x | 0.0 | 1.0 | C |
| ORG1 | 2x_4x | 2x | 0.0 | 1.0 | C |
| AT5G17470 | 4x | - | 10.629205925999996 | 2.0 | C |
| AT1G12310 | 4x | - | 10.629205925999996 | 2.0 | C |
| AT3G03410 | 4x | - | 10.629205925999996 | 2.0 | C |
| APRR2 | 4x | - | 1.9999999999999998 | 3.0 | C |
| AT3G25600 | 4x | - | 10.629205925999996 | 2.0 | C |
| PC1 | 4x | - | 10.629205925999996 | 2.0 | C |
| AT2G21040 | 4x | - | 0.17740648981811588 | 7.0 | C |
| AT2G16710 | 2x_4x | - | 34.218791862240145 | 15.0 | C |
| UNE14 | 4x | - | 10.629205925999996 | 2.0 | C |
| CAM8 | 4x | - | 1190.6292059260093 | 3.0 | C |
| RHS2 | 4x | - | 10.629205925999996 | 2.0 | C |
| CML41 | 4x | - | 10.629205925999996 | 2.0 | C |
| AT3G03000 | 4x | - | 10.629205925999996 | 2.0 | C |
| PRF5 | 4x | - | 0.02631578947368421 | 2.0 | C |
| TCH2 | 4x | - | 10.629205925999996 | 2.0 | C |
| AT1G62820 | 4x | - | 10.629205925999996 | 2.0 | C |
| TCH3 | 4x | - | 10.629205925999996 | 2.0 | C |
| VHA-A | 2x_4x | - | 19187.980602457345 | 54.0 | HB |
| TUB4 | 4x | - | 86.5602336903805 | 36.0 | C |
| TPR10 | 2x_4x | - | 1699.1666906996759 | 24.0 | H |
| SAP130a | 2x_4x | - | 3441.7778077673397 | 96.0 | HB |
| VAB1 | 2x_4x | - | 6084.949280450183 | 43.0 | HB |
| DET3 | 2x_4x | - | 2180.9353280000096 | 42.0 | HB |
| AT3G11960 | 2x_4x | - | 3441.7778077673397 | 96.0 | HB |
| TTL3 | 2x_4x | 2x | 21617.049755213153 | 67.0 | HB |
| AT1G56500 | 4x | - | 19376.46427030187 | 91.0 | HB |
| TOC64-III | 2x_4x | - | 1338.3561406293188 | 60.0 | H |
| SCO1 | 2x_4x | - | 51208.66462645317 | 88.0 | HB |
| DGK1 | 4x | - | 3.6552702025449424 | 24.0 | C |
| CR88 | 2x_4x | - | 7158.988046099259 | 33.0 | HB |
| MAC3A | 2x_4x | - | 10284.641956068353 | 136.0 | HB |
| PANK2 | 4x | - | 49.26320336813481 | 35.0 | C |
| AT5G10370 | 2x_4x | - | 540.725263556452 | 46.0 | H |
| AT1G29310 | 4x | - | 500.150874030143 | 49.0 | C |
| MAC3B | 2x_4x | - | 12307.12252072089 | 138.0 | HB |
| LPLAT2 | 4x | 2x | 6.7161101988688685 | 31.0 | C |
| AT1G10580 | 2x_4x | - | 9586.49775918346 | 131.0 | HB |
| CA1 | 4x | 2x | 50.685367536589666 | 35.0 | C |
| AT5G28740 | 2x_4x | - | 3440.6678171246267 | 84.0 | HB |
| PIS1 | 2x_4x | - | 2802.8129114442563 | 15.0 | HB |
| AT2G45030 | 4x | - | 168.62870985146014 | 60.0 | H |
| AT1G63660 | 2x_4x | - | 1266.3260229275588 | 82.0 | H |
| TTL4 | 4x | - | 66.2508594697682 | 44.0 | C |
| AT1G57720 | 4x | - | 375.5248066351241 | 54.0 | H |
| PCNA2 | 2x_4x | - | 77157.42161913148 | 123.0 | HB |
| UBP27 | 4x | - | 50.2250535527633 | 37.0 | C |
| AT3G04480 | 4x | - | 844.2227932936019 | 37.0 | C |
| P5CS2 | 2x_4x | - | 3013.3198338295515 | 50.0 | H |
| AT2G01250 | 4x | - | 130.46445678263467 | 67.0 | H |
| MEE5 | 2x_4x | - | 2168.166279502903 | 83.0 | HB |
| SHD | 4x | - | 973.4341855015269 | 76.0 | H |
| HSP90.1 | 4x | - | 635.2175618968138 | 67.0 | H |
| HAP6 | 4x | - | 99.51659967003306 | 67.0 | H |
| AT4G01020 | 2x_4x | - | 540.725263556452 | 46.0 | H |
| P5CS1 | 2x_4x | 2x | 12987.18840350855 | 63.0 | HB |
| NIA1 | 2x_4x | - | 270.3460242779832 | 59.0 | H |
| AT5G25230 | 2x_4x | - | 11373.477311176019 | 86.0 | HB |
| CDS5 | 4x | - | 2339.2354983645755 | 61.0 | H |
| LPAT5 | 4x | - | 26.119667314844015 | 30.0 | C |
| DGK7 | 4x | - | 3.6552702025449424 | 24.0 | C |
| AT4G26770 | 4x | - | 4414.470935939054 | 76.0 | H |
| AT4G14250 | 2x_4x | - | 204.27344960262874 | 16.0 | H |
| AT4G16660 | 2x_4x | - | 24478.36741617948 | 65.0 | HB |
| TTL1 | 4x | - | 66.2508594697682 | 44.0 | C |
| AT5G10540 | 4x | - | 48.6812583253885 | 32.0 | C |
| AT1G09640 | 4x | - | 375.5248066351241 | 54.0 | H |
| DGK2 | 4x | - | 3.6552702025449424 | 24.0 | C |
| RCK | 2x_4x | - | 13858.782785989637 | 120.0 | HB |
| PIS2 | 2x_4x | - | 631.1788659103142 | 3.0 | B |
| AT1G79990 | 4x | - | 1868.711633735197 | 45.0 | C |
| SAP130b | 2x_4x | - | 3441.7778077673397 | 96.0 | HB |
| AT1G45332 | 4x | - | 168.62870985146014 | 60.0 | H |
| HSP81-3 | 4x | - | 635.2175618968138 | 67.0 | H |
| TOC64-V | 2x_4x | - | 1338.3561406293188 | 60.0 | H |
| DGL1 | 4x | - | 87.05092458640443 | 55.0 | H |
| AT5G61140 | 2x_4x | - | 13858.782785989637 | 120.0 | HB |
| KEG | 4x | - | 262.1005931360752 | 66.0 | H |
| NBR1 | 4x | - | 48.82513182058382 | 34.0 | C |
| AT5G13400 | 4x | 4x | 28620.732102147867 | 41.0 | B |
| KAK | 2x_4x | - | 32.598586271266676 | 38.0 | H |
| SDP1-LIKE | 2x_4x | - | 18525.56323957165 | 56.0 | HB |
| PAH1 | 4x | - | 27.081976259861964 | 27.0 | C |
| AT2G34250 | 4x | - | 500.150874030143 | 49.0 | C |
| AT1G24510 | 4x | - | 229.5325431256238 | 50.0 | C |
| AT5G58410 | 4x | - | 48.6812583253885 | 33.0 | C |
| CDS2 | 4x | - | 4414.470935939054 | 76.0 | H |
| DGK5 | 4x | - | 3.6552702025449424 | 24.0 | C |
| LPAT2 | 4x | - | 26.119667314844015 | 29.0 | C |
| CLS | 4x | - | 1.2186234577234705 | 10.0 | C |
| CDS1 | 4x | - | 4414.470935939054 | 76.0 | H |
| CA2 | 4x | - | 48.6812583253885 | 34.0 | C |
| LPAT3 | 4x | - | 26.119667314844015 | 29.0 | C |
| ATG18B | 2x_4x | - | 17737.230289554223 | 14.0 | B |
| AT2G41620 | 2x_4x | - | 57.083852797557284 | 4.0 | C |
| AT1G72730 | 4x | - | 105.87181432778245 | 54.0 | H |
| AT4G33945 | 4x | - | 48.6812583253885 | 32.0 | C |
| HOG1 | 2x_4x | - | 4048.2438778777764 | 56.0 | H |
| emb1441 | 4x | - | 7136.378623453272 | 42.0 | B |
| PGP1 | 4x | - | 0.996401235501249 | 9.0 | C |
| SCY2 | 4x | - | 390.37815386385046 | 43.0 | C |
| AT3G54470 | 4x | - | 5563.635240909616 | 46.0 | B |
| AT4G38780 | 2x_4x | - | 16219.526234158722 | 141.0 | HB |
| SDP1 | 2x_4x | - | 18525.56323957165 | 56.0 | HB |
| SCY1 | 4x | - | 5656.051494762174 | 45.0 | B |
| AT3G28870 | 2x_4x | - | 0.0 | 1.0 | H |
| AT5G60460 | 4x | - | 50.94226076324678 | 44.0 | C |
| ATS2 | 2x_4x | - | 3708.9969129066394 | 62.0 | HB |
| AT3G22980 | 4x | 2x | 195.28627782031782 | 63.0 | H |
| ALDH2C4 | 2x_4x | - | 83081.3602072639 | 111.0 | HB |
| NIA2 | 2x_4x | - | 270.3460242779832 | 59.0 | H |
| SMO1-3 | 2x_4x | - | 3114.9935831769194 | 183.0 | H |
| TTL2 | 4x | - | 66.2508594697682 | 44.0 | C |
| DGK6 | 4x | - | 3.6552702025449424 | 24.0 | C |
| LPLAT1 | 4x | - | 6.7161101988688685 | 31.0 | C |
| RAN-1 | 2x_4x | - | 1057.221844300385 | 6.0 | C |
| F18B13.15 | 2x_4x | - | 23801.06811092902 | 140.0 | HB |
| AT5G64270 | 2x_4x | - | 11517.295651964265 | 106.0 | HB |
| AT3G20920 | 4x | - | 50.791910448314674 | 41.0 | C |
| BSL2 | 4x | - | 270.41690242259415 | 49.0 | C |
| LPAT4 | 4x | - | 26.119667314844015 | 30.0 | C |
| AT4G21110 | 2x_4x | - | 7878.225680239669 | 102.0 | HB |
| PGPS2 | 4x | - | 0.996401235501249 | 9.0 | C |
| ALATS | 4x | - | 52.374435495611074 | 34.0 | C |
| OPT7 | 4x | - | 0.0 | 1.0 | C |
| GAPC2 | 4x | - | 199.74151014070816 | 51.0 | C |
| Hsp81.4 | 4x | - | 635.2175618968138 | 67.0 | H |
| PCNA1 | 2x_4x | - | 70296.68213888575 | 120.0 | HB |
| AT1G78720 | 4x | - | 500.150874030143 | 49.0 | C |
| PI4KALPHA1 | 2x_4x | - | 33888.72054479958 | 22.0 | HB |
| UBP20 | 4x | - | 64.37959944086553 | 43.0 | C |
| AT2G43240 | 4x | 2x | 0.833411568280312 | 9.0 | C |
| DGK3 | 2x_4x | - | 321.50577380228674 | 4.0 | C |
| RAN2 | 2x_4x | - | 1057.221844300385 | 6.0 | C |
| AT3G01340 | 4x | - | 48.6812583253885 | 33.0 | C |
| AT1G51040 | 2x_4x | - | 893.6093830420385 | 11.0 | HB |
| UPL4 | 2x_4x | - | 68.01501868455043 | 36.0 | H |
| AT1G58684 | 2x_4x | - | 457.1647508314564 | 22.0 | H |
| AT4G24920 | 2x_4x | - | 12.111985453458592 | 7.0 | H |
| TIM | 2x_4x | 2x | 68.68410959758766 | 20.0 | HB |
| PUR4 | 2x_4x | - | 161.80205977508436 | 66.0 | H |
| TSK | 4x | - | 87.94172748828082 | 51.0 | H |
| PGK | 2x_4x | - | 0.0 | 1.0 | C |
| FUM1 | 2x_4x | - | 14044.030610639591 | 70.0 | HB |
| DGK4 | 4x | - | 3.6552702025449424 | 24.0 | C |
| GLDP1 | 2x_4x | - | 5251.356441669151 | 81.0 | H |
| PAH2 | 4x | - | 27.081976259861964 | 27.0 | C |
| RAN3 | 2x_4x | - | 1057.221844300385 | 6.0 | C |
| OPT4 | 4x | - | 0.0 | 2.0 | C |
| OPT6 | 4x | - | 0.0 | 1.0 | C |
| RSR4 | 4x | - | 0.0 | 1.0 | C |
| OPT1 | 4x | - | 0.0 | 1.0 | C |
| AT1G71060 | 4x | 4x | 0.0 | 1.0 | C |
| EMB2744 | 4x | 4x | 0.0 | 1.0 | C |
| OPT2 | 4x | - | 0.0 | 1.0 | C |
| OPT5 | 4x | - | 0.0 | 1.0 | C |
| SECE1 | 4x | - | 0.0 | 2.0 | C |
| ALB3 | 4x | - | 2.9464547902714777 | 3.0 | C |
| ADG1 | 4x | 2x | 0.0 | 1.0 | C |
| OPT8 | 4x | - | 0.0 | 1.0 | C |
| OPT3 | 4x | 2x | 0.0 | 1.0 | C |
| ExLA1 | 4x | - | 0.0 | 1.0 | C |
| OPT9 | 4x | - | 0.0 | 1.0 | C |
| AT5G47700 | 2x_4x | - | 266.4374769694175 | 41.0 | H |
| AT4G15000 | 2x_4x | - | 1912.5850164038227 | 67.0 | H |
| RPL24A | 2x_4x | - | 1699.257941708257 | 74.0 | H |
| AT4G00810 | 2x_4x | - | 1206.1196813472204 | 58.0 | H |
| AT1G07070 | 2x_4x | - | 3705.4621197959486 | 94.0 | H |
| AT3G22230 | 2x_4x | - | 10668.805877412835 | 101.0 | H |
| AT3G48570 | 2x_4x | - | 40.66322941170046 | 17.0 | H |
| AT3G28900 | 2x_4x | - | 863.2215158318561 | 70.0 | H |
| AT1G02830 | 2x_4x | - | 753.9323498721955 | 39.0 | H |
| RPL16B | 2x_4x | - | 17617.775765021717 | 82.0 | H |
| AT5G02450 | 2x_4x | - | 1829.5772163206082 | 75.0 | H |
| AT1G74270 | 2x_4x | - | 2122.8497048888307 | 47.0 | H |
| RPL34 | 2x_4x | - | 682.5630516075 | 52.0 | H |
| AT3G06700 | 2x_4x | - | 4152.024273814578 | 99.0 | H |
| AT5G27770 | 2x_4x | - | 7834.100900602356 | 55.0 | H |
| AT3G05560 | 2x_4x | - | 394.3704130393981 | 27.0 | H |
| AT1G15250 | 2x_4x | - | 2370.6807928789744 | 72.0 | H |
| AT1G41880 | 2x_4x | - | 10939.999641518994 | 73.0 | H |
| AT1G26880 | 2x_4x | - | 990.3904912873252 | 52.0 | H |
| AT4G18100 | 2x_4x | - | 1603.3891619885674 | 61.0 | H |
| SEC61G2 | 2x_4x | - | 30.963235378451333 | 19.0 | H |
| AT5G24510 | 2x_4x | - | 676.9620682117641 | 49.0 | H |
| AT2G19730 | 2x_4x | - | 1202.9941515682924 | 67.0 | H |
| AT3G16080 | 2x_4x | - | 17936.151680143565 | 100.0 | H |
| AT5G45775.2 | 2x_4x | - | 3972.116412717143 | 70.0 | H |
| AT2G32220 | 2x_4x | - | 476.2852977157622 | 55.0 | H |
| PGY1 | 2x_4x | - | 22793.712167729038 | 87.0 | H |
| AT3G23390 | 2x_4x | - | 2318.447416791236 | 68.0 | H |
| AT3G02190 | 2x_4x | - | 334.88934552228653 | 47.0 | H |
| BUB3.2 | 2x_4x | - | 21009.506707523637 | 62.0 | HB |
| AT1G01100 | 2x_4x | 2x | 166.09025836609362 | 30.0 | H |
| AT4G29410 | 2x_4x | - | 1115.5724259468275 | 59.0 | H |
| AT5G22440 | 2x_4x | - | 5848.324103707392 | 102.0 | H |
| AT1G57660 | 2x_4x | - | 2109.399929470746 | 63.0 | H |
| AT3G06680 | 2x_4x | - | 28535.88549160753 | 106.0 | H |
| STV1 | 2x_4x | - | 1378.609517920937 | 69.0 | H |
| AT3G58700.1 | 2x_4x | - | 12423.727509268781 | 73.0 | H |
| AT2G25210 | 2x_4x | - | 24959.86871125309 | 109.0 | H |
| AT5G46430 | 2x_4x | - | 1273.9763506840002 | 67.0 | H |
| AT2G37600 | 2x_4x | - | 25806.111787397534 | 102.0 | H |
| AT3G53740 | 2x_4x | - | 2291.635149177103 | 74.0 | H |
| AT3G55750 | 2x_4x | - | 1092.7762021531169 | 61.0 | H |
| AT1G52300 | 2x_4x | - | 521.6660384415301 | 41.0 | H |
| AT1G08360 | 2x_4x | - | 2098.2400140170134 | 66.0 | H |
| CLASP | 2x_4x | 4x | 193.60422677778698 | 19.0 | C |
| BUB3.1 | 2x_4x | - | 28999.200059673534 | 65.0 | HB |
| EB1a | 2x_4x | - | 1311.739056870822 | 22.0 | C |
| AT1G69400 | 2x_4x | - | 25462.323243455754 | 65.0 | HB |
| BUBR1 | 2x_4x | - | 633.4446652112356 | 32.0 | C |
| MAPKKK7 | 4x | 2x | 0.0 | 1.0 | C |
| RUB3 | 2x_4x | - | 104252.38226118709 | 269.0 | HB |
| CUL2 | 2x_4x | - | 188.58929881262318 | 7.0 | C |
| CUL3B | 2x_4x | - | 14.606109354950165 | 5.0 | C |
| RUB1 | 2x_4x | - | 60430.49613704171 | 428.0 | HB |
| AT1G53980 | 2x_4x | - | 61490.89642031232 | 432.0 | HB |
| AT3G11130 | 2x_4x | - | 20450.95892600996 | 8.0 | HB |
| AT5G37340 | 2x_4x | 2x | 0.0 | 2.0 | C |
| AT2G46500 | 2x_4x | - | 60183.14774105252 | 382.0 | HB |
| AT5G11710 | 4x | - | 741.0684605861611 | 32.0 | C |
| DL1 | 4x | - | 824.9028178000281 | 34.0 | C |
| alpha-ADR | 2x_4x | - | 0.5 | 4.0 | C |
| AT1G08670 | 4x | - | 580.0219861813513 | 32.0 | C |
| ERMO2 | 4x | - | 4102.338879118187 | 38.0 | C |
| AT5G47320.1 | 4x | - | 4247.061829077224 | 88.0 | H |
| AT4G01000 | 2x_4x | - | 277550.5568755011 | 453.0 | HB |
| AT3G24530 | 2x_4x | - | 37551.38999111104 | 93.0 | HB |
| AT3G59290 | 4x | - | 894.8014815717436 | 40.0 | C |
| AT1G20760 | 4x | - | 837.6583777171337 | 41.0 | C |
| CYTC-1 | 2x_4x | - | 11200.068061785765 | 31.0 | HB |
| AT1G27530 | 4x | - | 4.739260452187376 | 4.0 | C |
| AT1G14570 | 4x | 4x | 8812.640620931235 | 67.0 | HB |
| CDC48B | 4x | - | 79.59114109480174 | 47.0 | C |
| AT1G43140 | 2x_4x | - | 21.265934080039198 | 6.0 | C |
| UFD1 | 4x | - | 124.7937399465555 | 46.0 | C |
| AT1G53930 | 2x_4x | - | 57647.17474729158 | 430.0 | HB |
| NPL41 | 4x | - | 65.96024331428822 | 43.0 | C |
| AT4G04180 | 4x | - | 53.80945310070713 | 43.0 | C |
| AT3G18860 | 4x | - | 1885.2375539664329 | 62.0 | H |
| AT4G32640 | 4x | - | 500.3546565029131 | 12.0 | C |
| AT4G24550 | 4x | - | 3.8293234729022925 | 11.0 | C |
| AT3G46540 | 4x | - | 725.2222518876588 | 38.0 | C |
| CDC48 | 4x | - | 305.3105748524955 | 66.0 | H |
| CYTC-2 | 2x_4x | - | 10240.604735064493 | 28.0 | HB |
| AT1G59800 | 2x_4x | - | 7.685159612039546 | 5.0 | C |
| AtCDC48B | 4x | - | 504.54366374503394 | 69.0 | H |
| AT5G24240 | 2x_4x | - | 40235.98136806216 | 375.0 | HB |
| AT4G15420 | 4x | - | 121.85021932612985 | 45.0 | C |
| AT2G20790 | 4x | - | 1.2689255189255189 | 10.0 | C |
| AT3G23350 | 4x | - | 466.75452431005675 | 38.0 | C |
| AT2G29070 | 4x | - | 104.2313488035155 | 42.0 | C |
| PK1 | 2x_4x | - | 66.59207690808493 | 35.0 | C |
| AT4G38930 | 4x | - | 122.43635857902873 | 45.0 | C |
| AT2G47970 | 4x | - | 120.54859423199572 | 46.0 | C |
| HAP13 | 4x | - | 497.429179183857 | 10.0 | C |
| UBQ12 | 2x_4x | - | 58950.080258837836 | 436.0 | HB |
| AtCDC48C | 4x | - | 505.19208342798674 | 67.0 | H |
| CUL1 | 2x_4x | - | 143.0265540408114 | 6.0 | H |
| S6K2 | 2x_4x | 2x | 120453.93214834163 | 71.0 | HB |
| AT1G64470 | 2x_4x | - | 55975.07216787147 | 428.0 | HB |
| AT1G53950 | 2x_4x | - | 60773.74466581681 | 437.0 | HB |
| AT4G02620 | 2x_4x | 2x | 5647.6439428392005 | 42.0 | HB |
| AT1G11970 | 2x_4x | - | 105513.95966862842 | 262.0 | HB |
| AT1G59790 | 2x_4x | - | 28.93068383177068 | 5.0 | C |
| UCH3 | 4x | - | 1230.267514860752 | 35.0 | C |
| AT2G43160 | 4x | - | 850.9534047396736 | 37.0 | C |
| AT5G22480 | 4x | - | 2646.674364229131 | 33.0 | C |
| AT1G25240 | 4x | 4x | 24.269715235849628 | 16.0 | C |
| AT1G10730 | 4x | - | 3.8293234729022925 | 11.0 | C |
| AT1G15890 | 4x | 4x | 0.4855573129293439 | 4.0 | C |
| AT5G22780 | 2x_4x | - | 0.5 | 4.0 | C |
| PORB | 2x_4x | - | 16.842292541939784 | 24.0 | C |
| PORC | 2x_4x | - | 79.36647052865038 | 33.0 | B |
| CH1 | 4x | 2x | 618.2933951671637 | 5.0 | C |
| TAF15b | 4x | - | 25.83470077748972 | 6.0 | C |
| SRx | 4x | - | 0.3333333333333333 | 4.0 | C |
| NYC1 | 2x_4x | - | 5654.0 | 2.0 | C |
| HCAR | 4x | - | 0.0 | 3.0 | C |
| CLH1 | 4x | 2x | 3945.1924910269736 | 6.0 | C |
| AT1G67325 | 4x | - | 48.288120658326015 | 7.0 | C |
| AT1G77550 | 4x | - | 168.30805969105865 | 8.0 | C |
| NOL | 2x_4x | - | 0.0 | 1.0 | C |
| CEF | 4x | - | 380.88314475848875 | 10.0 | C |
| CCP2 | 4x | - | 3.064516129032258 | 4.0 | C |
| ACLB-1 | 2x_4x | - | 9527.12185801241 | 74.0 | HB |
| mMDH1 | 2x_4x | - | 17720.84495887309 | 35.0 | HB |
| CSY1 | 2x_4x | - | 11947.918497693074 | 69.0 | HB |
| CSY3 | 2x_4x | - | 6128.614569635705 | 69.0 | HB |
| SDH2-1 | 4x | - | 20.771443129114537 | 14.0 | C |
| AT5G23250 | 4x | - | 724.0435654322506 | 41.0 | C |
| MDH | 2x_4x | - | 18671.77809809337 | 31.0 | HB |
| ASP3 | 2x_4x | - | 16946.54234155214 | 59.0 | HB |
| KAT5 | 4x | - | 60.42986831860344 | 22.0 | C |
| CSY5 | 2x_4x | - | 4934.348094122781 | 66.0 | HB |
| SDH3-2 | 4x | - | 7996.664302434739 | 54.0 | H |
| PMDH1 | 2x_4x | 2x | 1209.7597671102162 | 25.0 | H |
| ACLB-2 | 2x_4x | - | 12228.889031073093 | 77.0 | HB |
| ACLA-2 | 2x_4x | 4x | 2862.8612866292783 | 9.0 | H |
| KLCR1 | 4x | - | 6734.9578149007375 | 3.0 | C |
| KUF1 | 4x | 4x | 4199.153971850507 | 4.0 | C |
| SDH2-2 | 4x | - | 20.771443129114537 | 14.0 | C |
| AT5G55070 | 4x | - | 101.09646339661313 | 30.0 | C |
| SDH1-1 | 4x | - | 53.18082049301127 | 19.0 | C |
| PKT4 | 4x | - | 60.42986831860344 | 22.0 | C |
| c-NAD-MDH3 | 2x_4x | - | 225.15883336448928 | 17.0 | H |
| ATCS | 2x_4x | - | 3604.518200577482 | 65.0 | HB |
| PMDH2 | 2x_4x | - | 101127.6711267514 | 171.0 | HB |
| AT1G36280 | 2x_4x | - | 7.342577368129646 | 5.0 | C |
| BCE2 | 4x | - | 6.246292772537631 | 21.0 | C |
| SDH3-1 | 4x | - | 7996.664302434739 | 54.0 | H |
| AT3G08530 | 4x | - | 8882.693847587952 | 45.0 | B |
| PMI1 | 4x | 4x | 0.0 | 1.0 | C |
| AT3G55410 | 4x | - | 2387.1366150883164 | 37.0 | C |
| AT5G10920 | 4x | - | 20173.695276020255 | 33.0 | B |
| ACLA-3 | 2x_4x | - | 53.83807572608553 | 5.0 | H |
| ASP5 | 2x_4x | - | 29536.40065172064 | 60.0 | HB |
| AT5G58330 | 2x_4x | - | 749.1900434838503 | 21.0 | C |
| AT4G26910 | 4x | - | 101.02749787937177 | 29.0 | C |
| c-NAD-MDH1 | 2x_4x | - | 1745.8320535130247 | 30.0 | H |
| ASP4 | 2x_4x | - | 18856.088283708774 | 61.0 | HB |
| PCK1 | 2x_4x | - | 4287.848387329359 | 29.0 | H |
| AT4G18440 | 2x_4x | - | 396.8652233585354 | 2.0 | C |
| c-NAD-MDH2 | 2x_4x | - | 4211.078848572963 | 22.0 | H |
| LTA3 | 4x | - | 634.8585878103826 | 39.0 | C |
| AT4G17260 | 2x_4x | - | 96.43095582498198 | 15.0 | C |
| PCK2 | 2x_4x | - | 1729.1446350955114 | 28.0 | H |
| AT5G12040 | 4x | - | 30.081584713367207 | 31.0 | C |
| SDH2-3 | 4x | - | 20.771443129114537 | 14.0 | C |
| ASP1 | 2x_4x | 2x | 31094.28521339503 | 53.0 | HB |
| AT3G13930 | 4x | - | 641.9420880804372 | 40.0 | C |
| SDH1-2 | 4x | - | 53.18082049301127 | 19.0 | C |
| AT5G08300 | 4x | - | 1538.8003620007507 | 54.0 | H |
| AT5G65750 | 4x | - | 2387.1366150883164 | 37.0 | C |
| CSY2 | 2x_4x | - | 3794.241460752475 | 66.0 | HB |
| AT2G26800 | 4x | - | 13.450049792448567 | 26.0 | C |
| ACO3 | 4x | - | 1.3352183579551116 | 12.0 | C |
| PREP1 | 4x | - | 753.6846876245435 | 18.0 | C |
| mMDH2 | 2x_4x | - | 96.86181908616454 | 7.0 | C |
| EMB3003 | 4x | - | 646.0460601878843 | 38.0 | C |
| ASP2 | 2x_4x | - | 16173.839403931115 | 54.0 | HB |
| PKT3 | 4x | - | 60.42986831860344 | 22.0 | C |
| ARA7 | 4x | - | 1872.0231551612592 | 3.0 | C |
| F4B14.100 | 4x | - | 1.3352183579551116 | 12.0 | C |
| LTA2 | 4x | 2x | 6.246292772537631 | 22.0 | C |
| PREP2 | 4x | - | 753.6846876245435 | 18.0 | C |
| ARA6 | 4x | - | 3031.95546907081 | 4.0 | C |
| RABF2A | 4x | - | 1872.0231551612592 | 3.0 | C |
| ALDH5F1 | 2x_4x | - | 1.3554289332532663 | 3.0 | C |
| AT3G53910 | 4x | - | 50.225448641325016 | 24.0 | C |
| AT1G76110 | 4x | - | 14.665590573485693 | 6.0 | C |
| AT1G34300 | 4x | 4x | 0.0 | 2.0 | C |
| AT1G55650 | 4x | - | 14.665590573485693 | 6.0 | C |
| AT1G54220 | 4x | - | 634.8585878103826 | 39.0 | C |
| At4g26970 | 4x | - | 1.3352183579551116 | 12.0 | C |
| IMPA-4 | 4x | - | 0.0 | 1.0 | C |
| AT3G13350 | 4x | - | 14.665590573485693 | 6.0 | C |
| STZ | 4x | 2x | 1786.0184016796497 | 13.0 | C |
| WSIP2 | 2x_4x | - | 5171.8246768658 | 4.0 | C |
| AT1G04880 | 4x | - | 14.665590573485693 | 6.0 | C |
| SK18 | 4x | - | 354.3259366625151 | 4.0 | C |
| MPK3 | 4x | - | 0.0 | 3.0 | C |
| AT1G51200 | 4x | 4x | 2.3714187140049967 | 3.0 | C |
| MPK6 | 4x | - | 0.0 | 3.0 | C |
| MIRO3 | 4x | - | 3547.5294895632833 | 4.0 | C |
| STT3A | 4x | - | 446.035811050798 | 5.0 | C |
| AT1G11560 | 4x | - | 4145.363283611625 | 11.0 | B |
| RH8 | 4x | - | 2934.9999999995657 | 3.0 | C |
| AT1G66810 | 4x | - | 1788.9999999999995 | 10.0 | C |
| MIRO1 | 4x | - | 3547.5294895632833 | 4.0 | C |
| MIRO2 | 4x | - | 3547.5294895632833 | 4.0 | C |
| EBS1 | 4x | 4x | 17150.018656466735 | 28.0 | B |
| AT3G27325 | 4x | - | 5.015151515151515 | 8.0 | C |
| AT1G61790 | 4x | - | 4145.363283611625 | 11.0 | B |
| PLDALPHA3 | 4x | - | 3166.713963392942 | 58.0 | H |
| STT3B | 4x | - | 446.035811050798 | 5.0 | C |
| xYL1 | 4x | 2x | 0.18181818181818182 | 4.0 | C |
| PLDALPHA2 | 4x | - | 3166.713963392942 | 58.0 | H |
| AT2G16960 | 2x_4x | - | 103.23745238013893 | 55.0 | HB |
| PLDGAMMA2 | 4x | - | 3166.713963392942 | 58.0 | H |
| AT3G61240 | 2x_4x | - | 5654.0 | 2.0 | C |
| PLDP1 | 4x | - | 3166.713963392942 | 58.0 | H |
| AT3G45940 | 2x_4x | - | 10.177189324343969 | 7.0 | C |
| NAC075 | 4x | - | 0.0 | 1.0 | C |
| AT1G68200 | 4x | 4x | 8875.000000000002 | 12.0 | B |
| PLDP2 | 4x | - | 3166.713963392942 | 58.0 | H |
| HGL1 | 4x | - | 1065.933191243284 | 6.0 | C |
| AT1G05720 | 4x | - | 0.0 | 1.0 | C |
| COQ3 | 4x | - | 30561.332736520766 | 3.0 | C |
| AT2G03690 | 4x | - | 24.833333333333332 | 4.0 | C |
| AT5G19370 | 4x | - | 9979.666666665551 | 5.0 | C |
| PLDBETA1 | 4x | - | 3166.713963392942 | 58.0 | H |
| PLDALPHA1 | 4x | - | 3166.713963392942 | 58.0 | H |
| AT5G11720 | 2x_4x | - | 14355.383144444364 | 7.0 | B |
| AT5G38460 | 4x | - | 2.515151515151515 | 7.0 | C |
| ATTRN1 | 2x_4x | - | 103.23745238013893 | 55.0 | HB |
| AT2G45810 | 4x | - | 2934.9999999995657 | 3.0 | C |
| PLDDELTA | 4x | - | 3210.291980863522 | 59.0 | H |
| PLDGAMMA3 | 4x | - | 3166.713963392942 | 58.0 | H |
| AT1G71810 | 4x | 4x | 78323.41850894086 | 9.0 | B |
| CHLM | 2x_4x | - | 725.74978105912 | 91.0 | HB |
| PLDBETA2 | 4x | - | 3166.713963392942 | 58.0 | H |
| AT1G19140 | 4x | - | 0.0 | 2.0 | C |
| AT5G42470 | 4x | - | 0.0 | 1.0 | C |
| PIN1AT | 2x_4x | - | 0.0 | 4.0 | C |
| CYL1 | 4x | - | 0.0 | 1.0 | C |
| RSW3 | 4x | - | 1065.933191243284 | 6.0 | C |
| AT4G17650 | 4x | - | 0.0 | 1.0 | C |
| AT1G26550 | 4x | - | 0.0 | 3.0 | C |
| PNG1 | 4x | - | 0.0 | 2.0 | C |
| PLDEPSILON | 4x | - | 3166.713963392942 | 58.0 | H |
| AT1G60080 | 2x_4x | - | 396.6956495682533 | 18.0 | C |
| AT2G03500 | 2x_4x | - | 8.819317908133698 | 3.0 | C |
| PLDGAMMA1 | 4x | - | 3166.713963392942 | 58.0 | H |
| AT4G27280 | 4x | - | 58.47217042753109 | 14.0 | C |
| AT1G73110 | 4x | - | 1887.1670167145628 | 83.0 | H |
| UCH2 | 2x_4x | - | 327.5565996309639 | 85.0 | H |
| PBD2 | 2x_4x | - | 364.58494762497975 | 83.0 | H |
| UBP6 | 2x_4x | - | 96.56118047811636 | 69.0 | H |
| PBD1 | 2x_4x | - | 195.3658968743033 | 84.0 | H |
| FUS12 | 4x | - | 194.83501304479717 | 71.0 | H |
| EMB2107 | 2x_4x | - | 642.4127345258997 | 87.0 | H |
| DSS1(V) | 2x_4x | - | 1211.7921495599733 | 44.0 | H |
| HMGS | 4x | - | 7085.251280328748 | 24.0 | B |
| HMG1 | 4x | 4x | 0.0 | 2.0 | C |
| BRCC36A | 2x_4x | - | 898.149186087103 | 88.0 | H |
| WOL | 2x_4x | - | 4.386362282517885 | 10.0 | C |
| HP6 | 2x_4x | 4x | 17.56349206349204 | 11.0 | C |
| AT5G05510 | 2x_4x | - | 643.5586074362297 | 31.0 | C |
| MAD2 | 2x_4x | - | 29392.120920268244 | 72.0 | HB |
| MEE32 | 2x_4x | - | 64.48790253326345 | 9.0 | C |
| AT1G53790 | 2x_4x | - | 481.69620666359333 | 83.0 | H |
| RR22 | 4x | - | 0.0 | 4.0 | C |
| RCA | 2x_4x | 2x | 76600.66785258464 | 205.0 | HB |
| UBP7 | 2x_4x | - | 234.5064672026832 | 76.0 | H |
| BRCC36B | 2x_4x | - | 779.6881259654542 | 87.0 | H |
| DSS1(I) | 2x_4x | - | 503.30242745902774 | 46.0 | H |
| UCH1 | 2x_4x | - | 458.92286020092587 | 89.0 | H |
| RPN13 | 2x_4x | - | 1977.4891217291492 | 79.0 | H |
| AT3G15180 | 2x_4x | - | 8.35348631295578 | 40.0 | H |
| HK2 | 2x_4x | - | 4.386362282517885 | 10.0 | B |
| F24B18.11 | 4x | - | 58.47217042753109 | 14.0 | C |
| AT5G51830 | 2x_4x | - | 889.182158030136 | 10.0 | C |
| AT4G10260 | 2x_4x | - | 1474.6667469872289 | 13.0 | C |
| PPK1 | 2x_4x | 4x | 11523.403898737412 | 47.0 | HB |
| CML19 | 4x | - | 3840.0263197878025 | 16.0 | C |
| HK3 | 2x_4x | - | 4.386362282517885 | 10.0 | C |
| FBA7 | 2x_4x | - | 4281.403237372111 | 57.0 | H |
| AT1G76550 | 4x | 4x | 35308.0 | 11.0 | C |
| AT2G31390 | 2x_4x | - | 1271.727600747634 | 8.0 | C |
| AT2G46600 | 4x | - | 58.47217042753109 | 14.0 | C |
| FBA4 | 2x_4x | - | 684.3007450087234 | 38.0 | C |
| PDE345 | 2x_4x | - | 1705.1271205065377 | 39.0 | C |
| AT2G20635 | 2x_4x | - | 328.8032597758532 | 21.0 | C |
| AT1G61000 | 4x | - | 0.3333333333333333 | 5.0 | C |
| FBA5 | 2x_4x | - | 6878.876749514859 | 64.0 | H |
| FBA2 | 2x_4x | - | 38409.628575082876 | 132.0 | HB |
| AT1G60995 | 4x | - | 0.3333333333333333 | 5.0 | C |
| FBA8 | 2x_4x | - | 10820.668135141459 | 45.0 | C |
| AT3G54630 | 2x_4x | - | 3.1478021978021977 | 7.0 | C |
| CML20 | 4x | - | 3631.8490367241507 | 15.0 | C |
| MAD1 | 2x_4x | - | 314.6854971248933 | 28.0 | C |
| AT3G59480 | 2x_4x | - | 5218.747525230832 | 11.0 | C |
| MK | 4x | - | 0.0 | 2.0 | C |
| AT3G32040 | 4x | - | 273.30122535125327 | 96.0 | H |
| ABC4 | 2x_4x | 2x | 282.7422864217438 | 13.0 | B |
| GOx2 | 2x_4x | - | 74.24791774663464 | 45.0 | HB |
| AT2G18620 | 4x | - | 273.30122535125327 | 96.0 | H |
| cPT5 | 4x | - | 18.939573488286776 | 30.0 | C |
| G4 | 4x | - | 6533.651443289094 | 63.0 | B |
| HAOx1 | 4x | - | 318.838819212513 | 49.0 | C |
| AT3G14510 | 4x | - | 273.30122535125327 | 96.0 | H |
| HAOx2 | 4x | - | 277.5717056050594 | 48.0 | C |
| SPS2 | 4x | - | 118.00715688353316 | 70.0 | C |
| FPS1 | 4x | - | 39602.96472164577 | 84.0 | HB |
| MVD1 | 4x | - | 45.73524620930909 | 51.0 | C |
| GOx3 | 4x | - | 39.97263746894236 | 42.0 | C |
| GGPS3 | 4x | - | 273.30122535125327 | 96.0 | H |
| IPT6 | 4x | - | 504.5714285714661 | 37.0 | C |
| At2g24210 | 4x | - | 0.0 | 19.0 | C |
| GGPS4 | 4x | - | 273.30122535125327 | 96.0 | H |
| cPT9 | 4x | - | 24.03232770144903 | 32.0 | C |
| GGPS2 | 4x | - | 273.30122535125327 | 96.0 | H |
| AT2G23400 | 4x | - | 18.939573488286776 | 30.0 | C |
| IPT9 | 4x | - | 0.0 | 36.0 | C |
| FPS2 | 4x | - | 39602.96472164577 | 84.0 | HB |
| IPT5 | 4x | - | 504.5714285714661 | 37.0 | C |
| IPT3 | 4x | - | 504.5714285714661 | 37.0 | C |
| GPS1 | 4x | 4x | 241.6420145485693 | 93.0 | H |
| GGPS6 | 4x | - | 273.30122535125327 | 96.0 | H |
| APG5 | 2x_4x | 2x | 12458.577054494086 | 9.0 | B |
| ATG10 | 2x_4x | 4x | 85.50355093704545 | 2.0 | C |
| GGR | 4x | - | 273.30122535125327 | 96.0 | H |
| PPT1 | 4x | - | 3527.208786155201 | 59.0 | C |
| AT5G58784 | 4x | - | 18.939573488286776 | 30.0 | C |
| ECR1 | 4x | - | 21637.02163742976 | 8.0 | C |
| ATG12A | 2x_4x | - | 274.7001740495231 | 5.0 | C |
| cPT8 | 4x | - | 24.03232770144903 | 32.0 | C |
| AT3G54250 | 4x | - | 45.73524620930909 | 51.0 | C |
| IPP1 | 4x | - | 20969.118381956054 | 69.0 | B |
| AT3G29430 | 4x | - | 273.30122535125327 | 96.0 | H |
| IPT2 | 4x | - | 0.0 | 36.0 | C |
| AT3G25810 | 4x | - | 0.0 | 19.0 | C |
| AxL | 4x | - | 0.1111111111111111 | 5.0 | C |
| cPT6 | 4x | - | 18.939573488286776 | 30.0 | C |
| IPT8 | 4x | - | 504.5714285714661 | 37.0 | C |
| IPT1 | 4x | - | 504.5714285714661 | 37.0 | C |
| HPT2 | 4x | - | 1879.2808272772877 | 60.0 | C |
| HPT1 | 4x | 2x | 2115.240212044776 | 62.0 | C |
| AT3G20160 | 4x | - | 273.30122535125327 | 96.0 | H |
| CUL3 | 2x_4x | - | 37.51081986472767 | 4.0 | B |
| CPT | 4x | - | 18.939573488286776 | 30.0 | C |
| GGPS1 | 4x | - | 273.30122535125327 | 96.0 | H |
| AT3G14530 | 4x | - | 273.30122535125327 | 96.0 | H |
| TPS23 | 4x | - | 0.10526315789473684 | 20.0 | C |
| IPP2 | 4x | - | 20969.118381956054 | 69.0 | B |
| SPS1 | 4x | - | 118.00715688353316 | 70.0 | C |
| AxR1 | 4x | - | 0.1111111111111111 | 5.0 | C |
| IPT4 | 4x | - | 504.5714285714661 | 37.0 | C |
| AT-SAE1-2 | 4x | - | 12655.540935673003 | 6.0 | C |
| APG7 | 4x | - | 0.4444444444444444 | 7.0 | C |
| GOx1 | 4x | - | 10944.334714791137 | 50.0 | C |
| HDR | 4x | - | 66.17794703556595 | 63.0 | C |
| cPT4 | 4x | - | 8252.032476473147 | 31.0 | C |
| APG12B | 2x_4x | - | 0.0 | 3.0 | B |
| UBA1 | 4x | - | 0.1111111111111111 | 5.0 | C |
| ATG3 | 2x_4x | - | 17404.56642815189 | 9.0 | B |
| UBA2 | 2x_4x | - | 0.0 | 1.0 | C |
| AT2G44140 | 4x | - | 0.1111111111111111 | 6.0 | C |
| cPT1 | 4x | - | 24.03232770144903 | 32.0 | C |
| TPS14 | 4x | - | 0.10526315789473684 | 20.0 | C |
| At5g50680 | 4x | - | 12655.540935673003 | 7.0 | C |
| IPT7 | 4x | - | 504.5714285714661 | 37.0 | C |
| AT3G59950 | 4x | - | 0.1111111111111111 | 6.0 | C |
| AT1G05350 | 4x | - | 0.1111111111111111 | 4.0 | C |
| SAE2 | 4x | - | 1110.7356725144962 | 9.0 | C |
| AT3G25880 | 4x | - | 0.1111111111111111 | 5.0 | C |
| CNx5 | 4x | - | 0.1111111111111111 | 4.0 | C |
| BPM2 | 4x | 4x | 7228.089282630704 | 7.0 | B |
| RAP2.4 | 4x | - | 0.0 | 1.0 | C |
| HAG2 | 2x_4x | - | 8024.283597699289 | 7.0 | B |
| CUL4 | 4x | - | 30698.022991077636 | 56.0 | HB |
| SAE1A | 4x | - | 12655.540935673003 | 7.0 | C |
| AT4G12100 | 4x | - | 9682.40603211784 | 41.0 | B |
| NAC083 | 4x | - | 0.0 | 1.0 | C |
| AT3G46910 | 4x | - | 11900.512673376477 | 46.0 | B |
| GSTT1 | 4x | - | 0.27053898482469907 | 8.0 | C |
| GPx6 | 4x | 4x | 31263.12574089186 | 61.0 | HB |
| AT5G48700 | 4x | - | 35.006209169081735 | 9.0 | C |
| D22 | 2x_4x | - | 0.0 | 1.0 | C |
| AT1G65820 | 4x | - | 0.27053898482469907 | 8.0 | C |
| GGT2 | 2x_4x | - | 2833.0 | 3.0 | H |
| GSH2 | 4x | - | 220.88495132686907 | 52.0 | H |
| GSTL3 | 4x | - | 15.157718472004179 | 21.0 | C |
| AT5G19490 | 4x | - | 0.3333333333333333 | 5.0 | C |
| NF-YB12 | 2x_4x | - | 594.4294873970596 | 2.0 | C |
| GSTU9 | 4x | - | 0.37580214271943596 | 10.0 | C |
| GR | 2x_4x | - | 4.045984527390821 | 3.0 | HB |
| NRPC1 | 2x_4x | - | 275.74944512393904 | 6.0 | B |
| GSTU5 | 4x | - | 0.27053898482469907 | 8.0 | C |
| GSTF11 | 4x | - | 0.27053898482469907 | 8.0 | C |
| GGT3 | 2x_4x | - | 8482.0 | 4.0 | HB |
| GSTF4 | 4x | - | 0.27053898482469907 | 8.0 | C |
| GSTL2 | 4x | - | 47.22741544170154 | 30.0 | C |
| GSTU23 | 4x | - | 0.27053898482469907 | 9.0 | C |
| RPOC2 | 4x | - | 171.15792010281473 | 16.0 | C |
| AT5G55856 | 4x | - | 35.006209169081735 | 12.0 | C |
| GSTL1 | 4x | - | 21.035496249781964 | 23.0 | C |
| LOG4 | 4x | 4x | 1.641025641025642 | 6.0 | C |
| SUMO2 | 4x | - | 35.006209169081735 | 9.0 | C |
| HMG | 2x_4x | - | 7795.858505026674 | 15.0 | HB |
| AT5G59140 | 4x | - | 1184.8145068296787 | 20.0 | C |
| GGT4 | 2x_4x | - | 5654.0 | 2.0 | B |
| GSTF3 | 4x | - | 0.27053898482469907 | 8.0 | C |
| GSTU21 | 4x | - | 0.27053898482469907 | 9.0 | C |
| GSTU14 | 4x | - | 0.37053898482469916 | 10.0 | C |
| GSTU13 | 4x | - | 0.37053898482469916 | 10.0 | C |
| TRx1 | 4x | 2x | 2027.2312854681447 | 10.0 | C |
| PER1 | 2x_4x | - | 7937.199753724566 | 19.0 | B |
| GSTF12 | 4x | - | 0.27053898482469907 | 8.0 | C |
| GSTU10 | 4x | - | 0.27053898482469907 | 9.0 | C |
| GSTU2 | 2x_4x | - | 0.0 | 1.0 | C |
| GSTF6 | 4x | - | 0.27053898482469907 | 9.0 | C |
| At3g24170 | 2x_4x | - | 78825.03654873164 | 5.0 | HB |
| ERD9 | 4x | - | 0.37580214271943596 | 10.0 | C |
| SUMO1 | 4x | - | 296.97097927868737 | 13.0 | C |
| GSTF7 | 4x | - | 0.4927612070469213 | 10.0 | C |
| GSTU7 | 2x_4x | 2x | 11330.0 | 5.0 | B |
| AT5G08565 | 2x_4x | - | 741.8793686205621 | 8.0 | C |
| GSTU24 | 4x | - | 0.600802142719436 | 11.0 | C |
| RLP51 | 4x | 4x | 15.08139723337347 | 4.0 | C |
| AT5G48710 | 4x | - | 35.006209169081735 | 9.0 | C |
| GSTU19 | 4x | - | 0.7190238333095477 | 13.0 | C |
| GSTU26 | 2x_4x | - | 0.0 | 1.0 | C |
| GSTU22 | 4x | - | 0.5622056514913658 | 11.0 | C |
| GSTU11 | 4x | - | 0.27053898482469907 | 9.0 | C |
| GSTF8 | 4x | - | 0.27053898482469907 | 9.0 | C |
| GSTU16 | 4x | - | 0.27053898482469907 | 8.0 | C |
| GSTU15 | 4x | - | 0.37053898482469916 | 10.0 | C |
| GSTU3 | 4x | - | 0.600802142719436 | 11.0 | C |
| GSTF5 | 4x | - | 0.27053898482469907 | 8.0 | C |
| GSTU1 | 2x_4x | - | 5665.0 | 3.0 | C |
| SUMO3 | 4x | - | 35.006209169081735 | 12.0 | C |
| GSTU27 | 4x | - | 0.600802142719436 | 11.0 | C |
| mtLPD1 | 4x | - | 2099.4566487798224 | 27.0 | C |
| NRPA1 | 2x_4x | - | 471.34609955551963 | 12.0 | B |
| SUMO5 | 4x | - | 35.006209169081735 | 12.0 | C |
| RGD3 | 4x | 4x | 3376.462888110037 | 27.0 | C |
| SPT42 | 2x_4x | - | 1749.412223335745 | 5.0 | C |
| GSTU18 | 4x | - | 0.27053898482469907 | 10.0 | C |
| mtLPD2 | 4x | - | 2099.4566487798224 | 27.0 | C |
| GSTU12 | 4x | - | 0.37053898482469916 | 10.0 | C |
| GSTF10 | 4x | - | 0.27053898482469907 | 8.0 | C |
| GSTF2 | 4x | - | 0.27053898482469907 | 8.0 | C |
| ATGSTF13 | 4x | - | 0.27053898482469907 | 8.0 | C |
| SGT1A | 2x_4x | - | 1288.9227939562943 | 16.0 | H |
| NF-YB13 | 2x_4x | - | 998.2073137216881 | 4.0 | C |
| AT4G16155 | 4x | - | 2099.4566487798224 | 26.0 | C |
| GSTF14 | 4x | - | 0.27053898482469907 | 8.0 | C |
| LPD1 | 4x | - | 2099.4566487798224 | 26.0 | C |
| NF-YC11 | 4x | - | 0.3333333333333333 | 5.0 | C |
| GSTU4 | 4x | - | 0.600802142719436 | 11.0 | C |
| GSTU20 | 4x | - | 0.27053898482469907 | 9.0 | C |
| GSTU25 | 2x_4x | - | 6.0 | 2.0 | C |
| KLCR2 | 4x | - | 29470.12120674595 | 3.0 | B |
| GSTU6 | 4x | - | 0.37580214271943596 | 10.0 | C |
| RPOC1 | 4x | - | 171.15792010281473 | 16.0 | C |
| GSTU8 | 4x | - | 0.27053898482469907 | 9.0 | C |
| SGT1B | 2x_4x | - | 12099.357603039602 | 21.0 | HB |
| GSTU28 | 4x | - | 0.27053898482469907 | 9.0 | C |
| AT3G11630 | 4x | - | 0.0 | 4.0 | C |
| At5g06290 | 4x | - | 0.0 | 4.0 | C |
| MC9 | 4x | - | 3544.025641025641 | 3.0 | C |
| AT3G53040 | 4x | 4x | 0.0 | 1.0 | C |
| RPT4A | 4x | - | 3182.362232561916 | 132.0 | H |
| HBT | 2x_4x | - | 1478.7305784122589 | 32.0 | HB |
| AKRP | 4x | - | 48.06449037760433 | 100.0 | H |
| RPN8A | 2x_4x | - | 664.1679715779146 | 87.0 | H |
| RPT5A | 2x_4x | - | 614.4680221289218 | 85.0 | H |
| AT4G24820 | 2x_4x | - | 18490.550783775565 | 147.0 | HB |
| AT2G03430 | 2x_4x | - | 697.1049346529701 | 60.0 | H |
| APC4 | 2x_4x | - | 8276.614346019747 | 59.0 | HB |
| APC7 | 4x | - | 287.39347975453916 | 87.0 | H |
| CDC20.4 | 2x_4x | - | 279.7196648281754 | 37.0 | H |
| AT1G04810 | 2x_4x | - | 3139.6273341690658 | 96.0 | H |
| PAC1 | 2x_4x | - | 418.817059158905 | 88.0 | HB |
| UBC10 | 2x_4x | - | 2.6958134201394226 | 15.0 | H |
| UBC20 | 2x_4x | - | 10753.870415931333 | 71.0 | HB |
| FUS9 | 2x_4x | - | 2.6958134201394226 | 15.0 | H |
| PAE1 | 2x_4x | - | 419.2070490178861 | 82.0 | H |
| AT1G53780 | 2x_4x | - | 3544.7014737786844 | 97.0 | H |
| RPN1A | 2x_4x | - | 16662.13529873574 | 127.0 | HB |
| UBC19 | 2x_4x | - | 10753.870415931333 | 71.0 | HB |
| AT2G05840.1 | 2x_4x | - | 353.01765719040685 | 89.0 | H |
| EMB2719 | 2x_4x | - | 408.1512084804405 | 100.0 | H |
| ATS9 | 2x_4x | - | 311.24580507580606 | 91.0 | H |
| RPN1B | 2x_4x | - | 800.0210408103007 | 96.0 | H |
| RPT2a | 2x_4x | - | 609.4555945126184 | 81.0 | HB |
| APC10 | 2x_4x | - | 1244.2930961597174 | 33.0 | H |
| PAG1 | 2x_4x | - | 392.9654636786567 | 80.0 | H |
| CDC27a | 2x_4x | - | 141.0709798834121 | 31.0 | H |
| AT1G75990 | 2x_4x | 4x | 503.79097534844647 | 89.0 | H |
| AT5G20000 | 2x_4x | - | 6326.66311003321 | 109.0 | HB |
| CDC20.1 | 2x_4x | - | 385.14930730157795 | 38.0 | H |
| APC8 | 2x_4x | - | 2230.9779430202416 | 33.0 | HB |
| AT1G45000 | 4x | - | 10816.943818077934 | 173.0 | H |
| PBC2 | 2x_4x | - | 77.29121935781176 | 85.0 | H |
| FZR2 | 4x | - | 947.1213957932918 | 95.0 | H |
| UBC12 | 2x_4x | - | 2.6958134201394226 | 15.0 | H |
| MEE34 | 2x_4x | - | 691.2984022195226 | 88.0 | H |
| APC6 | 2x_4x | - | 197.27800354253924 | 32.0 | H |
| UBC9 | 2x_4x | - | 928.6548812388934 | 16.0 | H |
| AT2G04660.1 | 2x_4x | - | 41.162473205385936 | 26.0 | H |
| AT5G45620 | 2x_4x | - | 487.84507570395016 | 91.0 | H |
| PAF2 | 2x_4x | - | 397.05910142105813 | 81.0 | H |
| PBC1 | 2x_4x | - | 244.3967753996914 | 87.0 | H |
| K2K18.4 | 2x_4x | - | 431.83348033987085 | 91.0 | H |
| PAF1 | 2x_4x | - | 3199.0350210285787 | 81.0 | HB |
| PAE2 | 2x_4x | - | 283.078698854568 | 84.0 | H |
| UBC30 | 2x_4x | - | 2.6958134201394226 | 15.0 | H |
| AT2G36170 | 2x_4x | - | 395.5488446124739 | 34.0 | HB |
| AT4G08140 | 2x_4x | - | 540.9761809726456 | 95.0 | H |
| AT5G27945 | 2x_4x | - | 279.7196648281754 | 37.0 | H |
| RPT2b | 2x_4x | - | 514.5390596687118 | 83.0 | H |
| AT5G23540 | 2x_4x | - | 745.388311101748 | 83.0 | HB |
| PBB1 | 2x_4x | - | 492.593288651162 | 86.0 | H |
| RPN12b | 2x_4x | - | 730.1432748523001 | 92.0 | H |
| RPT6A | 2x_4x | - | 7545.288581736027 | 102.0 | HB |
| RPN10 | 2x_4x | - | 4305.914464268491 | 101.0 | HB |
| FZR3 | 4x | - | 482.2869699075463 | 92.0 | H |
| UVI4 | 4x | - | 0.6188021480973758 | 9.0 | C |
| RPT5B | 2x_4x | - | 718.4121549969364 | 85.0 | H |
| EMB2771 | 2x_4x | - | 68.28886762642982 | 28.0 | H |
| AT2G20050 | 2x_4x | - | 77216.89442317798 | 76.0 | HB |
| RPN5B | 2x_4x | 2x | 629.6269579205463 | 90.0 | H |
| PAD2 | 2x_4x | - | 495.5937286615499 | 90.0 | H |
| F3O9.27 | 2x_4x | - | 447.55045898206316 | 87.0 | H |
| AT5G57950 | 2x_4x | - | 168.37832357103107 | 64.0 | H |
| PBG1 | 2x_4x | - | 7013.2411969469795 | 107.0 | H |
| CDC2 | 2x_4x | - | 68020.85586268123 | 81.0 | HB |
| RPT1A | 2x_4x | - | 3731.742314861381 | 97.0 | H |
| UBC29 | 2x_4x | - | 2.6958134201394226 | 15.0 | H |
| CDC20.2 | 2x_4x | - | 24068.6188027118 | 51.0 | HB |
| AT4G15165 | 2x_4x | - | 346.21717509326146 | 91.0 | H |
| AT1G79210 | 2x_4x | - | 451.7575041276232 | 97.0 | H |
| PBE1 | 2x_4x | - | 177.4435034271909 | 79.0 | H |
| PAD1 | 2x_4x | - | 547.7195152319894 | 90.0 | H |
| CDC20.5 | 2x_4x | - | 279.7196648281754 | 37.0 | H |
| PBF1 | 2x_4x | - | 623.9541161076307 | 92.0 | HB |
| RPN12a | 2x_4x | - | 692.5586129465479 | 90.0 | H |
| UBC8 | 2x_4x | - | 928.6548812388934 | 16.0 | H |
| AT1G06590 | 2x_4x | - | 65.67741888338433 | 39.0 | H |
| PBB2 | 2x_4x | - | 334.8079773311077 | 88.0 | H |
| EMB506 | 4x | - | 48.06449037760433 | 101.0 | H |
| CCS52A2 | 4x | - | 171.62374765871073 | 89.0 | H |
| PA200 | 2x_4x | 2x | 368.8296621173498 | 85.0 | H |
| AT3G26340 | 2x_4x | - | 345.17798664329496 | 81.0 | H |
| CDC20.3 | 2x_4x | - | 279.7196648281754 | 37.0 | H |
| AT4G19006 | 2x_4x | - | 690.4960844218925 | 89.0 | H |
| AT1G52360 | 4x | - | 1207.429018281644 | 2.0 | C |
| AT2G32730 | 2x_4x | - | 12489.419162947857 | 154.0 | H |
| AT5G11980 | 4x | - | 24284.64444029264 | 8.0 | B |
| AT4G01400 | 4x | - | 2.8333333333333335 | 5.0 | C |
| RPT3 | 2x_4x | - | 10109.829219363475 | 148.0 | HB |
| PBA1 | 2x_4x | - | 210.97663844095402 | 85.0 | H |
| UBC11 | 2x_4x | - | 2.6958134201394226 | 15.0 | H |
| AT1G73430 | 4x | - | 29.418334730859975 | 6.0 | C |
| AT1G67930 | 4x | - | 6.098958030995474 | 5.0 | C |
| AT1G31780 | 4x | - | 2832.536100045444 | 7.0 | C |
| AT4G24840 | 4x | 4x | 7093.265624697659 | 8.0 | B |
| EMB2777 | 4x | - | 0.0 | 1.0 | C |
| AT2G28450 | 4x | 4x | 4711.054667015054 | 29.0 | B |
| AT5G16300 | 4x | - | 2.1666666666666665 | 4.0 | C |
| AT4G33080 | 2x_4x | 4x | 0.0 | 2.0 | B |
| MAP4K1 | 4x | - | 0.0 | 1.0 | C |
| UPL5 | 2x_4x | - | 49903.653932481255 | 99.0 | HB |
| AT5G03500 | 4x | - | 0.0 | 1.0 | C |
| AT4G28230 | 4x | - | 0.0 | 4.0 | C |
| EYE | 4x | - | 5.432291364328807 | 4.0 | C |
| AT5G15680 | 4x | - | 0.0 | 1.0 | C |
| LDOx | 4x | - | 2.7499999999999996 | 10.0 | C |
| UBC17 | 4x | - | 237.51505866343166 | 7.0 | C |
| AT3G42830 | 2x_4x | - | 1121.423733371012 | 8.0 | C |
| APC11 | 4x | - | 998.8204298279185 | 8.0 | C |
| AT4G38900 | 4x | 4x | 189.87209155207353 | 6.0 | C |
| CYCB1;3 | 4x | - | 1565.412058409052 | 9.0 | C |
| RPOB | 4x | - | 669.6569959825002 | 13.0 | C |
| NRPB11 | 2x_4x | - | 8773.361503140284 | 19.0 | C |
| RAP74 | 4x | - | 552.9877897571595 | 15.0 | C |
| FER4 | 2x_4x | - | 8312.201038368219 | 5.0 | C |
| TT7 | 4x | 4x | 30834.32620392956 | 16.0 | B |
| AT4G22870 | 4x | - | 2.7499999999999996 | 9.0 | C |
| AT4G16030 | 2x_4x | - | 22.93320865511363 | 16.0 | C |
| AT5G16050 | 2x_4x | - | 5654.0 | 3.0 | B |
| F2P16.14 | 2x_4x | - | 6253.803670650051 | 4.0 | B |
| AT2G40780 | 2x_4x | 2x | 5733.901701045178 | 6.0 | C |
| FER1 | 2x_4x | 4x | 7.767420814479632 | 3.0 | B |
| AT1G10585 | 4x | - | 299.6812034844575 | 7.0 | C |
| NRPD2B | 4x | - | 669.6569959825002 | 15.0 | C |
| NRPB3 | 2x_4x | - | 7266.625946019145 | 17.0 | C |
| NRPD2A | 2x_4x | - | 2495.4232312629533 | 10.0 | B |
| NRPD1A | 2x_4x | - | 53062.46636573571 | 65.0 | HB |
| NRPB9B | 2x_4x | - | 2328.5365382590435 | 17.0 | C |
| NRPB2 | 2x_4x | - | 2180.3869508012967 | 12.0 | B |
| RBx1 | 2x_4x | - | 893.3138923920502 | 5.0 | C |
| FER2 | 2x_4x | - | 0.0 | 1.0 | C |
| NRPB9A | 2x_4x | - | 2491.5508999474737 | 12.0 | C |
| FER3 | 2x_4x | - | 2658.201038368341 | 4.0 | B |
| NRPE3B | 2x_4x | - | 2865.0261342684034 | 18.0 | B |
| CAK4 | 2x_4x | - | 1717.7507039450559 | 28.0 | B |
| CYCB2;2 | 4x | - | 1398.0350537084455 | 8.0 | C |
| UGT78D1 | 4x | - | 10313.334072351417 | 8.0 | B |
| NRPB4 | 2x_4x | - | 9972.240573542364 | 19.0 | B |
| SGR9 | 4x | 4x | 0.46153846153846156 | 14.0 | C |
| FLS4 | 4x | - | 2.916666666666666 | 7.0 | C |
| HEM15 | 4x | - | 0.0 | 4.0 | C |
| NRPB1 | 2x_4x | - | 65733.34564774274 | 74.0 | HB |
| CDKB1;2 | 4x | - | 7209.642786483611 | 15.0 | C |
| UGT78D2 | 4x | - | 2.7499999999999996 | 7.0 | C |
| AT5G01210 | 4x | 4x | 3544.0 | 2.0 | C |
| At2g30390 | 4x | - | 260.1854223260717 | 5.0 | C |
| KRP2 | 2x_4x | - | 5086.6325452996525 | 22.0 | C |
| AT5G26640 | 4x | - | 998.8204298279185 | 8.0 | C |
| NRPD1B | 2x_4x | - | 10851.846045163928 | 57.0 | HB |
| ORA47 | 4x | - | 0.0 | 2.0 | C |
| AT5G55220 | 2x_4x | 2x | 13432.337081980835 | 27.0 | B |
| FLS6 | 4x | - | 2.916666666666666 | 9.0 | C |
| NAM | 4x | - | 0.0 | 3.0 | C |
| SWEETIE | 4x | - | 657.3159929265216 | 2.0 | C |
| AT5G40590 | 4x | - | 0.0 | 3.0 | C |
| LACS2 | 4x | - | 0.0 | 2.0 | C |
| AO | 2x_4x | - | 1310.8184595859277 | 16.0 | B |
| TPL | 2x_4x | - | 12413.973814366824 | 7.0 | B |
| F7H12 | 2x_4x | - | 6916.299420357773 | 6.0 | B |
| ARI1 | 4x | 4x | 8.511904761904761 | 4.0 | C |
| AT1G52100 | 4x | - | 0.0 | 1.0 | C |
| CCoAOMT1 | 2x_4x | - | 0.015748031496062992 | 5.0 | C |
| 4CL3 | 2x_4x | - | 2201.3375417369757 | 7.0 | C |
| SDG21 | 4x | - | 146.1933734583923 | 19.0 | C |
| HTR12 | 4x | - | 6088.559782220518 | 89.0 | H |
| AT1G09200 | 4x | - | 107142.30716625052 | 92.0 | HB |
| SUVH1 | 4x | - | 6.701148860303319 | 17.0 | C |
| AGO9 | 4x | - | 0.0 | 10.0 |  |
| AGO7 | 4x | - | 0.0 | 10.0 |  |
| SUVH7 | 4x | - | 146.1933734583923 | 19.0 | C |
| ATxR2 | 4x | - | 0.0 | 4.0 | C |
| AT2G18720 | 4x | - | 0.5 | 5.0 | C |
| SDG14 | 2x_4x | - | 416.7749606090871 | 13.0 | C |
| AT3G27360.1 | 4x | - | 6573.963125008142 | 83.0 | H |
| AT3G61723 | 4x | - | 1.342555406812436 | 15.0 | C |
| MGH3 | 4x | - | 6088.559782220518 | 89.0 | H |
| SDG29 | 2x_4x | - | 603.717017180892 | 16.0 | C |
| AT5G10390.1 | 2x_4x | 2x | 537.0191685723703 | 15.0 | HB |
| SUVR2 | 4x | - | 6.701148860303319 | 17.0 | C |
| DDB1A | 4x | - | 7784.868565154226 | 51.0 | HB |
| SDG38 | 4x | - | 0.0 | 4.0 | C |
| TFL2 | 4x | 4x | 117865.11233203193 | 81.0 | HB |
| SWN | 4x | - | 290.07684184980735 | 18.0 | C |
| AT1G09200.1 | 2x_4x | - | 7633.411274770982 | 20.0 | HB |
| AGO10 | 4x | - | 0.0 | 10.0 |  |
| AT4G40030 | 2x_4x | - | 3948.771693835147 | 10.0 | HB |
| SDG16 | 2x_4x | - | 96.2376616964226 | 10.0 | C |
| SUVH4 | 4x | - | 146.1933734583923 | 19.0 | C |
| AT5G12910 | 4x | - | 6576.547523930808 | 91.0 | H |
| AT4G40040.1 | 4x | - | 6573.963125008142 | 83.0 | H |
| 4CL5 | 2x_4x | - | 3190.4479452369933 | 9.0 | C |
| C4H | 2x_4x | - | 1545.7884966884121 | 59.0 | H |
| SDG11 | 4x | - | 6.701148860303319 | 17.0 | C |
| AT4G35110 | 4x | - | 6.231182795698924 | 3.0 | C |
| MSI1 | 4x | - | 9042.320895729028 | 44.0 | B |
| SUVR4 | 2x_4x | - | 0.0 | 1.0 | C |
| MS1 | 4x | - | 1.342555406812436 | 15.0 | C |
| AT1G75600 | 4x | - | 6088.559782220518 | 89.0 | H |
| AT5G10980.1 | 4x | - | 6573.963125008142 | 83.0 | H |
| SUVR5 | 2x_4x | - | 19.280531436627182 | 3.0 | C |
| HTR11 | 4x | - | 6576.547523930808 | 91.0 | H |
| SUVH3 | 4x | - | 146.1933734583923 | 19.0 | C |
| AT5G65360.1 | 2x_4x | - | 53.260763009233905 | 13.0 | H |
| AT3G62500 | 4x | - | 1.342555406812436 | 15.0 | C |
| AT4G18330 | 4x | - | 0.5 | 5.0 | C |
| AT5G28340 | 4x | - | 1.342555406812436 | 15.0 | C |
| HCT | 2x_4x | - | 3610.190555130528 | 11.0 | C |
| ATCCR2 | 2x_4x | - | 7947.838635904064 | 129.0 | H |
| AT1G13370 | 4x | - | 6088.559782220518 | 89.0 | H |
| AT4G10600 | 4x | - | 1.342555406812436 | 15.0 | C |
| AT4G26220 | 2x_4x | - | 0.015748031496062992 | 5.0 | C |
| 4CL1 | 2x_4x | - | 2892.3787355319337 | 9.0 | C |
| SUVR1 | 4x | - | 6.701148860303319 | 17.0 | C |
| AGO6 | 4x | - | 0.0 | 10.0 |  |
| IMPA-2 | 2x_4x | - | 3134.6067827829856 | 14.0 | B |
| AT1G24735 | 2x_4x | - | 0.015748031496062992 | 5.0 | C |
| AT5G66220 | 4x | - | 2.8730158730158735 | 4.0 | C |
| DFR | 4x | - | 91.37653478356496 | 13.0 | C |
| ATxR5 | 4x | - | 0.0 | 3.0 | C |
| TT4 | 2x_4x | 4x | 129.90748529053528 | 16.0 | B |
| AGO5 | 4x | - | 0.0 | 10.0 |  |
| DDB1B | 4x | - | 7696.139114185352 | 50.0 | B |
| F3H | 4x | - | 91.37653478356496 | 15.0 | C |
| SUVH2 | 4x | - | 146.1933734583923 | 19.0 | C |
| SDG20 | 4x | - | 6.701148860303319 | 17.0 | C |
| AT5G21030 | 4x | - | 0.0 | 10.0 | C |
| AGO1 | 4x | - | 0.0 | 10.0 |  |
| RDR1 | 4x | - | 0.0 | 1.0 | C |
| AGO4 | 4x | - | 0.0 | 10.0 |  |
| AT3G52105 | 4x | - | 0.0 | 1.0 | C |
| ACOS5 | 2x_4x | - | 2777.974521371694 | 8.0 | C |
| SUVH5 | 4x | - | 146.1933734583923 | 19.0 | C |
| F20D22.6 | 4x | - | 0.5 | 5.0 | C |
| 4CL2 | 2x_4x | - | 3193.8408023798547 | 10.0 | C |
| FLS1 | 4x | - | 56.73003018381496 | 8.0 | C |
| SCR | 4x | - | 0.0 | 2.0 | C |
| SUVH6 | 4x | - | 146.1933734583923 | 19.0 | C |
| ASHH4 | 4x | - | 0.0 | 1.0 | C |
| AT2G19920 | 4x | - | 0.0 | 1.0 | C |
| SDG25 | 4x | - | 1.342555406812436 | 15.0 | C |
| CMT3 | 4x | - | 2.2105263157894726 | 9.0 | C |
| AGO3 | 4x | - | 0.0 | 10.0 |  |
| AT1G15950 | 2x_4x | - | 7947.838635904064 | 130.0 | H |
| AGO2 | 4x | - | 0.0 | 10.0 |  |
| FLS5 | 4x | - | 56.73003018381496 | 8.0 | C |
| SDG2 | 4x | - | 1.342555406812436 | 15.0 | C |
| HY5 | 4x | - | 0.0 | 2.0 | C |
| HYH | 4x | - | 0.0 | 2.0 | C |
| EMF2 | 4x | - | 0.0 | 3.0 | C |
| AT3G48550 | 4x | - | 0.0 | 2.0 | C |
| IMPA-6 | 4x | - | 0.0 | 2.0 | C |
| LIF2 | 2x_4x | - | 112.38381389179249 | 6.0 | C |
| RDR2 | 4x | - | 0.0 | 1.0 | C |
| RDR6 | 4x | - | 0.0 | 1.0 | C |
| AT4G05160 | 2x_4x | - | 83126.57818612413 | 182.0 | HB |
| FLS3 | 4x | - | 56.73003018381496 | 8.0 | C |
| AT2G19930 | 4x | - | 0.0 | 1.0 | C |
| CCOAMT | 2x_4x | - | 0.015748031496062992 | 5.0 | C |
| AT3G52100 | 4x | - | 0.0 | 1.0 | C |
| TT5 | 4x | - | 2.8730158730158735 | 4.0 | C |
| RING1 | 4x | - | 0.0 | 1.0 | C |
| RR14 | 4x | - | 0.0 | 2.0 | C |
| AT2G19910 | 4x | - | 0.0 | 1.0 | C |
| SDG4 | 4x | - | 0.0 | 1.0 | C |
| RIF10 | 2x_4x | - | 73.4038125167762 | 31.0 | C |
| ENOC | 2x_4x | - | 3000.872918280764 | 32.0 | B |
| LOS2 | 2x_4x | - | 8235.654627479274 | 42.0 | HB |
| AT2G25500 | 4x | - | 0.0 | 1.0 | C |
| AT5G10910 | 4x | - | 1.457142857142857 | 12.0 | C |
| MGD1 | 4x | - | 7.266666666666657 | 14.0 | C |
| LpxC4 | 4x | - | 0.8571428571428569 | 9.0 | C |
| AT1G18270 | 2x_4x | - | 2.0768890398462734 | 3.0 | HB |
| LpxC3 | 4x | - | 0.8571428571428569 | 9.0 | C |
| MURE | 4x | - | 8.123809523809511 | 17.0 | C |
| RPL27 | 4x | - | 0.5 | 3.0 | C |
| AT4G26860 | 4x | - | 2.4 | 7.0 | C |
| YLMG2 | 4x | - | 0.2857142857142857 | 5.0 | C |
| MGD2 | 4x | - | 7.266666666666657 | 14.0 | C |
| AT3G08840 | 4x | - | 8.123809523809511 | 17.0 | C |
| FAC1 | 4x | - | 0.0 | 3.0 | C |
| AT1G33360 | 4x | - | 0.0 | 3.0 | C |
| AT3G56900 | 4x | - | 0.0 | 1.0 | C |
| LpxC2 | 4x | - | 0.8571428571428569 | 9.0 | C |
| TRANS11 | 4x | - | 0.8571428571428569 | 10.0 | C |
| AT5G15220 | 4x | - | 0.5 | 3.0 | C |
| AT2G16930 | 4x | - | 0.5 | 3.0 | C |
| AT1G07615 | 4x | - | 1.9999999999999998 | 4.0 | C |
| MGDC | 4x | - | 7.266666666666657 | 14.0 | C |
| AT3G26560 | 2x_4x | - | 89021.31808298948 | 146.0 | HB |
| LpxC1 | 4x | - | 0.8571428571428569 | 9.0 | C |
| AT5G14580 | 2x_4x | - | 73.4038125167762 | 31.0 | C |
| ENO1 | 2x_4x | - | 534.2364998049519 | 37.0 | C |
| CCB3 | 4x | - | 0.2857142857142857 | 5.0 | C |
| LpxC5 | 4x | - | 0.8571428571428569 | 9.0 | C |
| AT5G49840 | 4x | - | 0.0 | 3.0 | C |
| EMB269 | 4x | - | 1.9999999999999998 | 4.0 | C |
| AT3G57220 | 4x | - | 0.8571428571428569 | 10.0 | C |
| YLMG1-2 | 4x | - | 0.2857142857142857 | 5.0 | C |
| CLPx | 4x | - | 0.0 | 3.0 | C |
| AT1G73740 | 4x | - | 7.266666666666657 | 14.0 | C |
| GPT | 4x | - | 0.8571428571428569 | 10.0 | C |
| YLMG1-1 | 4x | - | 0.2857142857142857 | 5.0 | C |
| AT1G11930 | 4x | - | 2.4 | 7.0 | C |
| AT4G04880 | 4x | - | 0.0 | 3.0 | C |
| PARC6 | 4x | - | 0.0 | 1.0 | C |
| AT5G52220 | 2x_4x | - | 722.8326578622346 | 16.0 | B |
| AT5G42770 | 4x | - | 0.0 | 1.0 | C |
| SAMBA | 4x | - | 0.0 | 1.0 | C |
| AT5G66550 | 4x | - | 0.0 | 1.0 | C |
| AT4G33865.1 | 4x | - | 103.52922871592529 | 155.0 | H |
| AT3G53870 | 2x_4x | - | 1120.6804558684664 | 56.0 | H |
| AT2G20450 | 2x_4x | - | 17599.357791631595 | 102.0 | HB |
| AT2G05220 | 2x_4x | - | 240.8153776740483 | 41.0 | H |
| AT2G41840 | 2x_4x | - | 10239.158889140102 | 63.0 | HB |
| SAC52 | 2x_4x | - | 150.34888834639213 | 23.0 | H |
| AT5G02960 | 2x_4x | - | 525.5881063571047 | 54.0 | H |
| AT2G40590 | 2x_4x | - | 63.98051622966612 | 23.0 | H |
| AT3G49910 | 2x_4x | - | 11392.265523007689 | 77.0 | HB |
| AT2G40010 | 2x_4x | - | 10417.67871204838 | 75.0 | HB |
| AT1G74060 | 2x_4x | - | 766.160640165093 | 55.0 | H |
| ATCG00380.1 | 4x | - | 150.37621482942527 | 170.0 | H |
| AT5G63070 | 2x_4x | - | 32.89147358923978 | 14.0 | H |
| AT3G09500 | 2x_4x | - | 6789.332199300963 | 43.0 | H |
| EMB2296 | 2x_4x | 4x | 1234.5005135274184 | 37.0 | H |
| UBQ3 | 2x_4x | - | 2779321.810408426 | 739.0 | HB |
| AT5G04800 | 2x_4x | - | 121.62127573281248 | 30.0 | H |
| RS27A | 2x_4x | - | 138.02384800408396 | 24.0 | H |
| AT5G09510 | 2x_4x | - | 639.002315501746 | 20.0 | H |
| AT3G10610 | 2x_4x | - | 452.3429913709538 | 38.0 | H |
| AT3G47370 | 2x_4x | 4x | 6043.9332715497385 | 60.0 | HB |
| AT3G11250 | 2x_4x | - | 20897.37287698144 | 98.0 | HB |
| AT5G35680 | 4x | - | 40.59852571831244 | 204.0 | H |
| AT2G34480 | 2x_4x | - | 1513.9825675512043 | 39.0 | H |
| FAB1A | 2x_4x | - | 5878.123879579761 | 12.0 | C |
| AT7SL-1 | 2x_4x | - | 85238.12826521977 | 430.0 | HB |
| AT5G39850 | 4x | - | 150.37621482942527 | 170.0 | H |
| AT5G52650 | 2x_4x | - | 244.2007982146763 | 29.0 | H |
| UBQ4 | 2x_4x | - | 87623.36404369946 | 433.0 | HB |
| MCCA | 4x | - | 1982.914276812817 | 31.0 | C |
| AT5G27700 | 2x_4x | - | 1785.878612174081 | 53.0 | H |
| AT4G34555 | 2x_4x | - | 209.50798328014395 | 31.0 | H |
| AT3G43980 | 4x | - | 103.52922871592529 | 155.0 | H |
| AT3G10330 | 2x_4x | - | 62.191477358122896 | 8.0 | C |
| TAF13 | 4x | - | 4.611548271927592 | 22.0 | C |
| AT4G39200 | 2x_4x | - | 1483.1219727920745 | 34.0 | H |
| AT4G03370 | 2x_4x | - | 57809.16508354996 | 438.0 | HB |
| GLN1.3 | 2x_4x | - | 53.745497538965786 | 42.0 | C |
| AT3G52580 | 2x_4x | - | 818.7882312171295 | 59.0 | HB |
| AT3G11510 | 2x_4x | - | 1230.1349142227166 | 62.0 | HB |
| AT3G43980.1 | 4x | - | 103.52922871592529 | 155.0 | H |
| UBQ7 | 2x_4x | - | 78211.06744904001 | 434.0 | HB |
| RPS5A | 2x_4x | - | 11533.888699359526 | 77.0 | HB |
| AT1G12960 | 2x_4x | - | 401.2269484949505 | 47.0 | H |
| AT2G21580 | 2x_4x | - | 143.08029477202865 | 37.0 | H |
| xW6 | 2x_4x | - | 3533.134623825418 | 53.0 | HB |
| AT3G53890 | 2x_4x | - | 1375.6965758275064 | 51.0 | H |
| AT3G18740 | 2x_4x | - | 1975.5456400403898 | 41.0 | H |
| AT5G16090 | 2x_4x | - | 10206.524030063345 | 236.0 | HB |
| RAD23C | 2x_4x | - | 8837.810149160347 | 229.0 | HB |
| BBC1 | 2x_4x | - | 2727.1930291240365 | 70.0 | H |
| AT5G59240 | 2x_4x | - | 1372.3863465968766 | 63.0 | HB |
| UBQ11 | 2x_4x | - | 57623.302462005726 | 426.0 | HB |
| AT3G25520 | 2x_4x | - | 1016.9377211241867 | 72.0 | HB |
| RPS13A | 2x_4x | - | 66233.70245722216 | 149.0 | HB |
| AT1G33850 | 4x | - | 662.6510207008764 | 231.0 | H |
| AT5G18380 | 2x_4x | - | 2341.525877217247 | 66.0 | H |
| GSA2 | 2x_4x | - | 309.0416459210548 | 11.0 | C |
| HEMA2 | 4x | - | 10.586856297053487 | 4.0 | C |
| RPL5B | 2x_4x | - | 1346.261284503755 | 68.0 | HB |
| AT2G17190 | 2x_4x | - | 9759.179216536648 | 235.0 | HB |
| AT1G67430 | 2x_4x | - | 62213.02551919917 | 120.0 | HB |
| AT3G53430 | 2x_4x | - | 767.7352121373523 | 73.0 | H |
| AT4G03360 | 2x_4x | - | 60602.00584782847 | 434.0 | HB |
| AT3G23145 | 2x_4x | - | 6.22447429480108 | 6.0 | C |
| UBQ13 | 2x_4x | - | 59536.3107239883 | 435.0 | HB |
| AT3G12915 | 2x_4x | - | 54776.582591492435 | 178.0 | HB |
| RPL18 | 2x_4x | - | 4377.923480416268 | 74.0 | H |
| KIN11 | 4x | - | 335.6843187575629 | 8.0 | C |
| AT5G61170 | 2x_4x | - | 673.5819015118127 | 58.0 | H |
| AT5G56710 | 2x_4x | - | 3213.776334689011 | 72.0 | H |
| RPS5B | 2x_4x | - | 21761.755558523106 | 93.0 | HB |
| AT2G32060 | 2x_4x | - | 76338.10991152017 | 166.0 | HB |
| AT3G14600 | 2x_4x | - | 420.0040239028433 | 41.0 | H |
| RPL18AA | 2x_4x | - | 31.02453425451082 | 16.0 | H |
| FAB1B | 2x_4x | - | 1860.4031126892576 | 11.0 | C |
| AT4G25740 | 2x_4x | - | 559.1317518417034 | 41.0 | H |
| AT3G51190 | 2x_4x | - | 231.76041650934238 | 37.0 | H |
| AT4G05270 | 2x_4x | - | 58947.383110115756 | 431.0 | HB |
| AT2G45710 | 2x_4x | - | 2346.075117670862 | 75.0 | HB |
| AT5G35530 | 2x_4x | - | 2034.2011009497014 | 62.0 | H |
| AT3G57490 | 2x_4x | - | 8680.459608918412 | 75.0 | HB |
| EMB1080 | 2x_4x | - | 728.6684636331796 | 49.0 | H |
| GDH1 | 2x_4x | - | 5229.444842467999 | 76.0 | HB |
| AT4G05240 | 2x_4x | - | 63530.110047886716 | 432.0 | HB |
| RPL23AB | 2x_4x | - | 659.8566453514649 | 34.0 | H |
| AT3G13120 | 2x_4x | - | 1567.876262507958 | 28.0 | HB |
| AT4G13170 | 2x_4x | - | 1263.0523639111068 | 52.0 | H |
| AT5G27850 | 2x_4x | - | 2152.5280929079386 | 52.0 | H |
| AT3G06455 | 2x_4x | - | 482772.907895711 | 518.0 | HB |
| BCCP2 | 4x | - | 663.5508008819205 | 53.0 | C |
| CAC3 | 4x | - | 1367.6328397674802 | 36.0 | C |
| AT1G74050 | 2x_4x | - | 1158.1301234004277 | 46.0 | H |
| AT4G34670 | 2x_4x | - | 1287.7081004548832 | 66.0 | H |
| AT3G09630 | 2x_4x | 2x | 15362.909861185213 | 97.0 | HB |
| HLL | 2x_4x | - | 10248.566880200382 | 58.0 | H |
| AT3G61111 | 2x_4x | - | 451.0505251786271 | 37.0 | H |
| AT2G26780 | 2x_4x | - | 480.8410902281605 | 91.0 | HB |
| AT3G07110 | 2x_4x | - | 555.9337320606176 | 52.0 | H |
| SAG24 | 2x_4x | - | 263.87428798645084 | 48.0 | H |
| AT4G05250 | 2x_4x | - | 63775.80227885937 | 437.0 | HB |
| AT2G37190 | 2x_4x | - | 511.52743326984853 | 49.0 | H |
| AT3G60245 | 2x_4x | - | 1760.2414463612542 | 62.0 | H |
| AT2G40510 | 2x_4x | - | 1306.4606079199953 | 21.0 | H |
| emb2386 | 2x_4x | - | 48.988619638415756 | 23.0 | H |
| AT5G09340 | 2x_4x | - | 60328.41705774903 | 424.0 | HB |
| GSR2 | 2x_4x | - | 53.414623144160856 | 38.0 | C |
| AT5G67510 | 2x_4x | - | 14672.41892025249 | 66.0 | HB |
| UBQ9 | 2x_4x | - | 65118.61362287296 | 428.0 | HB |
| OVA6 | 2x_4x | - | 155.2031111954163 | 13.0 | H |
| AT4G05230 | 2x_4x | - | 89144.82558481471 | 435.0 | HB |
| AT1G36240 | 2x_4x | - | 506.8198200108006 | 49.0 | H |
| RPS15AD | 2x_4x | - | 444.4457558517955 | 34.0 | H |
| AT1G15930 | 2x_4x | - | 28362.793106659417 | 113.0 | H |
| RAD23B | 2x_4x | - | 9571.71871525661 | 233.0 | HB |
| AT4G27090 | 2x_4x | - | 2216.0217979905165 | 69.0 | H |
| RPL10B | 2x_4x | - | 122.85324334658478 | 25.0 | H |
| AT3G45030 | 2x_4x | - | 256.9768006502008 | 25.0 | HB |
| AT1G70600 | 2x_4x | - | 144.1868737147584 | 39.0 | H |
| AT5G07090 | 2x_4x | - | 1161.4061807549638 | 43.0 | H |
| RPSAb | 2x_4x | - | 10041.75149763215 | 50.0 | HB |
| AT5G28060 | 2x_4x | - | 2226.8519171354988 | 73.0 | H |
| RPL23AA | 2x_4x | - | 532.4037650360366 | 28.0 | H |
| NFD3 | 4x | - | 366.2193446562762 | 148.0 | H |
| AT5G62300.1 | 2x_4x | - | 75.09469878747416 | 21.0 | HB |
| AT3G56340 | 2x_4x | - | 1004.7652258005519 | 46.0 | H |
| EVE1 | 2x_4x | - | 68337.94750545199 | 435.0 | HB |
| GAD4 | 2x_4x | 2x | 1694.866015023312 | 19.0 | C |
| AT1G77940 | 2x_4x | - | 1902.8497054917077 | 54.0 | H |
| AT3G04920 | 2x_4x | 2x | 6233.369577190423 | 64.0 | HB |
| AT4G05260 | 2x_4x | - | 83460.20805229654 | 438.0 | HB |
| F19P19.29 | 4x | - | 662.6510207008764 | 231.0 | H |
| At3g13445 | 2x_4x | - | 20019.422254149264 | 8.0 | HB |
| AT3G09360 | 2x_4x | - | 0.0 | 3.0 | C |
| ALDH12A1 | 2x_4x | 2x | 12.785858963865254 | 15.0 | C |
| AT1G80470 | 2x_4x | - | 264.67018490023776 | 18.0 | H |
| AT2G36160 | 2x_4x | - | 1269.0937689555076 | 70.0 | HB |
| AT2G09990 | 2x_4x | - | 1115.6745853241255 | 49.0 | H |
| AT4G05310 | 2x_4x | - | 57767.30860176769 | 437.0 | HB |
| AT5G16130 | 2x_4x | - | 1307.7059909118732 | 79.0 | H |
| AT5G23900 | 2x_4x | - | 1820.3882003194406 | 73.0 | H |
| AT4G30800 | 2x_4x | - | 11366.278928806903 | 98.0 | HB |
| RPS11-BETA | 2x_4x | - | 652.9602341999932 | 57.0 | H |
| AT3G58140 | 4x | - | 1723.6491061907152 | 57.0 | H |
| AT5G42220 | 2x_4x | - | 7537.865430558057 | 172.0 | HB |
| RPS18C | 4x | - | 141.14292191467362 | 161.0 | H |
| AT4G26230 | 2x_4x | - | 24775.555487412643 | 103.0 | HB |
| P40 | 2x_4x | - | 3684.850749784302 | 39.0 | HB |
| AT5G20290 | 2x_4x | 2x | 1880.0428219383073 | 64.0 | HB |
| AT1G18540 | 2x_4x | - | 581.3931226733575 | 46.0 | H |
| AT5G43640 | 2x_4x | - | 28.046033612471376 | 18.0 | H |
| UBQ6 | 2x_4x | - | 21468.5969723834 | 254.0 | HB |
| AT2G04390 | 2x_4x | - | 583.6748362448003 | 45.0 | H |
| AT3G48960 | 2x_4x | - | 2229.470050448837 | 56.0 | H |
| AT5G19720 | 2x_4x | - | 20.709055042522063 | 7.0 | C |
| AT4G26870 | 4x | - | 518.5933991716281 | 42.0 | C |
| AT1G34030 | 4x | - | 141.14292191467362 | 161.0 | H |
| AT5G58420 | 2x_4x | - | 565.4555499586806 | 59.0 | H |
| AAE18 | 2x_4x | - | 0.0 | 1.0 | C |
| ATKRS-1 | 2x_4x | - | 6042.487401952855 | 33.0 | HB |
| AT2G04520 | 4x | - | 40.59852571831244 | 204.0 | H |
| PFL | 4x | - | 141.14292191467362 | 161.0 | H |
| AT2G32350 | 2x_4x | - | 64286.68233656379 | 434.0 | HB |
| emb2171 | 2x_4x | - | 15852.522406789596 | 69.0 | H |
| AT5G46160 | 2x_4x | - | 12972.60529520542 | 62.0 | H |
| MIND | 4x | 4x | 3222.718013761551 | 6.0 | C |
| RNEE/G | 4x | - | 30698.286564677386 | 15.0 | B |
| AT5G48760 | 2x_4x | - | 2271.6264782180665 | 58.0 | H |
| RPL27AB | 2x_4x | - | 248.26701082759692 | 36.0 | H |
| RPS6A | 2x_4x | - | 3209.734023812632 | 58.0 | H |
| AT4G02230 | 2x_4x | - | 511.0535213524843 | 37.0 | HB |
| AT3G10950 | 2x_4x | - | 2188.2702606861894 | 70.0 | H |
| AT3G04840 | 2x_4x | - | 1042.8695478484615 | 69.0 | H |
| AT1G64880 | 2x_4x | - | 958.1167517547808 | 30.0 | HB |
| UBQ14 | 2x_4x | - | 56963.49394910289 | 437.0 | HB |
| AT3G09680 | 2x_4x | - | 800.5045629064086 | 61.0 | H |
| AT5G15520 | 2x_4x | - | 21346.278594725827 | 96.0 | HB |
| UBQ10 | 2x_4x | - | 57628.159064207925 | 433.0 | HB |
| ACC1 | 2x_4x | - | 19428.010926432664 | 115.0 | HB |
| GAD | 2x_4x | - | 2483.2527021367036 | 21.0 | C |
| GAD3 | 2x_4x | - | 2141.974915102507 | 20.0 | C |
| AT3G55170 | 2x_4x | - | 309.146304283991 | 37.0 | H |
| AT3G02560 | 2x_4x | - | 205.2034791467083 | 37.0 | H |
| AT2G39390 | 2x_4x | - | 32541.50371719808 | 73.0 | HB |
| AT2G17360 | 2x_4x | - | 1347.0885682694866 | 60.0 | H |
| pBRP2 | 2x_4x | - | 62.191477358122896 | 8.0 | C |
| AT2G19740 | 2x_4x | - | 278.20243120668107 | 51.0 | H |
| AT3G60770 | 2x_4x | - | 61913.71620229497 | 138.0 | HB |
| At2g34520 | 4x | - | 3.2328391184117318 | 130.0 | H |
| AT3G57370 | 2x_4x | - | 62.191477358122896 | 8.0 | C |
| TAFII59 | 2x_4x | 2x | 1044.338390060989 | 13.0 | C |
| RPS11 | 4x | - | 366.2193446562762 | 148.0 | H |
| RAD23D | 2x_4x | 2x | 156438.27043607648 | 268.0 | HB |
| RPS28 | 2x_4x | - | 5912.877833962218 | 78.0 | HB |
| RPS10B | 2x_4x | - | 273.0981452766132 | 36.0 | H |
| AT1G27400 | 2x_4x | - | 34727.904812760506 | 89.0 | H |
| AT3G30805 | 2x_4x | - | 150.56612124681573 | 11.0 | C |
| AT5G09500 | 2x_4x | - | 80.51344425928939 | 14.0 | H |
| AT5G26710 | 2x_4x | - | 395.7765506883101 | 16.0 | HB |
| AT1G09620 | 2x_4x | - | 413.4275926161775 | 10.0 | C |
| AT5G47930 | 2x_4x | - | 190.81093347687587 | 30.0 | H |
| HD1 | 4x | - | 7400.596227023836 | 11.0 | B |
| SAP18 | 4x | - | 11233.186960645455 | 11.0 | B |
| AT5G09490 | 2x_4x | - | 38.00512563942123 | 13.0 | H |
| AT3G09200 | 2x_4x | - | 38226.439464084215 | 105.0 | HB |
| AT1G04480 | 2x_4x | - | 14732.647754269543 | 68.0 | H |
| ERS | 4x | 4x | 5362.974674828455 | 39.0 | C |
| GDH2 | 2x_4x | - | 7004.128301419322 | 79.0 | HB |
| GS2 | 2x_4x | - | 21109.755688522313 | 114.0 | HB |
| HAF2 | 4x | - | 8567.067411693355 | 51.0 | HB |
| AT4G16720 | 2x_4x | - | 2658.5663850756155 | 78.0 | H |
| AT5G02870 | 2x_4x | - | 13043.555662175175 | 89.0 | HB |
| AT1G48830 | 2x_4x | - | 541.217093982694 | 35.0 | H |
| RPL3B | 2x_4x | 2x | 1590.7907180298273 | 80.0 | H |
| EMB3010 | 2x_4x | - | 1353.2923402465985 | 56.0 | H |
| FTSZ1-1 | 4x | - | 43736.554347030265 | 34.0 | B |
| FTSZ2-2 | 4x | - | 36195.111558841534 | 32.0 | B |
| GLU1 | 2x_4x | - | 107730.8120479147 | 199.0 | HB |
| AT3G02080 | 2x_4x | - | 323.1488030804596 | 46.0 | H |
| AT5G60670 | 2x_4x | - | 12482.619547354398 | 135.0 | HB |
| AT2G31610 | 2x_4x | - | 1127.8661942342824 | 34.0 | HB |
| AT4G31180 | 2x_4x | 2x | 197.9109640278189 | 9.0 | C |
| UBQ8 | 2x_4x | - | 63804.60246922877 | 432.0 | HB |
| AT2G39590 | 2x_4x | - | 160.75853273927692 | 28.0 | H |
| GDH3 | 2x_4x | - | 5769.109816223879 | 78.0 | HB |
| TAF5 | 2x_4x | - | 102.074689969228 | 8.0 | HB |
| AT4G17390 | 2x_4x | - | 1357.0507547366717 | 69.0 | H |
| AT5G15200 | 4x | - | 150.37621482942527 | 170.0 | H |
| ACC2 | 2x_4x | - | 54558.72306898373 | 117.0 | HB |
| LOS1 | 2x_4x | - | 90126.59196924124 | 182.0 | HB |
| RPL16A | 2x_4x | - | 4547.094333067062 | 96.0 | HB |
| AT1G29965 | 2x_4x | - | 103.95795665000664 | 21.0 | H |
| TAF6B | 2x_4x | - | 1687.7718760137984 | 9.0 | C |
| TAF4B | 4x | - | 0.0 | 17.0 | C |
| AT2G04540 | 2x_4x | - | 678.857307231859 | 53.0 | H |
| OVA2 | 4x | - | 158.9078258205959 | 21.0 | C |
| rps14 | 4x | - | 3.2328391184117318 | 130.0 | H |
| At1g43170 | 2x_4x | - | 1211.2579153025497 | 60.0 | H |
| FAB1D | 2x_4x | - | 1521.9717440637876 | 14.0 | C |
| CAC1 | 4x | - | 663.5508008819205 | 53.0 | C |
| AT1G51720 | 2x_4x | - | 4596.748662683522 | 81.0 | HB |
| AT3G04230 | 2x_4x | - | 212.9432412609517 | 42.0 | H |
| AT3G62120 | 2x_4x | - | 191.70811435117471 | 11.0 | C |
| AT3G59980 | 2x_4x | - | 8643.210199898276 | 20.0 | H |
| AT4G35540 | 2x_4x | - | 0.0 | 3.0 | C |
| HAF01 | 2x_4x | - | 3933.589273137515 | 13.0 | HB |
| AT4G36130 | 2x_4x | - | 460.0321503807121 | 41.0 | H |
| NodGS | 2x_4x | - | 2011.4118668764384 | 34.0 | C |
| FAB1C | 2x_4x | - | 17156.104566116333 | 18.0 | HB |
| AT5G47720 | 4x | - | 256.2770237528868 | 35.0 | C |
| HOS15 | 4x | 4x | 3597.7633140113517 | 8.0 | C |
| HDA6 | 4x | - | 19591.94736959777 | 75.0 | HB |
| EMB3137 | 4x | - | 141.14292191467362 | 161.0 | H |
| GAD5 | 2x_4x | - | 1884.90350709605 | 20.0 | C |
| AAE17 | 2x_4x | - | 0.0 | 1.0 | C |
| GLU2 | 2x_4x | - | 50075.35263314873 | 136.0 | HB |
| AT3G24830 | 2x_4x | 4x | 1010.8252673216865 | 64.0 | H |
| AKINBETA1 | 2x_4x | - | 71.10755210781993 | 6.0 | C |
| ACS | 2x_4x | - | 4161.984682412028 | 9.0 | B |
| AT1G04480.1 | 2x_4x | - | 17320.132744228147 | 71.0 | H |
| AT4G02950 | 2x_4x | - | 62429.8254419384 | 429.0 | HB |
| RPL14 | 2x_4x | - | 15876.033359708013 | 72.0 | H |
| GLT1 | 2x_4x | - | 139570.61488504184 | 183.0 | HB |
| AT3G16780 | 2x_4x | - | 289.09591123899025 | 34.0 | HB |
| AT1G77750 | 4x | - | 141.14292191467362 | 161.0 | H |
| CAC2 | 2x_4x | - | 56.44033775450085 | 3.0 | C |
| HCS1 | 4x | - | 0.2312312312312312 | 6.0 | C |
| ARC6 | 4x | - | 2.0 | 4.0 | C |
| ARC3 | 4x | - | 4559.177290289152 | 6.0 | C |
| TBP2 | 2x_4x | - | 39788.37302622512 | 10.0 | HB |
| cICDH | 2x_4x | - | 343.4041402595043 | 17.0 | H |
| AT4G10320 | 2x_4x | - | 1589.2444501168077 | 15.0 | H |
| PSD | 4x | - | 24.600239864126998 | 26.0 | C |
| TAFII21 | 2x_4x | - | 1506.6182487961808 | 10.0 | B |
| AT4G39280 | 4x | - | 24.645694409581544 | 29.0 | C |
| AT5G10880 | 2x_4x | - | 17.350129274196266 | 7.0 | C |
| ACCD | 4x | - | 1562.8281269953466 | 37.0 | C |
| AT2G45100 | 2x_4x | - | 0.0 | 3.0 | C |
| SNF4 | 4x | - | 5828.098585771764 | 64.0 | H |
| EER4 | 2x_4x | - | 1776.6189752716273 | 10.0 | C |
| GAD2 | 2x_4x | - | 2148.0223942908992 | 20.0 | C |
| OVA9 | 2x_4x | - | 46.27262121529705 | 11.0 | H |
| AT4G13780 | 2x_4x | - | 819.1317834514556 | 22.0 | HB |
| GLN1;5 | 2x_4x | - | 53.745497538965786 | 42.0 | C |
| AT5G14590 | 2x_4x | - | 1.585014985014985 | 16.0 | C |
| ICDH | 2x_4x | - | 343.4041402595043 | 17.0 | C |
| AT4G10680 | 2x_4x | - | 62.191477358122896 | 8.0 | C |
| KASI | 2x_4x | - | 678.857307231859 | 53.0 | H |
| PBRP | 2x_4x | - | 0.0 | 3.0 | C |
| TAF11 | 4x | - | 9.99463714709235 | 23.0 | C |
| GLN1;4 | 2x_4x | - | 53.745497538965786 | 42.0 | C |
| GLN1-1 | 2x_4x | - | 53.745497538965786 | 42.0 | C |
| OVA5 | 2x_4x | - | 68.56165321221114 | 9.0 | C |
| PGR7 | 4x | - | 0.0 | 1.0 | C |
| HEMA1 | 4x | - | 7096.586856297052 | 6.0 | B |
| AT3G52250 | 4x | - | 54.906171154222946 | 9.0 | C |
| hda17 | 4x | - | 33.27202915629431 | 8.0 | C |
| TAF4 | 4x | - | 0.0 | 17.0 | C |
| TAFII15 | 2x_4x | - | 104.42687257778397 | 10.0 | C |
| AT1G69800 | 4x | - | 0.0 | 5.0 | C |
| CDPK6 | 4x | - | 0.0 | 2.0 | C |
| TAF2 | 4x | - | 4196.2912988454145 | 40.0 | B |
| TFIIB | 2x_4x | - | 1029.711079093261 | 9.0 | C |
| HDA7 | 4x | - | 33.27202915629431 | 10.0 | C |
| FVE | 4x | - | 58.53579264384448 | 11.0 | C |
| emb1027 | 4x | - | 1.5139645977272347 | 19.0 | C |
| TAF7 | 2x_4x | - | 4876.9177554231255 | 9.0 | C |
| RBR1 | 2x_4x | - | 7.208705621863507 | 17.0 | B |
| MEE65 | 2x_4x | - | 0.0 | 3.0 | C |
| AT4G16360 | 2x_4x | - | 71.10755210781993 | 6.0 | C |
| T21L8.140 | 2x_4x | - | 2851.8610326447633 | 32.0 | C |
| TAF11b | 4x | - | 9.99463714709235 | 23.0 | C |
| PYRB | 2x_4x | - | 29840.899826622604 | 35.0 | B |
| NOA1 | 4x | - | 88204.42740007251 | 95.0 | HB |
| GCT | 4x | - | 462.48570513650554 | 27.0 | C |
| NFC5 | 4x | - | 58.53579264384448 | 11.0 | C |
| KCO1 | 4x | 4x | 3.4602941176470585 | 4.0 | C |
| hda10 | 4x | - | 33.27202915629431 | 8.0 | C |
| AT5G60335 | 4x | - | 23.792036142398878 | 13.0 | C |
| AT1G66530 | 4x | - | 1.5139645977272347 | 19.0 | C |
| KIN10 | 4x | - | 335.6843187575629 | 8.0 | C |
| EMB3147 | 4x | - | 461.1631870502731 | 17.0 | C |
| FTSZ2-1 | 4x | - | 36650.55434702322 | 32.0 | B |
| SnRK1.3 | 4x | - | 0.0 | 7.0 | C |
| TAF12 | 2x_4x | - | 367.4328249732399 | 6.0 | C |
| AT2G40660 | 2x_4x | - | 6014.095717034159 | 22.0 | HB |
| AT5G03830 | 2x_4x | - | 9.136072612362048 | 14.0 | C |
| AAK6 | 4x | 4x | 7103.291991816228 | 7.0 | B |
| AT4G09000.2 | 2x_4x | - | 5738.364990744088 | 5.0 | B |
| AT2G44510 | 2x_4x | - | 9.136072612362048 | 14.0 | C |
| FAB1 | 2x_4x | - | 678.857307231859 | 53.0 | H |
| AT1G62640 | 2x_4x | - | 112.42112822877739 | 21.0 | C |
| FIE | 4x | - | 53.763314011365786 | 9.0 | C |
| ECH2 | 4x | - | 23.792036142398878 | 13.0 | C |
| GSA1 | 4x | - | 1551.7207331229206 | 4.0 | C |
| HEMA3 | 4x | - | 10.586856297053487 | 4.0 | C |
| HDA9 | 4x | - | 33.27202915629431 | 8.0 | C |
| SNL1 | 4x | - | 66.24765929854281 | 11.0 | C |
| GLY1 | 2x_4x | - | 0.0 | 3.0 | C |
| PEx1 | 4x | - | 0.0 | 1.0 | C |
| PEx6 | 4x | - | 3544.0 | 2.0 | C |
| ACAT2 | 4x | - | 3814.327611429506 | 36.0 | C |
| MINE1 | 4x | - | 0.0 | 2.0 | C |
| GPDHp | 2x_4x | - | 0.0 | 3.0 | C |
| HCS2 | 4x | - | 0.2312312312312312 | 6.0 | C |
| ERS1 | 4x | - | 0.0 | 2.0 | C |
| ETR1 | 2x_4x | - | 4.608584504740107 | 10.0 | C |
| AT3G07690 | 2x_4x | - | 0.0 | 3.0 | C |
| GPDHC1 | 2x_4x | - | 0.0 | 3.0 | C |
| CTR1 | 4x | - | 7404.147680121679 | 6.0 | C |
| AT1G30070 | 4x | 2x | 0.0 | 1.0 | C |
| ETR2 | 2x_4x | - | 4.386362282517885 | 9.0 | C |
| F12B17_200 | 4x | - | 2.261790310229195 | 3.0 | C |
| CML37 | 4x | 4x | 10.629205925999996 | 2.0 | C |
| NLM1 | 2x_4x | - | 360.85315652862494 | 7.0 | C |
| FLU | 4x | - | 0.0 | 1.0 | C |
| VTE1 | 4x | 2x | 0.0 | 2.0 | C |
| APG1 | 4x | - | 4639.867774380376 | 4.0 | C |
| NUB1 | 4x | - | 0.025974025974025976 | 3.0 | C |
| AT1G31910 | 4x | - | 880.3280622298482 | 5.0 | C |
| MENG | 4x | - | 9179.000626417937 | 3.0 | C |
| AT3G24200 | 4x | - | 46.43635750840518 | 2.0 | C |
| AT3G21060 | 2x_4x | - | 0.0 | 1.0 | C |
| TIM50 | 2x_4x | - | 4098.778373359051 | 14.0 | B |
| PDS1 | 4x | 2x | 0.0625 | 2.0 | C |
| AOAT2 | 2x_4x | - | 666.2394496289644 | 24.0 | C |
| ICL | 4x | 2x | 1983.492889379527 | 8.0 | C |
| CAD2 | 2x_4x | - | 7468.899493023753 | 95.0 | H |
| CAD3 | 2x_4x | - | 7565.770917602696 | 96.0 | H |
| MLS | 2x_4x | - | 173.30338298208486 | 17.0 | C |
| ERA1 | 4x | - | 2.752747252747252 | 26.0 | C |
| AGT | 2x_4x | - | 8209.611114866913 | 64.0 | HB |
| AT5G36790 | 2x_4x | - | 224.73944399097 | 9.0 | C |
| CAD9 | 2x_4x | - | 32486.808897057428 | 101.0 | HB |
| ELI3-2 | 2x_4x | - | 8931.112244769018 | 97.0 | HB |
| OMT1 | 2x_4x | 2x | 84676.4159011901 | 100.0 | HB |
| CYP735A1 | 2x_4x | - | 1179.145169617838 | 98.0 | HB |
| SQS2 | 2x_4x | - | 2654.1915149664837 | 153.0 | HB |
| AT1G50670 | 4x | - | 14.064397506020986 | 15.0 | C |
| APM1 | 4x | - | 42.49132996968116 | 61.0 | H |
| AT5G62910 | 4x | - | 3.312687705218831 | 31.0 | C |
| ATCAD4 | 2x_4x | - | 60099.19135364733 | 102.0 | HB |
| SQS1 | 2x_4x | - | 2654.1915149664837 | 153.0 | HB |
| AT4G35090 | 4x | - | 512.807959903503 | 5.0 | C |
| CAD6 | 2x_4x | - | 7468.899493023753 | 95.0 | H |
| CYP84A4 | 2x_4x | - | 4309.5113067731645 | 62.0 | H |
| FTA | 4x | - | 7464.037719847241 | 27.0 | C |
| HSP70 | 2x_4x | 2x | 4296.747819958552 | 38.0 | H |
| CHIP | 2x_4x | - | 4840.327860890595 | 23.0 | B |
| F7H19.150 | 4x | - | 396.3967127119641 | 85.0 | H |
| PAB4 | 2x_4x | - | 165.28216121465934 | 20.0 | H |
| AT4G36960 | 2x_4x | - | 226.8739906529671 | 4.0 | H |
| Hsp70-2 | 2x_4x | - | 1491.315860970672 | 37.0 | H |
| AT3G09440 | 2x_4x | - | 855.2821909082315 | 37.0 | H |
| CAD5 | 2x_4x | - | 101353.01481248303 | 101.0 | HB |
| PAB2 | 2x_4x | - | 53.48753439210697 | 19.0 | H |
| ORC1A | 4x | - | 0.32553990066355387 | 66.0 | H |
| RAD23A | 2x_4x | - | 9038.615349773121 | 231.0 | HB |
| AT1G74470 | 2x_4x | - | 28397.03540945109 | 55.0 | HB |
| AT1G74870 | 4x | - | 3.312687705218831 | 31.0 | C |
| HDS | 4x | - | 3965.4169837463037 | 11.0 | C |
| AT5G15400 | 4x | - | 227.42356624110315 | 33.0 | C |
| AT2G28540 | 4x | - | 3.312687705218831 | 31.0 | C |
| AT5G60170 | 4x | - | 3.312687705218831 | 31.0 | C |
| HUB2 | 4x | - | 193.10548061141938 | 17.0 | C |
| fah1 | 2x_4x | 2x | 124037.4016824806 | 145.0 | HB |
| CDC6 | 2x_4x | - | 36320.9034439335 | 103.0 | HB |
| AT3G45630 | 4x | - | 3.312687705218831 | 31.0 | C |
| HUB1 | 4x | - | 13.415409116385034 | 17.0 | C |
| UBQ1 | 2x_4x | 2x | 11340.434391980452 | 19.0 | B |
| ORC1B | 4x | - | 0.32553990066355387 | 66.0 | H |
| HSC70-1 | 2x_4x | - | 4489.324336589959 | 40.0 | H |
| Hsp70b | 2x_4x | - | 427.36171481934537 | 35.0 | H |
| AT3G48070 | 4x | - | 3.312687705218831 | 31.0 | C |
| TPP2 | 4x | - | 58.26824707698539 | 61.0 | H |
| HSP70-18 | 2x_4x | - | 427.36171481934537 | 35.0 | H |
| AG | 2x | 2x | 0.0 | 1.0 | C |
| TPS5 | 2x | - | 1859.963139434834 | 3.0 | C |
| RAD4 | 2x | - | 0.0 | 1.0 | C |
| TRE1 | 2x | - | 52.96420361247949 | 4.0 | C |
| TPS4 | 2x | - | 5.566666666666666 | 2.0 | C |
| T13D8.4 | 2x | - | 80.46952883717596 | 4.0 | C |
| ATRPAC42 | 2x | - | 35.46828629074225 | 6.0 | C |
| ATTPS6 | 2x | 2x | 3696.9262788696765 | 3.0 | C |
| NRPA2 | 2x | - | 46.73013205648626 | 6.0 | C |
| TPS7 | 2x | - | 28990.179346208108 | 4.0 | B |
| TPS11 | 2x | - | 3935.7116458732135 | 4.0 | C |
| TPS2 | 2x | - | 7456.698935821824 | 3.0 | B |
| TPS1 | 2x | - | 0.0 | 2.0 | C |
| PSAH2 | 2x | 2x | 1013.6827008670739 | 98.0 | H |
| PSI-P | 2x | - | 6695.098962377935 | 130.0 | H |
| PTB1 | 2x | 2x | 853.4158417207749 | 11.0 | C |
| ALDH7B4 | 2x | 2x | 0.0 | 1.0 | C |
| AT5G42250 | 2x | 2x | 5661.324872422843 | 15.0 | C |
| AT1G01210 | 2x | - | 335.04831876198676 | 11.0 | C |
| NRPB6B | 2x | - | 10623.034207898394 | 21.0 | C |
| RPAC43 | 2x | - | 285.3249591308783 | 8.0 | C |
| AT1G75510 | 2x | - | 6314.964439262918 | 9.0 | C |
| NRPB8B | 2x | - | 1332.1315124089597 | 16.0 | C |
| NRPB10 | 2x | - | 1334.7127357803115 | 13.0 | C |
| AT1G61700 | 2x | - | 4773.294873455922 | 16.0 | C |
| TPS3 | 2x | - | 14833.402456611022 | 5.0 | B |
| NRPB5 | 2x | - | 13205.759105474095 | 20.0 | B |
| NRPC2 | 2x | 2x | 82.91800655502442 | 6.0 | C |
| NDPK3 | 2x | 2x | 33.2818478547792 | 5.0 | C |
| TPS9 | 2x | - | 14171.083045907793 | 5.0 | B |
| TPS8 | 2x | - | 10268.74614304792 | 9.0 | B |
| RPB5C | 2x | - | 61.26742189089376 | 8.0 | C |
| AT1G53690 | 2x | - | 4542.828797342936 | 17.0 | C |
| AT3G52270 | 2x | - | 2690.4380952323863 | 17.0 | C |
| NRPB6A | 2x | - | 17564.384353550635 | 29.0 | B |
| AT4G07950 | 2x | - | 1658.0159435418896 | 10.0 | C |
| NRPB8A | 2x | - | 2600.638107975579 | 13.0 | C |
| EIF3A | 2x | - | 13737.008092569473 | 87.0 | H |
| NRPB12 | 2x | - | 19138.061280717273 | 41.0 | C |
| PFK6 | 2x | - | 481.3901155057141 | 19.0 | C |
| SNRK2.10 | 2x | - | 0.0 | 1.0 | C |
| SHM2 | 2x | - | 0.0 | 1.0 | C |
| ADH1 | 2x | 2x | 7.324872422851775 | 15.0 | C |
| PDC3 | 2x | - | 5578.948356737594 | 38.0 | C |
| PFK4 | 2x | - | 3175.6986898166188 | 19.0 | C |
| FBP | 2x | 2x | 7556.3118516901395 | 19.0 | B |
| LST8-2 | 2x | - | 0.0 | 1.0 | C |
| HPA1 | 2x | - | 691.7058216611819 | 10.0 | C |
| AT1G31690 | 2x | - | 335.83450831245676 | 15.0 | C |
| SK10 | 2x | - | 0.0 | 1.0 | C |
| ASN3 | 2x | - | 976.5834702578314 | 40.0 | C |
| GLx1 | 2x | - | 0.0 | 1.0 | C |
| AT2G42490 | 2x | - | 5654.0 | 14.0 | C |
| AT1G12000 | 2x | - | 0.0 | 1.0 | C |
| ALDH3I1 | 2x | - | 0.0 | 1.0 | C |
| PDC2 | 2x | - | 16667.321233088463 | 42.0 | B |
| AT5G03370 | 2x | - | 69.85861409463 | 3.0 | C |
| NIT1 | 2x | - | 31088.5752522978 | 25.0 | B |
| AT4G33070 | 2x | - | 8517.173139741788 | 38.0 | C |
| F2KP | 2x | - | 653.4631890747798 | 10.0 | C |
| AT4G28410 | 2x | - | 350.91297885388724 | 10.0 | C |
| AT4G28420 | 2x | - | 22.25279636936083 | 8.0 | C |
| AT4G12780 | 2x | 2x | 0.0 | 1.0 | C |
| AT2G45290 | 2x | 2x | 11738.83977203991 | 49.0 | B |
| SHM4 | 2x | - | 0.0 | 1.0 | C |
| AT1G32470 | 2x | 2x | 41187.58501969522 | 146.0 | HB |
| AT3G29010 | 2x | - | 0.0 | 1.0 | C |
| NIT2 | 2x | - | 6185.379579303675 | 27.0 | C |
| YUC11 | 2x | - | 0.4778977542135438 | 4.0 | C |
| NIT3 | 2x | - | 4725.22232960298 | 24.0 | C |
| AT1G12230 | 2x | - | 8082.682575225983 | 43.0 | C |
| AT5G01320 | 2x | - | 7218.606209324082 | 43.0 | C |
| SNRK2.3 | 2x | - | 390.5235493799751 | 5.0 | C |
| PFK3 | 2x | - | 3785.119609979327 | 16.0 | C |
| SHM6 | 2x | - | 11306.0 | 3.0 | B |
| DIN9 | 2x | - | 664.4692386341471 | 8.0 | C |
| PFK5 | 2x | - | 10723.260821493268 | 26.0 | B |
| HKL3 | 2x | - | 5672.337994614724 | 35.0 | C |
| TOR | 2x | - | 0.0 | 1.0 | C |
| MEE51 | 2x | - | 44.16926845845718 | 3.0 | C |
| AT4G08876 | 2x | - | 0.0 | 1.0 | C |
| AT5G24760 | 2x | - | 159.73032929122914 | 15.0 | C |
| MEE31 | 2x | - | 715.6586852766626 | 8.0 | C |
| AT1G31670 | 2x | 2x | 87.10473975077572 | 14.0 | C |
| TAT3 | 2x | - | 1034.2437426818267 | 8.0 | C |
| AT1G06020 | 2x | - | 2097.406072103216 | 13.0 | C |
| HKL1 | 2x | - | 291.7703668922424 | 14.0 | C |
| RLP7 | 2x | 2x | 0.0 | 1.0 | C |
| CuAO1 | 2x | - | 22213.06799396462 | 15.0 | B |
| SNRK2.2 | 2x | - | 6044.523549379955 | 6.0 | C |
| ADSS | 2x | - | 1.36778386758356 | 2.0 | C |
| AT3G43670 | 2x | - | 118.18039594970371 | 14.0 | C |
| PAO1 | 2x | - | 0.0 | 13.0 | C |
| ALDH2B7 | 2x | - | 0.0 | 1.0 | C |
| YUC10 | 2x | - | 5654.477897754215 | 5.0 | C |
| SK4 | 2x | - | 0.0 | 1.0 | C |
| SK21 | 2x | 2x | 5787.884205123038 | 10.0 | C |
| EIF3E | 2x | - | 0.0 | 1.0 | C |
| ALDH3H1 | 2x | 2x | 1538.0829240188607 | 3.0 | C |
| AT1G06030 | 2x | - | 485.5836804447246 | 7.0 | C |
| PFK7 | 2x | - | 3412.470317650654 | 21.0 | C |
| HxK1 | 2x | - | 84768.06627094463 | 36.0 | B |
| AT5G42740 | 2x | - | 63718.96087116544 | 97.0 | HB |
| ALDH2B4 | 2x | - | 132.0370148161471 | 4.0 | C |
| ExL2 | 2x | 2x | 0.0 | 1.0 | C |
| ERD15 | 2x | - | 5654.0 | 2.0 | C |
| ATG8D | 2x | - | 5713.10653987948 | 6.0 | B |
| AT5G62200 | 2x | 2x | 0.0 | 1.0 | C |
| AT2G46560 | 2x | - | 43.193354509077174 | 15.0 | C |
| eIFiso4G1 | 2x | - | 560.4778607813491 | 4.0 | C |
| Hsp70-15 | 2x | - | 32910.68442772078 | 68.0 | HB |
| GATB | 2x | - | 479.94216949983445 | 2.0 | C |
| IAA7 | 2x | - | 661.5878877056588 | 5.0 | C |
| AT1G80440 | 2x | - | 17117.26945462443 | 12.0 | B |
| AT5G07580 | 2x | - | 0.0 | 1.0 | C |
| FAAH | 2x | 2x | 140.20872549717737 | 3.0 | C |
| IAA5 | 2x | - | 640.8569765417376 | 4.0 | C |
| AT3G58730 | 2x | - | 2502.091612944198 | 38.0 | C |
| NAC032 | 2x | - | 0.0 | 1.0 | C |
| J8 | 2x | - | 1842.5216905957118 | 10.0 | C |
| AT4G05070 | 2x | - | 1094.724037026373 | 6.0 | C |
| IAA18 | 2x | - | 596.6193802497196 | 5.0 | C |
| AT5G66230 | 2x | 2x | 18.042324010805036 | 11.0 | C |
| ARF10 | 2x | - | 1953.9074835242354 | 5.0 | C |
| AT5G61590 | 2x | 2x | 1347.620467174298 | 5.0 | C |
| BZO2H3 | 2x | - | 351.6889805493795 | 4.0 | C |
| ARF12 | 2x | - | 2.0666666666666664 | 5.0 | C |
| IAA8 | 2x | - | 49.772612869699074 | 3.0 | C |
| ECT3 | 2x | - | 25.672160138371638 | 9.0 | C |
| AT5G08520 | 2x | - | 51.196947174814966 | 5.0 | C |
| RPS7.2 | 2x | - | 23036.992950095097 | 46.0 | C |
| AT1G59750 | 2x | - | 43563.16301713424 | 9.0 | B |
| CYCB1;4 | 2x | - | 7574.747708409384 | 11.0 | C |
| AT1G76810 | 2x | - | 18406.851066667263 | 101.0 | H |
| RPS7 | 2x | - | 24139.0616429615 | 45.0 | B |
| AT5G61820 | 2x | 2x | 0.0 | 1.0 | C |
| HIK | 2x | - | 79.89906757075775 | 12.0 | C |
| CYCB2;4 | 2x | - | 32407.63461751677 | 40.0 | B |
| ENODL14 | 2x | - | 4411.050221112195 | 18.0 | C |
| AT1G07930 | 2x | - | 52413.99255148271 | 11.0 | B |
| IAA19 | 2x | - | 451.6740387837321 | 9.0 | C |
| EMB3126 | 2x | - | 24319.09752340575 | 53.0 | H |
| ROC2 | 2x | - | 3942.0186389671603 | 27.0 | C |
| AT1G21160 | 2x | - | 19232.95932006923 | 109.0 | H |
| AT1G29250 | 2x | - | 54.37026738635611 | 23.0 | C |
| AT3G15450 | 2x | - | 7216.693604598536 | 10.0 | B |
| AT5G19120 | 2x | - | 16.93713901654852 | 7.0 | C |
| AT5G45700 | 2x | - | 8225.519469764853 | 13.0 | C |
| MP | 2x | - | 8.166666666666664 | 7.0 | C |
| RPL33 | 2x | - | 294.36131345098335 | 16.0 | C |
| AT5G16250 | 2x | - | 115.21029382563694 | 17.0 | C |
| AT1G23390 | 2x | - | 8.798250127659626 | 5.0 | C |
| AT3G15630 | 2x | - | 2417.585071339397 | 5.0 | C |
| AT2G42710 | 2x | - | 90.86483485715185 | 25.0 | C |
| AT5G22920 | 2x | - | 10832.344698709081 | 8.0 | B |
| AT3G03130 | 2x | - | 1751.4305948520637 | 16.0 | C |
| AT3G51280 | 2x | - | 1937.154982816843 | 13.0 | C |
| PRH75 | 2x | - | 971.5298521907357 | 13.0 | C |
| MEE14 | 2x | - | 37509.243138691694 | 19.0 | B |
| RMA1 | 2x | - | 2417.585071339397 | 5.0 | C |
| AT4G01310 | 2x | - | 16784.278449498855 | 59.0 | HB |
| AT2G42110 | 2x | - | 26.648049077320806 | 11.0 | C |
| AUR1 | 2x | - | 103.8634618386168 | 12.0 | C |
| RPS7.1 | 2x | - | 3617.85099654389 | 44.0 | C |
| IAA6 | 2x | - | 1552.9169580804298 | 6.0 | C |
| ARF6 | 2x | - | 15.802380952380952 | 8.0 | C |
| ARF9 | 2x | - | 4049.378133386662 | 8.0 | C |
| TIM10 | 2x | - | 160.35114026229172 | 22.0 | C |
| AT5G61310 | 2x | 2x | 192.97925701031534 | 26.0 | C |
| AT5G02610 | 2x | - | 14869.102100443492 | 37.0 | B |
| ARF18 | 2x | - | 3363.7198396079857 | 10.0 | C |
| IAA28 | 2x | - | 5616.226906193051 | 9.0 | C |
| AT3G10090 | 2x | - | 3140.529547742115 | 27.0 | C |
| AT5G08180 | 2x | - | 41279.76027833143 | 104.0 | HB |
| TIM8 | 2x | - | 136.23611592780836 | 26.0 | C |
| AT3G44590 | 2x | - | 1767.354143978998 | 35.0 | C |
| AT3G42050 | 2x | - | 4955.1550334201165 | 43.0 | C |
| AT1G76720 | 2x | - | 21941.956997702964 | 113.0 | H |
| ABCF1 | 2x | 2x | 3554.110783395244 | 21.0 | C |
| AT1G18070 | 2x | - | 1186.2076735175174 | 30.0 | C |
| CYCA1;1 | 2x | - | 135.7071881872082 | 12.0 | C |
| ATK5 | 2x | - | 385.47074224580564 | 23.0 | C |
| AT3G07230 | 2x | - | 47.43473163865481 | 24.0 | C |
| AT4G29390.1 | 2x | - | 4785.96768133266 | 32.0 | C |
| TOM5 | 2x | - | 133.43747305319314 | 27.0 | C |
| AtG2 | 2x | - | 358.56156633748367 | 41.0 | C |
| HDT4 | 2x | - | 34.118754120100604 | 29.0 | C |
| AT2G25210.1 | 2x | - | 1025.153951236981 | 67.0 | H |
| TIM13 | 2x | - | 18224.627173305656 | 39.0 | B |
| ROPGEF6 | 2x | - | 131.65236028635448 | 13.0 | C |
| AT4G25890 | 2x | - | 67.90883295274313 | 33.0 | C |
| NOP10 | 2x | - | 61.312055574045395 | 28.0 | C |
| AT2G41650 | 2x | - | 95.48667974765306 | 21.0 | C |
| AT3G06320 | 2x | - | 259.17746133560445 | 32.0 | C |
| AT4G02800 | 2x | - | 913.6860370801403 | 17.0 | C |
| AT4G15830 | 2x | - | 2204.213067229546 | 12.0 | C |
| 3xHMG-box2 | 2x | - | 139.5334553160387 | 16.0 | C |
| ENODL15 | 2x | - | 65.24301349592946 | 12.0 | C |
| TOM6 | 2x | - | 162.6056333174437 | 30.0 | C |
| AT2G27700 | 2x | - | 28096.04506765029 | 109.0 | H |
| CSLD5 | 2x | - | 139.09752465451356 | 15.0 | C |
| AT3G02640 | 2x | - | 157.7304772827615 | 13.0 | C |
| AT1G76820 | 2x | - | 12525.053401282308 | 100.0 | H |
| AT3G02120 | 2x | - | 293.88444338371346 | 16.0 | C |
| AT5G36710 | 2x | - | 161.09038309481204 | 14.0 | C |
| AT2G07696.1 | 2x | - | 1918.3372142034327 | 43.0 | C |
| AT2G45860 | 2x | - | 47.64360160602528 | 30.0 | C |
| AT3G25940 | 2x | - | 63.225911304878686 | 22.0 | C |
| AxR3 | 2x | - | 2692.0440349886917 | 9.0 | C |
| ARF4 | 2x | 2x | 46431.63982275034 | 8.0 | B |
| IAA34 | 2x | - | 14.58809523809524 | 8.0 | C |
| IAA9 | 2x | 2x | 2503.9810489424235 | 8.0 | C |
| EIF3C | 2x | - | 2065.2614376878555 | 18.0 | C |
| AT3G06320.1 | 2x | - | 6.352382940268371 | 9.0 | C |
| SNRNP-G | 2x | - | 43544.87939681106 | 128.0 | HB |
| AT2G19750 | 2x | - | 249.9223615873646 | 39.0 | C |
| AT1G08580 | 2x | - | 294.3879642324886 | 27.0 | C |
| RPL23.1 | 2x | - | 0.0 | 1.0 | C |
| RPL24 | 2x | - | 0.0 | 1.0 | C |
| AT4G33420 | 2x | - | 2.568046216679765 | 12.0 | C |
| PRxCB | 2x | - | 62.39829607263738 | 14.0 | C |
| AT4G11290 | 2x | - | 2.568046216679765 | 12.0 | C |
| AKHSDH1 | 2x | - | 0.0 | 1.0 | C |
| AT5G10350 | 2x | - | 0.0 | 2.0 | C |
| AT1G12800 | 2x | - | 7163.1521914839 | 36.0 | C |
| Lhca6 | 2x | - | 0.0 | 1.0 | C |
| AT4G30680 | 2x | - | 0.0 | 1.0 | C |
| AT3G49960 | 2x | - | 3.2823319309654786 | 19.0 | C |
| BT2 | 2x | - | 28039.35396825397 | 2.0 | B |
| PER64 | 2x | - | 2.568046216679765 | 12.0 | C |
| AT4G08780 | 2x | - | 2.568046216679765 | 13.0 | C |
| OST1 | 2x | - | 14058.99873694867 | 6.0 | B |
| EMB2770 | 2x | - | 2141.031762373932 | 97.0 | H |
| CYP71B19 | 2x | - | 33640.1873015873 | 2.0 | B |
| AT4G30170 | 2x | - | 2.568046216679765 | 14.0 | C |
| CYP81D3 | 2x | - | 0.0 | 1.0 | C |
| CYP71B24 | 2x | - | 113250.0523809831 | 2.0 | B |
| AT5G15180 | 2x | - | 2.568046216679765 | 12.0 | C |
| AT4G26650 | 2x | - | 196.12124096638834 | 5.0 | C |
| AT3G13224 | 2x | - | 1882.7725384991584 | 6.0 | C |
| PTB3 | 2x | - | 9.72623821246788 | 5.0 | C |
| RHS19 | 2x | - | 5.073203922446911 | 19.0 | C |
| AT3G17070 | 2x | - | 2.568046216679765 | 12.0 | C |
| AT3G52120 | 2x | - | 220.13049503181807 | 6.0 | C |
| CPSRP54 | 2x | - | 0.0 | 1.0 | C |
| PYL5 | 2x | - | 5654.0 | 3.0 | C |
| HAI1 | 2x | 2x | 0.0 | 1.0 | C |
| CYP81F2 | 2x | - | 0.0 | 1.0 | C |
| AT2G38380 | 2x | - | 2.568046216679765 | 13.0 | C |
| AT4G16270 | 2x | - | 2.568046216679765 | 12.0 | C |
| AT1G20580 | 2x | - | 5578.716029377784 | 103.0 | H |
| SR34 | 2x | - | 3490.270421437793 | 59.0 | H |
| CYP705A3 | 2x | - | 14892.633333331976 | 2.0 | B |
| CYP94D2 | 2x | - | 145932.84993152364 | 101.0 | HB |
| CYP81D8 | 2x | - | 20425.53809523987 | 2.0 | B |
| CYP71B4 | 2x | - | 0.0 | 1.0 | C |
| LIS | 2x | - | 3286.4736733740724 | 90.0 | H |
| CLPS3 | 2x | - | 7.92750546892167 | 5.0 | C |
| AT2G37130 | 2x | - | 2.568046216679765 | 12.0 | C |
| CYP81D4 | 2x | - | 16956.0 | 4.0 | B |
| AT5G12190 | 2x | - | 20.930684503273895 | 4.0 | C |
| SR30 | 2x | 2x | 11674.034958771676 | 61.0 | HB |
| AT5G54910 | 2x | 2x | 22.5968650932664 | 2.0 | C |
| CYP91A2 | 2x | - | 9471.999206348699 | 3.0 | B |
| CYP71B38 | 2x | - | 107965.15317463456 | 4.0 | B |
| CYP71B23 | 2x | - | 0.0 | 1.0 | C |
| U2B&apos; | 2x | - | 2008.1401757725164 | 81.0 | H |
| YLS8 | 2x | - | 2043.1241268523179 | 85.0 | H |
| AT4G25550 | 2x | - | 4295.672552688384 | 10.0 | C |
| AT3G50990 | 2x | - | 2.568046216679765 | 12.0 | C |
| CYP71A19 | 2x | - | 3960.161904761583 | 3.0 | C |
| PRPL11 | 2x | - | 6442.088324195918 | 33.0 | C |
| PnsB3 | 2x | - | 24284.958131545067 | 79.0 | HB |
| AT2G38390 | 2x | - | 2.568046216679765 | 13.0 | C |
| CYP96A4 | 2x | - | 106933.4308839147 | 100.0 | HB |
| AT3G28200 | 2x | - | 1575.1365743678484 | 14.0 | C |
| CYP71B3 | 2x | - | 2859.0 | 2.0 | C |
| CYP78A7 | 2x | - | 3799.7230158727166 | 3.0 | C |
| CYP81D2 | 2x | - | 22379.348412698422 | 3.0 | B |
| WEE1 | 2x | - | 29478.33899719601 | 51.0 | HB |
| AT5G64110 | 2x | - | 2.568046216679765 | 12.0 | C |
| FIP1[V] | 2x | - | 0.0 | 1.0 | C |
| PRPL28 | 2x | - | 0.0 | 1.0 | C |
| AT2G43770 | 2x | - | 5215.774724790287 | 64.0 | H |
| AT1G17640 | 2x | - | 8665.554372024651 | 89.0 | H |
| AT5G44500 | 2x | - | 20303.458117480375 | 132.0 | HB |
| CYP77B1 | 2x | - | 35.333333333333336 | 2.0 | C |
| AT5G40150 | 2x | - | 3.5732039224469134 | 13.0 | C |
| AT5G40490 | 2x | - | 8778.426238103839 | 91.0 | H |
| PA2 | 2x | - | 2.568046216679765 | 12.0 | C |
| AT3G07590 | 2x | - | 1851.2303145342817 | 98.0 | H |
| AT5G43620 | 2x | - | 545.7817083573751 | 7.0 | C |
| smB | 2x | - | 19458.75830559014 | 131.0 | HB |
| AT5G02530 | 2x | - | 39872.00354440758 | 56.0 | HB |
| CYP71B28 | 2x | - | 8539.233333333334 | 3.0 | B |
| ABH1 | 2x | - | 29350.13639126725 | 124.0 | HB |
| U1-70K | 2x | - | 1139.310800559759 | 66.0 | H |
| AT4G31760 | 2x | - | 2.568046216679765 | 12.0 | C |
| CYCH;1 | 2x | - | 14733.405479618108 | 44.0 | B |
| ALY4 | 2x | - | 18046.192945195904 | 52.0 | HB |
| AT4G37530 | 2x | - | 116.30552229381387 | 14.0 | C |
| CYP71A14 | 2x | - | 100234.819047619 | 2.0 | B |
| CYP71B31 | 2x | - | 39273.839682539656 | 3.0 | B |
| TTN8 | 2x | - | 364.2475582062225 | 10.0 | C |
| CYP71B30P | 2x | - | 187.25317460317456 | 3.0 | C |
| AT4G17690 | 2x | - | 2.568046216679765 | 12.0 | C |
| UGT72E3 | 2x | - | 2682.2471589551487 | 84.0 | H |
| CYP71B34 | 2x | - | 8552.549206349206 | 3.0 | B |
| AT5G65260 | 2x | - | 3897.8714836323893 | 11.0 | C |
| ATO | 2x | - | 4575.2298001972 | 106.0 | H |
| CFIM-25 | 2x | - | 622.173830947499 | 5.0 | C |
| CYP71B22 | 2x | - | 94819.10476190472 | 3.0 | B |
| CYP82C4 | 2x | 2x | 75630.34920634917 | 4.0 | B |
| CYP81F3 | 2x | - | 0.0 | 1.0 | C |
| PRx52 | 2x | - | 116.30552229381387 | 13.0 | C |
| CYP71A20 | 2x | - | 13.360317460317464 | 2.0 | C |
| AT2G36480 | 2x | - | 6261.560207915434 | 10.0 | C |
| LSM2 | 2x | - | 343.8092206145896 | 79.0 | H |
| ATU2AF35A | 2x | - | 888.9170109299073 | 5.0 | C |
| UGT72E1 | 2x | - | 762.1815933843235 | 81.0 | H |
| PCFS4 | 2x | - | 487.0776509852356 | 8.0 | C |
| AT4G12600 | 2x | - | 40354.617912734466 | 79.0 | HB |
| CYP77A4 | 2x | - | 0.0 | 1.0 | C |
| CYP78A5 | 2x | - | 0.0 | 1.0 | C |
| AT3G52660 | 2x | - | 36.96961725256401 | 4.0 | C |
| PTB2 | 2x | - | 182.50610318421414 | 8.0 | C |
| AT3G44785 | 2x | - | 58.601852659143645 | 7.0 | C |
| AT2G18740 | 2x | - | 1360.0212211900796 | 82.0 | H |
| CYP71A15 | 2x | - | 0.0 | 1.0 | C |
| CYP81D1 | 2x | - | 14120.98174603175 | 3.0 | B |
| AT4G26010 | 2x | - | 3.1013795500130974 | 19.0 | C |
| AT5G17820 | 2x | - | 6.617863066496607 | 22.0 | C |
| AT5G55550 | 2x | - | 149.3796022035241 | 5.0 | C |
| RSZ22a | 2x | - | 111.48932375270734 | 6.0 | C |
| U2AF35B | 2x | - | 84.05589838229008 | 6.0 | C |
| CYP71B26 | 2x | - | 15009.125396823447 | 3.0 | B |
| AT5G20160 | 2x | - | 6524.771357440457 | 70.0 | H |
| AT3G23325 | 2x | - | 1750.131186361348 | 13.0 | C |
| U1A | 2x | - | 2254.7626901537456 | 85.0 | H |
| AT2G44710 | 2x | - | 110.87634680947377 | 4.0 | C |
| CYP78A8 | 2x | - | 7.0 | 2.0 | C |
| CYP71A16 | 2x | - | 32947.77936507576 | 5.0 | B |
| CSP3 | 2x | - | 2661.9816061479914 | 5.0 | C |
| AT5G47620 | 2x | - | 11.218956474483795 | 2.0 | C |
| PAD3 | 2x | - | 10405.315873018046 | 4.0 | B |
| CYP81F4 | 2x | - | 43967.53571428376 | 5.0 | B |
| AT5G28390 | 2x | - | 115.00151545174818 | 3.0 | C |
| AT5G22080 | 2x | - | 227.42762215563 | 7.0 | C |
| AT2G32600 | 2x | - | 7619.074424302242 | 110.0 | H |
| AT1G06960 | 2x | - | 11185.968789277435 | 87.0 | HB |
| SUA | 2x | - | 534.0317149139312 | 4.0 | C |
| AT4G22380 | 2x | - | 40864.70511014179 | 77.0 | HB |
| AT5G09390 | 2x | - | 325.24037486249506 | 7.0 | C |
| AT5G60940 | 2x | - | 622.5625929916156 | 9.0 | C |
| CYP77A9 | 2x | - | 2912.081746031746 | 3.0 | C |
| CYP71B35 | 2x | 4x | 5654.0 | 2.0 | C |
| CYP71B10 | 2x | 2x | 42160.515079365054 | 5.0 | B |
| AT5G65220 | 2x | - | 0.0 | 1.0 | C |
| CSDP1 | 2x | - | 113.5860225945923 | 5.0 | C |
| BTR1L | 2x | - | 52.05976045920993 | 5.0 | C |
| AT4G21660 | 2x | - | 6314.34420912028 | 96.0 | H |
| SC35 | 2x | - | 257.54920426936775 | 11.0 | C |
| AT4G02840 | 2x | - | 4857.447057870706 | 101.0 | H |
| AT4G14342 | 2x | - | 51.893544603858224 | 5.0 | C |
| EMB3011 | 2x | - | 826.820372961841 | 59.0 | H |
| CYP71B5 | 2x | - | 90.78174603174604 | 3.0 | C |
| CYP81H1 | 2x | 2x | 2795.0 | 2.0 | C |
| CSTF64 | 2x | - | 6233.843165543462 | 8.0 | C |
| SR34b | 2x | - | 12988.25019667097 | 62.0 | HB |
| AT3G11500 | 2x | - | 1323.326467926209 | 97.0 | H |
| AT5G24070 | 2x | - | 2.568046216679765 | 12.0 | C |
| UGT72E2 | 2x | 2x | 762.1815933843235 | 81.0 | H |
| AT5G59950 | 2x | - | 18634.18197815457 | 50.0 | B |
| CYP71B17 | 2x | - | 11538.479365079362 | 5.0 | B |
| AT4G30330 | 2x | - | 1777.2612422048262 | 83.0 | H |
| SmD3 | 2x | - | 3310.7386915747616 | 103.0 | H |
| CPSF100 | 2x | - | 17.850505050505053 | 3.0 | C |
| PABN1 | 2x | 2x | 7654.491980688035 | 7.0 | C |
| SR45 | 2x | - | 13438.09012889056 | 92.0 | HB |
| CSTF77 | 2x | - | 6174.155336396834 | 11.0 | C |
| AT4G14300 | 2x | - | 362.57469624277087 | 7.0 | C |
| NRPB7 | 2x | - | 116.86625786185671 | 8.0 | C |
| CYP71B36 | 2x | - | 24.0 | 2.0 | C |
| CYP71B14 | 2x | - | 4696.900000000029 | 3.0 | C |
| CPSF160 | 2x | - | 767.7340752680262 | 5.0 | C |
| AT5G51890 | 2x | - | 2.568046216679765 | 12.0 | C |
| RSZ21 | 2x | - | 102.23912769453011 | 5.0 | C |
| AT2G14870 | 2x | - | 13.359670156965684 | 6.0 | C |
| AT3G07810 | 2x | - | 3736.100601978317 | 11.0 | C |
| UPF3 | 2x | - | 14140.09449192159 | 28.0 | B |
| RSZ22 | 2x | - | 163.54623560562968 | 5.0 | C |
| CYP71B11 | 2x | - | 22633.35396825397 | 3.0 | B |
| CYP71B12 | 2x | - | 228.1666666666666 | 2.0 | C |
| SR34a | 2x | - | 9835.675471127593 | 62.0 | HB |
| CBP20 | 2x | - | 366603.4398299623 | 221.0 | HB |
| RUxF | 2x | - | 3826.1119140875867 | 93.0 | H |
| CLPS5 | 2x | - | 70.35866858413605 | 8.0 | C |
| RBP1 | 2x | - | 6155.7055710425475 | 88.0 | H |
| GRP2 | 2x | - | 5307.055938742169 | 12.0 | C |
| AT2G47640 | 2x | - | 10855.612383637614 | 107.0 | H |
| SIG4 | 2x | 2x | 0.7095959595959597 | 2.0 | C |
| AT5G17010 | 2x | 2x | 0.0 | 1.0 | C |
| AT1G60900 | 2x | - | 0.0 | 2.0 | C |
| FAS2 | 2x | - | 0.75 | 3.0 | C |
| APG8H | 2x | - | 3.1666666666666665 | 2.0 | C |
| ALY2 | 2x | - | 0.0 | 1.0 | C |
| NPC4 | 2x | - | 0.0 | 2.0 | C |
| ATG8C | 2x | - | 1.3722222222222222 | 3.0 | C |
| NPC5 | 2x | - | 6049.15076820403 | 5.0 | C |
| AT2G32415 | 2x | - | 13250.30628647027 | 39.0 | B |
| ATG8H | 2x | - | 16.7443150554296 | 3.0 | C |
| AT5G20080 | 2x | 2x | 0.0 | 2.0 | C |
| AT1G60830 | 2x | - | 1.1572871572871573 | 2.0 | C |
| AT2G33440 | 2x | - | 0.0 | 1.0 | C |
| MAGO | 2x | - | 50202.52683040229 | 74.0 | HB |
| AT2G24350 | 2x | - | 4663.358989677025 | 42.0 | C |
| ATG8B | 2x | - | 0.0 | 2.0 | C |
| AT5G19440 | 2x | 2x | 767.6869883288049 | 2.0 | C |
| AT3G01590 | 2x | - | 21.0323191586824 | 14.0 | C |
| ASF1B | 2x | - | 507.0757465008968 | 10.0 | C |
| APG8A | 2x | - | 4553.571942455075 | 5.0 | C |
| AT5G35910 | 2x | - | 4235.721328625728 | 22.0 | C |
| NPC1 | 2x | - | 755.5535513752827 | 3.0 | C |
| AT3G02360 | 2x | - | 1.7853208449805118 | 5.0 | C |
| AT1G54440 | 2x | - | 5676.5595989099165 | 24.0 | C |
| PLC2 | 2x | 2x | 11771.488628352754 | 13.0 | C |
| SGA2 | 2x | - | 315.1983610998701 | 10.0 | C |
| AT1G01090.1 | 2x | - | 129.2248503709429 | 22.0 | C |
| NPC2 | 2x | - | 47.51524175688654 | 4.0 | C |
| PLC4 | 2x | - | 103.93250827308749 | 12.0 | C |
| AT5G64380 | 2x | - | 858.2943710558017 | 13.0 | C |
| RIN1 | 2x | - | 6166.978846527361 | 11.0 | C |
| PLC3 | 2x | - | 11074.35137636223 | 14.0 | C |
| ATPEN2 | 2x | - | 415.86613760835417 | 12.0 | C |
| F28P22.13 | 2x | - | 68.27310651754436 | 10.0 | C |
| NPC3 | 2x | - | 78.004928095636 | 4.0 | C |
| NPC6 | 2x | - | 2.0 | 3.0 | C |
| PIP5K3 | 2x | - | 6.433211233211234 | 8.0 | C |
| AT2G33435 | 2x | - | 4.91904057659838 | 3.0 | C |
| ELF6 | 2x | - | 8034.948052698016 | 9.0 | C |
| HAT3.1 | 2x | - | 4984.661506237591 | 9.0 | C |
| EER5 | 2x | - | 533.6121985983452 | 5.0 | C |
| AT4G28310 | 2x | - | 143.6517914197967 | 8.0 | C |
| AT3G55940 | 2x | - | 97.15879526318061 | 11.0 | C |
| AT2G40116 | 2x | - | 42.12012605350168 | 13.0 | C |
| PTEN1 | 2x | - | 538.1488423703887 | 13.0 | C |
| PIP5K9 | 2x | - | 58.44870453159933 | 6.0 | C |
| PIPK10 | 2x | - | 64.07371958918294 | 13.0 | C |
| PIP5K1 | 2x | - | 34.0756451756967 | 9.0 | C |
| VPS34 | 2x | - | 9918.888609201065 | 9.0 | C |
| GAPCP-2 | 2x | - | 1611.2731468343154 | 21.0 | C |
| THO2 | 2x | - | 5.684451079663247 | 3.0 | C |
| AT5G54970 | 2x | - | 122.51204791316387 | 6.0 | C |
| SAC3B | 2x | - | 2492.29121571291 | 7.0 | C |
| AT2G47250 | 2x | - | 1185.2221066064105 | 64.0 | H |
| AT3G62310 | 2x | - | 1488.6140162770157 | 65.0 | H |
| SAC3C | 2x | - | 878.9271507292764 | 9.0 | C |
| AT1G64190 | 2x | - | 7.889567163438692 | 6.0 | C |
| AT5G41670 | 2x | - | 972.1552844048446 | 9.0 | C |
| AT1G66260 | 2x | - | 12943.778791851446 | 46.0 | B |
| REF6 | 2x | - | 4.946140173561431 | 6.0 | C |
| AT1G10900 | 2x | - | 22.352515482126595 | 13.0 | C |
| AT1G60890 | 2x | - | 41.63135133269347 | 11.0 | C |
| HCEF1 | 2x | 2x | 69833.53987313021 | 153.0 | HB |
| At2g31630 | 2x | - | 292.2119791277937 | 12.0 | C |
| AT5G14900 | 2x | - | 1373.4970440638185 | 63.0 | H |
| AT3G25960 | 2x | - | 1196.2522680332115 | 25.0 | C |
| ATHxK4 | 2x | - | 4051.3084860824797 | 28.0 | C |
| AT3G61610 | 2x | - | 20.732820959380724 | 8.0 | C |
| ATx2 | 2x | - | 128.80947108598943 | 8.0 | C |
| AT1G22170 | 2x | - | 4363.226859787448 | 29.0 | C |
| NADP-ME3 | 2x | - | 2028.8168938576728 | 18.0 | C |
| PIPK11 | 2x | - | 13562.885296067932 | 13.0 | C |
| PIP5K5 | 2x | - | 13033.710376962501 | 13.0 | C |
| G6PD3 | 2x | - | 1532.9219572041277 | 28.0 | C |
| AT5G22620 | 2x | - | 220.16309659335647 | 10.0 | C |
| AT5G08570 | 2x | - | 1016.4110766906939 | 28.0 | C |
| PIP5K4 | 2x | - | 12.174636231141525 | 8.0 | C |
| ATU2AF65A | 2x | - | 1.6734566793690935 | 4.0 | C |
| UAP56a | 2x | 2x | 12601.849967405105 | 19.0 | B |
| MAB1 | 2x | - | 438.45754110668435 | 23.0 | C |
| AT4G26390 | 2x | - | 1752.4586439715472 | 29.0 | C |
| AT1G59900 | 2x | - | 283.56630527410374 | 24.0 | C |
| PIP5K6 | 2x | 2x | 21477.94329292994 | 15.0 | C |
| AT5G04120 | 2x | - | 62.09550958757079 | 14.0 | C |
| NAD-ME2 | 2x | - | 295.2564407344366 | 19.0 | C |
| PI-4KBETA2 | 2x | - | 7330.345232174224 | 16.0 | C |
| AT3G50520 | 2x | - | 28.511846099379547 | 12.0 | C |
| DUT1 | 2x | - | 30.041146067819835 | 8.0 | C |
| AT5G63680 | 2x | - | 4882.296531702037 | 29.0 | C |
| PPC4 | 2x | - | 1448.657142058239 | 28.0 | C |
| PPC2 | 2x | - | 3577.8868843516716 | 30.0 | C |
| ATG8E | 2x | - | 9.242052185860233 | 2.0 | C |
| NADP-ME1 | 2x | - | 859.4308445644926 | 25.0 | C |
| AT5G56350 | 2x | - | 2680.1013260262976 | 37.0 | C |
| AT4G23730 | 2x | - | 9.923127151487131 | 12.0 | C |
| PGMP | 2x | - | 363.9192087344264 | 22.0 | C |
| GAPC1 | 2x | - | 2917.7261829779186 | 31.0 | C |
| G6PD5 | 2x | - | 5385.5926926859265 | 24.0 | C |
| ATG8G | 2x | - | 0.0 | 2.0 | C |
| G6PD1 | 2x | - | 6417.447843273247 | 43.0 | C |
| PLC5 | 2x | - | 12969.878261315911 | 16.0 | C |
| NAD-ME1 | 2x | - | 274.5550984540627 | 21.0 | C |
| AT2G34590 | 2x | - | 204.63942247925664 | 24.0 | C |
| AT5G66530 | 2x | - | 47.043848378699906 | 12.0 | C |
| PGI1 | 2x | - | 64018.21461971316 | 93.0 | HB |
| AT5G57330 | 2x | - | 11.328885171720504 | 14.0 | C |
| iPGAM1 | 2x | 2x | 72.27698209625699 | 16.0 | C |
| AT2G36580 | 2x | - | 3185.3154839033646 | 34.0 | C |
| AT5G47435 | 2x | - | 785.306290258358 | 19.0 | C |
| PGM2 | 2x | - | 275.9911107506314 | 21.0 | C |
| PPC1 | 2x | 2x | 4243.598416271569 | 31.0 | C |
| AT4G37560 | 2x | - | 25.576699300497683 | 12.0 | C |
| HxK3 | 2x | - | 172.41641031051202 | 23.0 | C |
| NADP-ME2 | 2x | - | 573.9034235859034 | 23.0 | C |
| AT1G78050.1 | 2x | - | 134.5933506085609 | 20.0 | C |
| PIP5K2 | 2x | - | 351.8116848309592 | 15.0 | C |
| PKP-ALPHA | 2x | - | 5873.02444289214 | 31.0 | C |
| IAR4 | 2x | - | 154.80464612308225 | 21.0 | C |
| T2H7.8 | 2x | - | 301.15751091761683 | 25.0 | C |
| AT4G25900 | 2x | - | 34.40905238015695 | 13.0 | C |
| TRA2 | 2x | 2x | 2527.945966746124 | 34.0 | C |
| F3A4.190 | 2x | - | 358.66782418922514 | 14.0 | C |
| MZN1.12 | 2x | - | 187.40484349317668 | 15.0 | C |
| ATG8F | 2x | 2x | 56427.677240573335 | 5.0 | B |
| TPI | 2x | - | 3259.488414563983 | 33.0 | C |
| AT3G55810 | 2x | - | 4970.580501073333 | 25.0 | C |
| PGM3 | 2x | - | 2340.3156320743196 | 32.0 | C |
| PFK2 | 2x | - | 197.50293840477454 | 15.0 | C |
| PFK1 | 2x | - | 903.825305824382 | 21.0 | C |
| G6PD4 | 2x | - | 967.5195727698232 | 30.0 | C |
| GAPCP-1 | 2x | - | 34.88002909197067 | 17.0 | C |
| G6PD2 | 2x | - | 4923.565246934518 | 32.0 | C |
| AT3G60750 | 2x | - | 9670.555396843267 | 53.0 | HB |
| AT5G14500 | 2x | - | 37.53200270579686 | 13.0 | C |
| NADP-ME4 | 2x | - | 3667.379803671103 | 18.0 | C |
| AT3G04050 | 2x | - | 5837.69921354336 | 28.0 | C |
| iPGAM2 | 2x | - | 459.01192383209417 | 12.0 | C |
| AT3G49160 | 2x | - | 2829.1254935456527 | 27.0 | C |
| FDH | 2x | 2x | 2096.9793247183334 | 16.0 | C |
| PKp3 | 2x | - | 3403.423793219964 | 28.0 | C |
| G6PD6 | 2x | - | 595.7037771711903 | 31.0 | C |
| AT3G55650 | 2x | - | 561.9564771778021 | 27.0 | C |
| AT4G17360 | 2x | - | 841.6545298203896 | 21.0 | C |
| PPC3 | 2x | - | 4335.697075937474 | 35.0 | C |
| PKP-BETA1 | 2x | - | 6872.211832251488 | 30.0 | C |
| ALNS | 2x | - | 0.0 | 1.0 | C |
| YUC4 | 2x | - | 0.4778977542135438 | 4.0 | C |
| YUC5 | 2x | - | 0.4778977542135438 | 4.0 | C |
| UBP16 | 2x | - | 0.0 | 1.0 | C |
| YUC9 | 2x | - | 0.4778977542135438 | 4.0 | C |
| SIS | 2x | 2x | 0.0 | 2.0 | C |
| F3F9.16 | 2x | - | 5461.330245622461 | 12.0 | C |
| VIP3 | 2x | - | 85.92909906542961 | 4.0 | C |
| AT4G37520 | 2x | - | 201.2973694592343 | 15.0 | C |
| YUC3 | 2x | - | 0.4778977542135438 | 4.0 | C |
| PAO3 | 2x | - | 0.0 | 13.0 | C |
| AT2G18150 | 2x | - | 2.568046216679765 | 13.0 | C |
| AT4G29690 | 2x | - | 14.10177180394677 | 8.0 | C |
| AT5G50120 | 2x | - | 85.92909906542961 | 4.0 | C |
| AT3G51930 | 2x | - | 638.3626105040734 | 3.0 | C |
| ILA | 2x | - | 739.3480368186737 | 8.0 | C |
| APK3 | 2x | - | 1.0413667780358495 | 7.0 | C |
| PSP | 2x | - | 6.23030303030303 | 12.0 | C |
| AT1G32780 | 2x | - | 7.324872422851775 | 14.0 | C |
| AT4G29680 | 2x | - | 6466.01244217048 | 9.0 | C |
| AKN2 | 2x | - | 1.0413667780358495 | 7.0 | C |
| APK4 | 2x | - | 1.0413667780358495 | 7.0 | C |
| AT4G24710 | 2x | - | 164.35583373392237 | 7.0 | C |
| OMR1 | 2x | - | 35.904994639233486 | 13.0 | C |
| YUC6 | 2x | - | 0.4778977542135438 | 4.0 | C |
| YUC7 | 2x | - | 0.4778977542135438 | 4.0 | C |
| AT1G77100 | 2x | - | 2.568046216679765 | 12.0 | C |
| AT3G62550 | 2x | - | 553.3713545963408 | 9.0 | C |
| RHL1 | 2x | - | 0.0 | 1.0 | C |
| J11 | 2x | 2x | 204.01804250037696 | 4.0 | C |
| AT2G18980 | 2x | - | 2.568046216679765 | 13.0 | C |
| AT2G34060 | 2x | - | 213.37215738168425 | 14.0 | C |
| AT2G24800 | 2x | - | 1461.3990982907187 | 13.0 | C |
| atao1 | 2x | - | 8.406535947712417 | 14.0 | C |
| AT1G47400 | 2x | - | 5654.0 | 5.0 | C |
| AT5G05250 | 2x | 2x | 0.0 | 1.0 | C |
| AT3G18950 | 2x | - | 0.0 | 2.0 | C |
| AT4G34380 | 2x | - | 0.0 | 2.0 | C |
| PYD4 | 2x | 2x | 210.5445864732588 | 17.0 | C |
| SIR | 2x | 2x | 7758.385307619239 | 19.0 | B |
| AT4G36430 | 2x | - | 2.7218923705259184 | 14.0 | C |
| AT1G31710 | 2x | - | 0.0 | 13.0 | C |
| AT5G47000 | 2x | - | 2.568046216679765 | 12.0 | C |
| AT4G22110 | 2x | - | 7.324872422851775 | 14.0 | C |
| AT5G66390 | 2x | - | 2.568046216679765 | 12.0 | C |
| AT2G35380 | 2x | - | 2.568046216679765 | 13.0 | C |
| T14E10.210 | 2x | - | 1283.94579342266 | 11.0 | C |
| TRIP-1 | 2x | - | 1729.4257064802741 | 5.0 | C |
| AT3G01190 | 2x | - | 2.8537605023940515 | 16.0 | C |
| MTHFR1 | 2x | - | 2.885689380753159 | 15.0 | C |
| YUC1 | 2x | - | 0.4778977542135438 | 4.0 | C |
| AT5G03430 | 2x | 2x | 1616.1372606459583 | 14.0 | C |
| PAO2 | 2x | - | 0.0 | 13.0 | C |
| AT5G06730 | 2x | - | 2.568046216679765 | 12.0 | C |
| PRK | 2x | - | 16996.71298915196 | 137.0 | HB |
| AT2G41480 | 2x | - | 2.568046216679765 | 12.0 | C |
| AT5G64100 | 2x | - | 2.568046216679765 | 13.0 | C |
| MTHFR2 | 2x | - | 595.7665494070278 | 16.0 | C |
| AT5G14130 | 2x | - | 2.568046216679765 | 12.0 | C |
| MYBL2 | 2x | - | 16535.840423115656 | 7.0 | B |
| AT2G35120 | 2x | - | 12.302681609453787 | 17.0 | C |
| WCRKC1 | 2x | 2x | 76731.93645810745 | 16.0 | B |
| AT4G20930 | 2x | - | 73.93410615554737 | 3.0 | C |
| NIR1 | 2x | - | 8999.91177691948 | 17.0 | B |
| AT1G30870 | 2x | - | 3.1013795500130974 | 19.0 | C |
| PAO4 | 2x | - | 0.0 | 13.0 | C |
| PAO5 | 2x | - | 0.0 | 13.0 | C |
| AT4G00600 | 2x | - | 84.84649533327593 | 20.0 | C |
| AT3G03670 | 2x | - | 2.568046216679765 | 12.0 | C |
| THA1 | 2x | 2x | 2485.324869165893 | 23.0 | C |
| EMB3127 | 2x | - | 84.84649533327593 | 20.0 | C |
| AT2G18140 | 2x | - | 2.568046216679765 | 13.0 | C |
| PRxCA | 2x | - | 1461.3990982907187 | 13.0 | C |
| YUC2 | 2x | - | 0.4778977542135438 | 4.0 | C |
| AT4G38090 | 2x | 2x | 339.2208854244134 | 5.0 | C |
| VAR2 | 2x | - | 11.490766907511379 | 34.0 | C |
| AT1G64710 | 2x | - | 1766.1218307903175 | 17.0 | C |
| AT4G25980 | 2x | - | 2.568046216679765 | 12.0 | C |
| AT2G39040 | 2x | - | 2.568046216679765 | 14.0 | C |
| Fes1B | 2x | - | 0.0 | 7.0 | C |
| ALDH6B2 | 2x | - | 48.99322828209243 | 6.0 | C |
| SR | 2x | - | 6.23030303030303 | 12.0 | C |
| TSB2 | 2x | - | 528.0236615030112 | 9.0 | C |
| AT1G68850 | 2x | - | 2.568046216679765 | 13.0 | C |
| AT1G71500 | 2x | - | 1164.1589079464634 | 65.0 | H |
| AT2G22420 | 2x | - | 2.568046216679765 | 12.0 | C |
| AT1G80380 | 2x | - | 5743.771198542915 | 7.0 | C |
| Prx37 | 2x | - | 245.964883515147 | 15.0 | C |
| THA2 | 2x | - | 1333.9304315178547 | 22.0 | C |
| AT3G12290 | 2x | - | 119.2054309356589 | 21.0 | C |
| AGT3 | 2x | - | 2942.1548345004926 | 19.0 | C |
| SERK4 | 2x | - | 0.0 | 1.0 | C |
| GLDP2 | 2x | - | 3775.771564726446 | 41.0 | C |
| AT1G49570 | 2x | - | 2.568046216679765 | 13.0 | C |
| TSB1 | 2x | - | 528.0236615030112 | 9.0 | C |
| GUN5 | 2x | - | 2564.6556917015664 | 90.0 | H |
| AT5G58400 | 2x | - | 2.568046216679765 | 12.0 | C |
| RCI3 | 2x | - | 88.74680362654351 | 13.0 | C |
| RAD51C | 2x | - | 341.74004459810504 | 8.0 | C |
| SHM1 | 2x | 2x | 73803.2485038506 | 169.0 | HB |
| HOT5 | 2x | - | 2304.2422942057296 | 16.0 | C |
| AGT2 | 2x | - | 1606.534859828681 | 19.0 | C |
| AT1G14550 | 2x | - | 2.568046216679765 | 13.0 | C |
| AT1G71695 | 2x | - | 2.568046216679765 | 12.0 | C |
| CSP41A | 2x | - | 4564.62882685241 | 125.0 | H |
| AT4G29700 | 2x | - | 14.10177180394677 | 8.0 | C |
| EBS5 | 2x | - | 0.0 | 2.0 | C |
| APK | 2x | - | 62.53310426432395 | 8.0 | C |
| AT2G33830 | 2x | - | 1919.121634235166 | 11.0 | C |
| AT4G29710 | 2x | - | 14.10177180394677 | 8.0 | C |
| AT1G44000 | 2x | - | 54.522082509217135 | 57.0 | H |
| PSB28 | 2x | - | 2160.3309455046397 | 65.0 | H |
| GDCH | 2x | - | 4384.268879751142 | 91.0 | H |
| AT5G51010 | 2x | - | 1298.9975043396191 | 53.0 | H |
| TOP6B | 2x | - | 708.3340799780036 | 7.0 | C |
| PANC | 2x | - | 0.0 | 7.0 | C |
| THY-1 | 2x | - | 5416.044576495074 | 27.0 | C |
| THY-2 | 2x | - | 5416.044576495074 | 27.0 | C |
| AT1G22440 | 2x | - | 56.95954024428706 | 15.0 | C |
| SBPASE | 2x | 2x | 33876.22959767079 | 162.0 | HB |
| AT5G64120 | 2x | - | 215.68380556162256 | 13.0 | C |
| CYP38 | 2x | - | 2245.948980722233 | 119.0 | H |
| AT4G01150 | 2x | - | 748.7749537189349 | 111.0 | H |
| AT5G58390 | 2x | - | 2.568046216679765 | 12.0 | C |
| AT2G38660 | 2x | - | 84.84649533327593 | 20.0 | C |
| AT3G48420 | 2x | - | 19.75272796184334 | 62.0 | H |
| AT3G21770 | 2x | - | 2.568046216679765 | 12.0 | C |
| AT4G02610 | 2x | - | 528.3474710268208 | 10.0 | C |
| AT5G19880 | 2x | - | 2.568046216679765 | 12.0 | C |
| AT5G51970 | 2x | - | 3385.7105003662273 | 20.0 | C |
| AT1G14345 | 2x | - | 979.9333115223907 | 63.0 | H |
| DMC1 | 2x | - | 452.01601665218357 | 9.0 | C |
| YUC8 | 2x | - | 0.4778977542135438 | 4.0 | C |
| BETA-UP | 2x | - | 1381.9443908153387 | 12.0 | C |
| AT1G24110 | 2x | - | 2.568046216679765 | 12.0 | C |
| AT1G31220 | 2x | - | 24.041121945101516 | 13.0 | C |
| pde194 | 2x | - | 0.0 | 5.0 | C |
| AT5G38520 | 2x | - | 13586.923284762097 | 75.0 | HB |
| AT5G19890 | 2x | - | 2.568046216679765 | 13.0 | C |
| AT1G74730 | 2x | - | 17.449305004476397 | 61.0 | H |
| AT1G44970 | 2x | - | 32.53816273183268 | 13.0 | C |
| GDC1 | 2x | - | 7.5067398731972546 | 58.0 | H |
| AT4G02530 | 2x | - | 17.449305004476397 | 61.0 | H |
| PnsB1 | 2x | - | 619.0630720043041 | 79.0 | H |
| PSAK | 2x | 2x | 4607.21513598685 | 119.0 | H |
| PGRL1A | 2x | - | 1893.6895383018991 | 66.0 | H |
| ATPD | 2x | - | 53422.26218429814 | 182.0 | HB |
| PGLP1 | 2x | - | 7764.345114369649 | 95.0 | H |
| PDE334 | 2x | 2x | 12896.495624278761 | 133.0 | HB |
| NPQ4 | 2x | - | 2316.340836273879 | 99.0 | H |
| LQY1 | 2x | - | 954.6104832114478 | 117.0 | H |
| Rbcx1 | 2x | - | 9.359963276768006 | 9.0 | C |
| FNR2 | 2x | - | 15414.832089028374 | 94.0 | HB |
| ZKT | 2x | - | 597.4906868736666 | 80.0 | H |
| ALDH3F1 | 2x | 2x | 92523.8966202741 | 72.0 | HB |
| AT1G22430 | 2x | - | 4353.129843987645 | 31.0 | C |
| ATPC1 | 2x | - | 36791.656187216366 | 194.0 | HB |
| PER4 | 2x | - | 227.49504126165678 | 14.0 | C |
| OHP | 2x | - | 44.89124452801899 | 63.0 | H |
| AT5G08050 | 2x | 2x | 86.41151171649332 | 64.0 | H |
| AT4G12290 | 2x | 2x | 2670.3856463423363 | 16.0 | C |
| RHL2 | 2x | 2x | 12151.89086660423 | 17.0 | B |
| AT5G39770 | 2x | - | 110.34839008091616 | 7.0 | C |
| GGAT1 | 2x | - | 3426.1169333377543 | 95.0 | H |
| PRxQ | 2x | - | 2878.758257577259 | 119.0 | H |
| MUS81 | 2x | - | 110.34839008091616 | 7.0 | C |
| xRCC3 | 2x | - | 10669.785654213878 | 10.0 | C |
| CCL | 2x | - | 867.8180667372009 | 10.0 | C |
| AT4G32340 | 2x | - | 9.359963276768006 | 9.0 | C |
| AT3G47070 | 2x | - | 1076.1077133040199 | 116.0 | H |
| TLP18.3 | 2x | - | 9758.168191199504 | 129.0 | HB |
| PER2 | 2x | - | 2.568046216679765 | 12.0 | C |
| RPS1 | 2x | - | 110776.52861972898 | 111.0 | HB |
| RIBA2 | 2x | - | 2634.9905920956116 | 7.0 | C |
| AT3G47160 | 2x | - | 523.7469935080258 | 11.0 | C |
| CRB | 2x | - | 6373.712200506691 | 130.0 | H |
| AT2G21550 | 2x | - | 2995.9117730756657 | 19.0 | C |
| PRxR1 | 2x | 2x | 1616.252531677342 | 18.0 | C |
| PSBQA | 2x | - | 1835.628690473283 | 125.0 | H |
| AT3G32980 | 2x | - | 2.568046216679765 | 12.0 | C |
| AT2G43480 | 2x | - | 2.568046216679765 | 12.0 | C |
| AT1G11860 | 2x | - | 3478.5231507836224 | 82.0 | H |
| DRT112 | 2x | - | 1113.4770051411858 | 119.0 | H |
| HPR | 2x | - | 27459.111599605338 | 148.0 | HB |
| AT5G07020 | 2x | - | 834.370347151126 | 112.0 | H |
| DFD | 2x | - | 643.3258090184734 | 46.0 | C |
| PSBP-1 | 2x | - | 1031.8551196327696 | 121.0 | H |
| HCF244 | 2x | - | 258.33145424462685 | 64.0 | H |
| AT1G34510 | 2x | - | 2.568046216679765 | 18.0 | C |
| TRxF1 | 2x | - | 6262.9359411392825 | 122.0 | HB |
| xTH7 | 2x | 2x | 178.67448213535778 | 11.0 | C |
| GAPB | 2x | - | 36789.14216438173 | 159.0 | HB |
| PETC | 2x | - | 69505.25880434661 | 165.0 | HB |
| PGK1 | 2x | - | 17136.958774104416 | 74.0 | HB |
| AT5G28237 | 2x | - | 528.0236615030112 | 9.0 | C |
| TSBtype2 | 2x | - | 528.690328169678 | 11.0 | C |
| AT2G43560 | 2x | - | 2091.695082199198 | 117.0 | H |
| ACP4 | 2x | - | 10.228921704494526 | 61.0 | H |
| PSAN | 2x | - | 1933.0767642268418 | 126.0 | H |
| THFS | 2x | - | 427.94060381403506 | 19.0 | C |
| AT2G35040 | 2x | - | 15974.366306028889 | 83.0 | HB |
| RHS18 | 2x | - | 3.2823319309654786 | 19.0 | C |
| ACD2 | 2x | - | 0.0 | 1.0 | C |
| EBS | 2x | 2x | 0.0 | 1.0 | C |
| AT2G30160 | 2x | - | 0.0 | 2.0 | C |
| PPa2 | 2x | - | 3874.144219516904 | 33.0 | C |
| PAL3 | 2x | - | 1238.8202242477782 | 8.0 | C |
| AT1G07030 | 2x | - | 0.0 | 1.0 | C |
| MFDx2 | 2x | - | 2423.395188121553 | 76.0 | H |
| TRO | 2x | - | 5656.354754912586 | 3.0 | C |
| AT3G07180 | 2x | - | 0.0 | 1.0 | C |
| TAP38 | 2x | 2x | 0.0 | 1.0 | C |
| VHP2;2 | 2x | - | 113.09463747149977 | 31.0 | C |
| PPa1 | 2x | - | 1050.1442195168904 | 33.0 | C |
| PPa5 | 2x | - | 3878.20482557751 | 35.0 | C |
| AVP1 | 2x | 2x | 4958.699803708286 | 33.0 | C |
| AT3G07140 | 2x | - | 5654.0 | 2.0 | C |
| AT5G19130 | 2x | - | 11304.0 | 2.0 | B |
| ISU2 | 2x | - | 5902.583786280625 | 22.0 | C |
| AT3G26480 | 2x | - | 471.05108336575296 | 20.0 | C |
| AT5G06410 | 2x | - | 248.58378628066458 | 23.0 | C |
| AT5G46420 | 2x | - | 224.41279404612678 | 25.0 | C |
| AT1G58983.1 | 2x | - | 438.62630997614815 | 22.0 | C |
| PSAF | 2x | - | 2787.9660690976134 | 109.0 | H |
| NYE1 | 2x | 2x | 45154.0 | 4.0 | B |
| AT1G07210 | 2x | - | 361.972033440737 | 20.0 | C |
| AT4G35370 | 2x | - | 1675.1674533989062 | 27.0 | C |
| AT4G23540 | 2x | - | 377.1641510040974 | 17.0 | C |
| PYR4 | 2x | 2x | 163.9216367114506 | 3.0 | C |
| PAL4 | 2x | - | 1149.69810097198 | 8.0 | C |
| PAL2 | 2x | - | 1325.0138173366645 | 13.0 | C |
| AT4G04360 | 2x | - | 19.754458185762374 | 16.0 | C |
| AT2G32580 | 2x | - | 251.9848160910274 | 17.0 | C |
| NKS1 | 2x | - | 22.75016037187584 | 16.0 | C |
| PYRD | 2x | - | 168.91058848415778 | 15.0 | C |
| AT5G64650 | 2x | - | 392.2227740344413 | 17.0 | C |
| MFDx1 | 2x | - | 6079.84738873851 | 76.0 | H |
| AT4G28080 | 2x | - | 568.3201183696423 | 12.0 | C |
| AT4G18900 | 2x | - | 5019.854119887236 | 19.0 | C |
| AT4G30990 | 2x | - | 21.500884287245693 | 15.0 | C |
| AT3G16840 | 2x | - | 643.4712556469658 | 21.0 | C |
| NAP57 | 2x | - | 100810.5064470869 | 103.0 | HB |
| AT4G21445 | 2x | 2x | 32.10767087101975 | 7.0 | C |
| AT1G05070 | 2x | - | 193.5654832695411 | 23.0 | C |
| AT1G69070 | 2x | - | 1654.5011619951758 | 33.0 | C |
| AAT | 2x | - | 198.55751404924428 | 10.0 | C |
| At3g03600 | 2x | - | 281.7974832199277 | 21.0 | C |
| AT1G06720 | 2x | - | 189.82546574838415 | 23.0 | C |
| ISU1 | 2x | 2x | 400.83190425963335 | 22.0 | C |
| NFS1 | 2x | - | 20776.280995571724 | 25.0 | B |
| AT2G43030 | 2x | 2x | 3504.5256082279507 | 40.0 | C |
| PAL1 | 2x | - | 2482.70684435934 | 15.0 | C |
| AT1G42440 | 2x | - | 5213.279399448493 | 26.0 | C |
| PPa6 | 2x | - | 15859.707906837424 | 58.0 | H |
| AT2G18220 | 2x | - | 1492.2253232365767 | 34.0 | C |
| AT2G24290 | 2x | - | 288.2639835432112 | 21.0 | C |
| emb2394 | 2x | - | 2872.9085557373246 | 32.0 | C |
| APE2 | 2x | - | 9052.961129344947 | 103.0 | H |
| AT5G52490 | 2x | - | 1533.4425440311934 | 29.0 | C |
| PWP2 | 2x | - | 2991.5441401861044 | 37.0 | C |
| AT3G02060 | 2x | - | 674.5978423021454 | 19.0 | C |
| EDA7 | 2x | - | 570.0589086928104 | 22.0 | C |
| PCN | 2x | - | 629.9873672993014 | 25.0 | C |
| AT5G02680 | 2x | - | 103.41080782254276 | 13.0 | C |
| PYD1 | 2x | - | 51702.49155763282 | 4.0 | B |
| ACD1 | 2x | 2x | 22592.0 | 2.0 | B |
| GAMMA-VPE | 2x | - | 16950.0 | 2.0 | B |
| FIB2 | 2x | - | 3651.584274567233 | 42.0 | C |
| RPS18 | 2x | - | 504.78400823964085 | 28.0 | C |
| FH | 2x | - | 11212.938433119229 | 19.0 | B |
| ISU3 | 2x | - | 1759.0402746006905 | 22.0 | C |
| rps2 | 2x | - | 799.0485043943545 | 21.0 | C |
| AT1G10490 | 2x | - | 3868.9105834994148 | 32.0 | C |
| AT2G18900 | 2x | - | 985.7150611356451 | 21.0 | C |
| RRF | 2x | - | 2188.1107342213304 | 28.0 | C |
| AT4G31520 | 2x | - | 73.82505832196473 | 22.0 | C |
| AT1G27461 | 2x | - | 9934.11013280613 | 30.0 | C |
| AT1G13160 | 2x | - | 2267.753659536261 | 36.0 | C |
| AT2G40360 | 2x | - | 990.5654613156742 | 32.0 | C |
| PUM24 | 2x | - | 2148.4563423508253 | 32.0 | C |
| RPL4 | 2x | - | 3058.384108579562 | 35.0 | C |
| AT5G09770 | 2x | - | 198.2952984108065 | 22.0 | C |
| AT4G19610 | 2x | - | 698.3579099186136 | 26.0 | C |
| AT5G14520 | 2x | - | 2808.8665365154716 | 32.0 | C |
| AT5G36160 | 2x | - | 8.753704690522465 | 6.0 | C |
| TAT7 | 2x | - | 21.93495127887086 | 6.0 | C |
| AT2G20060 | 2x | - | 1471.017757587453 | 30.0 | C |
| CPN60B | 2x | - | 71892.84789884416 | 81.0 | HB |
| emb1211 | 2x | - | 883.2472257362728 | 14.0 | C |
| AT5G05720 | 2x | - | 275.25063673463427 | 24.0 | C |
| AT3G54210 | 2x | - | 943.3691260694374 | 23.0 | C |
| emb2726 | 2x | 2x | 3928.599444333314 | 44.0 | C |
| AT5G15550 | 2x | - | 2105.070812756706 | 32.0 | C |
| At1g58380 | 2x | - | 77.52271166658683 | 15.0 | C |
| EMB3113 | 2x | 2x | 1437.369698573319 | 26.0 | C |
| EMB3136 | 2x | - | 1337.4141277380957 | 24.0 | C |
| EDA25 | 2x | - | 2716.8748887549473 | 24.0 | C |
| AT3G57940 | 2x | - | 4364.096075242233 | 34.0 | C |
| AT1G27470 | 2x | - | 455.8113690334568 | 34.0 | C |
| AT1G52930 | 2x | - | 4260.7495866085765 | 31.0 | C |
| AT5G66540 | 2x | - | 1102.9504559362745 | 26.0 | C |
| PDE338 | 2x | - | 5053.7542754514625 | 48.0 | C |
| emb1473 | 2x | - | 3115.181313434583 | 35.0 | C |
| FIB1 | 2x | - | 4784.114467299449 | 38.0 | C |
| CORI3 | 2x | - | 572.0895586473905 | 12.0 | C |
| AT2G34357 | 2x | - | 2240.536560572885 | 29.0 | C |
| AT2G21440 | 2x | - | 1701.526553862564 | 29.0 | C |
| AT3G23700 | 2x | - | 6963.997354668059 | 54.0 | H |
| AT4G04940 | 2x | - | 1833.8905940625693 | 31.0 | C |
| AT4G23590 | 2x | - | 418.01133864865824 | 12.0 | C |
| T22E16.170 | 2x | - | 1266.832823521596 | 31.0 | C |
| AT5G08695 | 2x | - | 61.4690481888364 | 21.0 | C |
| AT3G06530 | 2x | - | 442.62221477203155 | 24.0 | C |
| AT3G15460 | 2x | - | 5506.896443163233 | 34.0 | C |
| EMB2762 | 2x | - | 4268.719807390624 | 39.0 | C |
| AT5G14460 | 2x | - | 71934.40734093425 | 97.0 | HB |
| RABE1b | 2x | - | 1646.573628819666 | 24.0 | C |
| AT1G63810 | 2x | - | 1104.1983830204608 | 29.0 | C |
| AT4G02930 | 2x | - | 1996.1824094162835 | 26.0 | C |
| AT3G12370 | 2x | - | 443.0578094612443 | 28.0 | C |
| AT1G25260 | 2x | - | 6049.177316839354 | 53.0 | H |
| AT3G23620 | 2x | - | 735.3301592383376 | 27.0 | C |
| AT4G18905 | 2x | - | 798.0975708784365 | 24.0 | C |
| DIN10 | 2x | 2x | 0.0 | 1.0 | C |
| PHF1 | 2x | - | 0.0 | 1.0 | C |
| HExO3 | 2x | - | 11305.833333333336 | 3.0 | B |
| STL2P | 2x | - | 0.0 | 1.0 | C |
| STS | 2x | 2x | 14.983257678127705 | 4.0 | C |
| HAG4 | 2x | 2x | 416.59984396561265 | 6.0 | C |
| SIP2 | 2x | 2x | 19.329845875211937 | 4.0 | C |
| AT5G50650.1 | 2x | - | 0.0 | 1.0 | C |
| AT2G17930 | 2x | - | 638.4639785044945 | 8.0 | C |
| SWP | 2x | - | 2.2 | 2.0 | C |
| BSH | 2x | - | 48.96764869049888 | 3.0 | C |
| AT1G62660 | 2x | - | 14001.667596017825 | 8.0 | B |
| RFS1 | 2x | - | 9.425185421427027 | 3.0 | C |
| AT3G56310 | 2x | - | 18317.498104475053 | 8.0 | B |
| AT3G63460 | 2x | - | 14114.036242839891 | 4.0 | B |
| SAR2 | 2x | 2x | 16956.5 | 5.0 | B |
| cwINV4 | 2x | - | 16322.176682280642 | 8.0 | B |
| UBC14 | 2x | - | 6555.001401456778 | 42.0 | C |
| AT4G24440 | 2x | - | 93.46539468645994 | 6.0 | C |
| TAF14 | 2x | - | 188.9446266623685 | 7.0 | C |
| AT5G40880 | 2x | - | 5.323076923076921 | 5.0 | C |
| ALN | 2x | 2x | 0.0 | 2.0 | C |
| SKIP16 | 2x | - | 14.69174082373374 | 4.0 | C |
| ADA2A | 2x | - | 47.163578526736444 | 9.0 | C |
| AT4G36080 | 2x | - | 1298.8801209286485 | 10.0 | C |
| CARA | 2x | - | 90.75610527423181 | 5.0 | C |
| MDAR6 | 2x | - | 5928.712706762272 | 8.0 | C |
| CWINV5 | 2x | - | 4903.670230238312 | 8.0 | C |
| cwINV6 | 2x | - | 7667.332431023362 | 6.0 | C |
| HAG5 | 2x | - | 5.966945225381895 | 3.0 | C |
| AGAL2 | 2x | - | 46.876955588564584 | 6.0 | C |
| UBC13 | 2x | - | 19629.79224375323 | 43.0 | C |
| SAPx | 2x | 2x | 3.543064000758508 | 2.0 | C |
| ATMDAR2 | 2x | - | 186.36331705676517 | 3.0 | C |
| EP3 | 2x | 2x | 5654.0 | 2.0 | C |
| HExO1 | 2x | - | 0.0 | 1.0 | C |
| ADA2B | 2x | - | 5892.213854102417 | 11.0 | C |
| UBP22 | 2x | - | 25226.710797089567 | 8.0 | C |
| MDAR4 | 2x | - | 0.0 | 2.0 | C |
| GLDH | 2x | - | 21593.925533717975 | 9.0 | B |
| GAS41 | 2x | - | 2345.109622308371 | 9.0 | C |
| AT1G18830 | 2x | - | 14114.036242839891 | 4.0 | B |
| BS14A | 2x | - | 0.5 | 2.0 | C |
| PSAE-1 | 2x | - | 5005.942903724846 | 104.0 | H |
| AGAL1 | 2x | - | 4322.089521320987 | 5.0 | B |
| AT4G03460 | 2x | 2x | 0.0 | 1.0 | C |
| ELO3 | 2x | - | 27671.904613314207 | 7.0 | C |
| AT3G61870 | 2x | - | 175.06484140493984 | 35.0 | C |
| PSAD-2 | 2x | - | 980.9295266093432 | 102.0 | H |
| AT1G66430 | 2x | - | 3052.6625817405666 | 11.0 | C |
| GAPA-2 | 2x | - | 13624.862760254111 | 100.0 | HB |
| LHCB4.1 | 2x | - | 3228.4105878966357 | 94.0 | H |
| ATBFRUCT1 | 2x | - | 4867.618041023563 | 10.0 | C |
| RFS5 | 2x | 2x | 0.75 | 2.0 | C |
| HAG1 | 2x | 2x | 1168.8059822673135 | 9.0 | C |
| PSBO1 | 2x | - | 326.90462975212245 | 93.0 | H |
| PSBx | 2x | - | 468.5719909988956 | 87.0 | H |
| SGF29b | 2x | - | 25347.615534694713 | 9.0 | C |
| PSBQ-2 | 2x | - | 2634.6326933645073 | 94.0 | H |
| SGF29a | 2x | - | 343.59530242254954 | 8.0 | C |
| FBA1 | 2x | - | 605.1980774714997 | 58.0 | H |
| LHCA1 | 2x | 2x | 624.6424549266114 | 97.0 | H |
| PSBY | 2x | 2x | 298.15360009773536 | 93.0 | H |
| LHCA2 | 2x | - | 328.5531576867859 | 92.0 | H |
| PSBO2 | 2x | 2x | 244.16013051152038 | 90.0 | H |
| LHCB3 | 2x | - | 2618.764899989206 | 92.0 | H |
| CAB3 | 2x | - | 105.73979239200487 | 64.0 | H |
| PETE1 | 2x | - | 376.5266796818823 | 88.0 | H |
| PSAD-1 | 2x | - | 11135.65001393404 | 109.0 | HB |
| AT1G51400 | 2x | - | 70.1925220270346 | 84.0 | H |
| AT5G58575 | 2x | - | 48.546257309920016 | 8.0 | C |
| PSAA | 2x | - | 1272.8397001952762 | 47.0 | C |
| LHCB6 | 2x | 2x | 940.1339584273611 | 90.0 | H |
| LHCA4 | 2x | - | 2801.3710114228575 | 85.0 | H |
| PSBTN | 2x | - | 217.85235826146783 | 87.0 | H |
| GAPA | 2x | 2x | 23183.905750100355 | 126.0 | HB |
| PSAG | 2x | - | 827.7469341533782 | 94.0 | H |
| LHCA3 | 2x | - | 505.2868325227985 | 97.0 | H |
| AT5G49200 | 2x | - | 7.364622978647369 | 5.0 | C |
| LHCB4.2 | 2x | - | 115.4140395499783 | 80.0 | H |
| CRD1 | 2x | 2x | 377.1135203407685 | 91.0 | H |
| PSAE-2 | 2x | - | 1051.338964260445 | 100.0 | H |
| UBC7 | 2x | - | 19943.60466745506 | 46.0 | C |
| AT1G65230 | 2x | - | 516.8296417534575 | 85.0 | H |
| LHB1B1 | 2x | - | 74.89402272280576 | 66.0 | H |
| PSAH-1 | 2x | - | 390.42552319264695 | 94.0 | H |
| LHCB5 | 2x | 2x | 7261.936280027752 | 98.0 | HB |
| PSAO | 2x | 2x | 14652.621183901485 | 97.0 | HB |
| PSAL | 2x | - | 502.47645295645384 | 103.0 | H |
| CaS | 2x | - | 18.820265234702003 | 34.0 | C |
| FNR1 | 2x | - | 34015.69149586518 | 136.0 | HB |
| PPL1 | 2x | - | 722.3134853945186 | 91.0 | H |
| PSBR | 2x | - | 48.83107602928029 | 70.0 | H |
| PP2AB2 | 2x | - | 5696.541028907499 | 8.0 | C |
| PP2AA2 | 2x | 2x | 0.0 | 1.0 | C |
| ABCE2 | 2x | - | 4.751792944521121 | 3.0 | C |
| ABCE1 | 2x | - | 0.0 | 1.0 | C |
| LBA1 | 2x | - | 437.27327506524955 | 15.0 | C |
| PP2AB1 | 2x | - | 5696.541028907499 | 8.0 | C |
| AT3G26020 | 2x | - | 0.0 | 1.0 | C |
| AT1G62410 | 2x | - | 30.533825123881147 | 22.0 | C |
| ERF1-2 | 2x | - | 418.7818226228689 | 15.0 | C |
| ABCE3 | 2x | - | 4.291836821558075 | 3.0 | C |
| UBP24 | 2x | - | 9932.001321533657 | 43.0 | C |
| Y14 | 2x | - | 667.5107635862583 | 15.0 | C |
| EIF4A-III | 2x | - | 125.87604634592688 | 20.0 | C |
| PAB8 | 2x | - | 105.45162137726314 | 21.0 | C |
| AT2G43460 | 2x | - | 134.83680759435663 | 33.0 | C |
| PAB5 | 2x | - | 9.91729444884222 | 16.0 | C |
| PAB3 | 2x | - | 135.2139171935589 | 19.0 | C |
| ERF1-3 | 2x | - | 35.24987282762241 | 18.0 | C |
| AT2G39260 | 2x | - | 27.63920180068216 | 17.0 | C |
| ERF1-1 | 2x | - | 75.84643229954284 | 21.0 | C |
| AT3G59540.1 | 2x | - | 171.12153308074448 | 35.0 | C |
| AT4G14320 | 2x | - | 159.98031041893782 | 37.0 | C |
| AT1G77932 | 2x | - | 148.0887850215544 | 31.0 | C |
| At1g43 | 2x | - | 0.0 | 1.0 | C |
| AT3G16650 | 2x | - | 327.41229543875477 | 86.0 | H |
| ABF4 | 2x | 2x | 0.0 | 1.0 | C |
| PGY2 | 2x | - | 133.146595889232 | 32.0 | C |
| AT1G33120 | 2x | - | 100.07753594118556 | 32.0 | C |
| AT1G48900 | 2x | - | 51.35573756939785 | 22.0 | C |
| AT2G47610 | 2x | - | 45184.457655027916 | 101.0 | H |
| ATHSRP54A | 2x | - | 41.39040134332341 | 20.0 | C |
| AT2G47570 | 2x | - | 295.0492719957042 | 37.0 | C |
| AT4G10450 | 2x | - | 286.6966902386338 | 44.0 | C |
| AT3G62870 | 2x | - | 45411.79274067113 | 101.0 | H |
| AT2G44120 | 2x | - | 34.848736899543766 | 12.0 | C |
| AT5G49500 | 2x | - | 49.28388146930471 | 20.0 | C |
| AT3G13580 | 2x | - | 278.5819430712359 | 21.0 | C |
| U2A | 2x | - | 8357.432140684703 | 134.0 | H |
| ANNAT3 | 2x | - | 0.0 | 1.0 | C |
| ABCG34 | 2x | 2x | 3186.8869964060473 | 4.0 | C |
| SERAT2;2 | 2x | - | 0.8815075729685281 | 3.0 | C |
| MAK10 | 2x | - | 0.0 | 1.0 | C |
| CBL | 2x | - | 98.79926347418109 | 10.0 | C |
| SERAT2;1 | 2x | - | 286.77521804916483 | 7.0 | C |
| AT1G33320 | 2x | - | 422.2887733628528 | 11.0 | C |
| LP1 | 2x | 2x | 0.0 | 2.0 | C |
| CAM1 | 2x | - | 0.0 | 2.0 | C |
| SERAT3;2 | 2x | - | 18.70316027759826 | 3.0 | C |
| xK-2 | 2x | - | 1311.7235433641495 | 5.0 | C |
| AT5G02080 | 2x | - | 1.9177745592829507 | 6.0 | C |
| AT3G55180 | 2x | - | 143.64240065830185 | 12.0 | C |
| AT5G14980 | 2x | - | 104.47134739209775 | 13.0 | C |
| AT4G09784 | 2x | - | 2.7444668235949594 | 4.0 | C |
| AT2G37500 | 2x | - | 125.73774250787805 | 19.0 | C |
| SERAT1;1 | 2x | 2x | 888.6695677415775 | 5.0 | C |
| SKL2 | 2x | 2x | 2.197591304678715 | 8.0 | C |
| ATSERAT3;1 | 2x | - | 0.8815075729685281 | 3.0 | C |
| MTO1 | 2x | - | 213.65369753392892 | 11.0 | C |
| HDA08 | 2x | - | 4.8786391158771405 | 6.0 | C |
| ATMAK3 | 2x | 2x | 5661.932790603237 | 5.0 | C |
| HDA18 | 2x | - | 4.8786391158771405 | 6.0 | C |
| AT1G18360 | 2x | - | 22.754625190422352 | 12.0 | C |
| AT3G61010 | 2x | - | 0.0 | 1.0 | C |
| AT2G39410 | 2x | - | 70.75211840596036 | 11.0 | C |
| AT5G16120 | 2x | - | 1884.650259724035 | 15.0 | C |
| AT2G41790 | 2x | 2x | 102.88442417587636 | 27.0 | C |
| CKS1 | 2x | - | 81023.28050383153 | 94.0 | HB |
| AT1G11090 | 2x | - | 77.24468141236417 | 13.0 | C |
| AT2G47630 | 2x | - | 175.8888726044181 | 14.0 | C |
| AT3G62860 | 2x | - | 105.43065870115026 | 14.0 | C |
| At3g03780 | 2x | - | 965.5849972361852 | 33.0 | C |
| AT1G77420 | 2x | - | 173.87852179088236 | 12.0 | C |
| AT3G55190 | 2x | - | 18.923985371188557 | 11.0 | C |
| ATMS1 | 2x | - | 2126.3858885191476 | 36.0 | C |
| NAGS2 | 2x | - | 1375.6153830328544 | 22.0 | C |
| HDA05 | 2x | - | 2.4138111230078727 | 4.0 | C |
| AT1G73480 | 2x | - | 2370.4914810669593 | 13.0 | C |
| AT1G52220 | 2x | - | 223.02993113482637 | 90.0 | H |
| ANNAT1 | 2x | - | 0.0 | 1.0 | C |
| ANNAT4 | 2x | 2x | 11306.0 | 3.0 | B |
| OASB | 2x | 2x | 17519.66600000262 | 39.0 | B |
| DELTA-OAT | 2x | - | 7304.836347273107 | 39.0 | C |
| AT2G37660 | 2x | - | 61.962726438465 | 36.0 | C |
| HDA14 | 2x | - | 297.281599708639 | 19.0 | C |
| WIN1 | 2x | - | 4.270873602029825 | 8.0 | C |
| AT5G16280 | 2x | - | 228.55315527407006 | 30.0 | C |
| ASN2 | 2x | - | 2487.10028415082 | 45.0 | C |
| LysoPL2 | 2x | - | 9586.82600075694 | 17.0 | B |
| AT2G39400 | 2x | - | 174.28256219492278 | 13.0 | C |
| NAGS1 | 2x | - | 588.0145036882965 | 17.0 | C |
| AT1G33270 | 2x | - | 87.96396950060397 | 13.0 | C |
| AT5G19290 | 2x | - | 43224.12488078639 | 15.0 | B |
| EMB2753 | 2x | - | 2801.8385172761086 | 49.0 | C |
| AT2G39420 | 2x | 2x | 176.54189567369937 | 14.0 | C |
| AT5G11650 | 2x | - | 15584.25467115772 | 15.0 | B |
| AT5G58450 | 2x | - | 5076.8703720054145 | 34.0 | C |
| HDA15 | 2x | - | 0.12310606060606061 | 5.0 | C |
| P5CR | 2x | - | 702.5865887427682 | 37.0 | C |
| AT1G10520 | 2x | - | 3.757569985400776 | 22.0 | C |
| ASN1 | 2x | - | 1370.4484491064736 | 40.0 | C |
| FD2 | 2x | - | 48305.10353807791 | 167.0 | HB |
| MS3 | 2x | - | 1089.161840559851 | 30.0 | C |
| ASA2 | 2x | - | 0.0 | 1.0 | C |
| AT1G25083.1 | 2x | - | 10311.808448227977 | 39.0 | B |
| CM3 | 2x | - | 109.67228633205022 | 2.0 | C |
| CM1 | 2x | - | 5654.0 | 2.0 | C |
| GPx1 | 2x | - | 0.0 | 1.0 | C |
| AT5G27410 | 2x | - | 0.0 | 1.0 | C |
| AT1G24909 | 2x | - | 12109.206787721678 | 39.0 | B |
| PEx5 | 2x | - | 0.0 | 1.0 | C |
| AT1G70570 | 2x | - | 0.0 | 2.0 | C |
| DIC3 | 2x | - | 0.0 | 1.0 | C |
| ACLA-1 | 2x | - | 54.68824297606503 | 3.0 | C |
| AT1G24807 | 2x | - | 16099.473278014551 | 40.0 | B |
| CM2 | 2x | - | 115.27774874455116 | 3.0 | C |
| GPx5 | 2x | - | 4.0 | 2.0 | C |
| AT5G19760 | 2x | - | 0.0 | 1.0 | C |
| AT3G55870 | 2x | - | 1827.7297512809428 | 2.0 | C |
| ADCL | 2x | - | 10.283483625256927 | 2.0 | C |
| HA5 | 2x | 2x | 0.06060606060606061 | 2.0 | C |
| UCP5 | 2x | - | 2303.0344310172018 | 22.0 | C |
| GPx8 | 2x | - | 0.0 | 1.0 | C |
| AT2G11270 | 2x | - | 86066.94129209836 | 56.0 | HB |
| DIC2 | 2x | - | 0.0 | 1.0 | C |
| AT2G04400 | 2x | - | 7501.098020549573 | 3.0 | B |
| ASA1 | 2x | - | 26.195746777905338 | 3.0 | C |
| GPx3 | 2x | - | 0.0 | 1.0 | C |
| GPx4 | 2x | - | 16905.0 | 3.0 | C |
| pat1 | 2x | - | 0.20887119777387558 | 2.0 | C |
| GPx2 | 2x | - | 45095.0 | 5.0 | C |
| BCAT3 | 2x | - | 2.801392109485375 | 2.0 | C |
| AT1G25155.1 | 2x | - | 11671.254821474724 | 41.0 | B |
| EMB1144 | 2x | - | 7654.942731632417 | 6.0 | B |
| AT2G27720 | 2x | 2x | 9.407755452849194 | 13.0 | C |
| AT1G74780 | 2x | 2x | 0.0 | 2.0 | C |
| IAA32 | 2x | - | 0.0 | 1.0 | C |
| AT3G61113 | 2x | - | 0.0 | 2.0 | C |
| BI1 | 2x | - | 0.0 | 1.0 | C |
| IAA1 | 2x | - | 2180.7267665288327 | 6.0 | C |
| AT1G30580 | 2x | - | 425.3236809467058 | 9.0 | C |
| MSD1 | 2x | - | 234.79165270920072 | 9.0 | C |
| ATSUV3 | 2x | - | 0.0 | 2.0 | C |
| AT3G16830.1 | 2x | - | 40.88618806163557 | 4.0 | C |
| MDHAR | 2x | - | 372.73909289833637 | 4.0 | C |
| AT3G56350 | 2x | - | 362.94719014375505 | 6.0 | C |
| VPS60.2 | 2x | - | 0.65 | 5.0 | C |
| FSD3 | 2x | - | 210.29746310721555 | 9.0 | C |
| SKD1 | 2x | - | 2493.4867499879583 | 9.0 | C |
| AT1G48970 | 2x | - | 1310.7393806215625 | 15.0 | C |
| AT5G65740 | 2x | - | 35.0324222820929 | 11.0 | C |
| STI | 2x | - | 0.0 | 12.0 | C |
| AT1G80630 | 2x | 2x | 6015.78998857832 | 9.0 | C |
| AT1G14460 | 2x | - | 0.0 | 12.0 | C |
| AT1G53880 | 2x | - | 0.0 | 12.0 | C |
| NBS1 | 2x | - | 6.584105620986955 | 15.0 | C |
| AT3G07300 | 2x | - | 1310.7393806215625 | 15.0 | C |
| PRT6 | 2x | - | 0.0 | 3.0 | C |
| AT2G34970 | 2x | - | 1310.7393806215625 | 15.0 | C |
| DAD1 | 2x | - | 593.9402764417456 | 6.0 | C |
| OPCL1 | 2x | - | 327.0368866924635 | 21.0 | C |
| AT2G45695 | 2x | - | 0.0 | 2.0 | C |
| RLP14 | 2x | 2x | 18.69290205530474 | 3.0 | C |
| CTF18 | 2x | - | 3667.37140345813 | 41.0 | C |
| AT4G24380 | 2x | - | 327.0368866924635 | 21.0 | C |
| AT2G46520 | 2x | - | 40.986794739521976 | 7.0 | C |
| AT4G18300 | 2x | - | 1310.7393806215625 | 15.0 | C |
| AT1G52530 | 2x | - | 41.15698793877203 | 32.0 | C |
| HPL1 | 2x | 2x | 0.0 | 3.0 | C |
| AT1G08270 | 2x | - | 1777.3967635866522 | 8.0 | C |
| DCL1 | 2x | - | 4477.0598851176865 | 30.0 | C |
| AT4G00651 | 2x | - | 72.88064719954559 | 4.0 | C |
| POLA3 | 2x | - | 24.55279571712713 | 29.0 | C |
| MEB2 | 2x | - | 0.0 | 1.0 | C |
| CER7 | 2x | - | 3.318754598749934 | 16.0 | C |
| IAA29 | 2x | - | 0.0 | 2.0 | C |
| AT4G24790 | 2x | - | 0.0 | 12.0 | C |
| FSD1 | 2x | - | 339.12824584246766 | 6.0 | C |
| AT2G25355 | 2x | - | 756.410036812798 | 23.0 | C |
| VAB2 | 2x | - | 6080.851391448514 | 43.0 | C |
| AT4G32175 | 2x | - | 554.1623467494923 | 22.0 | C |
| LOx5 | 2x | - | 861.9254683044161 | 9.0 | C |
| WRKY40 | 2x | - | 25.23723015140212 | 21.0 | C |
| AOS | 2x | 2x | 27.460377932875723 | 9.0 | C |
| AT4G14970 | 2x | - | 26.278922214506935 | 31.0 | C |
| AT4G18820 | 2x | - | 0.0 | 12.0 | C |
| AT1G07270 | 2x | - | 18834.135007626337 | 78.0 | HB |
| VHA-A2 | 2x | - | 1018.77553375243 | 37.0 | C |
| MDAR1 | 2x | - | 0.0 | 2.0 | C |
| SCL13 | 2x | - | 0.35882636594896733 | 20.0 | C |
| VHA-A1 | 2x | - | 1018.77553375243 | 37.0 | C |
| LOx3 | 2x | 2x | 6466.703201593312 | 31.0 | C |
| PLA2-ALPHA | 2x | - | 1.3555555555555556 | 6.0 | C |
| WRKY18 | 2x | - | 25.23723015140212 | 21.0 | C |
| AT5G45720 | 2x | - | 0.0 | 12.0 | C |
| AT3G28715 | 2x | - | 1398.6525839607148 | 37.0 | C |
| AT5G01940 | 2x | - | 1310.7393806215625 | 15.0 | C |
| AT5G25080 | 2x | - | 178.74806106589912 | 17.0 | C |
| RPA32B | 2x | - | 225.00505331967184 | 52.0 | H |
| LOx1 | 2x | - | 890.1141163752823 | 10.0 | C |
| VATG3 | 2x | - | 87.0264734575094 | 35.0 | C |
| AT1G12470 | 2x | 2x | 22635.299965507522 | 6.0 | C |
| MCM9 | 2x | - | 3363.5929867493983 | 44.0 | C |
| AT2G26500 | 2x | - | 9.524218630280236 | 77.0 | H |
| PB | 2x | - | 23015.413979716956 | 94.0 | HB |
| VPS46.2 | 2x | - | 15.263098051922379 | 5.0 | C |
| VHA-E2 | 2x | - | 87.0264734575094 | 35.0 | C |
| AT1G77680 | 2x | 2x | 22382.97296287328 | 33.0 | B |
| RAN4 | 2x | - | 1057.221844300385 | 6.0 | C |
| VPS60.1 | 2x | - | 3912.7517851185494 | 17.0 | C |
| DOx1 | 2x | - | 594.2736097750791 | 7.0 | C |
| VAG2 | 2x | - | 288.1360177491718 | 36.0 | C |
| AT3G07750 | 2x | - | 85.55723323759527 | 17.0 | C |
| AT3G46210 | 2x | - | 309.93771952396276 | 18.0 | C |
| AT3G02840 | 2x | - | 25.23723015140212 | 21.0 | C |
| AT2G25610 | 2x | - | 48.18502242523529 | 27.0 | C |
| PPa4 | 2x | - | 1050.1442195168904 | 32.0 | C |
| DCL3 | 2x | - | 4477.0598851176865 | 30.0 | C |
| ARF17 | 2x | 2x | 9190.766655354399 | 8.0 | B |
| EMB1401 | 2x | - | 25155.488421788483 | 56.0 | HB |
| AT5G49110 | 2x | - | 2.3907718297962197 | 13.0 | C |
| EMB1968 | 2x | - | 14993.3674850998 | 66.0 | HB |
| AT2G42220 | 2x | - | 49.55871574419793 | 79.0 | H |
| LOx4 | 2x | 2x | 6697.841777583145 | 33.0 | C |
| SZF1 | 2x | - | 1186.4672100523792 | 21.0 | C |
| PSAB | 2x | - | 308.56932609122396 | 40.0 | C |
| AT5G19485 | 2x | - | 1310.7393806215625 | 15.0 | C |
| AVA-P1 | 2x | - | 55.20756331376413 | 24.0 | C |
| AOC3 | 2x | - | 613.8634963670704 | 23.0 | C |
| RRP41 | 2x | - | 3.318754598749934 | 16.0 | C |
| AT3G02820 | 2x | - | 28.959056312563145 | 38.0 | C |
| VMA10 | 2x | - | 275.31293837440444 | 38.0 | C |
| ATPH | 2x | - | 1340.834266277416 | 62.0 | H |
| CPK28 | 2x | - | 25.23723015140212 | 21.0 | C |
| AT5G38890 | 2x | - | 3.318754598749934 | 16.0 | C |
| YCF3 | 2x | - | 2.685681218596983 | 15.0 | C |
| LHCB2.1 | 2x | 2x | 921.9107514253527 | 37.0 | C |
| POLD2 | 2x | - | 1252.5424912025414 | 22.0 | C |
| MEI1 | 2x | - | 164.2620087997577 | 40.0 | C |
| RFC2 | 2x | - | 3314.0881230789023 | 63.0 | H |
| AT5G08690 | 2x | - | 21170.99032255002 | 93.0 | HB |
| AT4G29780 | 2x | - | 0.35882636594896733 | 20.0 | C |
| AT4G32530 | 2x | - | 87.12658606508023 | 30.0 | C |
| JAZ5 | 2x | - | 1256.0335720813866 | 25.0 | C |
| BCS1 | 2x | 2x | 7651.7100540884285 | 23.0 | C |
| RRP4 | 2x | - | 600.9234558539645 | 23.0 | C |
| AT5G39840 | 2x | - | 0.0 | 2.0 | C |
| AT1G72340 | 2x | - | 0.0 | 12.0 | C |
| AT3G07920 | 2x | - | 1310.7393806215625 | 15.0 | C |
| AVA-P2 | 2x | - | 2582.1552455991223 | 59.0 | H |
| Pnsl5 | 2x | - | 571.4973757193134 | 29.0 | C |
| ATIM | 2x | - | 16.787867120442858 | 33.0 | C |
| TIFY4A | 2x | - | 0.0 | 8.0 | C |
| AT1G56050 | 2x | - | 539.3254733725547 | 20.0 | C |
| POLD4 | 2x | - | 315.0660948508708 | 20.0 | C |
| POLA2 | 2x | - | 56.822378177812276 | 43.0 | C |
| ATR | 2x | - | 7904.440029468632 | 48.0 | C |
| EMB2780 | 2x | - | 2975.1422283929955 | 49.0 | C |
| AT2G16510.1 | 2x | - | 47.363271478431244 | 21.0 | C |
| ROC4 | 2x | - | 3710.827755183356 | 61.0 | H |
| PSAC | 2x | - | 116.42084341121294 | 34.0 | C |
| SOS2 | 2x | - | 1643.7654633860107 | 15.0 | C |
| RRP45a | 2x | - | 3403.8990015087943 | 20.0 | C |
| RRP41L | 2x | - | 0.3606060606060606 | 14.0 | C |
| LIG1 | 2x | - | 2613.6938791881794 | 27.0 | C |
| VHA-E3 | 2x | - | 1348.197836921734 | 36.0 | C |
| FdC2 | 2x | - | 23697.684383186534 | 139.0 | HB |
| VHA-C3 | 2x | - | 586.7143259209316 | 60.0 | H |
| VHA-A3 | 2x | - | 1092.860305606565 | 38.0 | C |
| DCL2 | 2x | - | 4477.0598851176865 | 30.0 | C |
| AT4G02110 | 2x | - | 211.45332633849375 | 43.0 | C |
| AT5G10630 | 2x | - | 9523.672264737532 | 18.0 | B |
| RFC1 | 2x | - | 3719.076021493117 | 41.0 | C |
| ERF11 | 2x | - | 159.3050950153473 | 22.0 | C |
| AT1G53900.1 | 2x | - | 0.0 | 12.0 | C |
| AT5G38640 | 2x | - | 1781.1899655914183 | 16.0 | C |
| PRL | 2x | - | 2424.8776431955525 | 42.0 | C |
| VPS46.1 | 2x | - | 15.263098051922379 | 5.0 | C |
| AT2G14830 | 2x | 2x | 10609.13738482119 | 25.0 | B |
| GUN4 | 2x | - | 267.74154799827323 | 77.0 | H |
| RPA2 | 2x | - | 627.9782051774906 | 55.0 | H |
| AT5G19940 | 2x | - | 9.741891001857669 | 75.0 | H |
| AT2G44070 | 2x | - | 1310.7393806215625 | 15.0 | C |
| AT3G02270 | 2x | - | 1310.7393806215625 | 15.0 | C |
| AT3G28710 | 2x | - | 1305.6293771004534 | 29.0 | C |
| TUF | 2x | - | 165.41384689934497 | 37.0 | C |
| AT1G49250 | 2x | - | 1022.0512252194688 | 25.0 | C |
| EMB2775 | 2x | - | 8411.179423569973 | 61.0 | H |
| FSD2 | 2x | 2x | 2752.0609322532646 | 14.0 | C |
| JAZ1 | 2x | - | 363.8161270673426 | 22.0 | C |
| WRKY33 | 2x | - | 19.62063709087697 | 20.0 | C |
| MCM2 | 2x | - | 3560.9048146731875 | 45.0 | C |
| AT3G04780 | 2x | - | 1247.2565795263602 | 7.0 | C |
| AT5G08670 | 2x | - | 13636.867608526625 | 72.0 | HB |
| AT5G02160 | 2x | - | 7.561086210708747 | 68.0 | H |
| AT5G35170 | 2x | - | 2161.6247140721603 | 66.0 | H |
| THM1 | 2x | - | 3549.0229186538972 | 79.0 | H |
| emb2184 | 2x | - | 309.1334812417378 | 79.0 | H |
| PSRP3/1 | 2x | - | 190.01816222591626 | 77.0 | H |
| PPa3 | 2x | - | 1050.1442195168904 | 33.0 | C |
| AT5G08680 | 2x | - | 19001.041138027787 | 92.0 | HB |
| TAPx | 2x | 2x | 9269.572092698781 | 82.0 | HB |
| T5M16.6 | 2x | 2x | 8515.138168134867 | 62.0 | H |
| AT2G39650 | 2x | - | 25.23723015140212 | 21.0 | C |
| TRx-M4 | 2x | - | 1266.2755391463506 | 80.0 | H |
| AVA-P4 | 2x | - | 12202.068984379719 | 60.0 | HB |
| AT1G75880 | 2x | 2x | 5654.0 | 2.0 | C |
| DCL4 | 2x | - | 4477.0598851176865 | 30.0 | C |
| RAD9 | 2x | - | 42.54805771560177 | 33.0 | C |
| AT1G67700 | 2x | - | 4743.164597279176 | 77.0 | H |
| POLD3 | 2x | - | 1.7281962886745312 | 22.0 | C |
| ATRAD17 | 2x | - | 621.7713843468615 | 57.0 | H |
| VAB3 | 2x | - | 5888.925396844689 | 41.0 | C |
| AT2G05720 | 2x | - | 1850.432738689046 | 87.0 | H |
| SMG7 | 2x | - | 0.0 | 1.0 | C |
| EDA9 | 2x | - | 0.0 | 1.0 | C |
| RS31 | 2x | - | 0.0 | 1.0 | C |
| AT2G29210 | 2x | - | 0.0 | 1.0 | C |
| AT5G10960 | 2x | - | 0.0 | 1.0 | C |
| EMB2754 | 2x | - | 16950.0 | 2.0 | C |
| AT1G66500 | 2x | - | 0.0 | 1.0 | C |
| ATVPS33 | 2x | - | 0.0 | 1.0 | C |
| ATSYP24 | 2x | - | 11306.0 | 3.0 | C |
| VCL1 | 2x | - | 0.0 | 1.0 | C |
| CPSF73-I | 2x | - | 0.0 | 1.0 | C |
| AT2G33410 | 2x | - | 0.0 | 1.0 | C |
| SCL30 | 2x | - | 0.0 | 1.0 | C |
| AtRZ-1c | 2x | - | 0.0 | 1.0 | C |
| AT1G28180 | 2x | - | 89.65181255285088 | 51.0 | H |
| TAF15 | 2x | - | 5.25614952452208 | 2.0 | C |
| AT1G07170 | 2x | - | 0.0 | 1.0 | C |
| emb1579 | 2x | - | 5.679078407572875 | 3.0 | C |
| AT1G14650 | 2x | - | 1194.88566880276 | 83.0 | H |
| AT1G14640 | 2x | - | 4099.55805771434 | 83.0 | H |
| AT2G33730 | 2x | - | 4.075601449735738 | 50.0 | C |
| AHP1 | 2x | - | 1241.0654919594506 | 21.0 | C |
| AHP3 | 2x | - | 1241.0654919594506 | 21.0 | C |
| AT4G37230 | 2x | - | 0.0 | 1.0 | C |
| RBOHD | 2x | - | 0.0 | 1.0 | C |
| AT3G61260 | 2x | 2x | 0.0 | 1.0 | C |
| PYL2 | 2x | - | 0.0 | 2.0 | C |
| AT3G17020 | 2x | 2x | 0.0 | 1.0 | C |
| CCH | 2x | - | 11.199999999999992 | 8.0 | C |
| RCAR3 | 2x | - | 1732.076491998471 | 3.0 | C |
| AT3G46710 | 2x | 2x | 0.0 | 2.0 | C |
| MUO10.6 | 2x | - | 1232.8440200979765 | 20.0 | C |
| PSBA | 2x | - | 6.3275719117407085 | 4.0 | C |
| ISA1 | 2x | - | 0.0 | 2.0 | C |
| PYL3 | 2x | - | 0.0 | 2.0 | C |
| HMA5 | 2x | - | 315.5257829097315 | 5.0 | C |
| PSBC | 2x | - | 292.74840693327997 | 4.0 | C |
| HMA4 | 2x | - | 315.5257829097315 | 5.0 | C |
| RHD2 | 2x | - | 0.0 | 1.0 | C |
| PYL4 | 2x | - | 0.0 | 2.0 | C |
| AT5G02420 | 2x | - | 0.04195240016135539 | 4.0 | C |
| CIPK26 | 2x | - | 227.16973841668266 | 3.0 | C |
| PMT5 | 2x | 2x | 0.0 | 3.0 | C |
| RBOHA | 2x | - | 0.0 | 1.0 | C |
| T18K17.8 | 2x | - | 2.906083107163918 | 13.0 | C |
| AHP5 | 2x | - | 1241.0654919594506 | 21.0 | C |
| ARR3 | 2x | - | 0.5857142857142856 | 5.0 | C |
| RCAR1 | 2x | - | 0.0 | 2.0 | C |
| AT1G07500 | 2x | - | 0.0 | 2.0 | C |
| TIP2;2 | 2x | - | 0.0 | 4.0 | C |
| NIP5;1 | 2x | - | 0.0 | 3.0 | C |
| NIP4;1 | 2x | - | 0.0 | 3.0 | C |
| HMA3 | 2x | - | 315.5257829097315 | 5.0 | C |
| NIP1;2 | 2x | - | 0.0 | 3.0 | C |
| PIP2;4 | 2x | - | 203.46842129343253 | 6.0 | C |
| GCP2 | 2x | - | 466.41469797563803 | 15.0 | C |
| PYR1 | 2x | - | 2914.1226207081368 | 5.0 | C |
| CTF7 | 2x | - | 15.90950591805868 | 6.0 | C |
| PSBB | 2x | - | 641.5980444269272 | 4.0 | C |
| CDKD1;1 | 2x | - | 59.955511771184405 | 16.0 | C |
| AT2G07687.1 | 2x | - | 200.985983183352 | 31.0 | C |
| AT5G17410 | 2x | - | 47.79529050628508 | 8.0 | C |
| AT4G14310 | 2x | - | 124.35888951472656 | 7.0 | C |
| T6J19.6 | 2x | - | 11.199999999999992 | 8.0 | C |
| MAP65-2 | 2x | - | 62.7453573377639 | 10.0 | C |
| PIP1;5 | 2x | - | 0.5621621621621622 | 6.0 | C |
| PTAC16 | 2x | - | 0.0 | 1.0 | C |
| OZS1 | 2x | - | 86.28733868926263 | 5.0 | C |
| SNRK2-8 | 2x | - | 113.01266657651232 | 3.0 | C |
| IDH1 | 2x | - | 352.116119338963 | 16.0 | C |
| PSBD | 2x | - | 6.3275719117407085 | 4.0 | C |
| RBOHF | 2x | - | 33.68101669351602 | 3.0 | C |
| CPK23 | 2x | 2x | 16973.71021111013 | 6.0 | B |
| PLE | 2x | - | 122.43001162131405 | 11.0 | C |
| SQD1 | 2x | - | 10.666763313890456 | 14.0 | C |
| PETA | 2x | - | 5.0766512805911335 | 9.0 | C |
| AT1G09995 | 2x | - | 193.62249421073605 | 17.0 | C |
| HMA2 | 2x | - | 14705.655138833336 | 6.0 | B |
| RBP45A | 2x | - | 455.2913799901275 | 4.0 | C |
| DAN1 | 2x | - | 0.0 | 3.0 | C |
| GAE2 | 2x | - | 2.906083107163918 | 20.0 | C |
| KRP4 | 2x | - | 301.7896883568386 | 22.0 | C |
| AT1G79890 | 2x | - | 4402.441452227893 | 18.0 | C |
| SIP1;2 | 2x | - | 0.0 | 3.0 | C |
| RPL22 | 2x | - | 0.7844282914341373 | 11.0 | C |
| NIP4;2 | 2x | - | 0.0 | 3.0 | C |
| AT4G28360 | 2x | - | 0.7844282914341373 | 11.0 | C |
| CYCD3;2 | 2x | - | 46.61883940539295 | 15.0 | C |
| ATPE | 2x | - | 1274.6995436143345 | 23.0 | C |
| ICK6 | 2x | - | 138.62799956709247 | 19.0 | C |
| PETB | 2x | - | 60.509367617099464 | 28.0 | C |
| ORC2 | 2x | - | 28.211147318690607 | 9.0 | C |
| GME | 2x | - | 2.906083107163918 | 13.0 | C |
| DEG1 | 2x | 2x | 21907.515952007783 | 13.0 | B |
| CP29 | 2x | - | 1123.7117587086757 | 2.0 | C |
| RHD1 | 2x | - | 9.084945132072278 | 10.0 | C |
| UGE5 | 2x | - | 9.084945132072278 | 11.0 | C |
| UGE2 | 2x | - | 9.266763313890461 | 12.0 | C |
| AT2G01090 | 2x | - | 7.300905580383058 | 26.0 | C |
| PETG | 2x | - | 4.269047292116341 | 8.0 | C |
| cob | 2x | - | 87.37069325756087 | 28.0 | C |
| ICK1 | 2x | - | 28.667740627981157 | 19.0 | C |
| AT5G12410 | 2x | - | 0.21052631578947367 | 4.0 | C |
| EB1C | 2x | - | 1016.3581404680277 | 19.0 | C |
| AT3G15640 | 2x | - | 8.07491466249984 | 27.0 | C |
| T6G21 | 2x | - | 315.5257829097315 | 5.0 | C |
| AT1G51650 | 2x | 4x | 1426.9096694454004 | 25.0 | C |
| IDH-V | 2x | - | 10.736554504034476 | 16.0 | C |
| AT2G07727.1 | 2x | - | 60.509367617099464 | 27.0 | C |
| ATPF | 2x | - | 3.390941383426937 | 22.0 | C |
| COx1 | 2x | - | 13.100597799286584 | 31.0 | C |
| AT1G51980 | 2x | - | 11698.373392597774 | 32.0 | B |
| CYCD6;1 | 2x | - | 63.04993942500038 | 20.0 | C |
| ATPI | 2x | - | 2438.163575305779 | 25.0 | C |
| CYCA3;3 | 2x | - | 2471.380595681053 | 35.0 | C |
| AT1G10690 | 2x | - | 0.9185220293625636 | 7.0 | C |
| UGD1 | 2x | - | 1769.7207466904586 | 29.0 | C |
| IDH-III | 2x | - | 10.296994064474035 | 15.0 | C |
| EB1B | 2x | - | 1311.739056870822 | 22.0 | C |
| AT3G52730 | 2x | - | 13.100597799286584 | 31.0 | C |
| GAE4 | 2x | - | 2.906083107163918 | 20.0 | C |
| CYC1BAT | 2x | - | 514.8894388294773 | 30.0 | C |
| CYCD1;1 | 2x | - | 20.914156574127478 | 15.0 | C |
| AT3G27240 | 2x | - | 8883.660662268494 | 36.0 | C |
| AT5G13430 | 2x | - | 15531.242864445056 | 33.0 | B |
| ATPQ | 2x | - | 1274.6995436143345 | 23.0 | C |
| ATP3 | 2x | - | 3.390941383426937 | 22.0 | C |
| PYL1 | 2x | - | 0.0 | 3.0 | C |
| ATPC2 | 2x | - | 3.390941383426937 | 22.0 | C |
| ARR4 | 2x | - | 6818.541204477588 | 7.0 | C |
| PHYB | 2x | - | 20081.02546284166 | 5.0 | B |
| AT4G37830 | 2x | - | 8.297136884722063 | 29.0 | C |
| PYL6 | 2x | - | 223.3808036632025 | 4.0 | C |
| AT1G15120 | 2x | - | 7.300905580383058 | 26.0 | C |
| HMA1 | 2x | - | 315.5257829097315 | 5.0 | C |
| AT5G19090 | 2x | 2x | 11.199999999999992 | 8.0 | C |
| CYCD4;2 | 2x | - | 62.44250357666847 | 19.0 | C |
| MPPBETA | 2x | - | 17261.07552972762 | 35.0 | B |
| ICK5 | 2x | - | 29.129100060306808 | 15.0 | C |
| CYCD4;1 | 2x | - | 83.73973948455472 | 21.0 | C |
| CYCD2;1 | 2x | - | 653.3675365447758 | 25.0 | C |
| GAE6 | 2x | - | 2.906083107163918 | 20.0 | C |
| CAT7 | 2x | 2x | 53.414623144160856 | 29.0 | C |
| F17M5.280 | 2x | - | 315.5257829097315 | 5.0 | C |
| COx6B | 2x | - | 4.849843437428617 | 20.0 | C |
| ATPA | 2x | - | 56214.228491340946 | 59.0 | HB |
| TUBG1 | 2x | - | 965.8236925092698 | 16.0 | C |
| COx3 | 2x | - | 200.985983183352 | 31.0 | C |
| AT4G32470 | 2x | - | 13.100597799286584 | 31.0 | C |
| TIP2;3 | 2x | - | 0.0 | 3.0 | C |
| AxS1 | 2x | - | 4291.8316972294415 | 21.0 | C |
| AT1G52710 | 2x | - | 8.07491466249984 | 27.0 | C |
| UDG4 | 2x | - | 277.4083971042909 | 30.0 | C |
| AT5G56730 | 2x | 2x | 439.2494065155389 | 28.0 | C |
| AT5G57815 | 2x | - | 4.849843437428617 | 20.0 | C |
| AT1G74910 | 2x | - | 5900.719654744374 | 27.0 | C |
| GAE5 | 2x | - | 2.906083107163918 | 20.0 | C |
| AT5G47030 | 2x | - | 505.28939131293527 | 27.0 | C |
| CYCA2;3 | 2x | - | 641.9191760245556 | 36.0 | C |
| PIP3 | 2x | - | 429.4538488741927 | 7.0 | C |
| TTN1 | 2x | - | 141.66115841651964 | 17.0 | C |
| AT5G23910 | 2x | - | 309.2339002884968 | 18.0 | C |
| CKS2 | 2x | - | 71518.33278361752 | 78.0 | HB |
| RAN1 | 2x | - | 315.5257829097315 | 5.0 | C |
| AxS2 | 2x | - | 2.906083107163918 | 20.0 | C |
| AT2G21300 | 2x | 2x | 0.3634674922600619 | 7.0 | C |
| MPPalpha | 2x | - | 439.2494065155389 | 30.0 | C |
| BPC2 | 2x | - | 18.715674305698048 | 5.0 | C |
| AT5G25450 | 2x | - | 13.100597799286584 | 31.0 | C |
| COx2 | 2x | - | 12504.048412822242 | 32.0 | B |
| CYT1 | 2x | - | 37039.87618027882 | 54.0 | HB |
| AT3G55590 | 2x | - | 37039.87618027882 | 54.0 | HB |
| FIB | 2x | - | 2.4105458570220635 | 4.0 | C |
| AT3G01010 | 2x | - | 277.19657444419227 | 27.0 | C |
| RHM3 | 2x | - | 391.1736664226776 | 28.0 | C |
| GAE1 | 2x | - | 2.906083107163918 | 20.0 | C |
| UGE3 | 2x | - | 9.084945132072278 | 10.0 | C |
| RHM1 | 2x | - | 272.59950692419363 | 27.0 | C |
| CDKD1;3 | 2x | - | 151.50491534901175 | 22.0 | C |
| IDH2 | 2x | - | 352.116119338963 | 16.0 | C |
| CYCD7;1 | 2x | - | 59.52332533026682 | 12.0 | C |
| AT5G02220 | 2x | - | 1.8583768528117146 | 6.0 | C |
| atp6-1 | 2x | - | 217.2140214951446 | 24.0 | C |
| ATP6-2 | 2x | - | 217.2140214951446 | 24.0 | C |
| PETD | 2x | - | 60.509367617099464 | 28.0 | C |
| AT5G40810 | 2x | - | 4721.139493333052 | 35.0 | C |
| COx15 | 2x | - | 0.0 | 3.0 | C |
| UGD2 | 2x | - | 1769.7207466904586 | 29.0 | C |
| GAE3 | 2x | - | 2.906083107163918 | 20.0 | C |
| AT4G28060 | 2x | - | 4.849843437428617 | 20.0 | C |
| CYCA3;4 | 2x | - | 1633.8166060825197 | 41.0 | C |
| AT1G52370 | 2x | - | 0.7844282914341373 | 11.0 | C |
| ICK3 | 2x | - | 11.334139479871608 | 11.0 | C |
| CYCD5;1 | 2x | - | 116.08019031401325 | 23.0 | C |
| CYCA3;1 | 2x | - | 1380.5246179182582 | 40.0 | C |
| KRP6 | 2x | - | 62.51178130344341 | 16.0 | C |
| CYCD3;1 | 2x | - | 85.53434170631897 | 16.0 | C |
| ATP6-1 | 2x | - | 217.2140214951446 | 24.0 | C |
| UGE1 | 2x | - | 9.084945132072278 | 11.0 | C |
| ARA1 | 2x | - | 11058.004246276663 | 9.0 | B |
| MUM4 | 2x | - | 14838.400625634778 | 28.0 | B |
| AT4G30470 | 2x | 2x | 2.906083107163918 | 13.0 | C |
| IDH-IV | 2x | - | 262.90638575793304 | 14.0 | C |
| IDH-VI | 2x | - | 10.736554504034476 | 16.0 | C |
| PYL13 | 2x | - | 49.41238715915894 | 10.0 | C |
| ABI1 | 2x | - | 71464.47437230883 | 18.0 | B |
| AT2G02400 | 2x | 2x | 2.906083107163918 | 13.0 | C |
| AT1G32710 | 2x | - | 4.849843437428617 | 20.0 | C |
| MUR1 | 2x | - | 4646.28928182305 | 22.0 | C |
| UGD3 | 2x | - | 15043.549012003572 | 29.0 | B |
| TIP1;3 | 2x | - | 0.0 | 3.0 | C |
| AT1G14810 | 2x | - | 6388.125010246513 | 40.0 | C |
| CYCD3;3 | 2x | - | 248.97777359957973 | 18.0 | C |
| AT5G40460 | 2x | - | 181.96410799625343 | 9.0 | C |
| AT4G32360 | 2x | - | 3263.6445526794146 | 68.0 | H |
| AT1G80230 | 2x | - | 8.07491466249984 | 27.0 | C |
| AT5G13440 | 2x | - | 15662.918105032259 | 34.0 | B |
| ATP5 | 2x | - | 279.3221295890634 | 25.0 | C |
| AT1G17890.1 | 2x | - | 2145.8801239156487 | 14.0 | C |
| GMD1 | 2x | - | 9.41223973026206 | 21.0 | C |
| GAI | 2x | - | 0.0 | 1.0 | C |
| AT3G15160 | 2x | - | 0.0 | 1.0 | C |
| PGR5 | 2x | - | 5.239391214214749 | 3.0 | C |
| WOx9 | 2x | - | 0.0 | 1.0 | C |
| RCD1 | 2x | - | 0.0 | 1.0 | C |
| AT3G48240 | 2x | - | 0.0 | 1.0 | C |
| AT5G63130 | 2x | - | 0.0 | 1.0 | C |
| AT5G17850 | 2x | - | 0.0 | 2.0 | C |
| HY2 | 2x | - | 0.0 | 1.0 | C |
| AT3G19400 | 2x | 2x | 0.0 | 1.0 | C |
| ZFP7 | 2x | - | 0.0 | 2.0 | C |
| AT2G46690 | 2x | - | 0.0 | 1.0 | C |
| AT2G18360 | 2x | - | 0.5539845566630971 | 11.0 | C |
| AT1G19680 | 2x | - | 0.0 | 1.0 | C |
| CAx9 | 2x | - | 0.0 | 2.0 | C |
| DWF1 | 2x | - | 10085.952349537178 | 15.0 | B |
| NTL9 | 2x | - | 36.046113418869005 | 6.0 | C |
| SUN1 | 2x | - | 3587.7557724461376 | 6.0 | C |
| AT5G19850 | 2x | - | 0.5539845566630971 | 11.0 | C |
| RGA1 | 2x | - | 0.0 | 1.0 | C |
| CAx11 | 2x | - | 0.0 | 2.0 | C |
| AT4G39050 | 2x | - | 173.48356924763064 | 3.0 | C |
| PGRL1B | 2x | - | 0.0 | 2.0 | C |
| AT5G17780 | 2x | - | 0.5539845566630971 | 11.0 | C |
| AT1G17430 | 2x | - | 0.5539845566630971 | 11.0 | C |
| CAx7 | 2x | - | 0.0 | 2.0 | C |
| AtkdsA1 | 2x | - | 12.714662042056503 | 5.0 | C |
| ACx2 | 2x | - | 2675.608367835126 | 16.0 | C |
| HYD1 | 2x | - | 2.479944575064897 | 8.0 | C |
| AT5G17720 | 2x | - | 0.5539845566630971 | 11.0 | C |
| ACx1 | 2x | - | 11700.085447504307 | 19.0 | B |
| CPI1 | 2x | - | 0.025477707006369428 | 4.0 | C |
| NHx1 | 2x | - | 3.541820877723112 | 12.0 | C |
| KAI2 | 2x | - | 0.5539845566630971 | 11.0 | C |
| AT3G43240 | 2x | 2x | 0.0 | 1.0 | C |
| GRIK1 | 2x | - | 1.9999999999999998 | 6.0 | C |
| AT4G15955 | 2x | - | 0.5539845566630971 | 11.0 | C |
| AT5G39790 | 2x | - | 71.10755210781993 | 5.0 | C |
| AT4G10030 | 2x | - | 0.5539845566630971 | 11.0 | C |
| TCP14 | 2x | - | 2717.264165616601 | 6.0 | C |
| AT1G72880 | 2x | - | 4.200205066110524 | 10.0 | C |
| AT2G28060 | 2x | - | 71.10755210781993 | 5.0 | C |
| AT4G15960 | 2x | - | 0.5539845566630971 | 11.0 | C |
| AT1G78210 | 2x | - | 0.5539845566630971 | 11.0 | C |
| CCx4 | 2x | - | 0.0 | 2.0 | C |
| AT4G12830 | 2x | - | 0.5539845566630971 | 11.0 | C |
| ACx6 | 2x | - | 2675.608367835126 | 16.0 | C |
| ACx3 | 2x | - | 8730.507091089863 | 17.0 | B |
| NAC089 | 2x | - | 111.94036654310256 | 5.0 | C |
| AT5G52450 | 2x | 2x | 0.6503856403126294 | 10.0 | C |
| AT3G03990 | 2x | - | 0.5539845566630971 | 11.0 | C |
| AT5G39220 | 2x | - | 0.5539845566630971 | 11.0 | C |
| NHx5 | 2x | - | 3.541820877723112 | 13.0 | C |
| AT5G22355 | 2x | - | 48103.912953571635 | 15.0 | B |
| AT1G52510 | 2x | - | 0.5539845566630971 | 11.0 | C |
| AT1G77460 | 2x | - | 65.20198990801754 | 33.0 | C |
| AT4G24140 | 2x | - | 0.5539845566630971 | 11.0 | C |
| SAHH2 | 2x | - | 164.22736882679214 | 47.0 | C |
| AT3G61540 | 2x | - | 0.5539845566630971 | 11.0 | C |
| AT1G72620 | 2x | - | 0.5539845566630971 | 11.0 | C |
| AT1G52750 | 2x | - | 0.5539845566630971 | 11.0 | C |
| NHx2 | 2x | - | 3.541820877723112 | 12.0 | C |
| AT1G27300 | 2x | - | 253.45776918303338 | 6.0 | C |
| EIF2 | 2x | - | 96.76853339557469 | 55.0 | H |
| NHx4 | 2x | - | 3.541820877723112 | 12.0 | C |
| AT5G24490 | 2x | - | 6340.626583495262 | 16.0 | C |
| AT4G33180 | 2x | - | 0.5539845566630971 | 11.0 | C |
| AT1G13820 | 2x | - | 0.5539845566630971 | 11.0 | C |
| AT4G36550 | 2x | - | 65.20198990801754 | 33.0 | C |
| PPH | 2x | - | 0.5539845566630971 | 11.0 | C |
| AT2G36260 | 2x | - | 34.218791862240145 | 15.0 | C |
| AT4G24160 | 2x | - | 0.5539845566630971 | 11.0 | C |
| TRx3 | 2x | - | 646.5796954898493 | 44.0 | C |
| AT3G06690 | 2x | - | 2675.608367835126 | 16.0 | C |
| LCBK2 | 2x | - | 69.47646857903916 | 53.0 | H |
| BRIZ2 | 2x | - | 3609.534453041015 | 51.0 | H |
| AT5G03905 | 2x | - | 232.70603525367918 | 16.0 | C |
| UGT84A2 | 2x | - | 33.08441341823758 | 7.0 | C |
| AT1G73920 | 2x | - | 1321.063674432007 | 47.0 | C |
| SOS1 | 2x | - | 5682.662286807672 | 14.0 | C |
| CYP702A5 | 2x | - | 1179.145169617838 | 98.0 | H |
| PTF1 | 2x | - | 6615.938229788126 | 9.0 | C |
| AT4G01920 | 2x | - | 8.819317908133698 | 3.0 | C |
| AT4G02340 | 2x | 2x | 0.5539845566630971 | 11.0 | C |
| AT2G40095 | 2x | - | 0.5539845566630971 | 11.0 | C |
| ATKDSA2 | 2x | - | 12.714662042056503 | 5.0 | C |
| CKS | 2x | - | 116.60990543249702 | 6.0 | C |
| AT4G14930 | 2x | - | 4.200205066110524 | 10.0 | C |
| AT5G21950 | 2x | - | 0.5539845566630971 | 11.0 | C |
| NHx3 | 2x | - | 3.541820877723112 | 12.0 | C |
| CYP77A5P | 2x | - | 787.6064545565157 | 52.0 | H |
| CYP96A9 | 2x | - | 1179.145169617838 | 98.0 | H |
| PUB8 | 2x | - | 65.20198990801754 | 33.0 | C |
| ATARCA | 2x | - | 1881.8145754723366 | 57.0 | H |
| BAS | 2x | - | 1722.9662280004463 | 143.0 | H |
| MFP2 | 2x | - | 45417.70987779679 | 68.0 | HB |
| ACx5 | 2x | - | 8730.507091089863 | 18.0 | B |
| AT5G67340 | 2x | - | 65.20198990801754 | 33.0 | C |
| PHYLLO | 2x | - | 1792.2308159618071 | 56.0 | H |
| LCBK1 | 2x | - | 69.47646857903916 | 53.0 | H |
| BR6Ox1 | 2x | - | 1179.145169617838 | 98.0 | H |
| DWF4 | 2x | - | 1447.4781552145996 | 102.0 | H |
| AT2G45720 | 2x | - | 65.20198990801754 | 33.0 | C |
| AT4G32590 | 2x | - | 2806.8836064261113 | 73.0 | H |
| AT4G13720 | 2x | - | 7281.99558582692 | 60.0 | H |
| CAx3 | 2x | - | 3004.0355340745978 | 41.0 | C |
| GRxS17 | 2x | - | 24370.478492150523 | 61.0 | HB |
| CYP51G1 | 2x | - | 3249.979978413782 | 103.0 | H |
| AT2G26750 | 2x | - | 0.5539845566630971 | 11.0 | C |
| AT1G15490 | 2x | - | 0.5539845566630971 | 11.0 | C |
| AT3G21670 | 2x | 2x | 65.20198990801754 | 32.0 | C |
| AT5G08250 | 2x | - | 1179.145169617838 | 98.0 | H |
| AT1G15810 | 2x | - | 648.7669362952698 | 70.0 | H |
| UPP | 2x | - | 1600.4088534747673 | 49.0 | C |
| AT1G18460 | 2x | - | 830.6052624192889 | 45.0 | C |
| SUE4 | 2x | - | 0.5539845566630971 | 11.0 | C |
| AT4G36530 | 2x | - | 0.5539845566630971 | 11.0 | C |
| GRIK2 | 2x | - | 1.9999999999999998 | 6.0 | C |
| At2g15230 | 2x | - | 830.6052624192889 | 45.0 | C |
| CAx6 | 2x | - | 3004.0355340745978 | 40.0 | C |
| AT5G09430 | 2x | - | 0.5539845566630971 | 11.0 | C |
| GGP3 | 2x | - | 72.56896215350157 | 56.0 | H |
| PUB43 | 2x | - | 65.20198990801754 | 33.0 | C |
| CYP96A12 | 2x | - | 1179.145169617838 | 98.0 | H |
| AT3G52570 | 2x | - | 0.5539845566630971 | 11.0 | C |
| THAS1 | 2x | - | 1722.9662280004463 | 143.0 | H |
| AT4G01790 | 2x | - | 515.7093253164239 | 66.0 | H |
| AT4G25290 | 2x | - | 0.5539845566630971 | 11.0 | C |
| BR6Ox2 | 2x | - | 1179.1649715980361 | 100.0 | H |
| CYP72A13 | 2x | 2x | 1179.145169617838 | 98.0 | H |
| AT1G01830 | 2x | - | 65.20198990801754 | 33.0 | C |
| AT4G36610 | 2x | - | 0.5539845566630971 | 11.0 | C |
| IBR3 | 2x | - | 0.3908915941282626 | 10.0 | C |
| SPHK2 | 2x | - | 17424.28409776902 | 58.0 | HB |
| ACD5 | 2x | - | 57.318624997615686 | 50.0 | C |
| CHx20 | 2x | 2x | 3157.1666153899696 | 43.0 | C |
| AT2G05810 | 2x | - | 65.20198990801754 | 33.0 | C |
| CYP707A4 | 2x | - | 1179.145169617838 | 98.0 | H |
| AT1G15165 | 2x | - | 65.20198990801754 | 33.0 | C |
| emb1997 | 2x | 2x | 236.5648038909372 | 69.0 | H |
| AT5G50900 | 2x | - | 65.20198990801754 | 33.0 | C |
| AT1G79470 | 2x | - | 3859.6315030804744 | 102.0 | H |
| AT2G23970 | 2x | 2x | 72.56896215350157 | 56.0 | H |
| CYP72A9 | 2x | - | 1179.145169617838 | 98.0 | H |
| AT5G07840 | 2x | - | 13515.953748626482 | 35.0 | B |
| AT5G36150.1 | 2x | - | 1722.9662280004463 | 138.0 | H |
| CPD | 2x | - | 1179.1649715980361 | 101.0 | H |
| PEN1 | 2x | - | 1722.9662280004463 | 143.0 | H |
| AT2G23960 | 2x | - | 72.56896215350157 | 56.0 | H |
| AT3G26590 | 2x | 2x | 0.6503856403126294 | 10.0 | C |
| ECHID | 2x | - | 1553.5737500193757 | 62.0 | H |
| AT4G12710 | 2x | - | 65.20198990801754 | 33.0 | C |
| At2g26260 | 2x | - | 1190.695987241267 | 146.0 | H |
| AT4G11160 | 2x | - | 1449.5191823176026 | 65.0 | H |
| AT4G36140 | 2x | - | 65.20198990801754 | 33.0 | C |
| GGP1 | 2x | - | 72.56896215350157 | 56.0 | H |
| ERG28 | 2x | - | 1127.765254733098 | 114.0 | H |
| CYP716A2 | 2x | - | 1179.145169617838 | 98.0 | H |
| CPISCA | 2x | - | 34.218791862240145 | 15.0 | C |
| CYP702A8 | 2x | - | 1179.145169617838 | 98.0 | H |
| CAx2 | 2x | - | 4052.806421560106 | 41.0 | C |
| LpxK | 2x | - | 27.63007021382563 | 7.0 | C |
| KEA2 | 2x | - | 5566.5816099948015 | 63.0 | H |
| ASB1 | 2x | - | 307.01300930376493 | 38.0 | C |
| CYP724A1 | 2x | - | 1179.145169617838 | 98.0 | H |
| AT5G23330 | 2x | - | 419.30561238834935 | 32.0 | C |
| ARIA | 2x | - | 65.20198990801754 | 33.0 | C |
| SPHK1 | 2x | - | 9981.992066658224 | 57.0 | HB |
| CYP90D1 | 2x | - | 1179.185169617838 | 101.0 | H |
| NHx8 | 2x | - | 3.541820877723112 | 12.0 | C |
| AT1G08315 | 2x | - | 65.20198990801754 | 33.0 | C |
| AT4G34730 | 2x | - | 137.8145551622624 | 31.0 | C |
| G-TMT | 2x | - | 1134.9063344658493 | 132.0 | H |
| FdC1 | 2x | - | 1975.4573673840594 | 72.0 | H |
| ASE2 | 2x | - | 13332.214046964404 | 66.0 | HB |
| CAx5 | 2x | - | 3004.0355340745978 | 40.0 | C |
| AT2G27430 | 2x | - | 65.20198990801754 | 33.0 | C |
| FAH2 | 2x | - | 502.3787369801068 | 102.0 | H |
| PUB12 | 2x | - | 594.2484974475183 | 34.0 | C |
| AT4G10050 | 2x | - | 0.5539845566630971 | 11.0 | C |
| AT4G19010 | 2x | - | 35072.34284754601 | 164.0 | HB |
| CYP704A2 | 2x | - | 1179.145169617838 | 98.0 | H |
| BARS1 | 2x | - | 1722.9662280004463 | 138.0 | H |
| AT1G80280 | 2x | - | 0.5539845566630971 | 11.0 | C |
| PUB15 | 2x | - | 65.20198990801754 | 33.0 | C |
| CYP86B1 | 2x | - | 1179.145169617838 | 98.0 | H |
| AT5G16340 | 2x | - | 35072.34284754601 | 164.0 | HB |
| CYP86A8 | 2x | - | 1179.145169617838 | 98.0 | H |
| AT5G63380 | 2x | - | 35072.34284754601 | 164.0 | HB |
| CYP704A1 | 2x | - | 1179.145169617838 | 98.0 | H |
| AT-HF | 2x | - | 434.1075234500636 | 73.0 | H |
| SQE3 | 2x | - | 1206.656593795035 | 146.0 | H |
| DWF5 | 2x | - | 7863.786026104707 | 142.0 | H |
| CYP72A7 | 2x | - | 1179.145169617838 | 98.0 | H |
| LAS1 | 2x | - | 1947.615410344972 | 140.0 | H |
| AT3G44970 | 2x | - | 1179.145169617838 | 98.0 | H |
| CYP72A10 | 2x | - | 1179.145169617838 | 98.0 | H |
| SQE6 | 2x | - | 1206.656593795035 | 146.0 | H |
| FUM2 | 2x | - | 77.27630879732364 | 54.0 | H |
| ROT3 | 2x | - | 1179.185169617838 | 101.0 | H |
| AT3G01660 | 2x | - | 1134.9063344658493 | 132.0 | H |
| SMO1-1 | 2x | - | 2395.2915652488114 | 158.0 | H |
| CYP96A13 | 2x | - | 1179.145169617838 | 98.0 | H |
| MPL1 | 2x | - | 830.6052624192889 | 45.0 | C |
| FMN/FHY | 2x | - | 929.5698837301181 | 66.0 | H |
| CAMS1 | 2x | - | 1722.9662280004463 | 143.0 | H |
| CYP702A6 | 2x | - | 1179.145169617838 | 98.0 | H |
| AT5G14510 | 2x | - | 65.20198990801754 | 33.0 | C |
| SQP2 | 2x | - | 1206.656593795035 | 146.0 | H |
| AT2G43420 | 2x | - | 1190.695987241267 | 146.0 | H |
| AT3G03440 | 2x | - | 65.20198990801754 | 33.0 | C |
| CAS1 | 2x | - | 1722.9662280004463 | 139.0 | H |
| PMEAMT | 2x | - | 1134.9063344658493 | 132.0 | H |
| AT2G33630 | 2x | - | 1190.695987241267 | 146.0 | H |
| CYP715A1 | 2x | - | 1179.145169617838 | 98.0 | H |
| CYP94B1 | 2x | - | 1179.145169617838 | 98.0 | H |
| xPL1 | 2x | - | 1134.9063344658493 | 132.0 | H |
| LUP1 | 2x | - | 1722.9662280004463 | 143.0 | H |
| BRIZ1 | 2x | - | 3609.534453041015 | 51.0 | H |
| AT1G68940 | 2x | - | 65.20198990801754 | 33.0 | C |
| AT1G78480 | 2x | - | 1722.9662280004463 | 138.0 | H |
| xF1 | 2x | - | 1206.656593795035 | 146.0 | H |
| FD3 | 2x | 4x | 1975.4573673840594 | 72.0 | H |
| CYP97B3 | 2x | - | 1179.145169617838 | 98.0 | H |
| CYP709B1 | 2x | - | 1179.145169617838 | 98.0 | H |
| MRN1 | 2x | - | 1722.9662280004463 | 138.0 | H |
| AT3G02590 | 2x | - | 2131.122478885803 | 164.0 | H |
| CYP714A2 | 2x | - | 1179.145169617838 | 98.0 | H |
| KEA1 | 2x | - | 5566.5816099948015 | 63.0 | H |
| AT4G39955 | 2x | - | 0.5539845566630971 | 11.0 | C |
| SQE2 | 2x | - | 1206.656593795035 | 146.0 | H |
| CYP72A8 | 2x | - | 1179.145169617838 | 98.0 | H |
| STE1 | 2x | - | 2131.122478885803 | 164.0 | H |
| AT1G44120 | 2x | - | 65.20198990801754 | 33.0 | C |
| AAE3 | 2x | - | 35442.45393132847 | 165.0 | HB |
| FD1 | 2x | - | 2864.238774722979 | 74.0 | H |
| AT1G78500 | 2x | - | 1722.9662280004463 | 138.0 | H |
| CAx1 | 2x | - | 3437.3272464805605 | 42.0 | C |
| CYP96A2 | 2x | - | 1179.145169617838 | 98.0 | H |
| AIM1 | 2x | - | 45417.70987779679 | 68.0 | HB |
| AT1G16350 | 2x | - | 3859.6315030804744 | 102.0 | H |
| AAE5 | 2x | - | 35442.45393132847 | 165.0 | HB |
| CYP708A2 | 2x | - | 1179.145169617838 | 98.0 | H |
| LUP5 | 2x | - | 1722.9662280004463 | 138.0 | H |
| CYP72A11 | 2x | - | 1179.145169617838 | 98.0 | H |
| AT3G29255 | 2x | - | 1722.9662280004463 | 138.0 | H |
| SMO2-2 | 2x | - | 2567.858313069768 | 157.0 | H |
| CYP702A3 | 2x | - | 1179.145169617838 | 98.0 | H |
| NHx6 | 2x | - | 736.1537314253222 | 14.0 | C |
| ACx4 | 2x | - | 2675.608367835126 | 16.0 | C |
| AT5G24155 | 2x | - | 1206.656593795035 | 146.0 | H |
| AT1G73600 | 2x | - | 1134.9063344658493 | 132.0 | H |
| CYP86C2 | 2x | - | 1179.145169617838 | 98.0 | H |
| CYP709B2 | 2x | - | 1179.145169617838 | 98.0 | H |
| AT4G30540 | 2x | - | 72.56896215350157 | 56.0 | H |
| CYP86A1 | 2x | - | 1179.145169617838 | 98.0 | H |
| FK | 2x | - | 2739.376821660361 | 138.0 | H |
| CYP96A10 | 2x | - | 1179.145169617838 | 98.0 | H |
| AT5G57890 | 2x | - | 307.01300930376493 | 38.0 | C |
| AT5G51900 | 2x | - | 1179.145169617838 | 98.0 | H |
| ASE3 | 2x | - | 213.77439761208515 | 64.0 | H |
| SMO2-1 | 2x | - | 1881.9662967804347 | 157.0 | H |
| CYP709B3 | 2x | - | 1179.145169617838 | 98.0 | H |
| CYP707A3 | 2x | - | 1179.145169617838 | 98.0 | H |
| ATFAH1 | 2x | - | 502.3787369801068 | 102.0 | H |
| SMT1 | 2x | 2x | 2026.067002858369 | 134.0 | H |
| SMT3 | 2x | - | 1817.3376139009777 | 133.0 | H |
| CYP72A14 | 2x | 2x | 1179.145169617838 | 98.0 | H |
| CYP94B3 | 2x | - | 1232.4194751800799 | 99.0 | H |
| SMO1-2 | 2x | - | 2395.2915652488114 | 158.0 | H |
| BDG1 | 2x | - | 0.5539845566630971 | 11.0 | C |
| MOD1 | 2x | - | 8226.079124104057 | 58.0 | H |
| T3F24.9 | 2x | - | 2511.7504451152954 | 147.0 | H |
| FD4 | 2x | - | 1975.4573673840594 | 72.0 | H |
| CYP716A1 | 2x | - | 1179.145169617838 | 98.0 | H |
| AT4G16490 | 2x | - | 65.20198990801754 | 33.0 | C |
| SETH3 | 2x | - | 59140.7629454874 | 110.0 | HB |
| SQP1 | 2x | - | 1206.656593795035 | 146.0 | H |
| CYP94B2 | 2x | - | 1179.145169617838 | 98.0 | H |
| rps15 | 2x | - | 648.7669362952698 | 70.0 | H |
| CYP86A2 | 2x | - | 1179.145169617838 | 98.0 | H |
| KEA3 | 2x | - | 5566.5816099948015 | 63.0 | H |
| CYP714A1 | 2x | - | 1179.145169617838 | 98.0 | H |
| 4CL8 | 2x | - | 35619.76932045945 | 165.0 | HB |
| CYP72A15 | 2x | - | 1179.145169617838 | 98.0 | H |
| FUG1 | 2x | - | 1449.5191823176026 | 65.0 | H |
| AT1G80620 | 2x | - | 648.7669362952698 | 70.0 | H |
| PUR2 | 2x | - | 407.28686906552423 | 73.0 | H |
| ASE1 | 2x | - | 213.77439761208515 | 64.0 | H |
| CAx4 | 2x | - | 3004.0355340745978 | 40.0 | C |
| AT1G61350 | 2x | - | 65.20198990801754 | 33.0 | C |
| CYP96A11 | 2x | - | 1179.145169617838 | 98.0 | H |
| CYP702A2 | 2x | - | 1179.145169617838 | 98.0 | H |
| PSY | 2x | - | 2654.1915149664837 | 152.0 | H |
| LUP2 | 2x | - | 1722.9662280004463 | 143.0 | H |
| SMT2 | 2x | - | 1817.3376139009777 | 133.0 | H |
| CYP707A1 | 2x | - | 1179.145169617838 | 98.0 | H |
| IAMT1 | 2x | - | 0.0 | 1.0 | C |
| AT1G15670 | 2x | - | 0.0 | 1.0 | C |
| AT5G57120 | 2x | - | 0.0 | 1.0 | C |
| AT3G59940 | 2x | - | 0.0 | 1.0 | C |
| AT3G28430 | 2x | 2x | 0.0 | 1.0 | C |
| ESC | 2x | - | 0.0 | 1.0 | C |
| AT1G34575 | 2x | - | 0.0 | 2.0 | C |
| AT2G38905 | 2x | 2x | 0.0 | 1.0 | C |
| AT3G23310 | 2x | - | 0.0 | 2.0 | C |
| AT5G09890 | 2x | - | 0.0 | 2.0 | C |
| AT2G19400 | 2x | - | 0.0 | 2.0 | C |
| AT2G44130 | 2x | - | 0.0 | 1.0 | C |
| CKx3 | 2x | - | 0.0 | 2.0 | C |
| AT1G26420 | 2x | - | 0.0 | 2.0 | C |
| AT1G11770 | 2x | - | 0.0 | 2.0 | C |
| AT1G26400 | 2x | - | 0.0 | 2.0 | C |
| AT1G26410 | 2x | - | 0.0 | 2.0 | C |
| AT4G20800 | 2x | - | 0.0 | 2.0 | C |
| AT2G17760 | 2x | 2x | 0.0 | 2.0 | C |
| AT1G03920 | 2x | - | 0.0 | 2.0 | C |
| CKx6 | 2x | - | 0.0 | 2.0 | C |
| CKx7 | 2x | - | 0.0 | 2.0 | C |
| GulLO2 | 2x | - | 0.0 | 2.0 | C |
| TT1 | 2x | - | 445.53742090600997 | 2.0 | C |
| AT1G30700 | 2x | - | 0.0 | 2.0 | C |
| AT5G06580 | 2x | - | 0.0 | 2.0 | C |
| AT5G44390 | 2x | - | 0.0 | 2.0 | C |
| AT5G44440 | 2x | - | 0.0 | 2.0 | C |
| AT3G49640 | 2x | - | 7.541088999515896 | 9.0 | C |
| GulLO4 | 2x | - | 0.0 | 2.0 | C |
| BIO1 | 2x | - | 0.0 | 1.0 | C |
| AT2G20470 | 2x | - | 0.0 | 2.0 | C |
| AT5G44410 | 2x | - | 0.0 | 2.0 | C |
| AT4G14350 | 2x | - | 0.0 | 2.0 | C |
| AT1G30640 | 2x | - | 0.0 | 2.0 | C |
| CKx5 | 2x | - | 0.0 | 2.0 | C |
| PEx4 | 2x | - | 1.0835306705285934 | 5.0 | C |
| WPP2 | 2x | - | 1.0 | 4.0 | C |
| ATSEC1A | 2x | - | 0.0 | 2.0 | C |
| GulLO5 | 2x | - | 0.0 | 2.0 | C |
| SUN2 | 2x | - | 0.0 | 3.0 | C |
| AT5G44380 | 2x | - | 0.0 | 2.0 | C |
| AT2G34810 | 2x | - | 0.0 | 2.0 | C |
| AT4G20840 | 2x | - | 0.0 | 2.0 | C |
| UBC37 | 2x | - | 1.0835306705285934 | 5.0 | C |
| AT3G13360.1 | 2x | - | 1568.3001069145143 | 4.0 | C |
| AT1G26390 | 2x | - | 0.0 | 2.0 | C |
| AT4G20830 | 2x | - | 0.0 | 2.0 | C |
| GulLO1 | 2x | - | 0.0 | 2.0 | C |
| GulLO3 | 2x | - | 0.0 | 2.0 | C |
| AT5G44360 | 2x | - | 0.0 | 2.0 | C |
| UBC35 | 2x | - | 1.0835306705285934 | 5.0 | C |
| MEE23 | 2x | 2x | 0.0 | 2.0 | C |
| AT4G26450 | 2x | - | 1.0 | 3.0 | C |
| WIP1 | 2x | - | 13275.457383848066 | 12.0 | B |
| AT1G30740 | 2x | - | 0.0 | 2.0 | C |
| AT1G30730 | 2x | - | 0.0 | 2.0 | C |
| CKx1 | 2x | - | 0.0 | 2.0 | C |
| CRF7 | 2x | - | 1.5770737449684822 | 13.0 | C |
| ETFQO | 2x | - | 0.0 | 2.0 | C |
| SOB3 | 2x | - | 16974.809481567067 | 5.0 | B |
| MEE60 | 2x | 2x | 0.0 | 1.0 | C |
| GulLO6 | 2x | - | 0.0 | 2.0 | C |
| AT1G30760 | 2x | - | 0.0 | 2.0 | C |
| AT3G49645 | 2x | - | 7.541088999515896 | 9.0 | C |
| AT5G44400 | 2x | - | 0.0 | 2.0 | C |
| AT4G20860 | 2x | - | 0.0 | 2.0 | C |
| CRF3 | 2x | - | 0.8373479152426522 | 12.0 | C |
| AT1G30710 | 2x | - | 0.0 | 2.0 | C |
| GulLO7 | 2x | - | 0.0 | 2.0 | C |
| CRF6 | 2x | - | 1.5770737449684822 | 13.0 | C |
| CRF4 | 2x | - | 1.5770737449684822 | 13.0 | C |
| WIT1 | 2x | - | 5881.799786843433 | 7.0 | C |
| PHYE | 2x | - | 119.86511692981968 | 9.0 | C |
| CRF5 | 2x | - | 1.5770737449684822 | 13.0 | C |
| ERS2 | 2x | - | 4.386362282517885 | 9.0 | C |
| HSP91 | 2x | - | 21325.244679597272 | 64.0 | HB |
| UBP12 | 2x | - | 1073.4372877507706 | 35.0 | C |
| CRF2 | 2x | - | 1.5770737449684822 | 13.0 | C |
| AT2G32790 | 2x | - | 1.0835306705285934 | 5.0 | C |
| PDK | 2x | - | 4.386362282517885 | 8.0 | C |
| RANGAP2 | 2x | - | 19801.2620041265 | 35.0 | B |
| ARP2 | 2x | - | 65.20198990801754 | 33.0 | C |
| UBC36 | 2x | - | 1.0835306705285934 | 5.0 | C |
| WPP1 | 2x | - | 1.0 | 4.0 | C |
| WIP2 | 2x | - | 4488.187976335858 | 8.0 | C |
| AT1G11660 | 2x | - | 21325.244679597272 | 64.0 | HB |
| AT4G20820 | 2x | - | 0.0 | 2.0 | C |
| CKx4 | 2x | - | 0.0 | 2.0 | C |
| ETFBETA | 2x | - | 101650.1758017344 | 59.0 | HB |
| CRF1 | 2x | - | 1.5770737449684822 | 13.0 | C |
| CKx2 | 2x | - | 0.0 | 2.0 | C |
| RANGAP1 | 2x | - | 26998.462103906393 | 36.0 | B |
| UBP26 | 2x | - | 1073.4372877507706 | 35.0 | C |
| AT1G26380 | 2x | - | 0.0 | 2.0 | C |
| AT1G30720 | 2x | - | 0.0 | 2.0 | C |
| ETFALPHA | 2x | - | 146071.04628014305 | 61.0 | HB |
| D2HGDH | 2x | - | 30226.20287817505 | 34.0 | B |
| UBC31 | 2x | - | 1.0835306705285934 | 5.0 | C |
| PHYC | 2x | - | 119.86511692981968 | 9.0 | C |
| AT5G03495 | 2x | - | 1073.4372877507706 | 35.0 | C |
| CRF8 | 2x | - | 1.5770737449684822 | 13.0 | C |
| AHP4 | 2x | - | 54.56069094304384 | 19.0 | C |
| AT4G04402 | 2x | - | 17.56349206349204 | 11.0 | C |
| AT2G07698 | 2x | - | 14471.815558496444 | 33.0 | B |
| AT1G44180 | 2x | - | 65.20198990801754 | 33.0 | C |
| RR24 | 2x | - | 4.386362282517885 | 8.0 | C |
| RR1 | 2x | - | 176841.0291804203 | 31.0 | B |
| AT5G03480 | 2x | - | 1073.4372877507706 | 35.0 | C |
| UBC27 | 2x | - | 8365.086281002812 | 48.0 | C |
| AQI | 2x | - | 65.20198990801754 | 33.0 | C |
| UBC28 | 2x | - | 1.0835306705285934 | 6.0 | C |
| AT1G44820 | 2x | - | 65.20198990801754 | 33.0 | C |
| UBP13 | 2x | - | 1073.4372877507706 | 35.0 | C |
| AT1G15130 | 2x | - | 65.20198990801754 | 33.0 | C |
| ATP1 | 2x | - | 14471.815558496444 | 34.0 | B |
| ABCG41 | 2x | - | 0.0 | 2.0 | C |
| ABCG35 | 2x | - | 0.0 | 2.0 | C |
| ABCG32 | 2x | - | 0.0 | 2.0 | C |
| ABCG33 | 2x | - | 0.0 | 2.0 | C |
| ABCG29 | 2x | - | 0.0 | 2.0 | C |
| TPR7 | 2x | - | 0.0 | 2.0 | C |
| AT4G27900 | 2x | 2x | 0.0 | 1.0 | C |
| ABCG36 | 2x | - | 0.0 | 2.0 | C |
| AT4G23460 | 2x | - | 0.5 | 4.0 | C |
| ABCG40 | 2x | 2x | 0.0 | 2.0 | C |
| LCB1 | 2x | - | 1106.023196381669 | 6.0 | C |
| AT5G11340 | 2x | - | 0.014925373134328358 | 5.0 | C |
| AT2G25830 | 2x | - | 0.0 | 2.0 | C |
| ZR3 | 2x | - | 1.2673992673992673 | 5.0 | C |
| AT1G01350 | 2x | - | 0.11053940822204764 | 10.0 | C |
| SMC6A | 2x | - | 0.5201976690750535 | 8.0 | C |
| SBH2 | 2x | - | 13.799978403136215 | 5.0 | C |
| ABCG37 | 2x | - | 0.0 | 2.0 | C |
| ABCG38 | 2x | - | 0.0 | 2.0 | C |
| AT3G58490 | 2x | - | 3832.4079472903377 | 6.0 | C |
| AT3G45180 | 2x | - | 5.6152757117861976 | 14.0 | C |
| AT1G56290 | 2x | - | 0.014925373134328358 | 7.0 | C |
| cpHsc70-2 | 2x | - | 1340.2668060781393 | 21.0 | C |
| ABCG30 | 2x | - | 0.0 | 2.0 | C |
| TIM44-2 | 2x | - | 459.5981396786786 | 21.0 | C |
| AT3G53680 | 2x | 2x | 0.0 | 1.0 | C |
| ABCG42 | 2x | - | 0.0 | 2.0 | C |
| ABCG39 | 2x | 2x | 0.0 | 2.0 | C |
| ABCG31 | 2x | - | 75841.20464704557 | 17.0 | B |
| SBH1 | 2x | - | 13.799978403136215 | 5.0 | C |
| TIM17-1 | 2x | - | 482.6071044563792 | 14.0 | C |
| LSM8 | 2x | - | 15.206944354896134 | 40.0 | C |
| CDT1A | 2x | - | 21.285902582865784 | 3.0 | C |
| CDT1B | 2x | - | 21.285902582865784 | 3.0 | C |
| AT4G32208 | 2x | - | 1340.2668060781393 | 21.0 | C |
| BIP1 | 2x | - | 3086.082890919864 | 21.0 | C |
| BIP3 | 2x | - | 14.34708555515764 | 19.0 | C |
| PRP39 | 2x | - | 444.8204603700707 | 55.0 | H |
| MIM | 2x | - | 0.5201976690750535 | 8.0 | C |
| AT3G47120 | 2x | - | 243.95115149712888 | 24.0 | C |
| EMB2765 | 2x | - | 23.405138062094093 | 11.0 | C |
| UBL5 | 2x | - | 5.6152757117861976 | 14.0 | C |
| AT3G05070 | 2x | - | 47.32632957993743 | 15.0 | C |
| AT5G17440 | 2x | - | 9.854041980339602 | 36.0 | C |
| AT3G09700 | 2x | - | 824.481317186884 | 31.0 | C |
| LOH2 | 2x | 2x | 0.5 | 2.0 | C |
| ATCES1 | 2x | - | 1822.1920311126248 | 4.0 | C |
| ABCG15 | 2x | 2x | 207.65330704425963 | 15.0 | C |
| ABCG43 | 2x | - | 0.0 | 2.0 | C |
| ATERDJ3A | 2x | - | 363.41200832561776 | 23.0 | C |
| AT1G80930 | 2x | - | 83.45407480258329 | 50.0 | C |
| MOS4 | 2x | - | 1561.350355495199 | 29.0 | C |
| AT2G17880 | 2x | - | 4839.0614007154845 | 27.0 | C |
| emb1644 | 2x | - | 100.58409545494204 | 48.0 | C |
| SMP2 | 2x | - | 18.600945278171825 | 29.0 | C |
| J3 | 2x | - | 4839.0614007154845 | 27.0 | C |
| HSP70T-2 | 2x | - | 121.9503039510255 | 23.0 | C |
| AT5G06420 | 2x | - | 0.11053940822204764 | 10.0 | C |
| TIM44-1 | 2x | - | 459.5981396786786 | 21.0 | C |
| LSM6B | 2x | - | 22.820506507221452 | 57.0 | H |
| cpHsc70-1 | 2x | - | 1745.6226684610072 | 22.0 | C |
| AT4G33060 | 2x | - | 0.8386244975725008 | 11.0 | C |
| AT4G01023 | 2x | - | 0.11053940822204764 | 10.0 | C |
| AT2G14285.1 | 2x | - | 177.25659929519767 | 81.0 | H |
| AT3G02710 | 2x | - | 0.06060606060606061 | 6.0 | C |
| EMB2769 | 2x | - | 487.84626251079084 | 35.0 | C |
| AT2G29430 | 2x | - | 10.214886500481898 | 31.0 | C |
| MED6 | 2x | - | 0.0 | 2.0 | C |
| AT2G21510 | 2x | 2x | 3526.9464102793413 | 26.0 | C |
| AT2G35795 | 2x | - | 824.481317186884 | 31.0 | C |
| AT1G66510 | 2x | - | 22.163712331479495 | 36.0 | C |
| MAC5A | 2x | - | 1731.6831836000904 | 52.0 | H |
| LSM3A | 2x | - | 6.543256197421319 | 44.0 | C |
| LSM6A | 2x | - | 23.404437718833357 | 59.0 | H |
| DOT2 | 2x | - | 1057.4556136263109 | 102.0 | H |
| MAC5B | 2x | - | 1721.1681579947942 | 51.0 | H |
| MAC5C | 2x | - | 1721.1681579947942 | 51.0 | H |
| AT5G50970 | 2x | - | 415.2432748692975 | 24.0 | C |
| ATERDJ3B | 2x | - | 363.41200832561776 | 23.0 | C |
| J2 | 2x | - | 4839.0614007154845 | 27.0 | C |
| sks4 | 2x | 2x | 0.0 | 1.0 | C |
| AT5G46630 | 2x | - | 10522.474677300135 | 11.0 | C |
| EMB2816 | 2x | - | 1924.3598325288726 | 45.0 | C |
| AT1G17130 | 2x | - | 10.214886500481898 | 31.0 | C |
| AT2G42330 | 2x | - | 46.04055956490673 | 46.0 | C |
| AT5G26610 | 2x | - | 46.04055956490673 | 46.0 | C |
| AT5G51410 | 2x | - | 9.854041980339602 | 36.0 | C |
| AT4G16200 | 2x | - | 78.71920949523543 | 58.0 | H |
| MTHSC70-2 | 2x | - | 1544.0519558735655 | 26.0 | C |
| TIM17-3 | 2x | - | 482.6071044563792 | 14.0 | C |
| AT1G72070 | 2x | - | 4839.0614007154845 | 27.0 | C |
| STIPL1 | 2x | - | 46.04055956490673 | 46.0 | C |
| AT5G58790 | 2x | - | 243.95115149712888 | 24.0 | C |
| mtHsc70-1 | 2x | - | 1544.0519558735655 | 26.0 | C |
| AT2G43370 | 2x | - | 759.5631963269497 | 51.0 | H |
| AT5G12280 | 2x | - | 78.71920949523543 | 58.0 | H |
| TIM17-2 | 2x | - | 482.6071044563792 | 14.0 | C |
| AR192 | 2x | - | 852.2891695698 | 37.0 | C |
| AT1G26370 | 2x | - | 540.725263556452 | 46.0 | C |
| At1g65660 | 2x | - | 18.600945278171825 | 29.0 | C |
| ESP3 | 2x | - | 815.1069627960359 | 77.0 | H |
| BGAL14 | 2x | - | 3557.2299212051726 | 79.0 | H |
| AT1G36390 | 2x | - | 382.0930466020905 | 32.0 | C |
| J20 | 2x | - | 4839.0614007154845 | 27.0 | C |
| AT3G43250 | 2x | - | 10.214886500481898 | 31.0 | C |
| AT5G19920 | 2x | - | 415.2432748692975 | 24.0 | C |
| AT2G44200 | 2x | - | 27.227291136395515 | 26.0 | C |
| AT1G28060 | 2x | - | 140.94658980802046 | 62.0 | H |
| BIP2 | 2x | - | 2687.7885861857103 | 20.0 | C |
| AT1G31870 | 2x | - | 858.4774877918263 | 30.0 | C |
| AT5G41770 | 2x | - | 5338.4741007750135 | 103.0 | H |
| CDC5 | 2x | - | 5784.0474658793555 | 120.0 | H |
| AT5G04210 | 2x | - | 1721.1681579947942 | 51.0 | H |
| AT1G11520 | 2x | - | 325.53929280103506 | 84.0 | H |
| AT3G56790 | 2x | - | 140.94658980802046 | 62.0 | H |
| AT1G60200 | 2x | - | 43.35013379833525 | 48.0 | C |
| EMB1241 | 2x | - | 382.0930466020905 | 32.0 | C |
| AT5G03030 | 2x | - | 824.481317186884 | 31.0 | C |
| AT4G11380 | 2x | - | 0.5 | 4.0 | C |
| AT2G32050 | 2x | - | 10.214886500481898 | 31.0 | C |
| AT3G51110 | 2x | - | 5315.157362473084 | 102.0 | H |
| AT1G52325 | 2x | - | 83.45407480258329 | 50.0 | C |
| AT4G18465 | 2x | - | 540.725263556452 | 46.0 | C |
| AT4G08580 | 2x | - | 124.75709611301774 | 63.0 | H |
| AT2G16860 | 2x | - | 592.628992364649 | 48.0 | C |
| SAD1 | 2x | - | 20.782995464156457 | 53.0 | H |
| AT3G13210 | 2x | - | 5675.1503421124125 | 103.0 | H |
| LSM3B | 2x | - | 6.543256197421319 | 44.0 | C |
| AT3G49601 | 2x | - | 1622.3886150795097 | 70.0 | H |
| AT3G18790 | 2x | - | 449.30913467226907 | 59.0 | H |
| AT3G27600 | 2x | - | 78.71920949523543 | 58.0 | H |
| MGE1 | 2x | - | 852.2891695698 | 37.0 | C |
| emb1220 | 2x | - | 145.4873374347714 | 65.0 | H |
| AT5G17900 | 2x | - | 124.75709611301774 | 63.0 | H |
| AT3G09850 | 2x | 2x | 46.04055956490673 | 46.0 | C |
| AT2G47640.1 | 2x | - | 294.69917801172284 | 83.0 | H |
| UNE6 | 2x | - | 9.854041980339602 | 36.0 | C |
| AT4G16680 | 2x | - | 800.0154204559144 | 76.0 | H |
| AT5G45990 | 2x | - | 5315.157362473084 | 102.0 | H |
| MEE29 | 2x | - | 800.0154204559144 | 76.0 | H |
| AT1G70400 | 2x | - | 145.4873374347714 | 65.0 | H |
| AT5G06520 | 2x | - | 78.71920949523543 | 58.0 | H |
| AT3G55930 | 2x | - | 140.94658980802046 | 62.0 | H |
| AT3G49130 | 2x | - | 78.71920949523543 | 58.0 | H |
| SKIP | 2x | - | 4216.1417011340345 | 88.0 | H |
| AT5G06890 | 2x | - | 78.71920949523543 | 58.0 | H |
| AT1G27900 | 2x | - | 540.725263556452 | 46.0 | C |
| AT3G45950 | 2x | - | 18.600945278171825 | 29.0 | C |
