# Supplementary material for: Different adaptation strategies of two citrus scion/rootstock combinations in response to drought stress
Source: PLoS One. 2017 May 17;12(5):e0177993. doi: 10.1371/journal.pone.0177993 (PMC5435350; doi:10.1371/journal.pone.0177993)
Supplement: S3 Table — (DOCX) [file pone.0177993.s003.docx]

**S3 Table.** List of the proteins present in the clusters showed in the Figure 2.

| ID | Position in Figura 2 |
| --- | --- |
| AT3G22660 | Figura 2.G |
| NRPB12 | Figura 2.G |
| AT2G27720 | Figura 2.G |
| PDE318 | Figura 2.G |
| RPC14 | Figura 2.G |
| AT1G50920 | Figura 2.G |
| AT4G33865.1 | Figura 2.G |
| AT3G53870 | Figura 2.G |
| AT2G20450 | Figura 2.G |
| AT4G25730 | Figura 2.G |
| AT2G05220 | Figura 2.G |
| AT2G41840 | Figura 2.G |
| SAC52 | Figura 2.G |
| AT5G02960 | Figura 2.G |
| eIF6B | Figura 2.G |
| AT2G40590 | Figura 2.G |
| AT1G10300 | Figura 2.G |
| AT3G49910 | Figura 2.G |
| AT2G40010 | Figura 2.G |
| AT1G74060 | Figura 2.G |
| ATCG00380.1 | Figura 2.G |
| AT5G63070 | Figura 2.G |
| AT3G09500 | Figura 2.G |
| eIFiso4G1 | Figura 2.G |
| EMB2296 | Figura 2.G |
| AT5G04800 | Figura 2.G |
| RS27A | Figura 2.G |
| AT5G09510 | Figura 2.G |
| AT3G10610 | Figura 2.G |
| AT3G47370 | Figura 2.G |
| AT5G35680 | Figura 2.G |
| AT2G34480 | Figura 2.G |
| AT2G37990 | Figura 2.G |
| AT5G39850 | Figura 2.G |
| AT5G52650 | Figura 2.G |
| AT3G23860 | Figura 2.G |
| AT5G27700 | Figura 2.G |
| AT4G34555 | Figura 2.G |
| AT3G43980 | Figura 2.G |
| ECT3 | Figura 2.G |
| AT4G39200 | Figura 2.G |
| AT3G52580 | Figura 2.G |
| AT3G11510 | Figura 2.G |
| AT5G13830 | Figura 2.G |
| AT3G43980.1 | Figura 2.G |
| RPS5A | Figura 2.G |
| AT5G01230 | Figura 2.G |
| AT1G12960 | Figura 2.G |
| AT1G07930 | Figura 2.G |
| AT2G21580 | Figura 2.G |
| XW6 | Figura 2.G |
| AT3G53890 | Figura 2.G |
| ROC2 | Figura 2.G |
| AT3G18740 | Figura 2.G |
| AT1G29250 | Figura 2.G |
| BBC1 | Figura 2.G |
| AT5G59240 | Figura 2.G |
| AT3G25520 | Figura 2.G |
| RPS13A | Figura 2.G |
| AT1G33850 | Figura 2.G |
| AT5G18380 | Figura 2.G |
| AT2G42710 | Figura 2.G |
| RPL5B | Figura 2.G |
| AT1G67430 | Figura 2.G |
| PRH75 | Figura 2.G |
| AT3G53430 | Figura 2.G |
| AT3G12915 | Figura 2.G |
| RPL18 | Figura 2.G |
| AT5G61170 | Figura 2.G |
| AT5G56710 | Figura 2.G |
| RPS5B | Figura 2.G |
| TIM10 | Figura 2.G |
| AT2G32060 | Figura 2.G |
| AT5G61310 | Figura 2.G |
| AT3G14600 | Figura 2.G |
| AT5G02610 | Figura 2.G |
| RPL18AA | Figura 2.G |
| AT4G25740 | Figura 2.G |
| AT3G10090 | Figura 2.G |
| AT3G51190 | Figura 2.G |
| TIM8 | Figura 2.G |
| AT2G45710 | Figura 2.G |
| AT3G44590 | Figura 2.G |
| AT5G35530 | Figura 2.G |
| AT3G57490 | Figura 2.G |
| EMB1080 | Figura 2.G |
| AT1G18070 | Figura 2.G |
| RPL23AB | Figura 2.G |
| AT3G07230 | Figura 2.G |
| AT4G13170 | Figura 2.G |
| AT4G29390.1 | Figura 2.G |
| AT5G27850 | Figura 2.G |
| TOM5 | Figura 2.G |
| AtG2 | Figura 2.G |
| HDT4 | Figura 2.G |
| AT2G25210.1 | Figura 2.G |
| AT1G74050 | Figura 2.G |
| TIM13 | Figura 2.G |
| AT4G34670 | Figura 2.G |
| AT3G09630 | Figura 2.G |
| AT4G25890 | Figura 2.G |
| HLL | Figura 2.G |
| NOP10 | Figura 2.G |
| AT3G61111 | Figura 2.G |
| AT2G41650 | Figura 2.G |
| AT3G06320 | Figura 2.G |
| AT3G07110 | Figura 2.G |
| SAG24 | Figura 2.G |
| AT2G37190 | Figura 2.G |
| AT3G60245 | Figura 2.G |
| TOM6 | Figura 2.G |
| AT2G40510 | Figura 2.G |
| emb2386 | Figura 2.G |
| AT5G67510 | Figura 2.G |
| AT2G45860 | Figura 2.G |
| AT1G36240 | Figura 2.G |
| AT3G25940 | Figura 2.G |
| RPS15AD | Figura 2.G |
| AT1G15930 | Figura 2.G |
| AT4G27090 | Figura 2.G |
| RPL10B | Figura 2.G |
| EIF3C | Figura 2.G |
| AT3G45030 | Figura 2.G |
| AT3G06320.1 | Figura 2.G |
| AT1G70600 | Figura 2.G |
| AT5G07090 | Figura 2.G |
| AT2G19750 | Figura 2.G |
| AT1G08580 | Figura 2.G |
| AT5G28060 | Figura 2.G |
| RPL23.1 | Figura 2.G |
| RPL23AA | Figura 2.G |
| NFD3 | Figura 2.G |
| AT5G62300.1 | Figura 2.G |
| AT3G56340 | Figura 2.G |
| AT1G77940 | Figura 2.G |
| AT3G04920 | Figura 2.G |
| F19P19.29 | Figura 2.G |
| AT1G80470 | Figura 2.G |
| AT2G36160 | Figura 2.G |
| AT2G09990 | Figura 2.G |
| AT5G16130 | Figura 2.G |
| AT5G23900 | Figura 2.G |
| AT4G30800 | Figura 2.G |
| RPS11-BETA | Figura 2.G |
| RPS18C | Figura 2.G |
| AT4G26230 | Figura 2.G |
| P40 | Figura 2.G |
| AT5G20290 | Figura 2.G |
| CPSRP54 | Figura 2.G |
| AT1G18540 | Figura 2.G |
| AT5G43640 | Figura 2.G |
| AT2G04390 | Figura 2.G |
| AT3G48960 | Figura 2.G |
| AT1G34030 | Figura 2.G |
| AT5G58420 | Figura 2.G |
| AT2G04520 | Figura 2.G |
| PFL | Figura 2.G |
| emb2171 | Figura 2.G |
| AT5G46160 | Figura 2.G |
| AT5G48760 | Figura 2.G |
| RPL27AB | Figura 2.G |
| RPS6A | Figura 2.G |
| AT4G02230 | Figura 2.G |
| AT3G10950 | Figura 2.G |
| AT3G04840 | Figura 2.G |
| AT3G09680 | Figura 2.G |
| AT5G15520 | Figura 2.G |
| AT3G55170 | Figura 2.G |
| AT3G02560 | Figura 2.G |
| AT2G39390 | Figura 2.G |
| AT2G17360 | Figura 2.G |
| AT2G19740 | Figura 2.G |
| PRPL28 | Figura 2.G |
| AT3G60770 | Figura 2.G |
| At2g34520 | Figura 2.G |
| RPS11 | Figura 2.G |
| RPS28 | Figura 2.G |
| RPS10B | Figura 2.G |
| AT1G27400 | Figura 2.G |
| AT5G09500 | Figura 2.G |
| AT5G47930 | Figura 2.G |
| AT5G09490 | Figura 2.G |
| AT1G04480 | Figura 2.G |
| AT4G16720 | Figura 2.G |
| AT5G02870 | Figura 2.G |
| AT1G48830 | Figura 2.G |
| RPL3B | Figura 2.G |
| EMB3010 | Figura 2.G |
| AT3G02080 | Figura 2.G |
| AT5G60670 | Figura 2.G |
| AT2G31610 | Figura 2.G |
| AT2G39590 | Figura 2.G |
| AT4G17390 | Figura 2.G |
| AT5G15200 | Figura 2.G |
| LOS1 | Figura 2.G |
| RPL16A | Figura 2.G |
| AT1G29965 | Figura 2.G |
| rps14 | Figura 2.G |
| At1g43170 | Figura 2.G |
| RPL22 | Figura 2.G |
| AT3G04230 | Figura 2.G |
| AT4G28360 | Figura 2.G |
| AT5G47700 | Figura 2.G |
| AT4G15000 | Figura 2.G |
| RPL24A | Figura 2.G |
| AT4G00810 | Figura 2.G |
| AT1G07070 | Figura 2.G |
| AT4G36130 | Figura 2.G |
| AT3G22230 | Figura 2.G |
| AT3G48570 | Figura 2.G |
| AT2G36170 | Figura 2.G |
| AT3G28900 | Figura 2.G |
| AT1G02830 | Figura 2.G |
| RPL16B | Figura 2.G |
| AT5G02450 | Figura 2.G |
| EMB3137 | Figura 2.G |
| AT1G74270 | Figura 2.G |
| RPL34 | Figura 2.G |
| AT3G06700 | Figura 2.G |
| AT5G27770 | Figura 2.G |
| AT3G24830 | Figura 2.G |
| AT3G05560 | Figura 2.G |
| AT1G15250 | Figura 2.G |
| AT1G41880 | Figura 2.G |
| AT1G04480.1 | Figura 2.G |
| AT1G26880 | Figura 2.G |
| AT4G18100 | Figura 2.G |
| RPL14 | Figura 2.G |
| AT5G65220 | Figura 2.G |
| SEC61G2 | Figura 2.G |
| AT5G24510 | Figura 2.G |
| AT3G16780 | Figura 2.G |
| AT2G19730 | Figura 2.G |
| AT1G77750 | Figura 2.G |
| AT3G16080 | Figura 2.G |
| AT5G45775.2 | Figura 2.G |
| AT2G32220 | Figura 2.G |
| PGY1 | Figura 2.G |
| AT3G23390 | Figura 2.G |
| AT3G02190 | Figura 2.G |
| AT1G01100 | Figura 2.G |
| AT4G29410 | Figura 2.G |
| AT5G22440 | Figura 2.G |
| AT1G57660 | Figura 2.G |
| AT3G06680 | Figura 2.G |
| STV1 | Figura 2.G |
| AT3G58700.1 | Figura 2.G |
| AT2G25210 | Figura 2.G |
| AT5G46430 | Figura 2.G |
| AT2G37600 | Figura 2.G |
| AT3G53740 | Figura 2.G |
| AT3G55750 | Figura 2.G |
| AT1G52300 | Figura 2.G |
| AT1G08360 | Figura 2.G |
| UPF3 | Figura 2.G |
| UBC17 | Figura 2.G |
| APC11 | Figura 2.G |
| FER4 | Figura 2.G |
| AT4G16030 | Figura 2.G |
| AT2G40780 | Figura 2.G |
| FER1 | Figura 2.G |
| AT1G10585 | Figura 2.G |
| AT5G03830 | Figura 2.G |
| AAK6 | Figura 2.G |
| AT2G44510 | Figura 2.G |
| FER2 | Figura 2.G |
| FER3 | Figura 2.G |
| PEX1 | Figura 2.G |
| PEX6 | Figura 2.G |
| HEM15 | Figura 2.G |
| AT1G52370 | Figura 2.G |
| At2g30390 | Figura 2.G |
| AT5G26640 | Figura 2.G |
| ORA47 | Figura 2.G |
| NAM | Figura 2.G |
| ABCE2 | Figura 2.G |
| AT5G40590 | Figura 2.G |
| ABCE1 | Figura 2.G |
| LBA1 | Figura 2.G |
| AT1G62410 | Figura 2.G |
| ERF1-2 | Figura 2.G |
| ABCE3 | Figura 2.G |
| Y14 | Figura 2.G |
| EIF4A-III | Figura 2.G |
| PAB8 | Figura 2.G |
| AT2G43460 | Figura 2.G |
| PAB5 | Figura 2.G |
| PAB3 | Figura 2.G |
| ERF1-3 | Figura 2.G |
| AT2G39260 | Figura 2.G |
| ERF1-1 | Figura 2.G |
| AT3G59540.1 | Figura 2.G |
| AT4G14320 | Figura 2.G |
| AT1G77932 | Figura 2.G |
| At1g43 | Figura 2.G |
| PGY2 | Figura 2.G |
| AT1G33120 | Figura 2.G |
| AT1G48900 | Figura 2.G |
| ATHSRP54A | Figura 2.G |
| AT2G47570 | Figura 2.G |
| AT4G10450 | Figura 2.G |
| AT2G44120 | Figura 2.G |
| AT5G49500 | Figura 2.G |
| AT3G13580 | Figura 2.G |
| UBQ1 | Figura 2.G |
| AT3G61010 | Figura 2.G |
| eIF6A | Figura 2.G |
| AT1G79150 | Figura 2.G |
| AT1G77030 | Figura 2.G |
| AT5G06360 | Figura 2.G |
| NRPB6A | Figura 2.B |
| FD2 | Figura 2.B |
| PFK6 | Figura 2.B |
| ADH1 | Figura 2.B |
| PDC3 | Figura 2.B |
| PFK4 | Figura 2.B |
| FBP | Figura 2.B |
| AT3G45940 | Figura 2.B |
| LST8-2 | Figura 2.B |
| HPA1 | Figura 2.B |
| AT1G12000 | Figura 2.B |
| PDC2 | Figura 2.B |
| AT5G11720 | Figura 2.B |
| NIT1 | Figura 2.B |
| HA5 | Figura 2.B |
| AT4G33070 | Figura 2.B |
| F2KP | Figura 2.B |
| AT4G28410 | Figura 2.B |
| AT4G28420 | Figura 2.B |
| DIC2 | Figura 2.B |
| AT2G45290 | Figura 2.B |
| CHLM | Figura 2.B |
| AT1G32470 | Figura 2.B |
| AT3G29010 | Figura 2.B |
| NIT2 | Figura 2.B |
| YUC11 | Figura 2.B |
| NIT3 | Figura 2.B |
| AT1G12230 | Figura 2.B |
| AT5G01320 | Figura 2.B |
| PFK3 | Figura 2.B |
| AT3G61113 | Figura 2.B |
| DIN9 | Figura 2.B |
| PFK5 | Figura 2.B |
| HKL3 | Figura 2.B |
| MSD1 | Figura 2.B |
| MEE51 | Figura 2.B |
| AT5G24760 | Figura 2.B |
| MDHAR | Figura 2.B |
| MEE31 | Figura 2.B |
| AT3G56350 | Figura 2.B |
| TAT3 | Figura 2.B |
| FSD3 | Figura 2.B |
| AT1G06020 | Figura 2.B |
| SKD1 | Figura 2.B |
| HKL1 | Figura 2.B |
| ALDH2B7 | Figura 2.B |
| YUC10 | Figura 2.B |
| AT2G45695 | Figura 2.B |
| AT1G06030 | Figura 2.B |
| PFK7 | Figura 2.B |
| HXK1 | Figura 2.B |
| ALDH2B4 | Figura 2.B |
| AT5G51830 | Figura 2.B |
| AT4G10260 | Figura 2.B |
| AT2G46560 | Figura 2.B |
| AT4G00651 | Figura 2.B |
| MEB2 | Figura 2.B |
| FBA7 | Figura 2.B |
| AT1G76550 | Figura 2.B |
| AT2G31390 | Figura 2.B |
| FSD1 | Figura 2.B |
| FBA4 | Figura 2.B |
| PDE345 | Figura 2.B |
| VAB2 | Figura 2.B |
| AT3G58730 | Figura 2.B |
| FBA5 | Figura 2.B |
| FBA2 | Figura 2.B |
| FBA8 | Figura 2.B |
| VHA-A2 | Figura 2.B |
| MDAR1 | Figura 2.B |
| AT3G59480 | Figura 2.B |
| VHA-A1 | Figura 2.B |
| AT3G32040 | Figura 2.B |
| ABC4 | Figura 2.B |
| GOX2 | Figura 2.B |
| RPS7.2 | Figura 2.B |
| AT2G18620 | Figura 2.B |
| cPT5 | Figura 2.B |
| AT3G28715 | Figura 2.B |
| G4 | Figura 2.B |
| HAOX1 | Figura 2.B |
| RPS7 | Figura 2.B |
| AT3G14510 | Figura 2.B |
| HAOX2 | Figura 2.B |
| SPS2 | Figura 2.B |
| VATG3 | Figura 2.B |
| FPS1 | Figura 2.B |
| MVD1 | Figura 2.B |
| GOX3 | Figura 2.B |
| AT2G26500 | Figura 2.B |
| GGPS3 | Figura 2.B |
| EMB3126 | Figura 2.B |
| PB | Figura 2.B |
| IPT6 | Figura 2.B |
| At2g24210 | Figura 2.B |
| VHA-E2 | Figura 2.B |
| GGPS4 | Figura 2.B |
| cPT9 | Figura 2.B |
| GGPS2 | Figura 2.B |
| AT2G23400 | Figura 2.B |
| IPT9 | Figura 2.B |
| VAG2 | Figura 2.B |
| FPS2 | Figura 2.B |
| RPL33 | Figura 2.B |
| IPT5 | Figura 2.B |
| IPT3 | Figura 2.B |
| GPS1 | Figura 2.B |
| AT2G25610 | Figura 2.B |
| GGPS6 | Figura 2.B |
| PPa4 | Figura 2.B |
| GGR | Figura 2.B |
| PPT1 | Figura 2.B |
| AT5G58784 | Figura 2.B |
| AT2G42220 | Figura 2.B |
| AT4G01310 | Figura 2.B |
| cPT8 | Figura 2.B |
| AT3G54250 | Figura 2.B |
| PSAB | Figura 2.B |
| IPP1 | Figura 2.B |
| RPS7.1 | Figura 2.B |
| AT3G29430 | Figura 2.B |
| AVA-P1 | Figura 2.B |
| IPT2 | Figura 2.B |
| AT3G25810 | Figura 2.B |
| cPT6 | Figura 2.B |
| VMA10 | Figura 2.B |
| IPT8 | Figura 2.B |
| ATPH | Figura 2.B |
| IPT1 | Figura 2.B |
| HPT2 | Figura 2.B |
| HPT1 | Figura 2.B |
| YCF3 | Figura 2.B |
| AT3G20160 | Figura 2.B |
| LHCB2.1 | Figura 2.B |
| CPT | Figura 2.B |
| GGPS1 | Figura 2.B |
| AT3G42050 | Figura 2.B |
| AT3G14530 | Figura 2.B |
| AT5G08690 | Figura 2.B |
| TPS23 | Figura 2.B |
| ABCF1 | Figura 2.B |
| IPP2 | Figura 2.B |
| AT4G32530 | Figura 2.B |
| SPS1 | Figura 2.B |
| IPT4 | Figura 2.B |
| GOX1 | Figura 2.B |
| HDR | Figura 2.B |
| AVA-P2 | Figura 2.B |
| cPT4 | Figura 2.B |
| Pnsl5 | Figura 2.B |
| TIFY4A | Figura 2.B |
| AT1G56050 | Figura 2.B |
| cPT1 | Figura 2.B |
| TPS14 | Figura 2.B |
| AT2G16510.1 | Figura 2.B |
| IPT7 | Figura 2.B |
| ROC4 | Figura 2.B |
| PSAC | Figura 2.B |
| VHA-E3 | Figura 2.B |
| FdC2 | Figura 2.B |
| VHA-C3 | Figura 2.B |
| AT2G07696.1 | Figura 2.B |
| VHA-A3 | Figura 2.B |
| GUN4 | Figura 2.B |
| RPL24 | Figura 2.B |
| AT5G19940 | Figura 2.B |
| AT4G33420 | Figura 2.B |
| PRXCB | Figura 2.B |
| AT4G11290 | Figura 2.B |
| AT3G28710 | Figura 2.B |
| AKHSDH1 | Figura 2.B |
| TUF | Figura 2.B |
| AT1G12800 | Figura 2.B |
| Lhca6 | Figura 2.B |
| FSD2 | Figura 2.B |
| AT3G49960 | Figura 2.B |
| PER64 | Figura 2.B |
| AT3G04780 | Figura 2.B |
| AT4G08780 | Figura 2.B |
| AT5G02160 | Figura 2.B |
| AT5G35170 | Figura 2.B |
| THM1 | Figura 2.B |
| AT4G30170 | Figura 2.B |
| emb2184 | Figura 2.B |
| PPa3 | Figura 2.B |
| AT5G15180 | Figura 2.B |
| AT5G08680 | Figura 2.B |
| TAPX | Figura 2.B |
| RHS19 | Figura 2.B |
| TRX-M4 | Figura 2.B |
| PER1 | Figura 2.B |
| AT3G17070 | Figura 2.B |
| AVA-P4 | Figura 2.B |
| AT1G75880 | Figura 2.B |
| AT1G67700 | Figura 2.B |
| AT2G38380 | Figura 2.B |
| AT4G16270 | Figura 2.B |
| VAB3 | Figura 2.B |
| EDA9 | Figura 2.B |
| AT2G37130 | Figura 2.B |
| AT3G50990 | Figura 2.B |
| PRPL11 | Figura 2.B |
| AT2G38390 | Figura 2.B |
| AT4G37230 | Figura 2.B |
| AT3G28200 | Figura 2.B |
| AT5G64110 | Figura 2.B |
| PSBA | Figura 2.B |
| AT5G40150 | Figura 2.B |
| PSBC | Figura 2.B |
| PA2 | Figura 2.B |
| AT4G31760 | Figura 2.B |
| AT4G37530 | Figura 2.B |
| AT4G17690 | Figura 2.B |
| UGT72E3 | Figura 2.B |
| PSBB | Figura 2.B |
| PRX52 | Figura 2.B |
| PTAC16 | Figura 2.B |
| UGT72E1 | Figura 2.B |
| PSBD | Figura 2.B |
| AT4G26010 | Figura 2.B |
| AT5G17820 | Figura 2.B |
| ATPE | Figura 2.B |
| DEG1 | Figura 2.B |
| CP29 | Figura 2.B |
| ATPF | Figura 2.B |
| ATPI | Figura 2.B |
| AT5G24070 | Figura 2.B |
| UGT72E2 | Figura 2.B |
| ATPQ | Figura 2.B |
| ATP3 | Figura 2.B |
| ATPC2 | Figura 2.B |
| AT5G51890 | Figura 2.B |
| SIG4 | Figura 2.B |
| AT5G47030 | Figura 2.B |
| AT5G20080 | Figura 2.B |
| AT5G19440 | Figura 2.B |
| AT3G01590 | Figura 2.B |
| AT3G02360 | Figura 2.B |
| AT1G01090.1 | Figura 2.B |
| AT5G64380 | Figura 2.B |
| atp6-1 | Figura 2.B |
| F28P22.13 | Figura 2.B |
| ATP6-2 | Figura 2.B |
| ATP6-1 | Figura 2.B |
| AT5G55220 | Figura 2.B |
| GAPCP-2 | Figura 2.B |
| AT1G64190 | Figura 2.B |
| AT5G41670 | Figura 2.B |
| CCoAOMT1 | Figura 2.B |
| 4CL3 | Figura 2.B |
| HCEF1 | Figura 2.B |
| AT3G25960 | Figura 2.B |
| ATHXK4 | Figura 2.B |
| AT3G61610 | Figura 2.B |
| ATP5 | Figura 2.B |
| AT1G22170 | Figura 2.B |
| NADP-ME3 | Figura 2.B |
| PGR5 | Figura 2.B |
| G6PD3 | Figura 2.B |
| AT5G22620 | Figura 2.B |
| AT5G08570 | Figura 2.B |
| HY2 | Figura 2.B |
| MAB1 | Figura 2.B |
| AT4G26390 | Figura 2.B |
| AT1G59900 | Figura 2.B |
| AT5G04120 | Figura 2.B |
| AT3G50520 | Figura 2.B |
| 4CL5 | Figura 2.B |
| AT5G63680 | Figura 2.B |
| C4H | Figura 2.B |
| NADP-ME1 | Figura 2.B |
| PGRL1B | Figura 2.B |
| AT5G56350 | Figura 2.B |
| AT4G23730 | Figura 2.B |
| PGMP | Figura 2.B |
| GAPC1 | Figura 2.B |
| G6PD5 | Figura 2.B |
| G6PD1 | Figura 2.B |
| NAD-ME1 | Figura 2.B |
| AT2G34590 | Figura 2.B |
| AT5G66530 | Figura 2.B |
| HCT | Figura 2.B |
| ATCCR2 | Figura 2.B |
| AT5G57330 | Figura 2.B |
| iPGAM1 | Figura 2.B |
| AT2G36580 | Figura 2.B |
| AT4G26220 | Figura 2.B |
| AT5G47435 | Figura 2.B |
| 4CL1 | Figura 2.B |
| PGM2 | Figura 2.B |
| HXK3 | Figura 2.B |
| AT1G24735 | Figura 2.B |
| NADP-ME2 | Figura 2.B |
| AT1G78050.1 | Figura 2.B |
| PKP-ALPHA | Figura 2.B |
| TT4 | Figura 2.B |
| IAR4 | Figura 2.B |
| AT4G17560 | Figura 2.B |
| T2H7.8 | Figura 2.B |
| AT4G25900 | Figura 2.B |
| TRA2 | Figura 2.B |
| TPI | Figura 2.B |
| AT3G55810 | Figura 2.B |
| PGM3 | Figura 2.B |
| PFK2 | Figura 2.B |
| ACOS5 | Figura 2.B |
| PFK1 | Figura 2.B |
| G6PD4 | Figura 2.B |
| GAPCP-1 | Figura 2.B |
| 4CL2 | Figura 2.B |
| G6PD2 | Figura 2.B |
| AT3G60750 | Figura 2.B |
| AT5G47190 | Figura 2.B |
| AT5G14500 | Figura 2.B |
| NADP-ME4 | Figura 2.B |
| AT3G04050 | Figura 2.B |
| EMB3105 | Figura 2.B |
| iPGAM2 | Figura 2.B |
| AT3G49160 | Figura 2.B |
| FDH | Figura 2.B |
| PKp3 | Figura 2.B |
| AT1G15950 | Figura 2.B |
| G6PD6 | Figura 2.B |
| AT3G55650 | Figura 2.B |
| AT4G17360 | Figura 2.B |
| PKP-BETA1 | Figura 2.B |
| YUC4 | Figura 2.B |
| YUC5 | Figura 2.B |
| UGT84A2 | Figura 2.B |
| UBP16 | Figura 2.B |
| YUC9 | Figura 2.B |
| F3F9.16 | Figura 2.B |
| AT4G37520 | Figura 2.B |
| YUC3 | Figura 2.B |
| CCOAMT | Figura 2.B |
| AT2G18150 | Figura 2.B |
| AT4G29690 | Figura 2.B |
| ILA | Figura 2.B |
| APK3 | Figura 2.B |
| PSP | Figura 2.B |
| ENOC | Figura 2.B |
| AT1G32780 | Figura 2.B |
| AT4G29680 | Figura 2.B |
| AKN2 | Figura 2.B |
| APK4 | Figura 2.B |
| AT4G24710 | Figura 2.B |
| OMR1 | Figura 2.B |
| YUC6 | Figura 2.B |
| YUC7 | Figura 2.B |
| AT1G77100 | Figura 2.B |
| AT5G04600 | Figura 2.B |
| AT2G18980 | Figura 2.B |
| AT2G34060 | Figura 2.B |
| AT2G24800 | Figura 2.B |
| PYD4 | Figura 2.B |
| AT4G36430 | Figura 2.B |
| AT5G47000 | Figura 2.B |
| AT4G22110 | Figura 2.B |
| ENO1 | Figura 2.B |
| AT5G66390 | Figura 2.B |
| AT2G35380 | Figura 2.B |
| T14E10.210 | Figura 2.B |
| AT3G01190 | Figura 2.B |
| MTHFR1 | Figura 2.B |
| YUC1 | Figura 2.B |
| AT5G03430 | Figura 2.B |
| AT5G06730 | Figura 2.B |
| PRK | Figura 2.B |
| AT2G41480 | Figura 2.B |
| AT5G64100 | Figura 2.B |
| MTHFR2 | Figura 2.B |
| AT5G14130 | Figura 2.B |
| AT2G35120 | Figura 2.B |
| AT4G20930 | Figura 2.B |
| NIR1 | Figura 2.B |
| AT1G30870 | Figura 2.B |
| AT4G00600 | Figura 2.B |
| AT3G03670 | Figura 2.B |
| THA1 | Figura 2.B |
| EMB3127 | Figura 2.B |
| AT2G18140 | Figura 2.B |
| PRXCA | Figura 2.B |
| YUC2 | Figura 2.B |
| AT4G38090 | Figura 2.B |
| VAR2 | Figura 2.B |
| AT1G64710 | Figura 2.B |
| AT4G25980 | Figura 2.B |
| AT2G39040 | Figura 2.B |
| ALDH6B2 | Figura 2.B |
| SR | Figura 2.B |
| TSB2 | Figura 2.B |
| AT3G11250 | Figura 2.B |
| AT1G68850 | Figura 2.B |
| AT1G71500 | Figura 2.B |
| AT2G22420 | Figura 2.B |
| AT1G80380 | Figura 2.B |
| Prx37 | Figura 2.B |
| THA2 | Figura 2.B |
| AT3G12290 | Figura 2.B |
| AGT3 | Figura 2.B |
| GLDP2 | Figura 2.B |
| AT1G49570 | Figura 2.B |
| TSB1 | Figura 2.B |
| GUN5 | Figura 2.B |
| AT5G58400 | Figura 2.B |
| RCI3 | Figura 2.B |
| SHM1 | Figura 2.B |
| HOT5 | Figura 2.B |
| AGT2 | Figura 2.B |
| AT1G14550 | Figura 2.B |
| AT1G71695 | Figura 2.B |
| CSP41A | Figura 2.B |
| AT4G29700 | Figura 2.B |
| APK | Figura 2.B |
| AT4G29710 | Figura 2.B |
| AT1G44000 | Figura 2.B |
| PSB28 | Figura 2.B |
| GDCH | Figura 2.B |
| AT5G51010 | Figura 2.B |
| TOP6B | Figura 2.B |
| THY-1 | Figura 2.B |
| THY-2 | Figura 2.B |
| AT1G22440 | Figura 2.B |
| GSA2 | Figura 2.B |
| SBPASE | Figura 2.B |
| HEMA2 | Figura 2.B |
| AT5G64120 | Figura 2.B |
| CYP38 | Figura 2.B |
| AT4G01150 | Figura 2.B |
| AT5G58390 | Figura 2.B |
| AT2G38660 | Figura 2.B |
| AT3G48420 | Figura 2.B |
| AT3G21770 | Figura 2.B |
| AT4G02610 | Figura 2.B |
| AT5G19880 | Figura 2.B |
| AT5G51970 | Figura 2.B |
| AT1G14345 | Figura 2.B |
| YUC8 | Figura 2.B |
| BETA-UP | Figura 2.B |
| AT1G24110 | Figura 2.B |
| AT1G31220 | Figura 2.B |
| pde194 | Figura 2.B |
| AT5G38520 | Figura 2.B |
| AT5G19890 | Figura 2.B |
| AT1G74730 | Figura 2.B |
| AT1G44970 | Figura 2.B |
| GDC1 | Figura 2.B |
| AT4G02530 | Figura 2.B |
| PnsB1 | Figura 2.B |
| PSAK | Figura 2.B |
| PGRL1A | Figura 2.B |
| ATPD | Figura 2.B |
| PGLP1 | Figura 2.B |
| AT3G13120 | Figura 2.B |
| PDE334 | Figura 2.B |
| NPQ4 | Figura 2.B |
| LQY1 | Figura 2.B |
| FNR2 | Figura 2.B |
| ZKT | Figura 2.B |
| ALDH3F1 | Figura 2.B |
| AT1G22430 | Figura 2.B |
| ATPC1 | Figura 2.B |
| PER4 | Figura 2.B |
| OHP | Figura 2.B |
| AT5G08050 | Figura 2.B |
| GGAT1 | Figura 2.B |
| PRXQ | Figura 2.B |
| SCO1 | Figura 2.B |
| AT3G47070 | Figura 2.B |
| TLP18.3 | Figura 2.B |
| PER2 | Figura 2.B |
| RPS1 | Figura 2.B |
| CRB | Figura 2.B |
| AT2G21550 | Figura 2.B |
| PRXR1 | Figura 2.B |
| PSBQA | Figura 2.B |
| AT3G32980 | Figura 2.B |
| AT2G43480 | Figura 2.B |
| AT1G11860 | Figura 2.B |
| RPSAb | Figura 2.B |
| DRT112 | Figura 2.B |
| HPR | Figura 2.B |
| AT5G07020 | Figura 2.B |
| DFD | Figura 2.B |
| PSBP-1 | Figura 2.B |
| HCF244 | Figura 2.B |
| AT1G34510 | Figura 2.B |
| TRXF1 | Figura 2.B |
| XTH7 | Figura 2.B |
| GAPB | Figura 2.B |
| PETC | Figura 2.B |
| PGK1 | Figura 2.B |
| AT5G28237 | Figura 2.B |
| TSBtype2 | Figura 2.B |
| AT2G43560 | Figura 2.B |
| ACP4 | Figura 2.B |
| PSAN | Figura 2.B |
| THFS | Figura 2.B |
| RHS18 | Figura 2.B |
| ACD2 | Figura 2.B |
| EBS | Figura 2.B |
| PPa2 | Figura 2.B |
| PAL3 | Figura 2.B |
| TRO | Figura 2.B |
| TAP38 | Figura 2.B |
| PPa1 | Figura 2.B |
| PPa5 | Figura 2.B |
| AVP1 | Figura 2.B |
| AT3G26480 | Figura 2.B |
| AT5G46420 | Figura 2.B |
| AT1G58983.1 | Figura 2.B |
| PSAF | Figura 2.B |
| NYE1 | Figura 2.B |
| AT1G07210 | Figura 2.B |
| AT4G35370 | Figura 2.B |
| AT4G23540 | Figura 2.B |
| PYR4 | Figura 2.B |
| PAL4 | Figura 2.B |
| PAL2 | Figura 2.B |
| AT4G04360 | Figura 2.B |
| AT2G32580 | Figura 2.B |
| NKS1 | Figura 2.B |
| PYRD | Figura 2.B |
| AT1G64880 | Figura 2.B |
| AT5G64650 | Figura 2.B |
| AT4G28080 | Figura 2.B |
| AT4G18900 | Figura 2.B |
| AT4G30990 | Figura 2.B |
| AT3G16840 | Figura 2.B |
| AT4G21445 | Figura 2.B |
| AT1G05070 | Figura 2.B |
| AT1G69070 | Figura 2.B |
| AAT | Figura 2.B |
| At3g03600 | Figura 2.B |
| AT1G06720 | Figura 2.B |
| AT2G43030 | Figura 2.B |
| PAL1 | Figura 2.B |
| AT1G42440 | Figura 2.B |
| PPa6 | Figura 2.B |
| AT2G18220 | Figura 2.B |
| AT2G24290 | Figura 2.B |
| emb2394 | Figura 2.B |
| APE2 | Figura 2.B |
| AT5G52490 | Figura 2.B |
| PWP2 | Figura 2.B |
| AT3G02060 | Figura 2.B |
| EDA7 | Figura 2.B |
| PCN | Figura 2.B |
| PYD1 | Figura 2.B |
| AT3G09200 | Figura 2.B |
| FIB2 | Figura 2.B |
| RPS18 | Figura 2.B |
| rps2 | Figura 2.B |
| AT1G10490 | Figura 2.B |
| AT2G18900 | Figura 2.B |
| RRF | Figura 2.B |
| AT4G31520 | Figura 2.B |
| AT1G27461 | Figura 2.B |
| AT1G13160 | Figura 2.B |
| AT2G40360 | Figura 2.B |
| PUM24 | Figura 2.B |
| RPL4 | Figura 2.B |
| AT5G09770 | Figura 2.B |
| AT4G19610 | Figura 2.B |
| AT5G14520 | Figura 2.B |
| AT5G36160 | Figura 2.B |
| TAT7 | Figura 2.B |
| GLDP1 | Figura 2.B |
| AT2G20060 | Figura 2.B |
| emb1211 | Figura 2.B |
| AT5G05720 | Figura 2.B |
| AT3G54210 | Figura 2.B |
| emb2726 | Figura 2.B |
| AT5G15550 | Figura 2.B |
| At1g58380 | Figura 2.B |
| EMB3113 | Figura 2.B |
| EMB3136 | Figura 2.B |
| EDA25 | Figura 2.B |
| AT3G57940 | Figura 2.B |
| AT1G27470 | Figura 2.B |
| AT1G52930 | Figura 2.B |
| AT5G66540 | Figura 2.B |
| PDE338 | Figura 2.B |
| emb1473 | Figura 2.B |
| FIB1 | Figura 2.B |
| CORI3 | Figura 2.B |
| AT2G34357 | Figura 2.B |
| AT2G21440 | Figura 2.B |
| AT3G23700 | Figura 2.B |
| AT4G04940 | Figura 2.B |
| AT4G23590 | Figura 2.B |
| T22E16.170 | Figura 2.B |
| AT5G08695 | Figura 2.B |
| AT3G06530 | Figura 2.B |
| AT3G15460 | Figura 2.B |
| EMB2762 | Figura 2.B |
| RABE1b | Figura 2.B |
| AT1G63810 | Figura 2.B |
| AT4G02930 | Figura 2.B |
| AT3G12370 | Figura 2.B |
| AT1G25260 | Figura 2.B |
| AT3G23620 | Figura 2.B |
| AT4G18905 | Figura 2.B |
| DIN10 | Figura 2.B |
| HEXO3 | Figura 2.B |
| STS | Figura 2.B |
| SIP2 | Figura 2.B |
| AT1G62660 | Figura 2.B |
| RFS1 | Figura 2.B |
| AT3G56310 | Figura 2.B |
| cwINV4 | Figura 2.B |
| MDAR6 | Figura 2.B |
| CWINV5 | Figura 2.B |
| cwINV6 | Figura 2.B |
| AGAL2 | Figura 2.B |
| PGR7 | Figura 2.B |
| SAPX | Figura 2.B |
| HEMA1 | Figura 2.B |
| ATMDAR2 | Figura 2.B |
| EP3 | Figura 2.B |
| HEXO1 | Figura 2.B |
| MDAR4 | Figura 2.B |
| GLDH | Figura 2.B |
| PSAE-1 | Figura 2.B |
| AGAL1 | Figura 2.B |
| AT4G03460 | Figura 2.B |
| AT3G61870 | Figura 2.B |
| PSAD-2 | Figura 2.B |
| AT1G66430 | Figura 2.B |
| GAPA-2 | Figura 2.B |
| LHCB4.1 | Figura 2.B |
| ATBFRUCT1 | Figura 2.B |
| RFS5 | Figura 2.B |
| PSBO1 | Figura 2.B |
| PSBX | Figura 2.B |
| PSBQ-2 | Figura 2.B |
| FBA1 | Figura 2.B |
| LHCA1 | Figura 2.B |
| PSBY | Figura 2.B |
| LHCA2 | Figura 2.B |
| PSBO2 | Figura 2.B |
| LHCB3 | Figura 2.B |
| CAB3 | Figura 2.B |
| PETE1 | Figura 2.B |
| PSAD-1 | Figura 2.B |
| AT1G51400 | Figura 2.B |
| PSAA | Figura 2.B |
| LHCB6 | Figura 2.B |
| GSA1 | Figura 2.B |
| LHCA4 | Figura 2.B |
| HEMA3 | Figura 2.B |
| PSBTN | Figura 2.B |
| GAPA | Figura 2.B |
| PSAG | Figura 2.B |
| LHCA3 | Figura 2.B |
| LHCB4.2 | Figura 2.B |
| CRD1 | Figura 2.B |
| PSAE-2 | Figura 2.B |
| AT1G65230 | Figura 2.B |
| LHB1B1 | Figura 2.B |
| PSAH-1 | Figura 2.B |
| LHCB5 | Figura 2.B |
| PSAO | Figura 2.B |
| PSAL | Figura 2.B |
| CaS | Figura 2.B |
| FNR1 | Figura 2.B |
| PORB | Figura 2.B |
| PPL1 | Figura 2.B |
| PORC | Figura 2.B |
| PSBR | Figura 2.B |
| CH1 | Figura 2.B |
| FLU | Figura 2.B |
| VTE1 | Figura 2.B |
| NYC1 | Figura 2.B |
| APG1 | Figura 2.B |
| HCAR | Figura 2.B |
| CLH1 | Figura 2.B |
| AT1G31910 | Figura 2.B |
| MENG | Figura 2.B |
| AT3G24200 | Figura 2.B |
| NOL | Figura 2.B |
| PDS1 | Figura 2.B |
| AOAT2 | Figura 2.B |
| ICL | Figura 2.B |
| CAD2 | Figura 2.B |
| CAD3 | Figura 2.B |
| MLS | Figura 2.B |
| ERA1 | Figura 2.B |
| AGT | Figura 2.B |
| AT5G36790 | Figura 2.B |
| CAD9 | Figura 2.B |
| ELI3-2 | Figura 2.B |
| OMT1 | Figura 2.B |
| SQS2 | Figura 2.B |
| ATCAD4 | Figura 2.B |
| SQS1 | Figura 2.B |
| AT4G35090 | Figura 2.B |
| CAD6 | Figura 2.B |
| CYP84A4 | Figura 2.B |
| FTA | Figura 2.B |
| PMDH2 | Figura 2.B |
| CAD5 | Figura 2.B |
| AT1G74470 | Figura 2.B |
| HDS | Figura 2.B |
| fah1 | Figura 2.B |
| SKL2 | Figura 2.B |
| PSAH2 | Figura 2.B |
| PSI-P | Figura 2.B |
| AT1G52220 | Figura 2.B |
| ALDH7B4 | Figura 2.B |
| AT5G42250 | Figura 2.B |
| NRPB6B | Figura 2.B |
| AT2G37660 | Figura 2.B |
| HDA14 | Figura 2.B |
| BKI1 | Figura 2.C |
| TPS9 | Figura 2.C |
| TPS8 | Figura 2.C |
| MEE14 | Figura 2.C |
| RMA1 | Figura 2.C |
| ALNS | Figura 2.C |
| SIS | Figura 2.C |
| AT4G36180 | Figura 2.C |
| AT3G28040 | Figura 2.C |
| GSO2 | Figura 2.C |
| RbcX1 | Figura 2.C |
| AT3G47570 | Figura 2.C |
| AT5G63930 | Figura 2.C |
| AT3G62550 | Figura 2.C |
| J11 | Figura 2.C |
| GSO1 | Figura 2.C |
| fls2 | Figura 2.C |
| XBAT31 | Figura 2.C |
| CCL | Figura 2.C |
| AT4G32340 | Figura 2.C |
| EFR | Figura 2.C |
| AT1G75640 | Figura 2.C |
| AT5G49770 | Figura 2.C |
| RIBA2 | Figura 2.C |
| PSY1R | Figura 2.C |
| AT3G47160 | Figura 2.C |
| BRI1 | Figura 2.C |
| AT1G74360 | Figura 2.C |
| AT3G49670.1 | Figura 2.C |
| MYBL2 | Figura 2.C |
| AT5G25930 | Figura 2.C |
| AT4G28650 | Figura 2.C |
| WCRKC1 | Figura 2.C |
| BSK8 | Figura 2.C |
| AT3G47090 | Figura 2.C |
| BSK1 | Figura 2.C |
| BRL3 | Figura 2.C |
| ERL2 | Figura 2.C |
| PEPR1 | Figura 2.C |
| HSL2 | Figura 2.C |
| EXL2 | Figura 2.C |
| PSKR2 | Figura 2.C |
| ERD15 | Figura 2.C |
| AT3G47110 | Figura 2.C |
| BAK1 | Figura 2.C |
| ERECTA | Figura 2.C |
| SERK1 | Figura 2.C |
| BIK1 | Figura 2.C |
| KAPP | Figura 2.C |
| EMS1 | Figura 2.C |
| AT2G24130 | Figura 2.C |
| AT3G47580 | Figura 2.C |
| AT1G80440 | Figura 2.C |
| Fes1B | Figura 2.C |
| BSK3 | Figura 2.C |
| AT5G07580 | Figura 2.C |
| ERL1 | Figura 2.C |
| AT5G37450 | Figura 2.C |
| RPK2 | Figura 2.C |
| J8 | Figura 2.C |
| AT4G05070 | Figura 2.C |
| AT5G65700.1 | Figura 2.C |
| BRL2 | Figura 2.C |
| AT5G61590 | Figura 2.C |
| BZO2H3 | Figura 2.C |
| SERK4 | Figura 2.C |
| TPS5 | Figura 2.C |
| AT5G08520 | Figura 2.C |
| TRE1 | Figura 2.C |
| TPS4 | Figura 2.C |
| T13D8.4 | Figura 2.C |
| ATTPS6 | Figura 2.C |
| TPS7 | Figura 2.C |
| TPS11 | Figura 2.C |
| TPS2 | Figura 2.C |
| TPS1 | Figura 2.C |
| EBS5 | Figura 2.C |
| AT2G33830 | Figura 2.C |
| AT3G15450 | Figura 2.C |
| AT5G19120 | Figura 2.C |
| AT1G23390 | Figura 2.C |
| AT2G40860 | Figura 2.C |
| AT3G15630 | Figura 2.C |
| TPS3 | Figura 2.C |
| AT5G22920 | Figura 2.C |
| EIF3A | Figura 2.A |
| SNRK2.10 | Figura 2.A |
| SK10 | Figura 2.A |
| ASN3 | Figura 2.A |
| ALDH3I1 | Figura 2.A |
| AT4G12780 | Figura 2.A |
| TOR | Figura 2.A |
| AT4G08876 | Figura 2.A |
| RLP7 | Figura 2.A |
| ADSS | Figura 2.A |
| SK4 | Figura 2.A |
| SK21 | Figura 2.A |
| EIF3E | Figura 2.A |
| AT5G42740 | Figura 2.A |
| Hsp70-15 | Figura 2.A |
| AT1G76810 | Figura 2.A |
| AT1G21160 | Figura 2.A |
| AT5G08180 | Figura 2.A |
| AT1G76720 | Figura 2.A |
| AT2G27700 | Figura 2.A |
| AT1G76820 | Figura 2.A |
| WEE1 | Figura 2.A |
| AT5G02530 | Figura 2.A |
| RAD50 | Figura 2.A |
| MRE11 | Figura 2.A |
| AT2G33840 | Figura 2.A |
| KU80 | Figura 2.A |
| KU70 | Figura 2.A |
| AT1G28350 | Figura 2.A |
| ALY4 | Figura 2.A |
| MSH2 | Figura 2.A |
| TERT | Figura 2.A |
| MSH5 | Figura 2.A |
| AT1G29630 | Figura 2.A |
| AT3G21300 | Figura 2.A |
| MNS3 | Figura 2.A |
| SAUL1 | Figura 2.A |
| LIG6 | Figura 2.A |
| AT2G02550 | Figura 2.A |
| AT3G14890 | Figura 2.A |
| APTX | Figura 2.A |
| ATM | Figura 2.A |
| MLH1 | Figura 2.A |
| WRNEXO | Figura 2.A |
| ATLIG4 | Figura 2.A |
| PMS1 | Figura 2.A |
| PUB14 | Figura 2.A |
| AT1G18090 | Figura 2.A |
| AT2G32170 | Figura 2.A |
| AT4G12600 | Figura 2.A |
| PUB9 | Figura 2.A |
| CHR1 | Figura 2.A |
| HTA12 | Figura 2.A |
| RAD54 | Figura 2.A |
| VIP5 | Figura 2.A |
| PPAN | Figura 2.A |
| VIP4 | Figura 2.A |
| AT3G09480 | Figura 2.A |
| AT5G20160 | Figura 2.A |
| HTA10 | Figura 2.A |
| CKA2 | Figura 2.A |
| AT3G30430 | Figura 2.A |
| MCM4 | Figura 2.A |
| CKB1 | Figura 2.A |
| SPT16 | Figura 2.A |
| AT5G02570 | Figura 2.A |
| GAMMA-H2AX | Figura 2.A |
| HTA13 | Figura 2.A |
| AT5G59690.1 | Figura 2.A |
| AT2G35280 | Figura 2.A |
| CHR4 | Figura 2.A |
| AT3G45930.1 | Figura 2.A |
| HTA4 | Figura 2.A |
| HTA7 | Figura 2.A |
| AT4G22380 | Figura 2.A |
| AT1G07660 | Figura 2.A |
| AT1G05910 | Figura 2.A |
| AT5G59970.1 | Figura 2.A |
| HTA9 | Figura 2.A |
| AT3G46320.1 | Figura 2.A |
| HTB2 | Figura 2.A |
| LEC1 | Figura 2.A |
| ASG3 | Figura 2.A |
| CHR11 | Figura 2.A |
| HTA8 | Figura 2.A |
| AT3G16600 | Figura 2.A |
| AT2G37470 | Figura 2.A |
| AT1G74875 | Figura 2.A |
| CHR18 | Figura 2.A |
| AT5G59950 | Figura 2.A |
| AT2G04740 | Figura 2.A |
| HTA11 | Figura 2.A |
| HTB11 | Figura 2.A |
| AT2G28720 | Figura 2.A |
| HTB9 | Figura 2.A |
| GTA2 | Figura 2.A |
| AT3G54460 | Figura 2.A |
| TOPII | Figura 2.A |
| RAT5 | Figura 2.A |
| AT1G08170 | Figura 2.A |
| AT1G07660.1 | Figura 2.A |
| ASHH1 | Figura 2.A |
| AT1G63210 | Figura 2.A |
| HTB1 | Figura 2.A |
| CHR17 | Figura 2.A |
| RPA70C | Figura 2.A |
| AT5G07810 | Figura 2.A |
| AT1G07820.1 | Figura 2.A |
| CSN5B | Figura 2.A |
| CKB4 | Figura 2.A |
| AT2G23070 | Figura 2.A |
| SYD | Figura 2.A |
| BRM | Figura 2.A |
| NF-YB2 | Figura 2.A |
| HTB4 | Figura 2.A |
| GTC2 | Figura 2.A |
| ELF8 | Figura 2.A |
| AT5G17010 | Figura 2.A |
| AT1G11100 | Figura 2.A |
| AT1G60900 | Figura 2.A |
| CKA3 | Figura 2.A |
| FAS2 | Figura 2.A |
| CAND1 | Figura 2.A |
| GTB1 | Figura 2.A |
| ALY2 | Figura 2.A |
| At3g22480 | Figura 2.A |
| NPC4 | Figura 2.A |
| PFD1 | Figura 2.A |
| AT3G53650 | Figura 2.A |
| AT2G32415 | Figura 2.A |
| PKR2 | Figura 2.A |
| RPA70B | Figura 2.A |
| AT1G60830 | Figura 2.A |
| AT3G15120 | Figura 2.A |
| AT2G33440 | Figura 2.A |
| AT5G41560 | Figura 2.A |
| DCAF1 | Figura 2.A |
| AT2G24350 | Figura 2.A |
| PHP | Figura 2.A |
| CKB3 | Figura 2.A |
| CHR34 | Figura 2.A |
| SLD5 | Figura 2.A |
| ASF1B | Figura 2.A |
| HIRA | Figura 2.A |
| CHR5 | Figura 2.A |
| AT5G35910 | Figura 2.A |
| AT3G17380 | Figura 2.A |
| AT2G34210 | Figura 2.A |
| CKA1 | Figura 2.A |
| AT1G54440 | Figura 2.A |
| KTF1 | Figura 2.A |
| ELF7 | Figura 2.A |
| SGA2 | Figura 2.A |
| HTA2 | Figura 2.A |
| NF-YB4 | Figura 2.A |
| NF-YB8 | Figura 2.A |
| HIS4 | Figura 2.A |
| COP1 | Figura 2.A |
| RIN1 | Figura 2.A |
| DWA1 | Figura 2.A |
| CHR40 | Figura 2.A |
| PRL1 | Figura 2.A |
| AT1G52950 | Figura 2.A |
| NF-YB3 | Figura 2.A |
| MCM5 | Figura 2.A |
| ELF6 | Figura 2.A |
| HAT3.1 | Figura 2.A |
| CSN5A | Figura 2.A |
| EER5 | Figura 2.A |
| AT4G28310 | Figura 2.A |
| DET1 | Figura 2.A |
| H2AXA | Figura 2.A |
| CKB2 | Figura 2.A |
| HTA6 | Figura 2.A |
| AT3G32280 | Figura 2.A |
| IMK2 | Figura 2.A |
| VPS34 | Figura 2.A |
| CHR42 | Figura 2.A |
| CHR38 | Figura 2.A |
| AT2G40770 | Figura 2.A |
| AT5G54970 | Figura 2.A |
| CPL1 | Figura 2.A |
| SAC3B | Figura 2.A |
| AT3G31900 | Figura 2.A |
| EFS | Figura 2.A |
| SAC3C | Figura 2.A |
| PFD3 | Figura 2.A |
| AIP3 | Figura 2.A |
| AT1G66260 | Figura 2.A |
| REF6 | Figura 2.A |
| NF-YB10 | Figura 2.A |
| ATRX | Figura 2.A |
| At2g31630 | Figura 2.A |
| RPA1A | Figura 2.A |
| AT3G32100 | Figura 2.A |
| ICU2 | Figura 2.A |
| ATX2 | Figura 2.A |
| AT1G36030 | Figura 2.A |
| chr31 | Figura 2.A |
| RPA70D | Figura 2.A |
| DWA2 | Figura 2.A |
| NF-YB6 | Figura 2.A |
| AT1G36510 | Figura 2.A |
| DRD1 | Figura 2.A |
| ATU2AF65A | Figura 2.A |
| UAP56a | Figura 2.A |
| AT3G32260 | Figura 2.A |
| MOM | Figura 2.A |
| NF-YB7 | Figura 2.A |
| NAD-ME2 | Figura 2.A |
| COP8 | Figura 2.A |
| DUT1 | Figura 2.A |
| AT1G05120 | Figura 2.A |
| PPC4 | Figura 2.A |
| PPC2 | Figura 2.A |
| PFD5 | Figura 2.A |
| DDB2 | Figura 2.A |
| ROC1 | Figura 2.A |
| AT1G21780 | Figura 2.A |
| AT1G67623 | Figura 2.A |
| NF-YB5 | Figura 2.A |
| CPL2 | Figura 2.A |
| AT3G32330 | Figura 2.A |
| PGI1 | Figura 2.A |
| AT2G05642 | Figura 2.A |
| AT4G19130 | Figura 2.A |
| NF-YB1 | Figura 2.A |
| CXE18 | Figura 2.A |
| PPC1 | Figura 2.A |
| AT4G37560 | Figura 2.A |
| SQN | Figura 2.A |
| PPC3 | Figura 2.A |
| AT2G47090 | Figura 2.A |
| AT1G47400 | Figura 2.A |
| AT5G05250 | Figura 2.A |
| SIR | Figura 2.A |
| AT3G62240 | Figura 2.A |
| NSF | Figura 2.A |
| PANC | Figura 2.A |
| VHA-A | Figura 2.A |
| TUB4 | Figura 2.A |
| TPR10 | Figura 2.A |
| VAB1 | Figura 2.A |
| DET3 | Figura 2.A |
| TTL3 | Figura 2.A |
| AT1G56500 | Figura 2.A |
| TOC64-III | Figura 2.A |
| DGK1 | Figura 2.A |
| CR88 | Figura 2.A |
| PANK2 | Figura 2.A |
| AT5G10370 | Figura 2.A |
| AT1G29310 | Figura 2.A |
| LPLAT2 | Figura 2.A |
| CA1 | Figura 2.A |
| PIS1 | Figura 2.A |
| AT2G45030 | Figura 2.A |
| AT1G63660 | Figura 2.A |
| TTL4 | Figura 2.A |
| AT1G57720 | Figura 2.A |
| PCNA2 | Figura 2.A |
| UBP27 | Figura 2.A |
| AT3G04480 | Figura 2.A |
| P5CS2 | Figura 2.A |
| AT2G01250 | Figura 2.A |
| SHD | Figura 2.A |
| HSP90.1 | Figura 2.A |
| HAP6 | Figura 2.A |
| AT4G01020 | Figura 2.A |
| P5CS1 | Figura 2.A |
| NIA1 | Figura 2.A |
| CDS5 | Figura 2.A |
| LPAT5 | Figura 2.A |
| AT2G35040 | Figura 2.A |
| DGK7 | Figura 2.A |
| AT4G26770 | Figura 2.A |
| AT4G14250 | Figura 2.A |
| AT4G16660 | Figura 2.A |
| TTL1 | Figura 2.A |
| AT5G10540 | Figura 2.A |
| AT1G09640 | Figura 2.A |
| DGK2 | Figura 2.A |
| PIS2 | Figura 2.A |
| AT1G79990 | Figura 2.A |
| AT1G45332 | Figura 2.A |
| HSP81-3 | Figura 2.A |
| TOC64-V | Figura 2.A |
| DGL1 | Figura 2.A |
| KEG | Figura 2.A |
| NBR1 | Figura 2.A |
| AT5G13400 | Figura 2.A |
| KAK | Figura 2.A |
| SDP1-LIKE | Figura 2.A |
| PAH1 | Figura 2.A |
| AT2G34250 | Figura 2.A |
| AT1G24510 | Figura 2.A |
| AT5G58410 | Figura 2.A |
| CDS2 | Figura 2.A |
| DGK5 | Figura 2.A |
| LPAT2 | Figura 2.A |
| CLS | Figura 2.A |
| CDS1 | Figura 2.A |
| CA2 | Figura 2.A |
| LPAT3 | Figura 2.A |
| ATG18B | Figura 2.A |
| AT2G41620 | Figura 2.A |
| AT1G72730 | Figura 2.A |
| AT4G33945 | Figura 2.A |
| HOG1 | Figura 2.A |
| emb1441 | Figura 2.A |
| PGP1 | Figura 2.A |
| SCY2 | Figura 2.A |
| NAP57 | Figura 2.A |
| AT3G54470 | Figura 2.A |
| SDP1 | Figura 2.A |
| SCY1 | Figura 2.A |
| AT3G28870 | Figura 2.A |
| AT5G60460 | Figura 2.A |
| ATS2 | Figura 2.A |
| AT3G22980 | Figura 2.A |
| ALDH2C4 | Figura 2.A |
| NIA2 | Figura 2.A |
| SMO1-3 | Figura 2.A |
| TTL2 | Figura 2.A |
| DGK6 | Figura 2.A |
| LPLAT1 | Figura 2.A |
| RAN-1 | Figura 2.A |
| AT3G20920 | Figura 2.A |
| BSL2 | Figura 2.A |
| LPAT4 | Figura 2.A |
| AT4G21110 | Figura 2.A |
| PGPS2 | Figura 2.A |
| AT5G02680 | Figura 2.A |
| ALATS | Figura 2.A |
| OPT7 | Figura 2.A |
| GAPC2 | Figura 2.A |
| Hsp81.4 | Figura 2.A |
| PCNA1 | Figura 2.A |
| AT1G78720 | Figura 2.A |
| PI4KALPHA1 | Figura 2.A |
| UBP20 | Figura 2.A |
| AT2G43240 | Figura 2.A |
| DGK3 | Figura 2.A |
| RAN2 | Figura 2.A |
| AT3G01340 | Figura 2.A |
| AT1G51040 | Figura 2.A |
| UPL4 | Figura 2.A |
| AT1G58684 | Figura 2.A |
| AT4G24920 | Figura 2.A |
| TIM | Figura 2.A |
| PUR4 | Figura 2.A |
| TSK | Figura 2.A |
| PGK | Figura 2.A |
| FUM1 | Figura 2.A |
| DGK4 | Figura 2.A |
| PAH2 | Figura 2.A |
| CPN60B | Figura 2.A |
| RAN3 | Figura 2.A |
| OPT4 | Figura 2.A |
| OPT6 | Figura 2.A |
| RSR4 | Figura 2.A |
| OPT1 | Figura 2.A |
| AT1G71060 | Figura 2.A |
| EMB2744 | Figura 2.A |
| OPT2 | Figura 2.A |
| OPT5 | Figura 2.A |
| SECE1 | Figura 2.A |
| ALB3 | Figura 2.A |
| ADG1 | Figura 2.A |
| OPT8 | Figura 2.A |
| OPT3 | Figura 2.A |
| EXLA1 | Figura 2.A |
| OPT9 | Figura 2.A |
| AT5G14460 | Figura 2.A |
| BUB3.2 | Figura 2.A |
| UBC14 | Figura 2.A |
| AT5G40880 | Figura 2.A |
| ALN | Figura 2.A |
| SKIP16 | Figura 2.A |
| CARA | Figura 2.A |
| BUB3.1 | Figura 2.A |
| AT1G69400 | Figura 2.A |
| BUBR1 | Figura 2.A |
| MAPKKK7 | Figura 2.A |
| RUB3 | Figura 2.A |
| UBC13 | Figura 2.A |
| CUL2 | Figura 2.A |
| CUL3B | Figura 2.A |
| RUB1 | Figura 2.A |
| AT1G53980 | Figura 2.A |
| AT3G11130 | Figura 2.A |
| AT5G37340 | Figura 2.A |
| AT2G46500 | Figura 2.A |
| AT5G11710 | Figura 2.A |
| DL1 | Figura 2.A |
| alpha-ADR | Figura 2.A |
| AT1G08670 | Figura 2.A |
| ERMO2 | Figura 2.A |
| AT5G47320.1 | Figura 2.A |
| AT4G01000 | Figura 2.A |
| AT3G24530 | Figura 2.A |
| AT3G59290 | Figura 2.A |
| AT1G20760 | Figura 2.A |
| CYTC-1 | Figura 2.A |
| AT1G27530 | Figura 2.A |
| AT1G14570 | Figura 2.A |
| CDC48B | Figura 2.A |
| AT1G43140 | Figura 2.A |
| UFD1 | Figura 2.A |
| AT1G53930 | Figura 2.A |
| NPL41 | Figura 2.A |
| AT4G04180 | Figura 2.A |
| AT3G18860 | Figura 2.A |
| AT4G32640 | Figura 2.A |
| AT4G24550 | Figura 2.A |
| AT3G46540 | Figura 2.A |
| CDC48 | Figura 2.A |
| CYTC-2 | Figura 2.A |
| AT1G59800 | Figura 2.A |
| AtCDC48B | Figura 2.A |
| AT5G24240 | Figura 2.A |
| AT4G15420 | Figura 2.A |
| AT2G20790 | Figura 2.A |
| AT3G23350 | Figura 2.A |
| AT2G29070 | Figura 2.A |
| PK1 | Figura 2.A |
| AT4G38930 | Figura 2.A |
| AT2G47970 | Figura 2.A |
| HAP13 | Figura 2.A |
| UBQ12 | Figura 2.A |
| AtCDC48C | Figura 2.A |
| CUL1 | Figura 2.A |
| S6K2 | Figura 2.A |
| AT5G49200 | Figura 2.A |
| AT1G64470 | Figura 2.A |
| AT1G53950 | Figura 2.A |
| AT4G02620 | Figura 2.A |
| AT1G11970 | Figura 2.A |
| UBC7 | Figura 2.A |
| AT1G59790 | Figura 2.A |
| UCH3 | Figura 2.A |
| AT2G43160 | Figura 2.A |
| AT5G22480 | Figura 2.A |
| AT1G25240 | Figura 2.A |
| AT1G10730 | Figura 2.A |
| AT1G15890 | Figura 2.A |
| AT5G22780 | Figura 2.A |
| PP2AB2 | Figura 2.A |
| TAF15b | Figura 2.A |
| PP2AA2 | Figura 2.A |
| SRX | Figura 2.A |
| PP2AB1 | Figura 2.A |
| AT1G67325 | Figura 2.A |
| AT3G26020 | Figura 2.A |
| AT1G77550 | Figura 2.A |
| CEF | Figura 2.A |
| CCP2 | Figura 2.A |
| UBP24 | Figura 2.A |
| ACLB-1 | Figura 2.A |
| mMDH1 | Figura 2.A |
| CSY1 | Figura 2.A |
| CSY3 | Figura 2.A |
| SDH2-1 | Figura 2.A |
| AT5G23250 | Figura 2.A |
| MDH | Figura 2.A |
| ASP3 | Figura 2.A |
| KAT5 | Figura 2.A |
| CSY5 | Figura 2.A |
| SDH3-2 | Figura 2.A |
| PMDH1 | Figura 2.A |
| ACLB-2 | Figura 2.A |
| ACLA-2 | Figura 2.A |
| SDH2-2 | Figura 2.A |
| AT5G55070 | Figura 2.A |
| SDH1-1 | Figura 2.A |
| AT2G47610 | Figura 2.A |
| PKT4 | Figura 2.A |
| c-NAD-MDH3 | Figura 2.A |
| ATCS | Figura 2.A |
| AT3G62870 | Figura 2.A |
| AT1G36280 | Figura 2.A |
| BCE2 | Figura 2.A |
| SDH3-1 | Figura 2.A |
| AT3G08530 | Figura 2.A |
| PMI1 | Figura 2.A |
| AT3G55410 | Figura 2.A |
| AT5G10920 | Figura 2.A |
| ACLA-3 | Figura 2.A |
| MAK10 | Figura 2.A |
| ASP5 | Figura 2.A |
| AT5G58330 | Figura 2.A |
| AT4G26910 | Figura 2.A |
| c-NAD-MDH1 | Figura 2.A |
| ASP4 | Figura 2.A |
| PCK1 | Figura 2.A |
| AT4G18440 | Figura 2.A |
| c-NAD-MDH2 | Figura 2.A |
| AT5G02080 | Figura 2.A |
| LTA3 | Figura 2.A |
| AT4G17260 | Figura 2.A |
| PCK2 | Figura 2.A |
| AT4G09784 | Figura 2.A |
| AT5G12040 | Figura 2.A |
| AT2G37500 | Figura 2.A |
| SDH2-3 | Figura 2.A |
| ASP1 | Figura 2.A |
| AT3G13930 | Figura 2.A |
| SDH1-2 | Figura 2.A |
| AT5G08300 | Figura 2.A |
| HDA08 | Figura 2.A |
| AT5G65750 | Figura 2.A |
| ATMAK3 | Figura 2.A |
| CSY2 | Figura 2.A |
| HDA18 | Figura 2.A |
| AT2G26800 | Figura 2.A |
| ACO3 | Figura 2.A |
| PREP1 | Figura 2.A |
| mMDH2 | Figura 2.A |
| EMB3003 | Figura 2.A |
| AT2G41790 | Figura 2.A |
| ASP2 | Figura 2.A |
| CKS1 | Figura 2.A |
| PKT3 | Figura 2.A |
| F4B14.100 | Figura 2.A |
| LTA2 | Figura 2.A |
| At3g03780 | Figura 2.A |
| PREP2 | Figura 2.A |
| ATMS1 | Figura 2.A |
| ALDH5F1 | Figura 2.A |
| NAGS2 | Figura 2.A |
| AT3G53910 | Figura 2.A |
| HDA05 | Figura 2.A |
| AT1G34300 | Figura 2.A |
| AT1G54220 | Figura 2.A |
| At4g26970 | Figura 2.A |
| OASB | Figura 2.A |
| DELTA-OAT | Figura 2.A |
| WIN1 | Figura 2.A |
| AT5G16280 | Figura 2.A |
| ASN2 | Figura 2.A |
| NAGS1 | Figura 2.A |
| EMB2753 | Figura 2.A |
| AT5G58450 | Figura 2.A |
| HDA15 | Figura 2.A |
| EBS1 | Figura 2.A |
| P5CR | Figura 2.A |
| AT1G10520 | Figura 2.A |
| ASN1 | Figura 2.A |
| PLDALPHA3 | Figura 2.A |
| MS3 | Figura 2.A |
| PLDALPHA2 | Figura 2.A |
| AT1G25083.1 | Figura 2.A |
| AT2G16960 | Figura 2.A |
| PLDGAMMA2 | Figura 2.A |
| PLDP1 | Figura 2.A |
| AT5G27410 | Figura 2.A |
| AT1G24909 | Figura 2.A |
| PEX5 | Figura 2.A |
| PLDP2 | Figura 2.A |
| DIC3 | Figura 2.A |
| ACLA-1 | Figura 2.A |
| AT1G05720 | Figura 2.A |
| AT1G24807 | Figura 2.A |
| AT5G19760 | Figura 2.A |
| PLDBETA1 | Figura 2.A |
| PLDALPHA1 | Figura 2.A |
| ATTRN1 | Figura 2.A |
| AT2G11270 | Figura 2.A |
| PLDDELTA | Figura 2.A |
| PLDGAMMA3 | Figura 2.A |
| PLDBETA2 | Figura 2.A |
| pat1 | Figura 2.A |
| AT5G42470 | Figura 2.A |
| PIN1AT | Figura 2.A |
| BCAT3 | Figura 2.A |
| CYL1 | Figura 2.A |
| AT1G25155.1 | Figura 2.A |
| AT1G74780 | Figura 2.A |
| PNG1 | Figura 2.A |
| PLDEPSILON | Figura 2.A |
| AT1G60080 | Figura 2.A |
| BI1 | Figura 2.A |
| PLDGAMMA1 | Figura 2.A |
| AT1G30580 | Figura 2.A |
| AT4G27280 | Figura 2.A |
| AT1G73110 | Figura 2.A |
| ATSUV3 | Figura 2.A |
| UCH2 | Figura 2.A |
| PBD2 | Figura 2.A |
| UBP6 | Figura 2.A |
| PBD1 | Figura 2.A |
| FUS12 | Figura 2.A |
| EMB2107 | Figura 2.A |
| HMGS | Figura 2.A |
| AT5G65740 | Figura 2.A |
| HMG1 | Figura 2.A |
| STI | Figura 2.A |
| BRCC36A | Figura 2.A |
| AT1G80630 | Figura 2.A |
| AT1G14460 | Figura 2.A |
| AT5G05510 | Figura 2.A |
| NBS1 | Figura 2.A |
| MAD2 | Figura 2.A |
| MEE32 | Figura 2.A |
| PRT6 | Figura 2.A |
| AT1G53790 | Figura 2.A |
| DAD1 | Figura 2.A |
| RCA | Figura 2.A |
| UBP7 | Figura 2.A |
| BRCC36B | Figura 2.A |
| RLP14 | Figura 2.A |
| CTF18 | Figura 2.A |
| UCH1 | Figura 2.A |
| RPN13 | Figura 2.A |
| AT2G46520 | Figura 2.A |
| AT3G15180 | Figura 2.A |
| AT1G52530 | Figura 2.A |
| F24B18.11 | Figura 2.A |
| DCL1 | Figura 2.A |
| PPK1 | Figura 2.A |
| CML19 | Figura 2.A |
| POLA3 | Figura 2.A |
| CER7 | Figura 2.A |
| AT4G24790 | Figura 2.A |
| AT2G46600 | Figura 2.A |
| AT2G25355 | Figura 2.A |
| AT2G20635 | Figura 2.A |
| AT4G32175 | Figura 2.A |
| AT1G61000 | Figura 2.A |
| LOX5 | Figura 2.A |
| AT1G60995 | Figura 2.A |
| AT4G14970 | Figura 2.A |
| AT4G18820 | Figura 2.A |
| AT3G54630 | Figura 2.A |
| AT1G07270 | Figura 2.A |
| CML20 | Figura 2.A |
| MAD1 | Figura 2.A |
| MK | Figura 2.A |
| LOX3 | Figura 2.A |
| PLA2-ALPHA | Figura 2.A |
| AT5G45720 | Figura 2.A |
| AT5G25080 | Figura 2.A |
| RPA32B | Figura 2.A |
| LOX1 | Figura 2.A |
| AT1G12470 | Figura 2.A |
| MCM9 | Figura 2.A |
| AT1G77680 | Figura 2.A |
| RAN4 | Figura 2.A |
| DOX1 | Figura 2.A |
| AT3G07750 | Figura 2.A |
| AT3G46210 | Figura 2.A |
| DCL3 | Figura 2.A |
| EMB1401 | Figura 2.A |
| AT5G49110 | Figura 2.A |
| EMB1968 | Figura 2.A |
| LOX4 | Figura 2.A |
| RRP41 | Figura 2.A |
| AT3G02820 | Figura 2.A |
| AT5G38890 | Figura 2.A |
| CUL3 | Figura 2.A |
| POLD2 | Figura 2.A |
| MEI1 | Figura 2.A |
| RFC2 | Figura 2.A |
| RRP4 | Figura 2.A |
| AT5G39840 | Figura 2.A |
| ATIM | Figura 2.A |
| POLD4 | Figura 2.A |
| POLA2 | Figura 2.A |
| ATR | Figura 2.A |
| EMB2780 | Figura 2.A |
| SOS2 | Figura 2.A |
| RRP45a | Figura 2.A |
| RRP41L | Figura 2.A |
| LIG1 | Figura 2.A |
| BPM2 | Figura 2.A |
| RAP2.4 | Figura 2.A |
| HAG2 | Figura 2.A |
| CUL4 | Figura 2.A |
| DCL2 | Figura 2.A |
| AT4G12100 | Figura 2.A |
| AT4G02110 | Figura 2.A |
| NAC083 | Figura 2.A |
| AT5G10630 | Figura 2.A |
| AT3G46910 | Figura 2.A |
| RFC1 | Figura 2.A |
| PRL | Figura 2.A |
| RPA2 | Figura 2.A |
| AT1G49250 | Figura 2.A |
| EMB2775 | Figura 2.A |
| MCM2 | Figura 2.A |
| AT5G08670 | Figura 2.A |
| LOG4 | Figura 2.A |
| HMG | Figura 2.A |
| AT5G59140 | Figura 2.A |
| T5M16.6 | Figura 2.A |
| DCL4 | Figura 2.A |
| RAD9 | Figura 2.A |
| POLD3 | Figura 2.A |
| ATRAD17 | Figura 2.A |
| RLP51 | Figura 2.A |
| ATVPS33 | Figura 2.A |
| mtLPD1 | Figura 2.A |
| mtLPD2 | Figura 2.A |
| SGT1A | Figura 2.A |
| AT4G16155 | Figura 2.A |
| AT3G46710 | Figura 2.A |
| LPD1 | Figura 2.A |
| SGT1B | Figura 2.A |
| PMT5 | Figura 2.A |
| RPT4A | Figura 2.A |
| HBT | Figura 2.A |
| AKRP | Figura 2.A |
| RPN8A | Figura 2.A |
| RPT5A | Figura 2.A |
| AT4G24820 | Figura 2.A |
| AT2G03430 | Figura 2.A |
| APC4 | Figura 2.A |
| APC7 | Figura 2.A |
| CDC20.4 | Figura 2.A |
| AT1G04810 | Figura 2.A |
| PAC1 | Figura 2.A |
| UBC10 | Figura 2.A |
| UBC20 | Figura 2.A |
| FUS9 | Figura 2.A |
| PAE1 | Figura 2.A |
| AT1G53780 | Figura 2.A |
| RPN1A | Figura 2.A |
| UBC19 | Figura 2.A |
| AT2G05840.1 | Figura 2.A |
| EMB2719 | Figura 2.A |
| ATS9 | Figura 2.A |
| RPN1B | Figura 2.A |
| IDH1 | Figura 2.A |
| RPT2a | Figura 2.A |
| APC10 | Figura 2.A |
| PAG1 | Figura 2.A |
| CDC27a | Figura 2.A |
| AT1G75990 | Figura 2.A |
| AT5G20000 | Figura 2.A |
| CDC20.1 | Figura 2.A |
| AT1G09995 | Figura 2.A |
| APC8 | Figura 2.A |
| AT1G45000 | Figura 2.A |
| PBC2 | Figura 2.A |
| FZR2 | Figura 2.A |
| UBC12 | Figura 2.A |
| MEE34 | Figura 2.A |
| AT1G79890 | Figura 2.A |
| APC6 | Figura 2.A |
| UBC9 | Figura 2.A |
| AT2G04660.1 | Figura 2.A |
| AT5G45620 | Figura 2.A |
| PAF2 | Figura 2.A |
| PBC1 | Figura 2.A |
| K2K18.4 | Figura 2.A |
| PAF1 | Figura 2.A |
| PAE2 | Figura 2.A |
| ORC2 | Figura 2.A |
| UBC30 | Figura 2.A |
| AT4G08140 | Figura 2.A |
| AT5G27945 | Figura 2.A |
| RPT2b | Figura 2.A |
| AT5G23540 | Figura 2.A |
| PBB1 | Figura 2.A |
| RPN12b | Figura 2.A |
| RPT6A | Figura 2.A |
| RPN10 | Figura 2.A |
| FZR3 | Figura 2.A |
| UVI4 | Figura 2.A |
| RPT5B | Figura 2.A |
| EMB2771 | Figura 2.A |
| AT2G20050 | Figura 2.A |
| RPN5B | Figura 2.A |
| IDH-V | Figura 2.A |
| PAD2 | Figura 2.A |
| F3O9.27 | Figura 2.A |
| AT5G57950 | Figura 2.A |
| PBG1 | Figura 2.A |
| RPT1A | Figura 2.A |
| UBC29 | Figura 2.A |
| CDC20.2 | Figura 2.A |
| AT4G15165 | Figura 2.A |
| AT1G79210 | Figura 2.A |
| IDH-III | Figura 2.A |
| PBE1 | Figura 2.A |
| PAD1 | Figura 2.A |
| CDC20.5 | Figura 2.A |
| PBF1 | Figura 2.A |
| CYC1BAT | Figura 2.A |
| RPN12a | Figura 2.A |
| UBC8 | Figura 2.A |
| AT1G06590 | Figura 2.A |
| PBB2 | Figura 2.A |
| EMB506 | Figura 2.A |
| CCS52A2 | Figura 2.A |
| PA200 | Figura 2.A |
| AT3G26340 | Figura 2.A |
| CDC20.3 | Figura 2.A |
| AT4G19006 | Figura 2.A |
| AT2G32730 | Figura 2.A |
| RPT3 | Figura 2.A |
| PBA1 | Figura 2.A |
| UBC11 | Figura 2.A |
| CAT7 | Figura 2.A |
| AT2G28450 | Figura 2.A |
| ATPA | Figura 2.A |
| UPL5 | Figura 2.A |
| AT4G28230 | Figura 2.A |
| AT3G42830 | Figura 2.A |
| PIP3 | Figura 2.A |
| CKS2 | Figura 2.A |
| CYT1 | Figura 2.A |
| AT3G55590 | Figura 2.A |
| NRPD1A | Figura 2.A |
| RBX1 | Figura 2.A |
| IDH2 | Figura 2.A |
| NRPB1 | Figura 2.A |
| NRPD1B | Figura 2.A |
| IDH-IV | Figura 2.A |
| IDH-VI | Figura 2.A |
| AO | Figura 2.A |
| SDG21 | Figura 2.A |
| AT1G14810 | Figura 2.A |
| HTR12 | Figura 2.A |
| AT1G09200 | Figura 2.A |
| SUVH1 | Figura 2.A |
| AT4G32360 | Figura 2.A |
| SUVH7 | Figura 2.A |
| SDG14 | Figura 2.A |
| AT3G27360.1 | Figura 2.A |
| AT3G61723 | Figura 2.A |
| MGH3 | Figura 2.A |
| SDG29 | Figura 2.A |
| RCD1 | Figura 2.A |
| AT5G10390.1 | Figura 2.A |
| SUVR2 | Figura 2.A |
| DDB1A | Figura 2.A |
| AT5G17850 | Figura 2.A |
| TFL2 | Figura 2.A |
| AT3G19400 | Figura 2.A |
| SWN | Figura 2.A |
| AT1G09200.1 | Figura 2.A |
| AT4G40030 | Figura 2.A |
| SDG16 | Figura 2.A |
| CAX9 | Figura 2.A |
| SUVH4 | Figura 2.A |
| AT5G12910 | Figura 2.A |
| AT4G40040.1 | Figura 2.A |
| SDG11 | Figura 2.A |
| CAX11 | Figura 2.A |
| AT4G35110 | Figura 2.A |
| MSI1 | Figura 2.A |
| SUVR4 | Figura 2.A |
| MS1 | Figura 2.A |
| AT1G75600 | Figura 2.A |
| CAX7 | Figura 2.A |
| AT5G10980.1 | Figura 2.A |
| AtkdsA1 | Figura 2.A |
| SUVR5 | Figura 2.A |
| HTR11 | Figura 2.A |
| SUVH3 | Figura 2.A |
| AT5G65360.1 | Figura 2.A |
| AT3G62500 | Figura 2.A |
| NHX1 | Figura 2.A |
| AT5G28340 | Figura 2.A |
| GRIK1 | Figura 2.A |
| AT1G13370 | Figura 2.A |
| AT4G10600 | Figura 2.A |
| AT5G39790 | Figura 2.A |
| SUVR1 | Figura 2.A |
| AT1G72880 | Figura 2.A |
| AT2G28060 | Figura 2.A |
| IMPA-2 | Figura 2.A |
| CCX4 | Figura 2.A |
| ATXR5 | Figura 2.A |
| DDB1B | Figura 2.A |
| AT5G52450 | Figura 2.A |
| SUVH2 | Figura 2.A |
| SDG20 | Figura 2.A |
| NHX5 | Figura 2.A |
| RDR1 | Figura 2.A |
| AT1G77460 | Figura 2.A |
| AT3G52105 | Figura 2.A |
| SAHH2 | Figura 2.A |
| SUVH5 | Figura 2.A |
| NHX2 | Figura 2.A |
| SCR | Figura 2.A |
| EIF2 | Figura 2.A |
| SUVH6 | Figura 2.A |
| NHX4 | Figura 2.A |
| ASHH4 | Figura 2.A |
| AT5G24490 | Figura 2.A |
| AT2G19920 | Figura 2.A |
| SDG25 | Figura 2.A |
| CMT3 | Figura 2.A |
| AT4G36550 | Figura 2.A |
| TRX3 | Figura 2.A |
| SDG2 | Figura 2.A |
| HY5 | Figura 2.A |
| LCBK2 | Figura 2.A |
| HYH | Figura 2.A |
| BRIZ2 | Figura 2.A |
| EMF2 | Figura 2.A |
| AT3G48550 | Figura 2.A |
| IMPA-6 | Figura 2.A |
| AT1G73920 | Figura 2.A |
| SOS1 | Figura 2.A |
| RDR2 | Figura 2.A |
| RDR6 | Figura 2.A |
| AT2G19930 | Figura 2.A |
| ATKDSA2 | Figura 2.A |
| AT3G52100 | Figura 2.A |
| CKS | Figura 2.A |
| AT4G14930 | Figura 2.A |
| RING1 | Figura 2.A |
| RR14 | Figura 2.A |
| NHX3 | Figura 2.A |
| AT2G19910 | Figura 2.A |
| SDG4 | Figura 2.A |
| RIF10 | Figura 2.A |
| PUB8 | Figura 2.A |
| ATARCA | Figura 2.A |
| LOS2 | Figura 2.A |
| MFP2 | Figura 2.A |
| AT5G67340 | Figura 2.A |
| AT1G18270 | Figura 2.A |
| LCBK1 | Figura 2.A |
| AT2G45720 | Figura 2.A |
| AT4G13720 | Figura 2.A |
| CAX3 | Figura 2.A |
| GRXS17 | Figura 2.A |
| AT3G21670 | Figura 2.A |
| AT1G15810 | Figura 2.A |
| UPP | Figura 2.A |
| AT1G18460 | Figura 2.A |
| AT3G26560 | Figura 2.A |
| GRIK2 | Figura 2.A |
| AT5G14580 | Figura 2.A |
| At2g15230 | Figura 2.A |
| CAX6 | Figura 2.A |
| GGP3 | Figura 2.A |
| PUB43 | Figura 2.A |
| AT4G01790 | Figura 2.A |
| AT1G01830 | Figura 2.A |
| SPHK2 | Figura 2.A |
| ACD5 | Figura 2.A |
| CHX20 | Figura 2.A |
| AT2G05810 | Figura 2.A |
| AT1G15165 | Figura 2.A |
| emb1997 | Figura 2.A |
| AT5G50900 | Figura 2.A |
| AT1G79470 | Figura 2.A |
| AT2G23970 | Figura 2.A |
| AT5G07840 | Figura 2.A |
| AT2G23960 | Figura 2.A |
| AT3G26590 | Figura 2.A |
| AT4G12710 | Figura 2.A |
| UBQ3 | Figura 2.A |
| AT4G11160 | Figura 2.A |
| AT4G36140 | Figura 2.A |
| GGP1 | Figura 2.A |
| CAX2 | Figura 2.A |
| FAB1A | Figura 2.A |
| LpxK | Figura 2.A |
| AT7SL-1 | Figura 2.A |
| KEA2 | Figura 2.A |
| ASB1 | Figura 2.A |
| UBQ4 | Figura 2.A |
| AT5G23330 | Figura 2.A |
| MCCA | Figura 2.A |
| ARIA | Figura 2.A |
| SPHK1 | Figura 2.A |
| NHX8 | Figura 2.A |
| AT1G08315 | Figura 2.A |
| AT4G34730 | Figura 2.A |
| AT4G03370 | Figura 2.A |
| GLN1.3 | Figura 2.A |
| ASE2 | Figura 2.A |
| CAX5 | Figura 2.A |
| AT2G27430 | Figura 2.A |
| FAH2 | Figura 2.A |
| UBQ7 | Figura 2.A |
| PUB12 | Figura 2.A |
| PUB15 | Figura 2.A |
| AT5G16090 | Figura 2.A |
| RAD23C | Figura 2.A |
| UBQ11 | Figura 2.A |
| AT-HF | Figura 2.A |
| AT2G17190 | Figura 2.A |
| FUM2 | Figura 2.A |
| AT4G03360 | Figura 2.A |
| AT3G23145 | Figura 2.A |
| UBQ13 | Figura 2.A |
| MPL1 | Figura 2.A |
| KIN11 | Figura 2.A |
| AT5G14510 | Figura 2.A |
| AT3G03440 | Figura 2.A |
| FAB1B | Figura 2.A |
| AT4G05270 | Figura 2.A |
| BRIZ1 | Figura 2.A |
| AT1G68940 | Figura 2.A |
| GDH1 | Figura 2.A |
| AT4G05240 | Figura 2.A |
| AT3G06455 | Figura 2.A |
| BCCP2 | Figura 2.A |
| CAC3 | Figura 2.A |
| KEA1 | Figura 2.A |
| AT1G44120 | Figura 2.A |
| AT2G26780 | Figura 2.A |
| AT4G05250 | Figura 2.A |
| CAX1 | Figura 2.A |
| AIM1 | Figura 2.A |
| AT1G16350 | Figura 2.A |
| AT5G09340 | Figura 2.A |
| GSR2 | Figura 2.A |
| UBQ9 | Figura 2.A |
| OVA6 | Figura 2.A |
| AT4G05230 | Figura 2.A |
| NHX6 | Figura 2.A |
| RAD23B | Figura 2.A |
| AT4G30540 | Figura 2.A |
| AT5G57890 | Figura 2.A |
| ASE3 | Figura 2.A |
| EVE1 | Figura 2.A |
| GAD4 | Figura 2.A |
| ATFAH1 | Figura 2.A |
| AT4G05260 | Figura 2.A |
| At3g13445 | Figura 2.A |
| AT3G09360 | Figura 2.A |
| ALDH12A1 | Figura 2.A |
| AT4G05310 | Figura 2.A |
| AT4G16490 | Figura 2.A |
| SETH3 | Figura 2.A |
| rps15 | Figura 2.A |
| AT3G58140 | Figura 2.A |
| AT5G42220 | Figura 2.A |
| KEA3 | Figura 2.A |
| FUG1 | Figura 2.A |
| AT1G80620 | Figura 2.A |
| PUR2 | Figura 2.A |
| UBQ6 | Figura 2.A |
| ASE1 | Figura 2.A |
| CAX4 | Figura 2.A |
| AT1G61350 | Figura 2.A |
| AT5G19720 | Figura 2.A |
| AT4G26870 | Figura 2.A |
| AAE18 | Figura 2.A |
| ATKRS-1 | Figura 2.A |
| AT2G32350 | Figura 2.A |
| AT5G57120 | Figura 2.A |
| AT2G38905 | Figura 2.A |
| AT3G23310 | Figura 2.A |
| AT5G09890 | Figura 2.A |
| AT2G19400 | Figura 2.A |
| UBQ14 | Figura 2.A |
| UBQ10 | Figura 2.A |
| ACC1 | Figura 2.A |
| GAD | Figura 2.A |
| AT1G03920 | Figura 2.A |
| GAD3 | Figura 2.A |
| AT3G49640 | Figura 2.A |
| AT2G20470 | Figura 2.A |
| RAD23D | Figura 2.A |
| AT4G14350 | Figura 2.A |
| AT1G30640 | Figura 2.A |
| AT3G30805 | Figura 2.A |
| PEX4 | Figura 2.A |
| AT5G26710 | Figura 2.A |
| AT1G09620 | Figura 2.A |
| UBC37 | Figura 2.A |
| ERS | Figura 2.A |
| GDH2 | Figura 2.A |
| GS2 | Figura 2.A |
| HAF2 | Figura 2.A |
| UBC35 | Figura 2.A |
| GLU1 | Figura 2.A |
| AT4G31180 | Figura 2.A |
| UBQ8 | Figura 2.A |
| GDH3 | Figura 2.A |
| AT3G49645 | Figura 2.A |
| TAF5 | Figura 2.A |
| ACC2 | Figura 2.A |
| AT2G04540 | Figura 2.A |
| OVA2 | Figura 2.A |
| HSP91 | Figura 2.A |
| UBP12 | Figura 2.A |
| FAB1D | Figura 2.A |
| CAC1 | Figura 2.A |
| AT2G32790 | Figura 2.A |
| AT1G51720 | Figura 2.A |
| RANGAP2 | Figura 2.A |
| AT3G62120 | Figura 2.A |
| ARP2 | Figura 2.A |
| AT3G59980 | Figura 2.A |
| UBC36 | Figura 2.A |
| AT4G35540 | Figura 2.A |
| HAF01 | Figura 2.A |
| AT1G11660 | Figura 2.A |
| NodGS | Figura 2.A |
| FAB1C | Figura 2.A |
| AT5G47720 | Figura 2.A |
| HDA6 | Figura 2.A |
| RANGAP1 | Figura 2.A |
| GAD5 | Figura 2.A |
| UBP26 | Figura 2.A |
| AAE17 | Figura 2.A |
| GLU2 | Figura 2.A |
| AKINBETA1 | Figura 2.A |
| D2HGDH | Figura 2.A |
| ACS | Figura 2.A |
| UBC31 | Figura 2.A |
| AT4G02950 | Figura 2.A |
| AT5G03495 | Figura 2.A |
| GLT1 | Figura 2.A |
| AT2G07698 | Figura 2.A |
| CAC2 | Figura 2.A |
| AT1G44180 | Figura 2.A |
| HCS1 | Figura 2.A |
| AT5G03480 | Figura 2.A |
| TBP2 | Figura 2.A |
| UBC27 | Figura 2.A |
| cICDH | Figura 2.A |
| AQI | Figura 2.A |
| AT4G10320 | Figura 2.A |
| UBC28 | Figura 2.A |
| PSD | Figura 2.A |
| AT1G44820 | Figura 2.A |
| UBP13 | Figura 2.A |
| AT4G39280 | Figura 2.A |
| AT1G15130 | Figura 2.A |
| AT5G10880 | Figura 2.A |
| ATP1 | Figura 2.A |
| ACCD | Figura 2.A |
| AT2G45100 | Figura 2.A |
| SNF4 | Figura 2.A |
| GAD2 | Figura 2.A |
| OVA9 | Figura 2.A |
| TPR7 | Figura 2.A |
| AT4G13780 | Figura 2.A |
| AT5G14590 | Figura 2.A |
| AT4G23460 | Figura 2.A |
| ICDH | Figura 2.A |
| KASI | Figura 2.A |
| PBRP | Figura 2.A |
| ZR3 | Figura 2.A |
| GLN1-1 | Figura 2.A |
| OVA5 | Figura 2.A |
| cpHsc70-2 | Figura 2.A |
| AT1G69800 | Figura 2.A |
| CDPK6 | Figura 2.A |
| TIM44-2 | Figura 2.A |
| TAF2 | Figura 2.A |
| AT3G53680 | Figura 2.A |
| emb1027 | Figura 2.A |
| TIM17-1 | Figura 2.A |
| RBR1 | Figura 2.A |
| MEE65 | Figura 2.A |
| AT4G16360 | Figura 2.A |
| T21L8.140 | Figura 2.A |
| AT4G32208 | Figura 2.A |
| BIP1 | Figura 2.A |
| PYRB | Figura 2.A |
| BIP3 | Figura 2.A |
| GCT | Figura 2.A |
| KCO1 | Figura 2.A |
| AT5G60335 | Figura 2.A |
| AT1G66530 | Figura 2.A |
| KIN10 | Figura 2.A |
| AT3G09700 | Figura 2.A |
| EMB3147 | Figura 2.A |
| SnRK1.3 | Figura 2.A |
| AT2G40660 | Figura 2.A |
| ATERDJ3A | Figura 2.A |
| AT4G09000.2 | Figura 2.A |
| AT2G17880 | Figura 2.A |
| FAB1 | Figura 2.A |
| AT1G62640 | Figura 2.A |
| J3 | Figura 2.A |
| HSP70T-2 | Figura 2.A |
| ECH2 | Figura 2.A |
| TIM44-1 | Figura 2.A |
| cpHsc70-1 | Figura 2.A |
| GLY1 | Figura 2.A |
| ACAT2 | Figura 2.A |
| GPDHp | Figura 2.A |
| MED6 | Figura 2.A |
| HCS2 | Figura 2.A |
| AT2G21510 | Figura 2.A |
| AT2G35795 | Figura 2.A |
| AT3G07690 | Figura 2.A |
| GPDHC1 | Figura 2.A |
| F12B17_200 | Figura 2.A |
| NLM1 | Figura 2.A |
| ATERDJ3B | Figura 2.A |
| J2 | Figura 2.A |
| sks4 | Figura 2.A |
| AT5G46630 | Figura 2.A |
| NUB1 | Figura 2.A |
| AT3G21060 | Figura 2.A |
| TIM50 | Figura 2.A |
| MTHSC70-2 | Figura 2.A |
| TIM17-3 | Figura 2.A |
| AT1G72070 | Figura 2.A |
| mtHsc70-1 | Figura 2.A |
| TIM17-2 | Figura 2.A |
| AR192 | Figura 2.A |
| AT1G50670 | Figura 2.A |
| AT1G36390 | Figura 2.A |
| APM1 | Figura 2.A |
| J20 | Figura 2.A |
| AT5G62910 | Figura 2.A |
| BIP2 | Figura 2.A |
| HSP70 | Figura 2.A |
| CHIP | Figura 2.A |
| F7H19.150 | Figura 2.A |
| PAB4 | Figura 2.A |
| AT4G36960 | Figura 2.A |
| Hsp70-2 | Figura 2.A |
| EMB1241 | Figura 2.A |
| AT3G09440 | Figura 2.A |
| AT5G03030 | Figura 2.A |
| AT4G11380 | Figura 2.A |
| PAB2 | Figura 2.A |
| ORC1A | Figura 2.A |
| RAD23A | Figura 2.A |
| AT1G74870 | Figura 2.A |
| AT5G15400 | Figura 2.A |
| AT2G28540 | Figura 2.A |
| AT5G60170 | Figura 2.A |
| HUB2 | Figura 2.A |
| CDC6 | Figura 2.A |
| AT3G45630 | Figura 2.A |
| MGE1 | Figura 2.A |
| HUB1 | Figura 2.A |
| ORC1B | Figura 2.A |
| HSC70-1 | Figura 2.A |
| Hsp70b | Figura 2.A |
| AT3G48070 | Figura 2.A |
| TPP2 | Figura 2.A |
| HSP70-18 | Figura 2.A |
| AG | Figura 2.A |
| RAD4 | Figura 2.A |
| PPH | Figura 2.F |
| AT2G36260 | Figura 2.F |
| AT4G24160 | Figura 2.F |
| AT3G06690 | Figura 2.F |
| AT5G03905 | Figura 2.F |
| CYP702A5 | Figura 2.F |
| PTF1 | Figura 2.F |
| AT4G05160 | Figura 2.F |
| AT4G01920 | Figura 2.F |
| AT4G02340 | Figura 2.F |
| AT2G40095 | Figura 2.F |
| AT5G21950 | Figura 2.F |
| CYP77A5P | Figura 2.F |
| CYP96A9 | Figura 2.F |
| BAS | Figura 2.F |
| ACX5 | Figura 2.F |
| PHYLLO | Figura 2.F |
| BR6OX1 | Figura 2.F |
| DWF4 | Figura 2.F |
| AT4G32590 | Figura 2.F |
| CYP51G1 | Figura 2.F |
| AT2G26750 | Figura 2.F |
| AT1G15490 | Figura 2.F |
| AT5G08250 | Figura 2.F |
| SUE4 | Figura 2.F |
| AT4G36530 | Figura 2.F |
| AT2G03500 | Figura 2.F |
| AT5G09430 | Figura 2.F |
| CYP96A12 | Figura 2.F |
| AT3G52570 | Figura 2.F |
| THAS1 | Figura 2.F |
| AT4G25290 | Figura 2.F |
| BR6OX2 | Figura 2.F |
| CYP72A13 | Figura 2.F |
| AT4G36610 | Figura 2.F |
| IBR3 | Figura 2.F |
| CYP707A4 | Figura 2.F |
| CYP72A9 | Figura 2.F |
| AT5G36150.1 | Figura 2.F |
| CPD | Figura 2.F |
| PEN1 | Figura 2.F |
| ECHID | Figura 2.F |
| GATB | Figura 2.F |
| At2g26260 | Figura 2.F |
| FAAH | Figura 2.F |
| ERG28 | Figura 2.F |
| CYP716A2 | Figura 2.F |
| CPISCA | Figura 2.F |
| CYP702A8 | Figura 2.F |
| CYP724A1 | Figura 2.F |
| CYP90D1 | Figura 2.F |
| G-TMT | Figura 2.F |
| FdC1 | Figura 2.F |
| AT4G10050 | Figura 2.F |
| AT4G19010 | Figura 2.F |
| CYP704A2 | Figura 2.F |
| BARS1 | Figura 2.F |
| AT1G80280 | Figura 2.F |
| CYP86B1 | Figura 2.F |
| AT5G16340 | Figura 2.F |
| CYP86A8 | Figura 2.F |
| AT5G63380 | Figura 2.F |
| CYP704A1 | Figura 2.F |
| SQE3 | Figura 2.F |
| DWF5 | Figura 2.F |
| CYP72A7 | Figura 2.F |
| LAS1 | Figura 2.F |
| AT3G44970 | Figura 2.F |
| CYP72A10 | Figura 2.F |
| SQE6 | Figura 2.F |
| ROT3 | Figura 2.F |
| AT3G01660 | Figura 2.F |
| SMO1-1 | Figura 2.F |
| CYP96A13 | Figura 2.F |
| CAMS1 | Figura 2.F |
| CYP702A6 | Figura 2.F |
| SQP2 | Figura 2.F |
| AT2G43420 | Figura 2.F |
| CAS1 | Figura 2.F |
| PMEAMT | Figura 2.F |
| AT2G33630 | Figura 2.F |
| CYP715A1 | Figura 2.F |
| CYP94B1 | Figura 2.F |
| XPL1 | Figura 2.F |
| LUP1 | Figura 2.F |
| AT1G78480 | Figura 2.F |
| XF1 | Figura 2.F |
| FD3 | Figura 2.F |
| CYP97B3 | Figura 2.F |
| AT2G16710 | Figura 2.F |
| CYP709B1 | Figura 2.F |
| MRN1 | Figura 2.F |
| AT3G02590 | Figura 2.F |
| CYP714A2 | Figura 2.F |
| AT4G39955 | Figura 2.F |
| SQE2 | Figura 2.F |
| CYP72A8 | Figura 2.F |
| STE1 | Figura 2.F |
| AAE3 | Figura 2.F |
| FD1 | Figura 2.F |
| AT1G78500 | Figura 2.F |
| CYP96A2 | Figura 2.F |
| AAE5 | Figura 2.F |
| CYP708A2 | Figura 2.F |
| LUP5 | Figura 2.F |
| CYP72A11 | Figura 2.F |
| AT3G29255 | Figura 2.F |
| SMO2-2 | Figura 2.F |
| CYP702A3 | Figura 2.F |
| ACX4 | Figura 2.F |
| AT5G24155 | Figura 2.F |
| AT1G73600 | Figura 2.F |
| CYP86C2 | Figura 2.F |
| CYP709B2 | Figura 2.F |
| CYP86A1 | Figura 2.F |
| FK | Figura 2.F |
| CYP96A10 | Figura 2.F |
| AT5G51900 | Figura 2.F |
| SMO2-1 | Figura 2.F |
| CYP709B3 | Figura 2.F |
| CYP707A3 | Figura 2.F |
| SMT1 | Figura 2.F |
| SMT3 | Figura 2.F |
| CYP72A14 | Figura 2.F |
| CYP94B3 | Figura 2.F |
| SMO1-2 | Figura 2.F |
| BDG1 | Figura 2.F |
| MOD1 | Figura 2.F |
| T3F24.9 | Figura 2.F |
| FD4 | Figura 2.F |
| CYP716A1 | Figura 2.F |
| SQP1 | Figura 2.F |
| CYP94B2 | Figura 2.F |
| AT2G30160 | Figura 2.F |
| CYP86A2 | Figura 2.F |
| AT1G07030 | Figura 2.F |
| CYP714A1 | Figura 2.F |
| MFDX2 | Figura 2.F |
| 4CL8 | Figura 2.F |
| CYP72A15 | Figura 2.F |
| CYP96A11 | Figura 2.F |
| CYP702A2 | Figura 2.F |
| ISU2 | Figura 2.F |
| PSY | Figura 2.F |
| CYP94D2 | Figura 2.F |
| LUP2 | Figura 2.F |
| AT5G06410 | Figura 2.F |
| SMT2 | Figura 2.F |
| CYP707A1 | Figura 2.F |
| AT1G34575 | Figura 2.F |
| CKX3 | Figura 2.F |
| AT1G26420 | Figura 2.F |
| MFDX1 | Figura 2.F |
| AT1G11770 | Figura 2.F |
| AT1G26400 | Figura 2.F |
| PnsB3 | Figura 2.F |
| AT1G26410 | Figura 2.F |
| AT4G20800 | Figura 2.F |
| LUT2 | Figura 2.F |
| CYP96A4 | Figura 2.F |
| CRTISO | Figura 2.F |
| LYC | Figura 2.F |
| CKX6 | Figura 2.F |
| L73G19.80 | Figura 2.F |
| CKX7 | Figura 2.F |
| LUT1 | Figura 2.F |
| GulLO2 | Figura 2.F |
| F6N7.5 | Figura 2.F |
| NPQ1 | Figura 2.F |
| AT1G30700 | Figura 2.F |
| ABA1 | Figura 2.F |
| AT5G06580 | Figura 2.F |
| AT1G77670 | Figura 2.F |
| ISU1 | Figura 2.F |
| AT5G44390 | Figura 2.F |
| CYP97A3 | Figura 2.F |
| NFS1 | Figura 2.F |
| AT5G44440 | Figura 2.F |
| AT5G57300 | Figura 2.F |
| ZDS | Figura 2.F |
| GulLO4 | Figura 2.F |
| D27 | Figura 2.F |
| BIO1 | Figura 2.F |
| PSB27 | Figura 2.F |
| AT5G36130 | Figura 2.F |
| AT5G44410 | Figura 2.F |
| AT1G78140 | Figura 2.F |
| CCD7 | Figura 2.F |
| AT2G41040 | Figura 2.F |
| CKX5 | Figura 2.F |
| NAPRT1 | Figura 2.F |
| NAPRT2 | Figura 2.F |
| ATSEC1A | Figura 2.F |
| GulLO5 | Figura 2.F |
| AT5G44380 | Figura 2.F |
| AT2G34810 | Figura 2.F |
| AT4G20840 | Figura 2.F |
| AT1G26390 | Figura 2.F |
| FH | Figura 2.F |
| AT4G20830 | Figura 2.F |
| ISU3 | Figura 2.F |
| GulLO1 | Figura 2.F |
| GulLO3 | Figura 2.F |
| AT5G44360 | Figura 2.F |
| MEE23 | Figura 2.F |
| AT1G30740 | Figura 2.F |
| AT1G30730 | Figura 2.F |
| CKX1 | Figura 2.F |
| ETFQO | Figura 2.F |
| GulLO6 | Figura 2.F |
| AT1G30760 | Figura 2.F |
| AT5G44400 | Figura 2.F |
| AT4G20860 | Figura 2.F |
| AT1G30710 | Figura 2.F |
| GulLO7 | Figura 2.F |
| AT4G20820 | Figura 2.F |
| CKX4 | Figura 2.F |
| ETFBETA | Figura 2.F |
| CKX2 | Figura 2.F |
| AT1G26380 | Figura 2.F |
| AT1G30720 | Figura 2.F |
| ETFALPHA | Figura 2.F |
| GAI | Figura 2.F |
| CYP735A1 | Figura 2.F |
| AT3G15160 | Figura 2.F |
| WOX9 | Figura 2.F |
| AT3G48240 | Figura 2.F |
| AT5G63130 | Figura 2.F |
| ZFP7 | Figura 2.F |
| AT2G46690 | Figura 2.F |
| AT2G18360 | Figura 2.F |
| AT1G19680 | Figura 2.F |
| DWF1 | Figura 2.F |
| NTL9 | Figura 2.F |
| AT5G19850 | Figura 2.F |
| RGA1 | Figura 2.F |
| AT4G39050 | Figura 2.F |
| AT5G17780 | Figura 2.F |
| AT1G17430 | Figura 2.F |
| ACX2 | Figura 2.F |
| HYD1 | Figura 2.F |
| AT5G17720 | Figura 2.F |
| ACX1 | Figura 2.F |
| CPI1 | Figura 2.F |
| KAI2 | Figura 2.F |
| AT3G43240 | Figura 2.F |
| AT4G15955 | Figura 2.F |
| AT4G10030 | Figura 2.F |
| TCP14 | Figura 2.F |
| AT4G15960 | Figura 2.F |
| AT1G78210 | Figura 2.F |
| AT4G12830 | Figura 2.F |
| ACX6 | Figura 2.F |
| ACX3 | Figura 2.F |
| NAC089 | Figura 2.F |
| AT3G03990 | Figura 2.F |
| AT5G39220 | Figura 2.F |
| AT5G22355 | Figura 2.F |
| AT1G52510 | Figura 2.F |
| AT4G24140 | Figura 2.F |
| AT3G61540 | Figura 2.F |
| AT1G72620 | Figura 2.F |
| AT1G52750 | Figura 2.F |
| AT1G27300 | Figura 2.F |
| AT4G33180 | Figura 2.F |
| AT1G13820 | Figura 2.F |
| NRPC2 | Figura 2.D |
| NDPK3 | Figura 2.D |
| RPB5C | Figura 2.D |
| AT1G53690 | Figura 2.D |
| CML11 | Figura 2.D |
| AT3G52270 | Figura 2.D |
| AT4G07950 | Figura 2.D |
| CAM3 | Figura 2.D |
| NRPB8A | Figura 2.D |
| CAM6 | Figura 2.D |
| LIF2 | Figura 2.D |
| VIP3 | Figura 2.D |
| AT3G15710 | Figura 2.D |
| AT2G25100 | Figura 2.D |
| AT5G50120 | Figura 2.D |
| Plsp2A | Figura 2.D |
| AT3G51930 | Figura 2.D |
| TON1B | Figura 2.D |
| AT1G24240 | Figura 2.D |
| CAM2 | Figura 2.D |
| PLSP1 | Figura 2.D |
| RHL1 | Figura 2.D |
| AT4G11630 | Figura 2.D |
| AT3G18950 | Figura 2.D |
| AT4G34380 | Figura 2.D |
| AT2G27480 | Figura 2.D |
| TPP | Figura 2.D |
| ADC2 | Figura 2.D |
| TRIP-1 | Figura 2.D |
| AT2G41410 | Figura 2.D |
| AT3G49000 | Figura 2.D |
| MSS3 | Figura 2.D |
| AT1G06200 | Figura 2.D |
| AGD11 | Figura 2.D |
| AT1G18530 | Figura 2.D |
| AT1G24620 | Figura 2.D |
| CAM4 | Figura 2.D |
| ARGAH1 | Figura 2.D |
| ADC1 | Figura 2.D |
| CAM7 | Figura 2.D |
| AT1G29960 | Figura 2.D |
| AT1G53530 | Figura 2.D |
| AT3G03400 | Figura 2.D |
| AT5G13240 | Figura 2.D |
| AT1G52600 | Figura 2.D |
| CML42 | Figura 2.D |
| AT5G11750 | Figura 2.D |
| AT1G23465 | Figura 2.D |
| AT3G28956 | Figura 2.D |
| AT5G62950 | Figura 2.D |
| RAD51C | Figura 2.D |
| AT5G23710 | Figura 2.D |
| AT1G06790 | Figura 2.D |
| AT5G04170 | Figura 2.D |
| AT1G32250 | Figura 2.D |
| CAM9 | Figura 2.D |
| CML30 | Figura 2.D |
| AT1G21550 | Figura 2.D |
| CML23 | Figura 2.D |
| AT3G03430 | Figura 2.D |
| AT5G49530 | Figura 2.D |
| AT2G41090 | Figura 2.D |
| AT1G18210 | Figura 2.D |
| TON1A | Figura 2.D |
| CML38 | Figura 2.D |
| AT4G03290 | Figura 2.D |
| AT4G26470 | Figura 2.D |
| AT3G59440 | Figura 2.D |
| AT3G10190 | Figura 2.D |
| AT1G73630 | Figura 2.D |
| DMC1 | Figura 2.D |
| AT3G24110 | Figura 2.D |
| AT3G47480 | Figura 2.D |
| CML43 | Figura 2.D |
| AT4G24830 | Figura 2.D |
| AT2G36180 | Figura 2.D |
| AT1G76640 | Figura 2.D |
| AT3G10300 | Figura 2.D |
| AT5G17470 | Figura 2.D |
| AT1G12310 | Figura 2.D |
| AT3G03410 | Figura 2.D |
| APRR2 | Figura 2.D |
| AT3G25600 | Figura 2.D |
| PC1 | Figura 2.D |
| UNE14 | Figura 2.D |
| CAM8 | Figura 2.D |
| RHS2 | Figura 2.D |
| CML41 | Figura 2.D |
| AT3G03000 | Figura 2.D |
| TCH2 | Figura 2.D |
| AT1G62820 | Figura 2.D |
| TCH3 | Figura 2.D |
| RHL2 | Figura 2.D |
| SAP130a | Figura 2.D |
| AT5G39770 | Figura 2.D |
| AT3G11960 | Figura 2.D |
| MUS81 | Figura 2.D |
| XRCC3 | Figura 2.D |
| MAC3A | Figura 2.D |
| MAC3B | Figura 2.D |
| AT1G10580 | Figura 2.D |
| AT5G48700 | Figura 2.D |
| AT5G28740 | Figura 2.D |
| SNRNP-G | Figura 2.D |
| AT5G19490 | Figura 2.D |
| NF-YB12 | Figura 2.D |
| NRPC1 | Figura 2.D |
| AT5G10350 | Figura 2.D |
| MEE5 | Figura 2.D |
| AT4G30680 | Figura 2.D |
| RPOC2 | Figura 2.D |
| AT5G55856 | Figura 2.D |
| AT5G25230 | Figura 2.D |
| EMB2770 | Figura 2.D |
| SUMO2 | Figura 2.D |
| AT4G26650 | Figura 2.D |
| AT3G13224 | Figura 2.D |
| PTB3 | Figura 2.D |
| RCK | Figura 2.D |
| AT3G52120 | Figura 2.D |
| SAP130b | Figura 2.D |
| SUMO1 | Figura 2.D |
| AT1G20580 | Figura 2.D |
| AT2G05720 | Figura 2.D |
| AT5G61140 | Figura 2.D |
| SR34 | Figura 2.D |
| SMG7 | Figura 2.D |
| AT5G08565 | Figura 2.D |
| RS31 | Figura 2.D |
| AT2G29210 | Figura 2.D |
| AT5G48710 | Figura 2.D |
| LIS | Figura 2.D |
| CLPS3 | Figura 2.D |
| AT1G66500 | Figura 2.D |
| AT5G12190 | Figura 2.D |
| SR30 | Figura 2.D |
| CPSF73-I | Figura 2.D |
| AT5G54910 | Figura 2.D |
| AT2G33410 | Figura 2.D |
| SCL30 | Figura 2.D |
| AtRZ-1c | Figura 2.D |
| AT1G28180 | Figura 2.D |
| SUMO3 | Figura 2.D |
| TAF15 | Figura 2.D |
| YLS8 | Figura 2.D |
| AT1G07170 | Figura 2.D |
| AT4G25550 | Figura 2.D |
| emb1579 | Figura 2.D |
| NRPA1 | Figura 2.D |
| AT1G14650 | Figura 2.D |
| SUMO5 | Figura 2.D |
| AT1G14640 | Figura 2.D |
| RGD3 | Figura 2.D |
| AT2G33730 | Figura 2.D |
| SPT42 | Figura 2.D |
| AT4G38780 | Figura 2.D |
| NF-YB13 | Figura 2.D |
| AT2G43770 | Figura 2.D |
| NF-YC11 | Figura 2.D |
| AT1G17640 | Figura 2.D |
| AT5G44500 | Figura 2.D |
| AT5G40490 | Figura 2.D |
| RPOC1 | Figura 2.D |
| AT3G07590 | Figura 2.D |
| F18B13.15 | Figura 2.D |
| AT5G43620 | Figura 2.D |
| AT5G64270 | Figura 2.D |
| smB | Figura 2.D |
| ABH1 | Figura 2.D |
| U1-70K | Figura 2.D |
| TTN8 | Figura 2.D |
| AT5G65260 | Figura 2.D |
| ATO | Figura 2.D |
| CFIM-25 | Figura 2.D |
| AT2G36480 | Figura 2.D |
| LSM2 | Figura 2.D |
| ATU2AF35A | Figura 2.D |
| PCFS4 | Figura 2.D |
| WRKY7 | Figura 2.D |
| AT3G52660 | Figura 2.D |
| CAM5 | Figura 2.D |
| PTB2 | Figura 2.D |
| AT3G44785 | Figura 2.D |
| AT2G18740 | Figura 2.D |
| AT5G55550 | Figura 2.D |
| RSZ22a | Figura 2.D |
| U2AF35B | Figura 2.D |
| AT3G23325 | Figura 2.D |
| U1A | Figura 2.D |
| AT2G44710 | Figura 2.D |
| CSP3 | Figura 2.D |
| AT5G47620 | Figura 2.D |
| AT5G28390 | Figura 2.D |
| AT5G22080 | Figura 2.D |
| AT2G32600 | Figura 2.D |
| AT1G06960 | Figura 2.D |
| SUA | Figura 2.D |
| AT5G09390 | Figura 2.D |
| AT5G60940 | Figura 2.D |
| CSDP1 | Figura 2.D |
| BTR1L | Figura 2.D |
| AT4G21660 | Figura 2.D |
| SC35 | Figura 2.D |
| AT4G02840 | Figura 2.D |
| AT4G14342 | Figura 2.D |
| EMB3011 | Figura 2.D |
| CSTF64 | Figura 2.D |
| SR34b | Figura 2.D |
| AT3G11500 | Figura 2.D |
| AT4G30330 | Figura 2.D |
| SmD3 | Figura 2.D |
| CPSF100 | Figura 2.D |
| PABN1 | Figura 2.D |
| SR45 | Figura 2.D |
| CSTF77 | Figura 2.D |
| AT4G14300 | Figura 2.D |
| NRPB7 | Figura 2.D |
| AT5G11340 | Figura 2.D |
| CPSF160 | Figura 2.D |
| AT2G25830 | Figura 2.D |
| RSZ21 | Figura 2.D |
| AT1G01350 | Figura 2.D |
| AT2G14870 | Figura 2.D |
| SMC6A | Figura 2.D |
| AT3G07810 | Figura 2.D |
| RSZ22 | Figura 2.D |
| AT3G45180 | Figura 2.D |
| SR34a | Figura 2.D |
| AT1G56290 | Figura 2.D |
| CBP20 | Figura 2.D |
| RUXF | Figura 2.D |
| CLPS5 | Figura 2.D |
| RBP1 | Figura 2.D |
| GRP2 | Figura 2.D |
| AT2G47640 | Figura 2.D |
| LSM8 | Figura 2.D |
| CDT1A | Figura 2.D |
| CDT1B | Figura 2.D |
| NOA1 | Figura 2.D |
| PRP39 | Figura 2.D |
| RPOB | Figura 2.D |
| MIM | Figura 2.D |
| NRPB11 | Figura 2.D |
| AT3G47120 | Figura 2.D |
| RAP74 | Figura 2.D |
| EMB2765 | Figura 2.D |
| UBL5 | Figura 2.D |
| MAGO | Figura 2.D |
| AT3G05070 | Figura 2.D |
| AT5G17440 | Figura 2.D |
| NRPD2B | Figura 2.D |
| AT1G80930 | Figura 2.D |
| NRPB3 | Figura 2.D |
| MOS4 | Figura 2.D |
| NRPD2A | Figura 2.D |
| emb1644 | Figura 2.D |
| NRPB9B | Figura 2.D |
| SMP2 | Figura 2.D |
| NRPB2 | Figura 2.D |
| AT5G06420 | Figura 2.D |
| NRPB9A | Figura 2.D |
| LSM6B | Figura 2.D |
| NRPE3B | Figura 2.D |
| CAK4 | Figura 2.D |
| AT4G33060 | Figura 2.D |
| AT4G01023 | Figura 2.D |
| AT2G14285.1 | Figura 2.D |
| NRPB4 | Figura 2.D |
| AT3G02710 | Figura 2.D |
| SGR9 | Figura 2.D |
| EMB2769 | Figura 2.D |
| AT2G33435 | Figura 2.D |
| AT2G29430 | Figura 2.D |
| AT1G66510 | Figura 2.D |
| MAC5A | Figura 2.D |
| LSM3A | Figura 2.D |
| LSM6A | Figura 2.D |
| DOT2 | Figura 2.D |
| MAC5B | Figura 2.D |
| MAC5C | Figura 2.D |
| CML37 | Figura 2.D |
| AT5G50970 | Figura 2.D |
| THO2 | Figura 2.D |
| AT2G47250 | Figura 2.D |
| EMB2816 | Figura 2.D |
| AT3G62310 | Figura 2.D |
| AT1G17130 | Figura 2.D |
| AT2G42330 | Figura 2.D |
| AT5G26610 | Figura 2.D |
| AT5G51410 | Figura 2.D |
| AT4G16200 | Figura 2.D |
| STIPL1 | Figura 2.D |
| AT5G58790 | Figura 2.D |
| AT5G14900 | Figura 2.D |
| AT2G43370 | Figura 2.D |
| AT5G12280 | Figura 2.D |
| AT1G26370 | Figura 2.D |
| At1g65660 | Figura 2.D |
| ESP3 | Figura 2.D |
| BGAL14 | Figura 2.D |
| AT3G16650 | Figura 2.D |
| AT3G43250 | Figura 2.D |
| AT5G19920 | Figura 2.D |
| AT2G44200 | Figura 2.D |
| AT1G28060 | Figura 2.D |
| AT1G31870 | Figura 2.D |
| AT5G41770 | Figura 2.D |
| CDC5 | Figura 2.D |
| AT5G04210 | Figura 2.D |
| AT1G11520 | Figura 2.D |
| AT3G56790 | Figura 2.D |
| U2A | Figura 2.D |
| AT1G60200 | Figura 2.D |
| AT2G32050 | Figura 2.D |
| AT3G51110 | Figura 2.D |
| AT1G52325 | Figura 2.D |
| AT4G18465 | Figura 2.D |
| LP1 | Figura 2.D |
| AT4G08580 | Figura 2.D |
| CAM1 | Figura 2.D |
| AT2G16860 | Figura 2.D |
| SAD1 | Figura 2.D |
| AT3G13210 | Figura 2.D |
| LSM3B | Figura 2.D |
| AT3G49601 | Figura 2.D |
| AT3G18790 | Figura 2.D |
| AT3G27600 | Figura 2.D |
| emb1220 | Figura 2.D |
| AT5G17900 | Figura 2.D |
| AT3G09850 | Figura 2.D |
| AT2G47640.1 | Figura 2.D |
| UNE6 | Figura 2.D |
| AT4G16680 | Figura 2.D |
| AT5G45990 | Figura 2.D |
| MEE29 | Figura 2.D |
| AT1G70400 | Figura 2.D |
| AT4G17830 | Figura 2.D |
| AT5G06520 | Figura 2.D |
| OTC | Figura 2.D |
| AT3G55930 | Figura 2.D |
| AT3G49130 | Figura 2.D |
| SKIP | Figura 2.D |
| CASP | Figura 2.D |
| AT5G06890 | Figura 2.D |
| ATRPAC42 | Figura 2.D |
| AT1G27900 | Figura 2.D |
| OPT3 | Figura 2.D |
| NRPA2 | Figura 2.D |
| PTB1 | Figura 2.D |
| AT1G01210 | Figura 2.D |
| RPAC43 | Figura 2.D |
| AT1G75510 | Figura 2.D |
| NRPB8B | Figura 2.D |
| AT3G08980 | Figura 2.D |
| NRPB10 | Figura 2.D |
| AT1G61700 | Figura 2.D |
| NRPB5 | Figura 2.D |
| SEC6 | Figura 2.I |
| RABE1c | Figura 2.I |
| SEC3B | Figura 2.I |
| CAK1AT | Figura 2.I |
| CDKB2;1 | Figura 2.I |
| EXO84B | Figura 2.I |
| RABE1e | Figura 2.I |
| SEC3A | Figura 2.I |
| EXO70B1 | Figura 2.I |
| EXO70E2 | Figura 2.I |
| RAB8 | Figura 2.I |
| RAB1C | Figura 2.I |
| STN7 | Figura 2.I |
| AT1G10180 | Figura 2.I |
| NTMC2T6.2 | Figura 2.I |
| AT5G59840 | Figura 2.I |
| EXO70A1 | Figura 2.I |
| AT3G60950 | Figura 2.I |
| EXO70H1 | Figura 2.I |
| AT3G53590 | Figura 2.I |
| SEC15B | Figura 2.I |
| CDKB1;1 | Figura 2.I |
| EXO70H6 | Figura 2.I |
| SEC8 | Figura 2.I |
| SYTB | Figura 2.I |
| RAB1A | Figura 2.I |
| EXO70H7 | Figura 2.I |
| EXO70H8 | Figura 2.I |
| RAB8C | Figura 2.I |
| EXO70B2 | Figura 2.I |
| SYP124 | Figura 2.I |
| SEC1A | Figura 2.I |
| EXO70A3 | Figura 2.I |
| EXO70D2 | Figura 2.I |
| AT2G21010 | Figura 2.I |
| SYP131 | Figura 2.I |
| MEMB11 | Figura 2.I |
| EXO70A2 | Figura 2.I |
| VPS9A | Figura 2.I |
| MEMB12 | Figura 2.I |
| EXO70H3 | Figura 2.I |
| AT4 | Figura 2.I |
| AT5G52220 | Figura 2.I |
| EXO70G2 | Figura 2.I |
| SYTA | Figura 2.I |
| AT3G61030 | Figura 2.I |
| PRF4 | Figura 2.I |
| RA-5 | Figura 2.I |
| SYP111 | Figura 2.I |
| EXO70G1 | Figura 2.I |
| NTMC2T6.1 | Figura 2.I |
| ATSYTF | Figura 2.I |
| SYP122 | Figura 2.I |
| CDKB2;2 | Figura 2.I |
| AT5G66230 | Figura 2.I |
| EXO70F1 | Figura 2.I |
| EXO70C1 | Figura 2.I |
| EXO70C2 | Figura 2.I |
| SYP121 | Figura 2.I |
| AT3G10330 | Figura 2.I |
| TAF13 | Figura 2.I |
| CYCB1;4 | Figura 2.I |
| NTMC2T5.2 | Figura 2.I |
| HIK | Figura 2.I |
| STN8 | Figura 2.I |
| ENODL14 | Figura 2.I |
| SYTD | Figura 2.I |
| SYTC | Figura 2.I |
| ATFP8 | Figura 2.I |
| SYP132 | Figura 2.I |
| AT5G45700 | Figura 2.I |
| SYP125 | Figura 2.I |
| AT5G16250 | Figura 2.I |
| PFN2 | Figura 2.I |
| SYP123 | Figura 2.I |
| AT3G03130 | Figura 2.I |
| PRF1 | Figura 2.I |
| AT3G51280 | Figura 2.I |
| VPS54 | Figura 2.I |
| AT2G42110 | Figura 2.I |
| NTMC2T5.1 | Figura 2.I |
| AUR1 | Figura 2.I |
| NTMC2T4 | Figura 2.I |
| PRF3 | Figura 2.I |
| ORG1 | Figura 2.I |
| CYCA1;1 | Figura 2.I |
| ATK5 | Figura 2.I |
| AT2G21040 | Figura 2.I |
| PRF5 | Figura 2.I |
| ROPGEF6 | Figura 2.I |
| AT4G02800 | Figura 2.I |
| AT4G15830 | Figura 2.I |
| 3xHMG-box2 | Figura 2.I |
| ENODL15 | Figura 2.I |
| CSLD5 | Figura 2.I |
| AT3G02640 | Figura 2.I |
| AT3G02120 | Figura 2.I |
| AT5G36710 | Figura 2.I |
| EMB2754 | Figura 2.I |
| ATSYP24 | Figura 2.I |
| VCL1 | Figura 2.I |
| AT3G17020 | Figura 2.I |
| pBRP2 | Figura 2.I |
| ISA1 | Figura 2.I |
| AT3G57370 | Figura 2.I |
| TAFII59 | Figura 2.I |
| AT5G02420 | Figura 2.I |
| CYCH;1 | Figura 2.I |
| AT1G07500 | Figura 2.I |
| GCP2 | Figura 2.I |
| CTF7 | Figura 2.I |
| CDKD1;1 | Figura 2.I |
| AT2G07687.1 | Figura 2.I |
| AT5G17410 | Figura 2.I |
| AT4G14310 | Figura 2.I |
| MAP65-2 | Figura 2.I |
| PLE | Figura 2.I |
| PETA | Figura 2.I |
| TAF6B | Figura 2.I |
| TAF4B | Figura 2.I |
| RBP45A | Figura 2.I |
| DAN1 | Figura 2.I |
| KRP4 | Figura 2.I |
| CYCD3;2 | Figura 2.I |
| ICK6 | Figura 2.I |
| PETB | Figura 2.I |
| AT2G01090 | Figura 2.I |
| PETG | Figura 2.I |
| cob | Figura 2.I |
| ICK1 | Figura 2.I |
| AT5G12410 | Figura 2.I |
| EB1C | Figura 2.I |
| AT3G15640 | Figura 2.I |
| AT2G07727.1 | Figura 2.I |
| COX1 | Figura 2.I |
| HAG4 | Figura 2.I |
| AT1G51980 | Figura 2.I |
| CDC2 | Figura 2.I |
| CYCD6;1 | Figura 2.I |
| AT2G17930 | Figura 2.I |
| CYCA3;3 | Figura 2.I |
| SWP | Figura 2.I |
| AT1G10690 | Figura 2.I |
| BSH | Figura 2.I |
| TAFII21 | Figura 2.I |
| EB1B | Figura 2.I |
| AT3G52730 | Figura 2.I |
| CYCD1;1 | Figura 2.I |
| AT3G27240 | Figura 2.I |
| EER4 | Figura 2.I |
| AT4G24440 | Figura 2.I |
| AT5G13430 | Figura 2.I |
| TAF14 | Figura 2.I |
| AT4G27900 | Figura 2.I |
| ADA2A | Figura 2.I |
| AT4G36080 | Figura 2.I |
| AT4G38900 | Figura 2.I |
| AT4G10680 | Figura 2.I |
| AT4G37830 | Figura 2.I |
| EB1a | Figura 2.I |
| AT1G15120 | Figura 2.I |
| TAF11 | Figura 2.I |
| HAG5 | Figura 2.I |
| CYCD4;2 | Figura 2.I |
| MPPBETA | Figura 2.I |
| ICK5 | Figura 2.I |
| CYCD4;1 | Figura 2.I |
| CYCD2;1 | Figura 2.I |
| TAF4 | Figura 2.I |
| ADA2B | Figura 2.I |
| TAFII15 | Figura 2.I |
| UBP22 | Figura 2.I |
| COX6B | Figura 2.I |
| GAS41 | Figura 2.I |
| TUBG1 | Figura 2.I |
| TFIIB | Figura 2.I |
| COX3 | Figura 2.I |
| AT4G32470 | Figura 2.I |
| TAF7 | Figura 2.I |
| AT1G52710 | Figura 2.I |
| ELO3 | Figura 2.I |
| AT5G56730 | Figura 2.I |
| AT5G57815 | Figura 2.I |
| TAF11b | Figura 2.I |
| AT4G38900 | Figura 2.I |
| CYCB1;3 | Figura 2.I |
| CYCA2;3 | Figura 2.I |
| HAG1 | Figura 2.I |
| TTN1 | Figura 2.I |
| AT5G23910 | Figura 2.I |
| SGF29b | Figura 2.I |
| SGF29a | Figura 2.I |
| AT2G21300 | Figura 2.I |
| AT5G16050 | Figura 2.I |
| MPPalpha | Figura 2.I |
| BPC2 | Figura 2.I |
| AT5G25450 | Figura 2.I |
| TAF12 | Figura 2.I |
| COX2 | Figura 2.I |
| FIB | Figura 2.I |
| AT5G58575 | Figura 2.I |
| CYCD7;1 | Figura 2.I |
| AT5G02220 | Figura 2.I |
| CYCB2;2 | Figura 2.I |
| PETD | Figura 2.I |
| AT5G40810 | Figura 2.I |
| COX15 | Figura 2.I |
| AT4G28060 | Figura 2.I |
| CDKB1;2 | Figura 2.I |
| CYCA3;4 | Figura 2.I |
| ICK3 | Figura 2.I |
| CYCD5;1 | Figura 2.I |
| KRP2 | Figura 2.I |
| CYCA3;1 | Figura 2.I |
| KRP6 | Figura 2.I |
| CYCD3;1 | Figura 2.I |
| AT1G32710 | Figura 2.I |
| CYCD3;3 | Figura 2.I |
| AT5G40460 | Figura 2.I |
| AT1G80230 | Figura 2.I |
| AT5G13440 | Figura 2.I |
| SEC10 | Figura 2.I |
| EXO70D1 | Figura 2.I |
| EXO70D3 | Figura 2.I |
| AT1G10385 | Figura 2.I |
| SEC1B | Figura 2.I |
| SYP112 | Figura 2.I |
| SEC5A | Figura 2.I |
| NTMC2T2.1 | Figura 2.I |
| EXO70H2 | Figura 2.I |
| SEC5B | Figura 2.I |
| VPS9B | Figura 2.I |
| EXO70H4 | Figura 2.I |
| EXO70E1 | Figura 2.I |
| EXO70H5 | Figura 2.I |
| KEU | Figura 2.I |
| SEC15A | Figura 2.I |
| ARC3 | Figura 2.J |
| ARC6 | Figura 2.J |
| AT1G07615 | Figura 2.J |
| AT1G11930 | Figura 2.J |
| AT1G33360 | Figura 2.J |
| AT1G73740 | Figura 2.J |
| AT2G16930 | Figura 2.J |
| AT2G25500 | Figura 2.J |
| AT3G08840 | Figura 2.J |
| AT3G56900 | Figura 2.J |
| AT3G57220 | Figura 2.J |
| AT4G04880 | Figura 2.J |
| AT4G26860 | Figura 2.J |
| AT5G10910 | Figura 2.J |
| AT5G15220 | Figura 2.J |
| AT5G42770 | Figura 2.J |
| AT5G49840 | Figura 2.J |
| AT5G66550 | Figura 2.J |
| CCB3 | Figura 2.J |
| CLPX | Figura 2.J |
| EMB269 | Figura 2.J |
| FAC1 | Figura 2.J |
| FTSZ1-1 | Figura 2.J |
| FTSZ2-1 | Figura 2.J |
| FTSZ2-2 | Figura 2.J |
| GPT | Figura 2.J |
| LpxC1 | Figura 2.J |
| LpxC2 | Figura 2.J |
| LpxC3 | Figura 2.J |
| LpxC4 | Figura 2.J |
| LpxC5 | Figura 2.J |
| MGD1 | Figura 2.J |
| MGD2 | Figura 2.J |
| MGDC | Figura 2.J |
| MIND | Figura 2.J |
| MINE1 | Figura 2.J |
| MURE | Figura 2.J |
| PARC6 | Figura 2.J |
| RNEE/G | Figura 2.J |
| RPL27 | Figura 2.J |
| SAMBA | Figura 2.J |
| TRANS11 | Figura 2.J |
| YLMG1-1 | Figura 2.J |
| YLMG1-2 | Figura 2.J |
| YLMG2 | Figura 2.J |
| MPK6 | Figura 2.E |
| ARF17 | Figura 2.E |
| SZF1 | Figura 2.E |
| F2P16.14 | Figura 2.E |
| AT5G19485 | Figura 2.E |
| AOC3 | Figura 2.E |
| CPK28 | Figura 2.E |
| AT4G29780 | Figura 2.E |
| JAZ5 | Figura 2.E |
| BCS1 | Figura 2.E |
| AT1G72340 | Figura 2.E |
| AT3G07920 | Figura 2.E |
| UCP5 | Figura 2.E |
| SWEETIE | Figura 2.E |
| TPL | Figura 2.E |
| F7H12 | Figura 2.E |
| IAA32 | Figura 2.E |
| ARI1 | Figura 2.E |
| IAA1 | Figura 2.E |
| AT3G16830.1 | Figura 2.E |
| ERF11 | Figura 2.E |
| VPS60.2 | Figura 2.E |
| AT1G53900.1 | Figura 2.E |
| AT3G01830 | Figura 2.E |
| AT5G38640 | Figura 2.E |
| AT1G48970 | Figura 2.E |
| VPS46.1 | Figura 2.E |
| AT2G14830 | Figura 2.E |
| AT1G53880 | Figura 2.E |
| AT2G44070 | Figura 2.E |
| AT3G02270 | Figura 2.E |
| AT3G07300 | Figura 2.E |
| AT2G34970 | Figura 2.E |
| OPCL1 | Figura 2.E |
| JAZ1 | Figura 2.E |
| WRKY33 | Figura 2.E |
| AT4G24380 | Figura 2.E |
| AT4G18300 | Figura 2.E |
| HPL1 | Figura 2.E |
| AT1G08270 | Figura 2.E |
| IAA29 | Figura 2.E |
| AT2G39650 | Figura 2.E |
| XK-2 | Figura 2.E |
| WRKY40 | Figura 2.E |
| AOS | Figura 2.E |
| SCL13 | Figura 2.E |
| WRKY18 | Figura 2.E |
| AT5G01940 | Figura 2.E |
| AT1G76110 | Figura 2.E |
| VPS46.2 | Figura 2.E |
| AT1G55650 | Figura 2.E |
| VPS60.1 | Figura 2.E |
| AT3G13350 | Figura 2.E |
| STZ | Figura 2.E |
| WSIP2 | Figura 2.E |
| AT1G04880 | Figura 2.E |
| AT3G02840 | Figura 2.E |
| MPK3 | Figura 2.E |
| AHP5 | Figura 2.H |
| ARR3 | Figura 2.H |
| ERS1 | Figura 2.H |
| ETR1 | Figura 2.H |
| CRF7 | Figura 2.H |
| SOB3 | Figura 2.H |
| CTR1 | Figura 2.H |
| MEE60 | Figura 2.H |
| ETR2 | Figura 2.H |
| CRF3 | Figura 2.H |
| CRF6 | Figura 2.H |
| CRF4 | Figura 2.H |
| PHYE | Figura 2.H |
| CRF5 | Figura 2.H |
| ERS2 | Figura 2.H |
| CRF2 | Figura 2.H |
| PDK | Figura 2.H |
| WOL | Figura 2.H |
| CRF1 | Figura 2.H |
| HP6 | Figura 2.H |
| RR22 | Figura 2.H |
| PHYC | Figura 2.H |
| CRF8 | Figura 2.H |
| AHP4 | Figura 2.H |
| AT4G04402 | Figura 2.H |
| HK2 | Figura 2.H |
| RR24 | Figura 2.H |
| RR1 | Figura 2.H |
| HK3 | Figura 2.H |
| ARR4 | Figura 2.H |
| PHYB | Figura 2.H |
| IAMT1 | Figura 2.H |
| AT1G15670 | Figura 2.H |
| AT3G59940 | Figura 2.H |
| AT3G28430 | Figura 2.H |
| ESC | Figura 2.H |
| AT2G44130 | Figura 2.H |
| AHP1 | Figura 2.H |
| AHP3 | Figura 2.H |
| AT3G61260 | Figura 2.H |
| MUO10.6 | Figura 2.H |
